# Mitochondrial SLC25A46 Rewires Fatty Acid Oxidation to Promote Cell Proliferation and Ferroptosis Evasion in Ovarian Cancer by Stabilizing CACT

**DOI:** 10.1002/advs.202523969

**Published:** 2026-07-20

**Authors:** Yunge Gao, Jiatao Hao, Xiaohong Zhang, Meiting Long, Yujing Feng, Xiaoyue Liu, Xuejian Li, Shujuan Liu, Xin Guo, Xiaohui Lv

**Affiliations:** ^1^ Department of Gynecology and Obstetrics Xijing Hospital Fourth Military Medical University Xi'an Shaanxi China; ^2^ Department of Obstetrics and Gynecology Xi'an People's Hospital (Xi'an Fourth Hospital) Xi'an Shaanxi China; ^3^ Department of General Surgery Xijing 986th Hospital Fourth Military Medical University Xi'an Shaanxi China

**Keywords:** CACT, fatty acid oxidation, MARCHF5, mitochondria, SLC25A46

## Abstract

SLC25A46 is a mitochondrial intermembrane bridging protein reported to play a crucial role in mitochondrial network maintenance, yet its functional roles in human cancer metabolic rewiring and disease progression remain unexplored, including ovarian cancer (OC). Here, we revealed that SLC25A46 is markedly upregulated in OC and associated with poor patient outcomes. Functionally, SLC25A46 promoted OC growth by facilitating cell proliferation and ferroptosis evasion. Mechanistically, SLC25A46 promotes cell proliferation and ferroptosis evasion of OC cells by activating fatty acid oxidation‐mediated ATP and NADPH production via protecting carnitine‐acylcarnitine translocase (CACT) from MARCHF5‐mediated ubiquitin‐degradation. Notably, knockdown of SLC25A46 significantly increased the sensitivity of OC cells to ferroptosis and enhanced their cytotoxic response to carboplatin. Additionally, we found that PBX1 directly binds and transactivates the SLC25A46 promoter. Overall, our results highlight the critical role of SLC25A46/MARCHF5/CACT axis in facilitating cell proliferation and ferroptosis evasion in OC cells via activating fatty acid oxidation‐mediated ATP and NADPH production. These findings suggest that targeting SLC25A46 represents a rational strategy to improve treatment outcomes in OC patients.

## Graphical abstract

Mitochondrial SLC25A46 is upregulated in ovarian cancer (OC) and correlates with poor prognosis. SLC25A46 stabilizes CACT to activate fatty acid oxidation, increasing ATP and NADPH production to drive cell proliferation and ferroptosis evasion. SLC25A46 silencing sensitizes OC cells to ferroptosis and carboplatin, offering a promising therapeutic strategy.

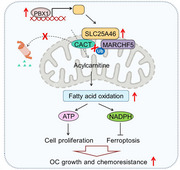

## 1 Introduction

Ovarian cancer (OC) ranks first among gynecologic malignancies in lethality worldwide [1]. Owing to the lack of distinctive symptoms in the early course of this malignancy, most cases are diagnosed at an advanced stage [2]. Despite incremental gains in surgery, chemotherapy, and targeted therapy, the 5‐year survival rate for OC remains unsatisfactory, largely due to the development of chemotherapy resistance and a high rate of tumor recurrence [3]. Ferroptosis denotes an iron‐catalyzed, lipid‐peroxidation‐driven form of regulated necrosis [4]. Mounting evidence suggests the contribution of ferroptosis to OC progression and chemotherapy resistance [5, 6, 7, 8]. Therefore, elucidating the molecular determinants of ferroptosis threshold may yield vulnerability nodes exploitable to improve the treatment outcomes of patients with OC.

Mitochondrial dysfunction has been associated with the pathogenesis of various human diseases, including cancer [9]. Solute carrier family 25 member A46 (SLC25A46) is a mitochondrial intermembrane bridging protein that has been reported to play a crucial role in mitochondrial network maintenance through interactions with fusion/fission machineries such as optic atrophy 1 (OPA1) and mitofusin 2 (MFN2) [10]. These findings suggest that SLC25A46 may play a critical role in modulating mitochondrial function. However, the functional role of SLC25A46 in human cancer metabolic rewiring and disease progression remains unexplored, including OC.

In the current study, we establish SLC25A46 up‐regulation as a metabolic switch that fuels OC growth by facilitating cell proliferation and conferring ferroptosis resistance via activating CACT‐modulated fatty acid oxidation, which leads to increased ATP and NADPH production. Especially, silencing SLC25A46 markedly lowers the ferroptosis threshold of OC cells and enhances their cytotoxic response to carboplatin. Collectively, our data suggest that targeting SLC25A46 represents a rational strategy to improve treatment outcomes in OC patients.

## 2 Results

### 2.1 SLC25A46 Was Markedly Upregulated in OC and Associated With Poor Patient Outcomes

**FIGURE 1:**
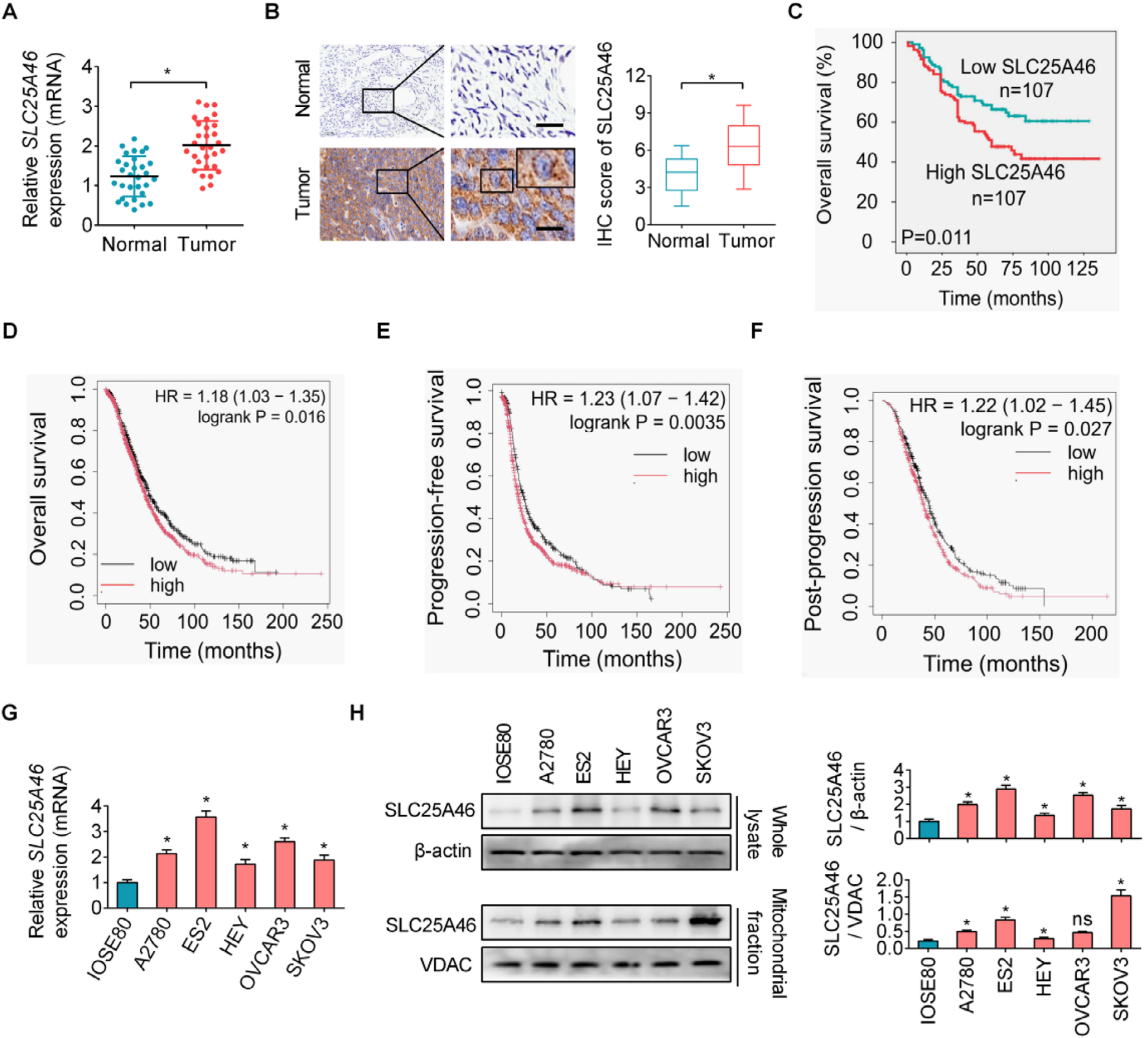
SLC25A46 was markedly upregulated in OC and associated with poor patient outcomes. (A, B) In 30 postoperative OC specimens and matched adjacent normal tissue by qRT‐PCR (panel A) and in another larger cohort (*n* = 214) by IHC assay (panel B, Scale bar = 20 µm). Statistical significance was determined by a paired Student's *t*‐test. (C) IHC‐derived staining scores stratified patients for Kaplan–Meier survival analysis. (D–F) Correlation between the expression of SLC25A46 and OS (panel D), PFS (panel E), and PPS (panel F) was analyzed by bioinformatics analyses using the online Kaplan–Meier Plotter database. (G, H) SLC25A46 expression was detected in both whole cell lysates and mitochondrial fraction derived from ovarian cancer and normal ovarian epithelial cell lines by qRT‐PCR (panel G) and by Western blotting (panel H, the upper for SLC25A46 expression in whole cell lysate, while the bottom for mitochondrial SLC25A46 expression). For in vitro assays (panels G, H), three independent biological replicates were performed, each consisting of three technical replicates. Data are presented as mean ± SD. Statistical analysis was performed using one‐way ANOVA followed by Tukey's post hoc test. ^*^ *p* < 0.05.

To explore the role of SLC25A46 in ovarian cancer (OC), its expression pattern was first assessed in 30 postoperative OC specimens and matched adjacent normal tissue by qRT‐PCR analysis. SLC25A46 expression is clearly higher in OC specimens compared to paired normal tissues (Figure 1A). To validate this result, immunohistochemistry (IHC) assay was carried out in an extended cohort (*n* = 214), which consistently revealed markedly increased SLC25A46 expression in OC (Figure 1B). Correlation analysis indicates a significant positive association between the expression of SLC25A46 and tumor size (Table S1). Kaplan–Meier survival analysis indicated that high SLC25A46 expression in OC patients was associated with worse overall survival (Figure 1C). Consistent with the results in our cohort, bioinformatics analyses using the online Kaplan–Meier Plotter database [11] corroborated our findings, linking elevated SLC25A46 to shorter overall survival (OS), progression‐free survival (PFS), and post‐progression survival (PPS) (Figure 1D–F). OC cell lines recapitulated tissue data, exhibiting pronounced SLC25A46 up‐regulation in both whole‐cell and mitochondrial fractions vs. normal ovarian epithelial controls (Figure 1G,H). To confirm whether SLC25A46 is localized in mitochondria, immunofluorescence staining (IF) analysis was conducted in ES2 and SKOV3 cells expressing relative high mitochondrial SLC25A46. We observed a significant co‐localization of SLC25A46 with VDAC (a mitochondrial marker) in ES2 and SKOV3 cells (Figure S1A), implying that SLC25A46 mainly functions in the mitochondria of OC cells. These findings suggest SLC25A46, mainly expressed in mitochondrial, as a frequently upregulated, survival‐associated candidate in OC. Moreover, significant upregulation of SLC25A46 was also observed in several other cancer types, as revealed by bioinformatics analysis using the online Sangerbox database [12] (Figure S1B), suggesting a frequent event of SLC25A46 upregulation in human cancers.

### 2.2 SLC25A46 Promotes OC Growth by Facilitating Cell Proliferation and Suppressing Cell Death

**FIGURE 2:**
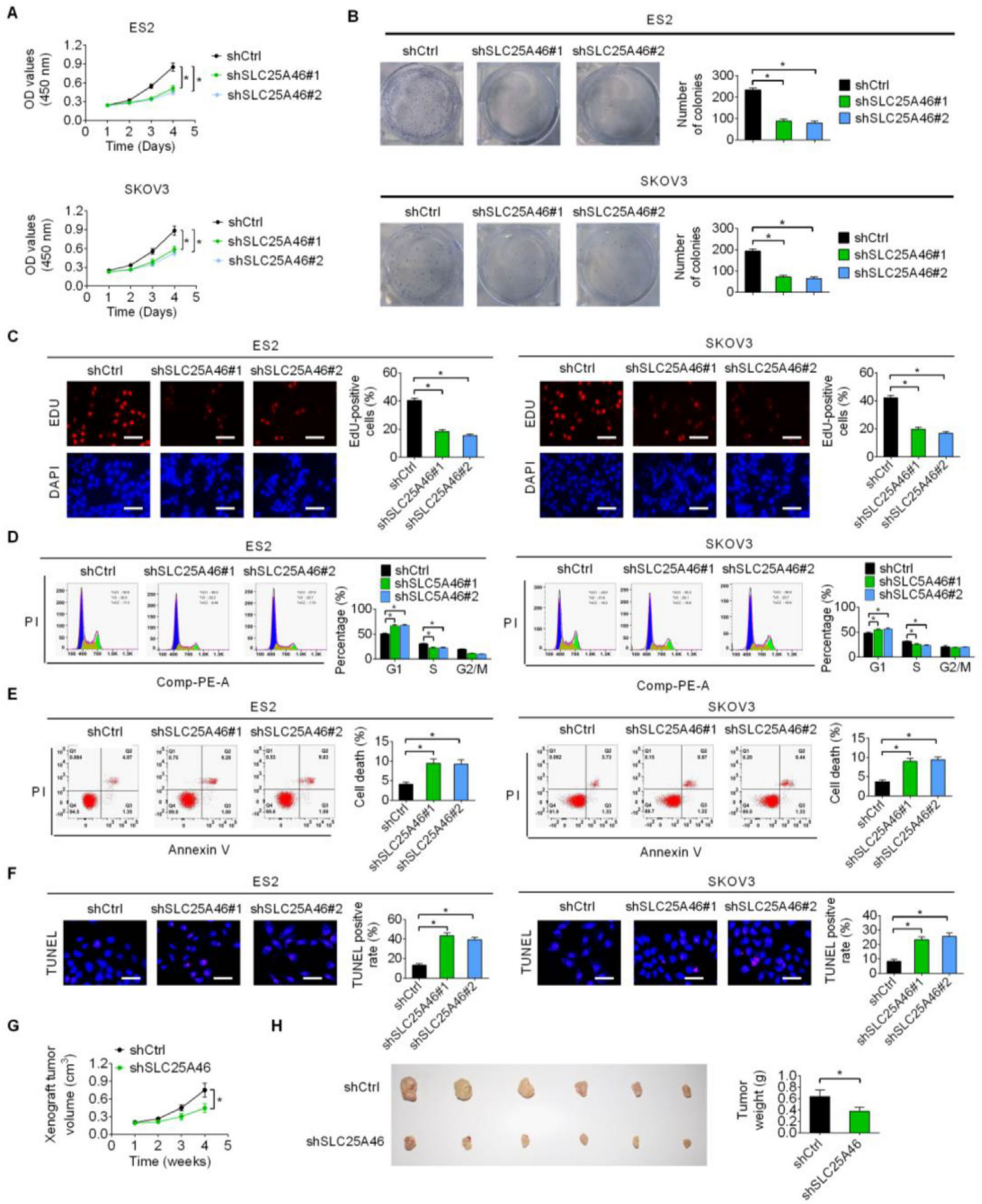
SLC25A46 promotes OC growth by facilitating cell proliferation and suppressing cell death. (A, B) Proliferative impact of SLC25A46 knockdown was assessed by CCK‐8 cell viability (panel A) and colony formation (panel B) assays. (C, D) EdU incorporation (panel C, Scale bar = 20 µm) and cell cycle analysis by flow cytometry (panel D) assays were used to examine the impact of SLC25A46 knockdown on cell cycle progression in OC cells. The percentage of EdU‐positive cells was calculated within five randomly selected fields per well as the ratio of EdU‐positive nuclei to DAPI‐stained nuclei and multiplied by 100%. (E, F) Annexin V/PI staining (panel E) and TUNEL (panel F, Scale bar = 20 µm) assays were used to examine the impact of SLC25A46 knockdown on the death of OC cells. (G, H) The effect of SLC25A46 knockdown on tumor growth of OC was evaluated utilizing a xenograft tumor model (Panel G shows tumor growth curves and Panel H shows xenograft tumors and their weights). For in vitro assays (panels A–F), three independent biological replicates were performed, each consisting of three technical replicates. Non‐targeting scrambled shRNA (shCtrl) was used as controls. Data are presented as mean ± SD. Statistical analysis was performed using Student's *t*‐test or two‐way ANOVA followed by Tukey's post hoc test or Bonferroni post hoc test. ^*^, *p* < 0.05.

The close link between SLC25A46 expression and disease progression and patient outcomes suggests that SLC25A46 may function as a potential oncogene in OC. To figure out the function of SLC25A46 in OC cells, ES2 and SKOV3 cells with low mitochondrial SLC25A46 expressions were selected for silencing experiments (Figure S2A,B). CCK‐8 cell proliferation and colony formation assays revealed that SLC25A46 knockdown obviously inhibited cell proliferation (Figure 2A,B). In the meantime, EdU and cell‐cycle flow cytometry assays showed inhibited cell cycle progression upon SLC25A46 silencing (Figure 2C,D). In agreement with this, positive correlations between expressions of SLC25A46 and key regulators driving cell cycle progression were revealed using the GEPIA database (Figure S2C). Subsequent Annexin V/PI staining and TUNEL assays indicated that SLC25A46 knockdown markedly increased cell death in OC (Figure 2E,F). However, migratory and invasive capacities remained unaltered when SLC25A46 was knocked‐down (Figure S2D,E).

To confirm the observations from in vitro studies, the impact of SLC25A46 knockdown on OC growth was evaluated utilizing a xenograft tumor model. Knockdown of SLC25A46 expression in ES2 cells (Figure S3A) notably slowed OC growth in nude mice, as revealed by significantly decreased tumor volumes and weights (Figure 2G,H), while it had no notable effect on lung metastasis (Figure S3B). IHC staining assay showed that downregulation of SLC25A46 was persisted in tissues of SLC25A46‐silenced xenografts (Figure S3C). Meanwhile, the expressions of Ki‐67 and PCNA were obviously reduced in the SLC25A46‐silenced group as compared to the control group (Figure S3D,E). These findings suggest that SLC25A46 promotes OC growth by both accelerating cell proliferation and suppressing cell death.

To provide more evidence for the modulation of cell proliferation by SLC25A46 in OC cells, HEY and OVCAR3 cell lines with relative low mitochondrial SLC25A46 level were chosen for gain‐of‐function studies (Figure S4A,B). CCK‐8 cell proliferation and colony formation assays indicated that forced SLC25A46 expression significantly augmented OC cell proliferation (Figure S4C,D). EdU incorporation and Annexin V/PI staining assays revealed markedly accelerated cell cycle progression and suppressed cell death in HEY and OVCAR3 cells upon SLC25A46 overexpression (Figure S4E,F). In line with these in vitro results, forced SLC25A46 expression markedly promoted OC growth in xenograft tumor models in vivo, as evidenced by increased tumor volumes and weights (Figure S4G,H). Nonetheless, in vitro migratory and invasive capacities, and in vivo lung metastasis remained unaltered when SLC25A46 was overexpressed (Figure S4I–K). Together, these data provide more evidence for the crucial role of SLC25A46 in promoting OC growth by facilitating cell proliferation and suppressing cell death.

### 2.3 SLC25A46 Suppressed Ferroptosis in Ovarian Cancer Cells

**FIGURE 3:**
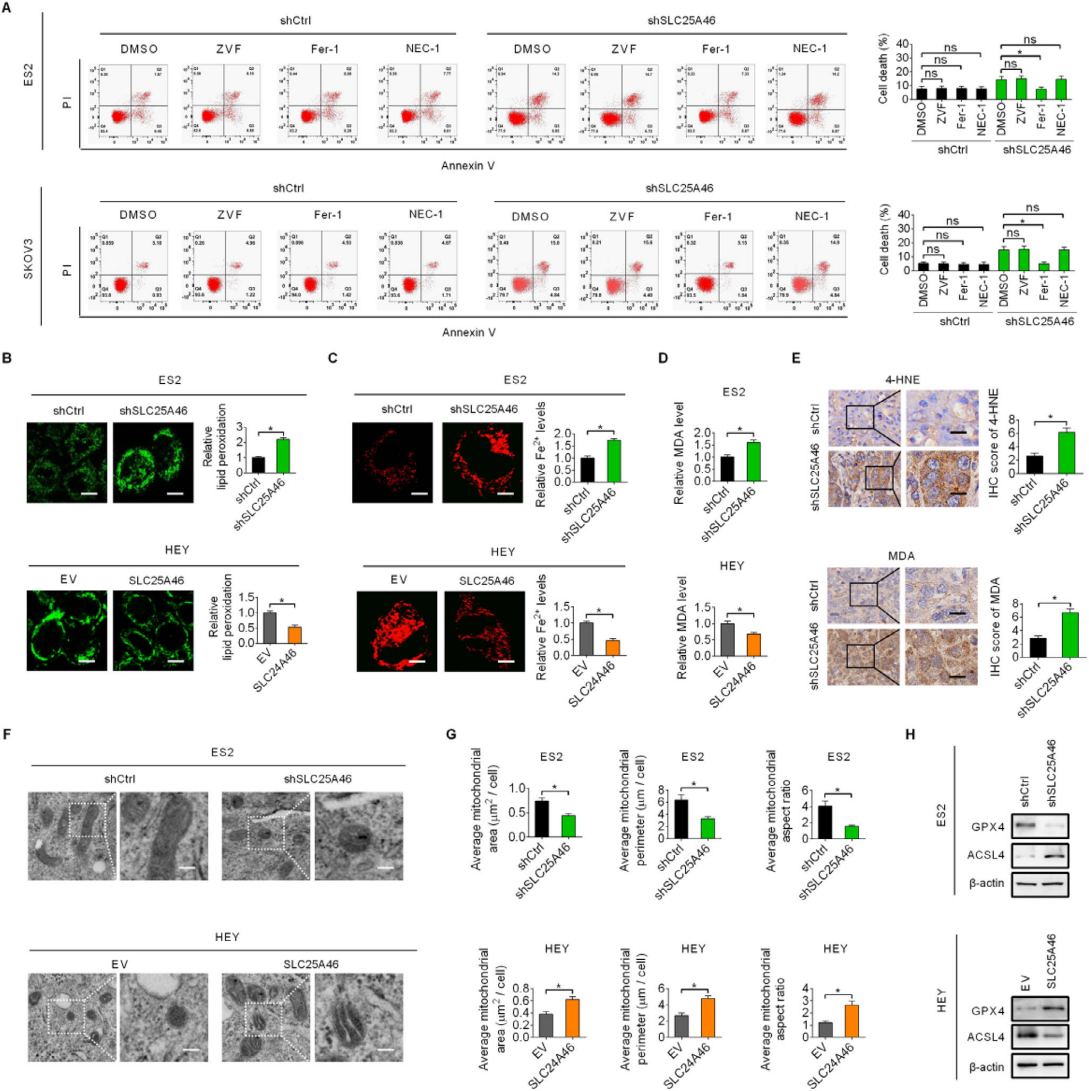
SLC25A46 suppressed ferroptosis in OC cells. (A) Cell death was analyzed by flow cytometry in SLC25A46‐silenced OC cells treated with inhibitors targeting different PCD pathways (10 µm ZVF for 12 h to inhibit apoptosis; 1 µm Fer‐1 for 12 h to inhibit ferroptosis; 10 µm NEC‐1 for 12 h to inhibit necroptosis). (B–D) The influence of SLC25A46 on the levels of lipid peroxidation (panel B), Fe^2+^ (panel C), and MDA (panel D) was evaluated in OC cells (Scale bar = 5 µm). (E) IHC staining of 4‐HNE and MDA in SLC25A46‐silenced xenograft tumor tissues (Scale bar = 10 µm). (F) Transmission electron microscopy (TEM) images of SLC25A46‐silenced and overexpressed OC cells. (G) Quantitative analysis of TEM results for average mitochondrial surface area, perimeter, and aspect ratio. (H) Expressions of GPX4 and ACSL4 were detected in OC cells when SLC25A46 was knocked‐down or overexpressed by Western blot. For in vitro assays (panels A‐D, F‐H), three independent biological replicates were performed, each consisting of three technical replicates. Non‐targeting scrambled shRNA (shCtrl) or empty plasmid (EV) was used as controls. Data are presented as mean ± SD. Statistical significance was analyzed by one‐way or two‐way ANOVA with Tukey's post hoc test. ^*^, *p* < 0.05; ns, not significant.

In recent years, various unique forms of programmed cell death (PCD) have been recognized [13]. To further delineate the type of inhibited PCD by SLC25A46 in OC, we treated SLC25A46‐silenced OC cells with inhibitors targeting different PCD pathways. The results showed that induction of cell death by SLC25A46 silencing was only mitigated by the Fer‐1, a ferroptosis inhibitor ferrostatin‐1(Fer‐1), whereas treatment with inhibitors targeting other PCD pathways had no notable impact on SLC25A46‐modulated cell death (Figure 3A and Figure S5A). This observation implies that SLC25A46 may suppress ferroptosis in OC cells. To confirm this, the influence of SLC25A46 on the levels of lipid peroxidation, Fe^2+^, and malondialdehyde (MDA) was evaluated in OC cells. SLC25A46 knockdown markedly increased lipid peroxidation, Fe^2+^, and MDA levels, while overexpression of SLC25A46 decreased these levels (Figure 3B–D). Similarly, IHC staining indicated that the levels of peroxidation markers of 4‐Hydroxynonenal (4‐HNE) and Malondialdehyde (MDA) were markedly upregulated in SLC25A46‐silenced xenograft tumor tissues compared to the controls (Figure 3E). Transmission electron microscopy assays revealed a significant decrease in average mitochondrial area, average mitochondrial perimeter, and average mitochondrial aspect ratio, which has been observed during ferroptosis [14] (Figure 3F,G). Consistently, SLC25A46 knockdown also decreased GPX4 expression and increased ACSL4 expression, whereas SLC25A46 overexpression yielded opposite results (Figure 3H), providing further supporting evidence for the inhibitory effect of SLC25A46 on ferroptosis in OC cells.

### 2.4 SLC25A46 Activates Fatty Acid Oxidation to Increase ATP and NADPH Production in OC Cells

**FIGURE 4:**
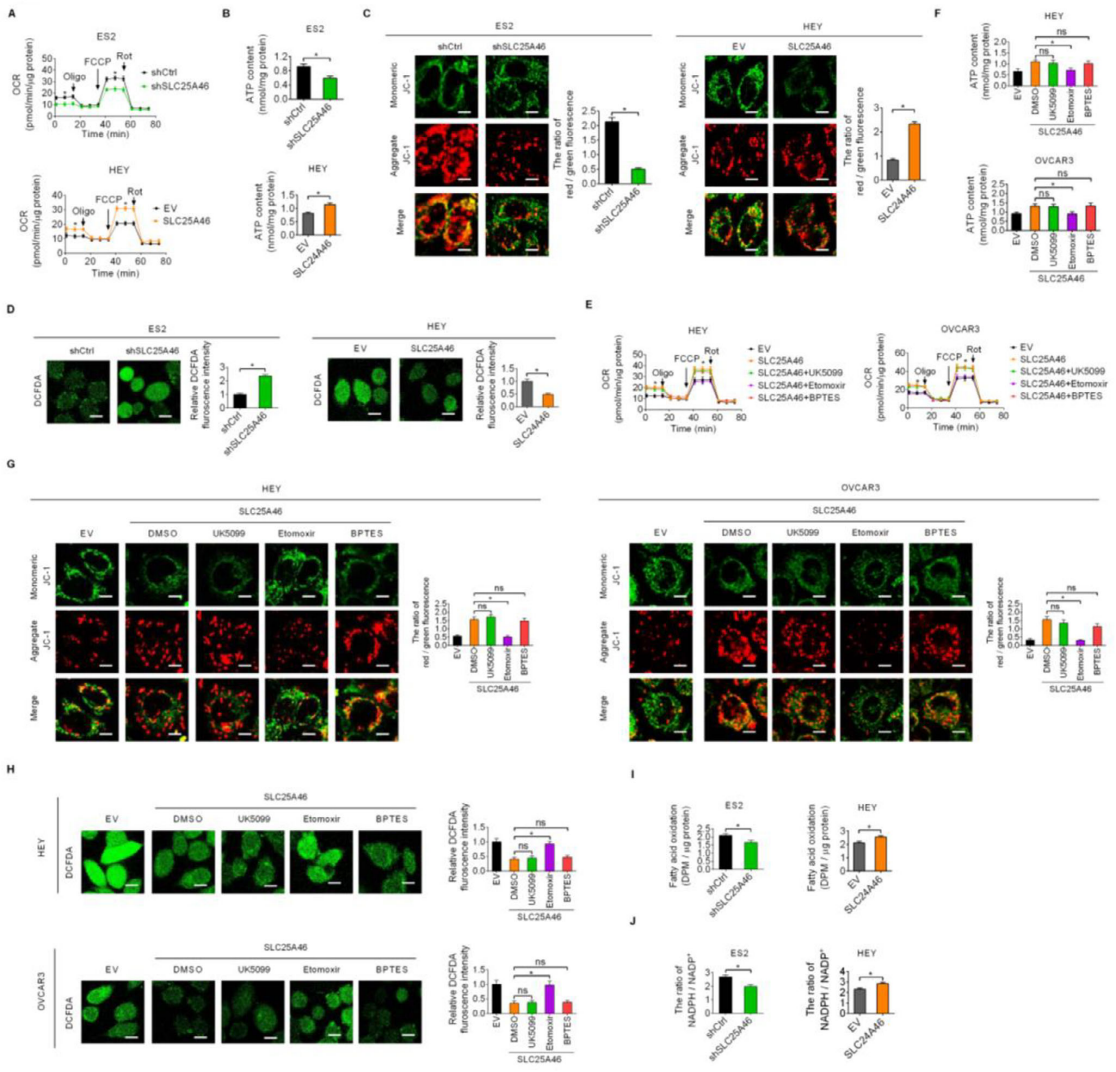
SLC25A46 activates fatty acid oxidation to increase ATP and NADPH production in OC cells. (A–D) Mitochondrial OCR (panel A), ATP production (panel B), membrane potential (panel C), and ROS production (panel D) were measured in OC cells when SLC25A46 was knocked‐down or overexpressed (Scale bar = 5 µm). (E–H) Mitochondrial OCR (panel E), ATP production (panel F), membrane potential (panel G) and ROS production (panel H) were measured in SLC25A46‐overexpressing OC cells treated with inhibitors of FAO, glycolysis or glutaminolysis (UK5099, 20 µm for 24 h; Etomoxir, 100 µm for 24 h; BPTES, 5 µm for 24 h) (Scale bar = 5 µm). (I) FAO was evaluated using ^3^H‐labeled oleic acid as a tracer in OC cells when SLC25A46 was knocked‐down or overexpressed. (J) NADPH level was evaluated in OC cells when SLC25A46 was knocked‐down or overexpressed. All experiments were performed three biologically independent replicates, each comprising three technical replicates. Non‐targeting scrambled shRNA (shCtrl) or empty plasmid (EV) was used as controls. Data are presented as mean ± SD. Statistical analysis was performed using Student's *t*‐test or one‐way ANOVA followed by Tukey's post hoc test. ^*^, *p* < 0.05; ns, not significant.

Next, the mechanism of SLC25A46 in inhibiting ferroptosis in OC cells was investigated. Its mitochondrial scaffolding function [15], we proposed that upregulation of SLC25A46 may affect mitochondrial metabolism, which probably suppresses ferroptosis in OC cells. To validate this hypothesis, the impact of SLC25A46 on mitochondrial metabolism was assessed by measuring mitochondrial oxygen consumption rate (OCR), ATP synthesis, membrane potential, and reactive oxygen species (ROS) generation. The results revealed a significant reduction in mitochondrial OCR, ATP synthesis, and membrane potential, and an increase in ROS production in the SLC25A46‐silenced OC cells. On the contrary, overexpression of SLC25A46 markedly enhanced these mitochondrial bioenergetic profiles, while reduced ROS production (Figure 4A–D). These results suggest that SLC25A46 promotes mitochondrial metabolic activity in OC cells. To elucidate the source of enhanced mitochondrial metabolism by SLC25A46, the principal metabolic pathways (glucose, fatty acid, and glutamine) providing substrates for mitochondrial metabolism were suppressed by specific inhibitors in SLC25A46‐overexpressing OC cells. The results indicated that the enhancement of mitochondrial metabolism and reduction of ROS production by SLC25A46 were significantly reversed upon the suppression of fatty acid oxidation (FAO) by etomoxir, whereas suppressions of glycolysis and glutaminolysis had no notable effect on those phenotypes (Figure 4E–H), indicating that SLC25A46 may enhance mitochondrial metabolism by activating FAO. To validate this, we evaluated the role of SLC25A46 in FAO. As indicated in Figure 4I, FAO was markedly suppressed by SLC25A46 knockdown, while enhanced by SLC25A46 overexpression. Consistent with expectations, the concentration of nicotinamide adenine dinucleotide phosphate hydrogen (NADPH), which is a byproduct of FAO, decreased following the knockdown of SLC25A46, while it increased with the overexpression of SLC25A46 (Figure 4J). Moreover, although the protein levels of key antioxidant enzymes SOD2, CAT, and GPX1 were not significantly altered by either SLC25A46 knockdown or overexpression (Figure S5B), the GSSG/GSH ratio was increased by SLC25A46 knockdown and decreased by SLC25A46 overexpression (Figure S5C), indicating that SLC25A46 enhances the antioxidant capacity primarily by upregulating fatty acid oxidation‐mediated NADPH production.

To dissect the lipid sources fueling SLC25A46‐driven FAO, we used BODIPY 493/503 to stain lipid droplets and found that lipid droplet abundance was significantly increased by SLC25A46 knockdown and decreased by SLC25A46 overexpression (Figure S5D). Furthermore, we performed rescue experiments using inhibitors targeting distinct lipid supply routes. Treatment with the fatty acid uptake inhibitor sulfo‐N‐succinimidyl oleate (SSO), but not de novo lipogenesis inhibitor 5‐(Tetradecyloxy)‐2‐furoic acid (TOFA), significantly blunted the elevation in ATP production induced by SLC25A46 overexpression (Figure S5E). These results suggest that SLC25A46 supports enhanced FAO mainly by facilitating lipid droplet utilization and fatty acid uptake. Together, these findings suggest that SLC25A46 activates FAO to produce more energy and NADPH in OC cells.

### 2.5 SLC25A46 Promotes FAO and Thus Cell Proliferation and Ferroptosis Evasion of OC Cells by Upregulating CACT Expression

**FIGURE 5:**
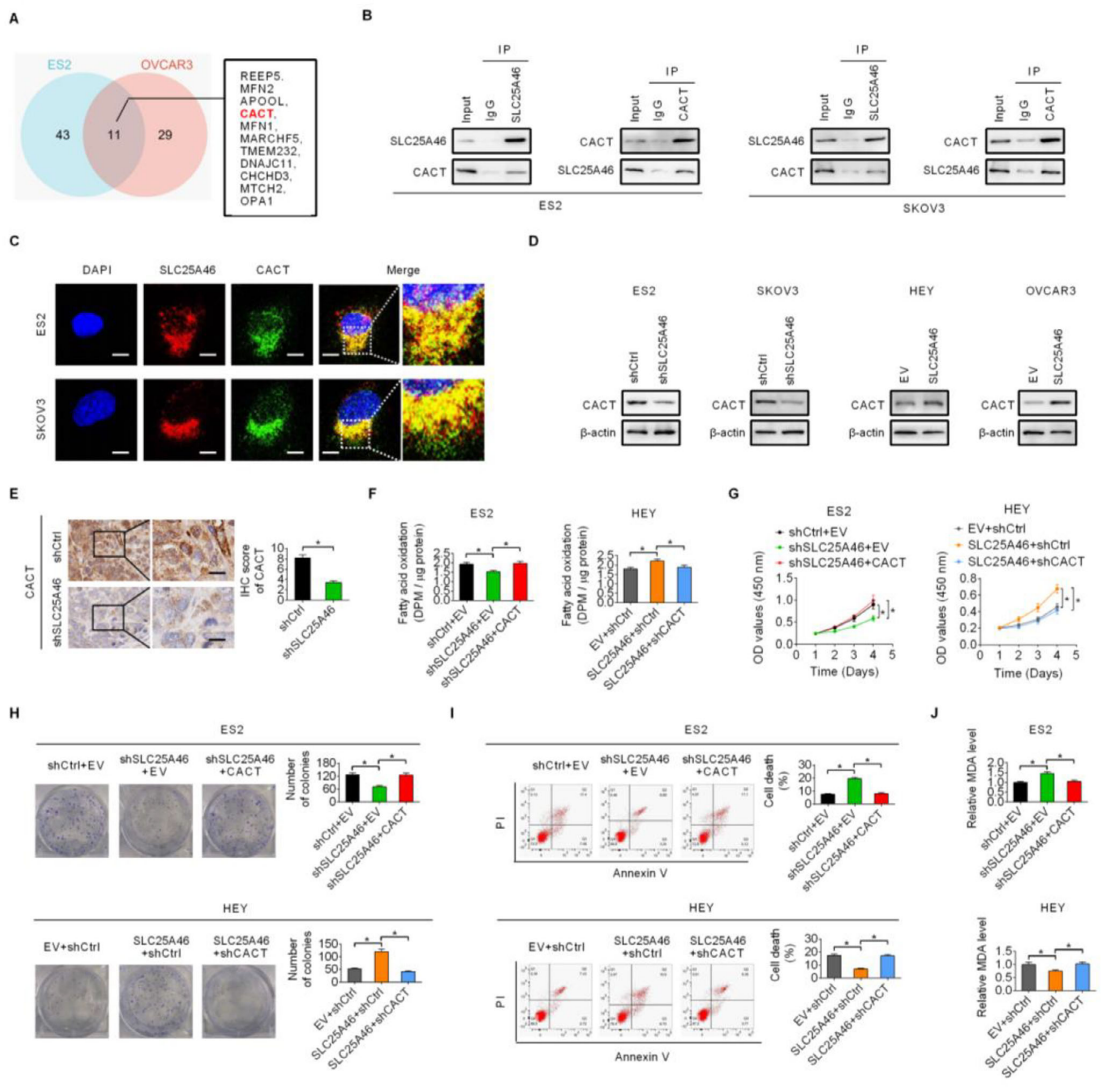
SLC25A46 promotes FAO and thus cell proliferation and *ferroptosis evasion* of OC cells by upregulating CACT expression. (A) Mass‐spectrometry (MS) analysis was used to identify SLC25A46‐interacting proteins in two OC cell lines. (B) Co‐IP assay SLC25A46 and CACT interaction in ES2 cells. (C) Immunofluorescent staining of SLC25A46 and CACT in ES2 and SKOV3 cells (Scale bar = 5 µm). (D) The impact of SLC25A46 on CACT protein level was determined using a Western blotting assay. (E) IHC staining of CACT in SLC25A46‐silenced xenograft tumor tissues (Scale bar = 10 µm). (F–J) A series of rescue assays was conducted to explore the involvement of CACT in SLC25A46‐modulated FAO, cell proliferation, and ferroptosis (panel F, FAO assay; panel G, CCK‐8 assay; panel H, Colony formation assay; panel I, Annexin V/PI staining assay; panel J, MDA level). For in vitro assays (panels B–D, F–J), three independent biological replicates were performed, each consisting of three technical replicates. Non‐targeting scrambled shRNA (shCtrl) or / and empty plasmid (EV) were used as controls. Data are presented as mean ± SD. Statistical analysis was conducted using one‐way ANOVA with Tukey's post hoc test. ^*^, *p* < 0.05.

Accumulating evidence suggests SLC25A46 as an intermembrane bridging protein in the mitochondrial outer membrane. To explore the molecular mechanisms through which SLC25A46 facilitates FAO in OC cells, the interactors of SLC25A46 were profiled by mass‐spectrometry (MS) analysis. Among the 11 identified SLC25A46‐interacting proteins, MFN1/2 and OPA1 have been previously reported to form complexes with SLC25A46. Strikingly, carnitine‐acylcarnitine translocase (CACT), a rate‐limiting gatekeeper of β‐oxidation located in IMM, was found to interact with SLC25A46 (Figure 5A). Consequently, we selected CACT for further investigation. Co‐IP assay demonstrated that SLC25A46 directly binds with CACT in ES2 and SKOV3 cells (Figure 5B). Consistently, significant spatial overlap of SLC25A46 and CACT was also observed in OC cells by immunofluorescent staining assay (Figure 5C). We next determined whether SLC25A46 could regulate the expression of CACT, and demonstrated that SLC25A46 knockdown reduced CACT protein without altering transcript abundance, whereas its overexpression increased the protein level of CACT (Figure 5D and Figure S6A). We also observed a marked reduction of CACT expression in SLC25A46 knockdown xenograft tissues compared with the control (Figure 5E). Next, the involvement of CACT in SLC25A46‐modulated FAO cell proliferation and ferroptosis evasion was evaluated. Upregulation of CACT markedly rescued the suppressions of FAO, proliferation, and ferroptosis evasion caused by SLC25A46 silencing in OC cells. By contrast, CACT silencing significantly abolished the activations of FAO, cell proliferation, and ferroptosis evasion caused by SLC25A46 upregulation (Figure 5F–J). These findings suggest that SLC25A46 promotes FAO and thereby OC progression by upregulating CACT protein expression.

### 2.6 SLC25A46 Upregulates CACT by Protecting CACT From MARCHF5‐mediated Proteasomal Degradation

**FIGURE 6:**
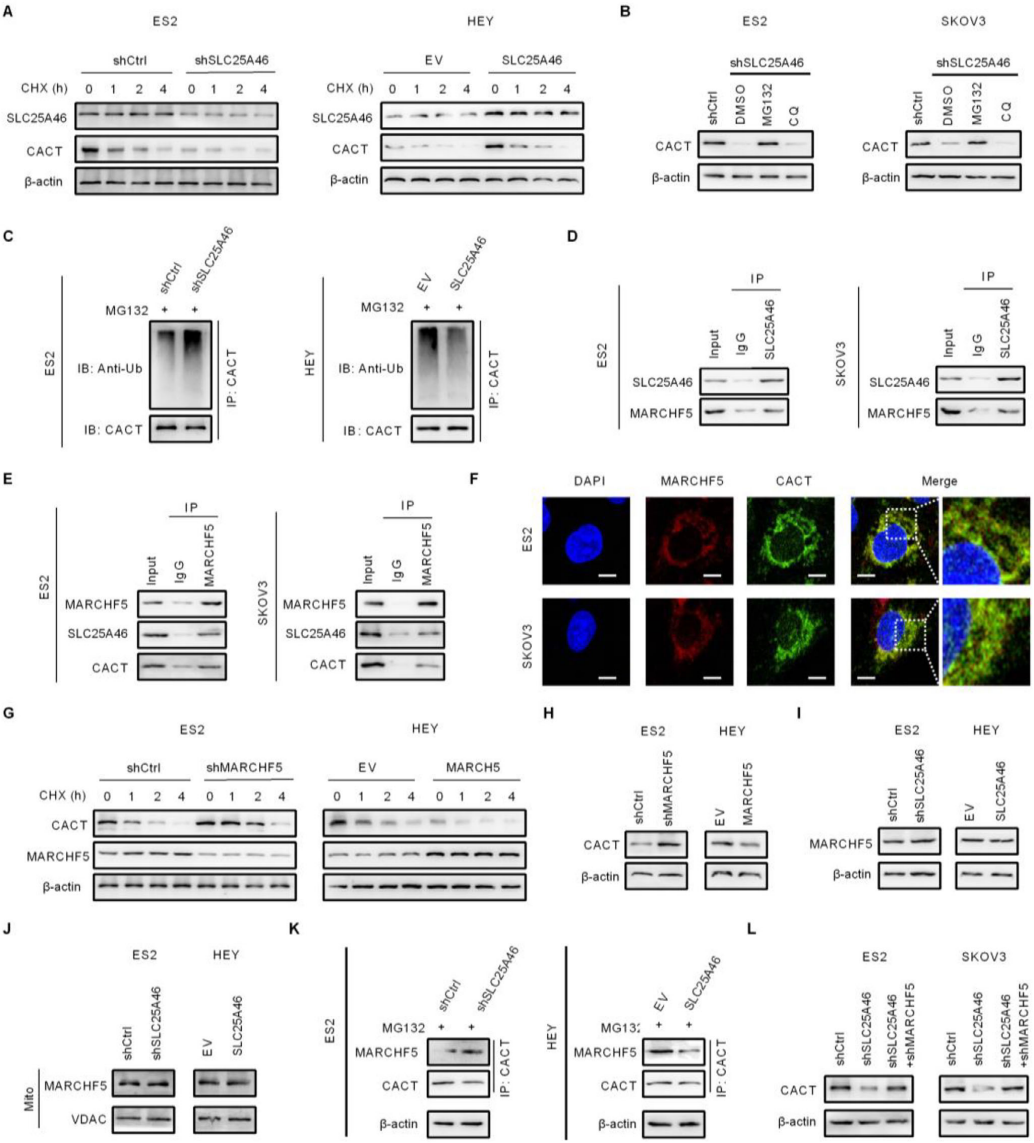
SLC25A46 upregulates CACT by protecting CACT from MARCHF5‐mediated proteasomal degradation. (A) The effect of SLC25A46 on the stability of CACT protein was evaluated in OC cells. (B) CACT expression was detected in SLC25A46‐silenced OC cells treated with CQ (20 µm for 24 h) or MG132 (10 µm for 24 h). (C) The influence of SLC25A46 on CACT ubiquitination was determined by Western blotting assay. (D, E) CO‐IP assay was carried out to determine SLC25A46 and MARCHF5 interaction (panel D), as well as interaction between MARCHF5 and CACT (panel E). (F) Immunofluorescent staining assay was conducted to confirm the co‐localization of MARCHF5 and CACT in ES2 and SKOV3 cells (Scale bar = 5 µm). (G, H) The effect of MARCHF5 on the expression (G) and stability (H) of CACT protein was evaluated in OC cells. (I, J) The effect of SLC25A46 on total cellular (panel I) or mitochondrial (panel J) MARCHF5 was evaluated by Western blotting assay. (K) The effect of SLC25A46 silencing or overexpression on the interaction between MARCHF5 and CACT was evaluated by CO‐IP assay in OC cells. (L) CACT expression was detected by Western blotting assay in OC cells subjected to specified treatments. All experiments included three independent biological replicates, each comprising three technical replicates.

To explore how SLC25A46 affects the protein expression of CACT, the change in stability of CACT was analyzed upon SLC25A46 silencing or overexpression in OC cells with protein synthesis suppressed by cycloheximide (CHX) treatment. The results showed that SLC25A46 knockdown reduced the stability of CACT protein in ES2 cells, whereas overexpression of SLC25A46 increased the stability of CACT in HEY cells (Figure 6A). This suggests that SLC25A46 inhibits the degradation of CACT in OC cells. To further explore the pathway by which SLC25A46 upregulates CACT protein stability, we treated SLC25A46‐silenced OC cells with chloroquine (CQ) or MG132, which are two inhibitors targeting lysosomal and proteasomal degradation pathways, respectively. As shown in Figure 6B, MG132 treatment obviously prevented the downregulation of CACT by SLC25A46 silencing, while CQ treatment had no notable effect on CACT expression decreased by SLC25A46 silencing, suggesting that the ubiquitin‐proteasome pathway is the primary degradation route for CACT in OC. As expected, we observed that the ubiquitination level of CACT was elevated upon SLC25A46 silencing, while reduced upon SLC25A46 overexpression (Figure 6C). These findings suggest that SLC25A46 upregulates CACT expression by protecting CACT from ubiquitination‐mediated proteasomal degradation in OC cells.

To acquire a deeper understanding of the mechanisms governing the protection of CACT from proteasomal degradation by SLC25A46, we reanalyzed the IP‐MS results and found Membrane Associated Ring‐CH‐Type Finger 5 (MARCHF5), a critical mitochondrial outer membrane‐localized E3 ubiquitin‐protein ligase [16], as a SLC25A46 interacting protein, which was validated by CO‐IP assay (Figure 6D). Meanwhile, the CO‐IP and immunofluorescent co‐staining experiments also revealed a clear interaction and co‐localization between MARCHF5 and CACT (Figure 6E,F and Figure S6B). Assessment of the effect of MARCHF5 on CACT expression revealed that overexpression of MARCHF5 resulted in a significant decrease in CACT expression, whereas knockdown of MARCHF5 led to a marked increase in CACT expression (Figure 6G). Correspondingly, we observed that the stability of CACT protein was increased by MARCHF5 knockdown and reduced by MARCHF5 overexpression (Figure 6H), suggesting MARCHF5 as a critical regulator of CACT protein stability. Next, we explored whether MARCHF5 contributes to the upregulation of CACT by SLC25A46. Analysis of the effect of SLC25A46 on MARCHF5 expression revealed that neither total cellular MARCHF5 nor mitochondrial MARCHF5 was significantly affected by SLC25A46 (Figure 6I,J), while the interaction between MARCHF5 and CACT was enhanced by SLC25A46 silencing but weakened by SLC25A46 overexpression (Figure 6K and Figure S6C). In agreement with this, the downregulation of CACT by SLC25A46 silencing was obviously attenuated by MARCHF5 knockdown (Figure 6L). These results collectively indicate that SLC25A46 upregulates CACT by protecting it from MARCHF5‐mediated proteasomal degradation.

### 2.7 SLC25A46 Expression is Positively Correlated With CACT and Upregulation of CACT Contributes to the Progression of OC

**FIGURE 7:**
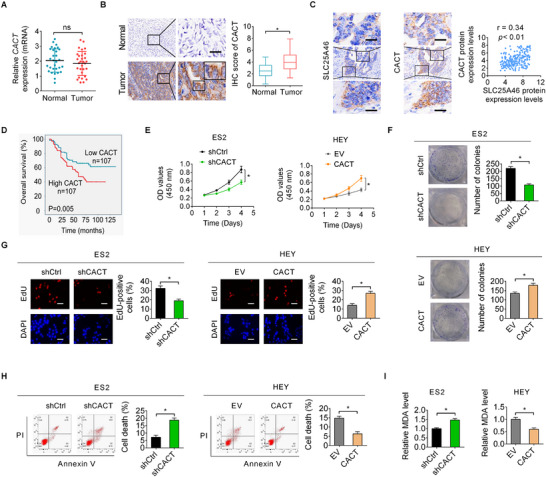
SLC25A46 expression is positively correlated with CACT, and upregulation of CACT contributes to the progression of OC. (A, B) qRT‐PCR (panel A) and IHC staining (panel B, Scale bar = 20 µm) assays for CACT expression levels in postoperative OC and corresponding normal tissues from OC patients (*n* = 214). (C) The relationship between the expression levels of SLC25A46 and CACT was analyzed in clinical OC tissues (Left panel, representative IHC images of SLC25A46 and CACT of the same fields from consecutive tissue sections show that regions with strong SLC25A46 staining also exhibited strong CACT staining, and vice versa. Scale bar = 20 µm; Right panel, Spearman's rank correlation analysis based on the IHC staining results). (D) Kaplan–Meier survival analysis for the association between CACT expression and patient survival in OC. (E–G) CCK‐8 (panel E), colony formation (panel F), and EdU (panel G, Scale bar = 20 µm) assays were conducted in CACT silencing or overexpression OC cells. (H) Annexin V/PI staining assay was carried out in CACT knockdown or overexpression OC cells. (I) Lipid peroxidation was evaluated by detecting MDA level in CACT knockdown or overexpression OC cells. For in vitro assays (panels E–I), three independent biological replicates were performed, each consisting of three technical replicates. Non‐targeting scrambled shRNA (shCtrl) or empty plasmid (EV) was used as controls. Data are presented as mean ± SD. Statistical analysis was performed using Student's *t*‐test or one‐way ANOVA, followed by Tukey's post hoc test or Bonferroni post hoc test. ^*^, *p* < 0.05.

As a critical FAO regulator, the role of CACT in OC remains unexplored. Quantitative PCR and IHC staining assays indicated significantly elevated CACT protein expression in clinical tumor tissue samples from OC patients (Figure 7A,B). Similar to OC, significant upregulation of CACT was also observed in a list of other cancer types, as revealed by bioinformatics analysis using the online Sangerbox database (Figure S6D). In agreement with the in vitro findings, a significant positive relationship between the expression levels of CACT and SLC25A46 in clinical OC samples was observed (Figure 7C). Similar to SLC25A46, immunofluorescence staining (IF) analysis showed a significant co‐localization of CACT with VDAC in ES2 and SKOV3 cells (Figure S6E). Furthermore, a negative relationship was observed between the expression of CACT and MARCHF5 in OC (Figure S6F). Kaplan–Meier survival analysis further revealed that upregulation of CACT was associated with poorer patient survival in OC (Figure 7D). We also investigated the biological functions of CACT in OC. The data revealed that CACT knockdown remarkably suppressed proliferation and colony formation, and induced ferroptosis. On the contrary, CACT overexpression promoted proliferation and colony formation, and suppressed ferroptosis (Figure 7E–I and Figure S7A,B). These data indicate that CACT has a comparable function to SLC25A46 in facilitating the advancement of OC.

### 2.8 SLC25A46 Silencing Markedly Increased the Susceptibility of OC Cells to both Ferroptosis Induction and Carboplatin Treatment

**FIGURE 8:**
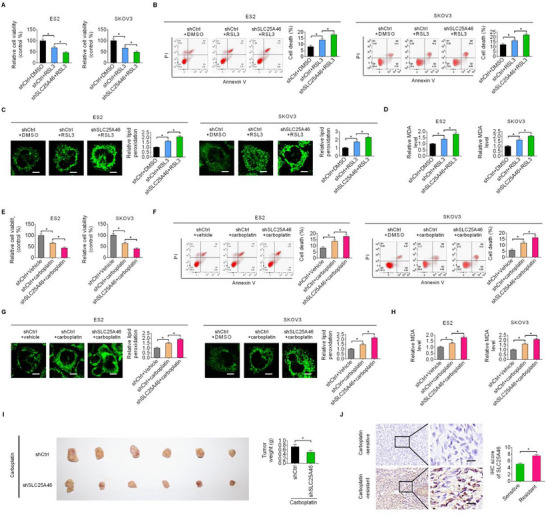
SLC25A46 silencing markedly increased the susceptibility of OC cells to both ferroptosis induction and carboplatin treatment. (A, B) Cell viability (panel A) and cell death (panel B) were analyzed by CCK‐8 assay and flow cytometry in SLC25A46 silenced OC cells treated with the commonly used ferroptosis inducer RSL3. (C, D) lipid peroxidation (panel C, Scale bar = 5 µm) and MDA (panel D) were evaluated in SLC25A46 silenced OC cells treated with RSL3. (E, F) Cell proliferation and cell death were analyzed with CCK‐8 (panel E) and flow cytometry (panel F) in SLC25A46 silenced OC cells treated with carboplatin (20 µm for 24 h). (G, H) Lipid peroxidation (panel G, Scale bar = 5 µm) and MDA (panel H) were evaluated in SLC25A46 silenced OC cells treated with carboplatin (20 µm for 24 h). (I) The effects of SLC25A46 knockdown on the sensitivity of OC cells to carboplatin treatment (50 mg/kg, every three days for 3 weeks) in vivo were evaluated using nude mouse xenograft models. (J) SLC25A46 expression was evaluated by IHC assay in carboplatin‐resistant and ‐sensitive OC patients (*n* = 30, Scale bar = 20 µm). For in vitro assays (panels A–H), three independent biological replicates were performed, each consisting of three technical replicates. Non‐targeting scrambled shRNA (shCtrl) and DMSO were used as controls. Data are presented as mean ± SD. Statistical analysis was performed using two‐way ANOVA or Student's *t*‐test. ^*^, *p* < 0.05.

Mounting evidence has suggested the contribution of ferroptosis to the progression and chemotherapy resistance in ovarian cancers [5]. In this context, we investigated whether the suppression of SLC25A46 could overcome ferroptosis evasion and carboplatin (the most effective chemotherapy drug against epithelial OC) resistance. The results indicated that the effect of RAS‐selective lethal 3 (RSL3, a commonly used ferroptosis inducer) on the induction of ferroptosis was markedly enhanced by SLC25A46 silencing (Figure 8A–D and Figure S7C). Analysis of carboplatin sensitivity showed that SLC25A46 knockdown also markedly enhanced the suppression of cell viability and promotion of cell death caused by carboplatin treatment (Figure 8E,F and Figure S7D,E). In agreement with this, SLC25A46 silencing also significantly enhanced the induction of ferroptosis caused by carboplatin treatment (Figure 8G,H and Figure S7F). Consistent with the in vitro findings, SLC25A46 knockdown also significantly enhanced the therapeutic efficacy of carboplatin against OC growth in nude mouse xenograft models in vivo (Figure 8I). Furthermore, a remarkable upregulation of SLC25A46 was observed in carboplatin‐resistant OC patients compared with carboplatin‐sensitive OC patients (Figure 8J). In accordance with this, established carboplatin‐resistant OC cells also had significantly higher SLC25A46 expression than their sensitive counterparts (Figure S7G,H). These findings demonstrate that SLC25A46 silencing markedly increased the susceptibility of OC cells to ferroptosis induction as well as to carboplatin treatment, suggesting that SLC25A46 may serve as a promising therapeutic target to mitigate carboplatin resistance in OC patients.

### 2.9 PBX1 Functions as a Transcription Factor That Likely Contributes to the Upregulation of SLC25A46 in OC Cells

**FIGURE 9:**
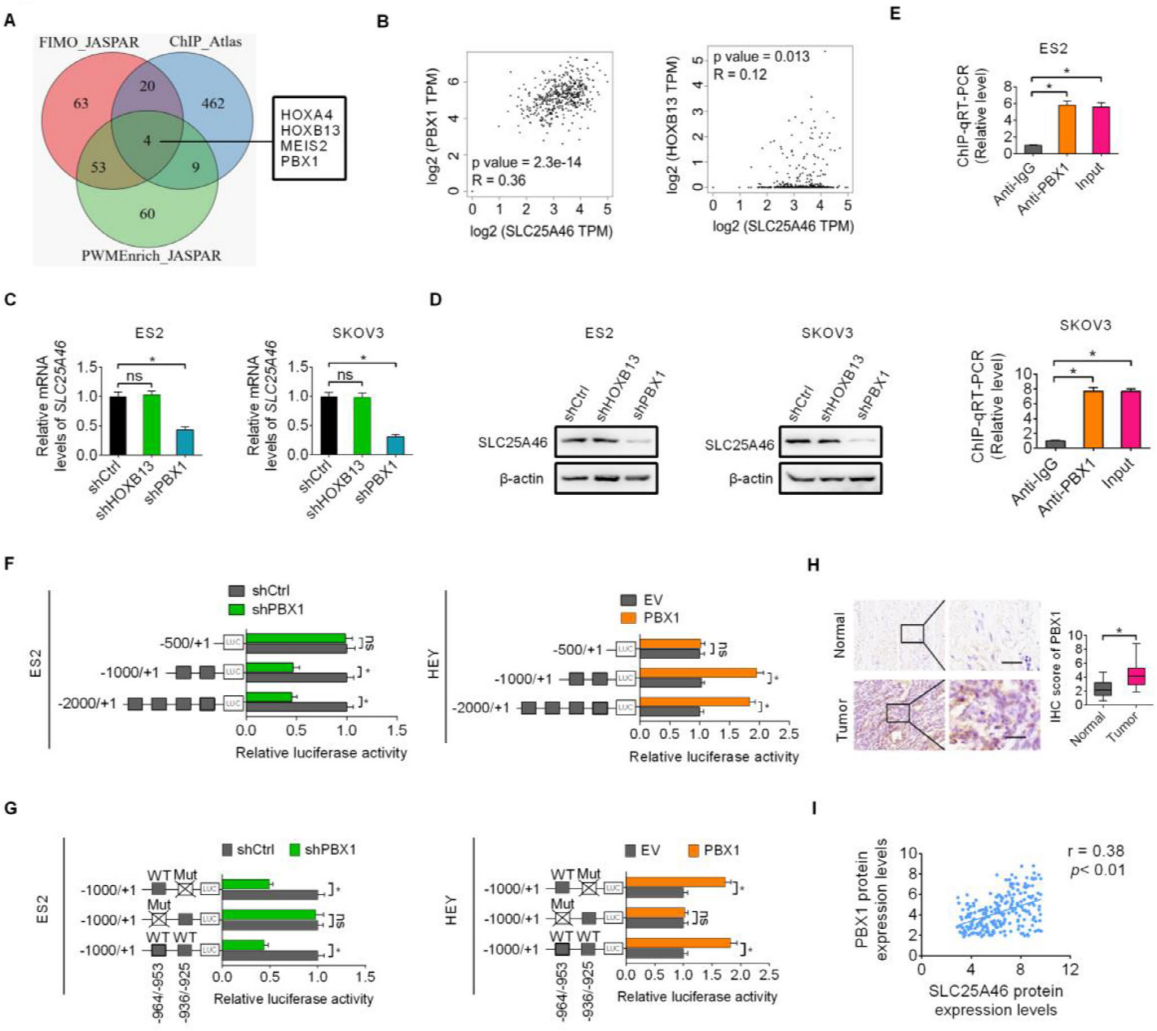
PBX1 functions as a transcription factor that likely contributes to the upregulation of SLC25A46 in OC cells. (A) Four possible transcription factors of SLC25A46 were predicted using the FIMO_JASPAR, ChIP_Atlas, and PWMEnrich_JASPAR databases. (B) Correlations between the expressions of SLC25A46 and indicated transcription factors were assessed in GEPIA database. (C, D) The influence of knockdown of PBX1 or HOXB13 on SLC25A46 expression was determined at mRNA level by qRT‐PCR (panel C) and at protein level by Western blotting (panel D) assays. (E) ChIP‐qPCR assay was carried out using an anti‐PBX1 antibody. (F) The transcriptional activity of PBX1 in truncated SLC25A46 promoter constructs was analyzed by a luciferase reporter assay. (G) The DNA‐binding site in the promoter of SLC25A46 was evaluated using a site‐directed mutagenesis assay. (H, I) PBX1 levels in postoperative tumor tissues and paired normal tissues were evaluated by IHC assay (H panel, comparison of PBX1 expression in tumor and normal tissues, Scale bar = 20 µm; I panel, correlation between the expression levels of PBX1 and SLC25A46 was analyzed using Spearman's rank correlation test). For in vitro assays (panels C–G), three independent biological replicates were performed, each consisting of three technical replicates. Non‐targeting scrambled shRNA (shCtrl) or empty plasmid (EV) was used as controls. Data are presented as mean ± SD. Statistical significance was determined by Student's *t*‐test or one‐way ANOVA. ^*^, *p* < 0.05; ns, not significant.

Finally, we investigated the potential upstream factor responsible for increased SLC25A46 expression in OC cells. Considering that SLC25A46 is upregulated at both the mRNA and protein levels, we screened potential transcription factors contributing to this upregulation utilizing the FIMO‐JASPAR, ChIP‐Atlas, and PWMEnrich‐JASPAR databases. Among the four intersecting transcription factors (Figure 9A), significant positive correlations were observed solely between the expression levels of SLC25A46 and Homeobox Protein Hox‐B13 (HOXB13) and PBX Homeobox 1 (PBX1), as revealed by bioinformatics analysis using the Gene Expression Profiling Interactive Analysis (GEPIA) database (Figure 9B and Figure S8). Knockdown of HOXB13 and PBX1 revealed that SLC25A46 expression was significantly decreased upon PBX1 knockdown, while it remained unchanged upon HOXB13 knockdown (Figure 9C,D). These results suggest PBX1 as a potential transcription factor regulating SLC25A46 in OC cells. To validate this, a ChIP‐qPCR assay was carried out using an anti‐PBX1 antibody, and the results showed significant enrichment of PBX1 at the promoter of SLC25A46 (Figure 9E). To delineate the binding sites, stepwise deletions of SLC25A46 promoter were generated by PCR amplification and inserted into reporter constructs. The transcriptional activity of PBX1 was significantly reduced in the construct truncated from −500 to +1 (Figure 9F). Additionally, the DNA‐binding site at nucleotides −964 and −953 of the SLC25A46 promoter was further uncovered using a site‐directed mutagenesis assay according to the prediction from the JASPAR database (Figure 9G). Furthermore, IHC staining assay revealed that PBX1 expression is elevated in OC and positively correlates with SLC25A46 expression in OC (Figure 9H,I). These data indicate that PBX1 acts as a transcription factor contributing to the upregulation of SLC25A46 in OC cells.

## 3 Discussion

Mitochondria are essential in the management of energy production, biosynthesis, and cell death [17]. Their dysfunction is closely associated with human cancers and is therefore considered a promising therapeutic target in cancer management [18]. SLC25A46 has been reported as a mitochondrial intermembrane bridging protein. However, its expression and functional role in the advancement of human cancer remains unexplored. Here, we found that SLC25A46 was markedly upregulated in OC and associated with poor patient outcomes. Although a significant association exists between high SLC25A46 expression and poor patient survival, the survival curves are not compelling, and further independent cohort validation is required to confirm the prognostic significance of SLC25A46 expression in OC. The current research elucidates the role and mechanisms of SLC25A46 in the promotion of OC progression. We found that upregulation of SLC25A46 plays a crucial role in promoting OC growth. Mechanistic studies demonstrated that SLC25A46 promotes proliferation and evasion of ferroptosis in OC cells through activating fatty acid oxidation‐mediated ATP and NADPH production via protecting MARCHF5‐mediated degradation of carnitine‐acylcarnitine translocase (CACT). Additionally, silencing SLC25A46 markedly increased the susceptibility of OC cells to ferroptosis and their cytotoxic response to carboplatin treatment.

Although it serves as a member of the solute carrier family 25, which functions as mitochondrial transporters involved in the import of substrates into mitochondria across the mitochondrial membrane [19], no known substrate has been associated with SLC25A46. The association between the upregulation of SLC25A46 and worse patient survival suggests SLC25A46 as a putative oncogenic gene in OC. Our investigation of the role of SLC25A46 in OC by loss‐of‐function and gain‐of function studies revealed that SLC25A46 promotes the proliferation of OC cells while inhibiting their death. However, SLC25A46 had no notable impact on migratory and invasive capacities of OC cells, suggesting that the metabolic reprogramming driven by SLC25A46 is more pivotal for coping with energy demands for growth and ferroptosis defense than for motility. Moreover, by employing inhibitors that target various types of programmed cell death (PCD), we further demonstrated that cell death triggered by SLC25A46 silencing was significantly mitigated by suppression of ferroptosis. This observation implies that SLC25A46 may suppress ferroptosis in OC cells, which was further validated by increased lipid peroxidation and MDA levels, as well as significant ferroptosis‐associated alterations in mitochondrial morphology. Mounting evidence has suggested the contribution of ferroptosis to the progression and chemotherapy resistance in human cancers [5, 7, 8]. We found that the induction of ferroptosis by RSL3 was significantly enhanced by SLC25A46 silencing. Consistent with this, knockdown of SLC25A46 also significantly enhanced the inhibition of cell viability and promotion of cell death caused by carboplatin therapy. Our findings suggest that SLC25A46 could serve as a biomarker for predicting carboplatin response in OC patients. Clinically, stratifying OC patients based on SLC25A46 expression levels could facilitate personalized treatment, wherein patients with high SLC25A46 expression, who are more prone to developing carboplatin resistance, could benefit from combined therapy targeting SLC25A46 and standard carboplatin‐based chemotherapy. In addition, targeting SLC25A46 could reverse carboplatin‐based chemotherapy resistance in OC. Similar to our findings, another mitochondrial protein NADJC15 has also been implicated in metabolic reprogramming and cisplatin resistance in OC cells [20]. These findings together support the concept that mitochondrial dysfunction plays crucial roles in chemoresistance in OC, and targeting mitochondrial proteins in combination with conventional chemotherapeutics could enhance treatment efficacy and overcome chemoresistance in ovarian cancer.

Previous studies indicated that SLC25A46 functions as a regulator of mitochondrial dynamics by serving as an intermembrane bridging protein [10], implying that upregulation of SLC25A46 in OC may have an impact on mitochondrial metabolism. Nevertheless, the involvement of SLC25A46 in metabolic processes and the advancement of human cancer remain unexplored. Our data indicate that SLC25A46 markedly enhanced mitochondrial OCR and energy production, while reduced ROS production. Glucose, fatty acids, and glutamine serve as the primary substrates utilized in mitochondrial metabolic processes. In addition, we revealed that the activation of mitochondrial metabolism by SLC25A46 is primarily achieved by activating FAO and thereby increased NADPH production. These data imply that SLC25A46 facilitates the proliferation and ferroptosis evasion of OC cells by activating FAO‐mediated ATP and NADPH production. In line with our observations that SLC25A46‐modulated activation of FAO plays a crucial role in promoting OC progression, enhanced FAO activity documented to play a significant role in cell proliferation, maintenance of cell stemness, and development of drug resistance in several other cancer types. These findings collectively reinforce the notion that FAO activation not only fuels tumor cell proliferation by providing energy products but also confers resistance to ferroptosis by preserving intracellular redox homeostasis.

We further investigated the molecular mechanisms by which SLC25A46 promotes FAO in OC cells. Given that SLC25A46 was reported as an intermembrane bridging protein in the mitochondrial outer membrane [10], we analyzed SLC25A46‐interacting proteins and identified that CACT (a mitochondrial membrane protein governing the rate of FAO) directly interacted with SLC25A46. In addition, we found that SLC25A46 upregulated CACT expression by protecting CACT from proteasome‐mediated degradation in OC cells. MARCHF5 has been reported as a critical mitochondrial outer membrane‐localized E3 ubiquitin‐protein ligase participating in OC progression [21, 22]. Our data revealed that both SLC25A46 and CACT directly interacted with MARCHF5, and that SLC25A46 silencing clearly diminished the interaction between MARCHF5 and CACT. Meanwhile, the downregulation of CACT by SLC25A46 silencing was significantly attenuated when MARCHF5 was knocked‐down. These findings provide strong evidence that SLC25A46 directly binds to CACT and increases its stability by competitively suppressing MARCHF5‐mediated CACT ubiquitination and degradation. Although the contribution of FAO to cancer progression has been well recognized, the role of CACT in OC remains unexplored. Similar to SLC25A46, we also observed a significant upregulation of CACT and an oncogenic role in OC. Additionally, a positive relationship was observed between the expressions of SLC25A46 and CACT in OC. These findings provide further support not only for the critical contribution of FAO to OC progression, but also for the critical role of SLC25A46 in the regulation of CACT expression.

PBX1 is a transcription factor that has been reported to promote multiple oncogenic signaling pathways and malignant processes in human cancer, including cell proliferation, epithelial‐mesenchymal transition (EMT), apoptosis resistance, migration, and metastasis [23]. In OC, PBX1 was indicated as a promotor of cancer stem cell‐like phenotypes and resistance of platinum treatment [24]. Herein, we demonstrate that PBX1 directly binds to the promoter of SLC25A46 at nucleotides −964 and −953 and transcriptionally upregulated SLC25A46 in OC cells. These findings collectively suggest that increased expression of PBX1 may contribute to the upregulation of SLC25A46 in OC cells.

## 4 Conclusion

Altogether, this study uncovers SLC25A46 as a critical oncogene in the advancement of OC by facilitating cell proliferation and evasion of ferroptosis via activating FAO‐mediated ATP and NADPH production through protecting carnitine‐acylcarnitine translocase (CACT) from MARCHF5‐mediated degradation. More importantly, SLC25A46 silencing markedly heightened the susceptibility of OC cells to ferroptosis induction and enhanced their cytotoxic response to carboplatin, holding promise for SLC25A46 as a potential target to improve treatment outcomes in OC patients.

### 4.1 Limitations of the Study

(1) Although selecting cell models based on total cellular expression in this study is reasonable for comprehensively exploring SLC25A46 functions beyond mitochondria, given that SLC25A46 is a mitochondrial protein, mitochondrial abundance is a more relevant criterion for selecting cell lines to study its functions. (2) The knockdown efficiency of SLC25A46 mediated by siRNA was modest. To achieve higher knockdown efficiency, alternative gene silencing approaches such as CRISPR‐Cas9 should be used in future studies.

## 5 Experimental Section

### 5.1 Chemicals

Carboplatin (catalog number #S1215), UK5099 (catalog number #S5317), Etomoxir sodium salt (catalog number #S8244), BPTES (catalog number #S7753), MG132 (catalog number #S2619), Chloroquine (catalog number #S6999), Sulfosuccinimidyl Oleate Sodium (SSO, catalog number E2988), and TOFA (catalog number #S6690) were purchased from Selleck Chemicals.

### 5.2 Clinical Specimens

Tissue samples were collected from a total of 244 individuals diagnosed with OC at the First Affiliated Hospital (Also known as Xijing Hospital) of the Air Force Medical University between 2019 and 2024. Informed consent was obtained from all participants. Among the 244 tissue samples, 30 paired fresh‐frozen tissues (comprising 27 high‐grade serous, 2 endometrioid, and 1 mucinous ovarian cancer) collected during surgery were used for qRT‐PCR analysis, and the rest 214 FFPE samples (including 189 high‐grade serous, 16 mucinous, 6 clear‐cell, and 3 endometrioid ovarian cancer) were used for immunohistochemistry analysis. RNA integrity was assessed by agarose gel electrophoresis. All patients were treatment‐naïve at the time of sample collection, and all samples originated from primary ovarian cancer. Tumors arising from the fallopian tube, primary peritoneal lesions, and secondary metastatic tumors were not included in our cohort. H&E staining‐based histopathological examination by two independent pathologists confirmed the diagnosis and tumor purity. Paired tumor adjacent normal tissues, primarily consisted of ovarian stromal tissue with surface epithelium, without microscopic lesions and tumor infiltration, were macroscopically and histologically confirmed as normal and dissected by a pathologist. The study was approved by our Hospital and performed under Helsinki‐compliant protocols.

### 5.3 Cell Lines Culture

Five ovarian cancer (OC) cell lines, including A2780, ES2, HEY, OVCAR3, and SKOV3, and a normal ovarian epithelial cell line IOSE‐80 were used in our investigation. These cell lines ensure the coverage of major OC histologically distinct subtypes, including OVCAR3, representing the most prevalent high‐grade serous subtype, ES2 and HEY, representing the low‐grade serous subtype, SKOV3, representing the clear cell subtype, and A2780, representing the endometrioid subtype [25, 26]. These cell lines were maintained in Dulbecco's Modified Eagle Medium (DMEM)‐10% (v/v) FBS. The genetic backgrounds of all cell lines were authenticated by STR (Short Tandem Repeat) profiling and cross‐referenced with publicly available data from CCLE and ATCC databases. Key genetic characteristics, including TP53, BRCA1/2, KRAS, and PIK3CA, are summarized in Table S2. The absence of mycoplasma was verified by PCR‐based MycoAlert.

### 5.4 Cell Transfection and Development of Stable Cell Lines

Lentiviral vectors were used for knockdown of SLC25A46 in OC cells (targeting sequences: SLC25A46#1, 5′‐TTGAAACAGTGCAGAGTATGTTG‐3′; SLC25A46#2, 5′‐GGCTATGAAGTGCTTCCAATTAA‐3′). Stable SLC25A46‐deficient OC cell lines were established by transducing shRNA‐pSilencer constructs (Lipofectamine 2000) followed by 30‐day neomycinclonal selection. For gain‐of‐function, SLC25A46‐pcDNA3.1 or empty vector was transfected into OC cells. Stable SLC25A46‐overexpression was obtained by isolating G418 resistant clones after 4‐week expansion.

### 5.5 RNA Isolation and Quantitative RT‐PCR

RNA was TRIzol‐extracted (Invitrogen), reverse‐transcribed (EZBioscience), and quantified by SYBR‐Green qPCR (Takara) on a CFX96 platform. The relative mRNA expression levels of target genes were calculated by the 2^−ΔΔCt^ method, with the β‐actin serving as the endogenous control. All specific primers utilized in the current study are provided in Table S3.

### 5.6 Western Blot Analysis

OC cells were solubilized in ice‐cold RIPA buffer containing protease blockers (Roche). Equal amounts (20 µg) of protein samples were resolved by 10% SDS‐PAGE and electro‐transferred to 0.2‐µm PVDF (Millipore). After that, the membranes were incubated in 5% non‐fat milk for 1.5 h, followed by probing with primary antibodies (4°C, 12 h) and subsequent HRP‐conjugated secondary antibodies (28°C, 2.5 h). Immunoreactive bands were detected by an enhanced ECL reagent kit (Bio‐Rad). All primary antibodies utilized in the current study are provided in Table S4.

### 5.7 Immunohistochemistry (IHC) Staining Assay

Tissues obtained from ovarian cancer (OC) patients or nude mice were embedded, sectioned, and rehydrated. Subsequently, heat‐induced epitope retrieval was performed in citrate buffer (pH 6.0, 100°C, 10 min). After that, endogenous peroxidase was quenched with 3% H_2_O_2_ (30 min) prior to overnight primary antibody (Table S4) incubation (4°C). DAB was added to the sections to develop the staining signal and counterstained with hematoxylin staining solution. The staining result was assessed by two pathologists under an Olympus microscope.

The staining intensity was classified into four categories (score ranging from 0 to 3): 0 for negative, 1 for weak, 2 for moderate, and 3 for strong staining, respectively. The proportion of positive staining was scored as follows (ranging from 1 to 4): 1 for less than 10%, 2 for 11%–50%, 3 for 51%–80%, and 4 for more than 80%. The final IHC score (ranging from 0 to 12) was derived by multiplying the intensity score by the proportion score. SLC25A46‐high and SLC25A46‐low expression groups were divided using the median IHC score as the cutoff.

### 5.8 Cell Viability Assay

OC cells were collected and seeded in 96‐well culture plates (3000 cells/well). After culturing for the indicated hours, CCK‐8 solution (10 µL) (Beyotime Biotech, China) was added (1.5 h, 37°C). Absorbance (450 nm) was read on a microplate reader.

### 5.9 Colony Formation Assay

OC cells were collected and seeded in 6‐well culture plates (1 × 10^3^ cells per well). Colonies were assessed after 15 days, fixed (4% PFA, 15 min), stained with 0.1% crystal violet, and quantified by ImageJ software.

### 5.10 5‐Ethynyl‐2′‐deoxyuridine (EdU) Assay

OC cells were collected and plated into glass‐bottom confocal dishes (Corning, USA). After being cultured for 12 h, cells were stained with EdU (10 µm, 1.5 h) and DAPI (10 min) in the dark. The percentage of EdU‐positive cells was calculated within five randomly selected fields per well as the ratio of EdU‐positive nuclei to DAPI‐stained nuclei and multiplied by 100%.

### 5.11 Wound Healing and Transwell Invasion Assays

The migration of OC cells was analyzed by a wound healing assay. Upon OC cells grown to 90% confluency, a pipette tip was used to create wound lines. Cell migration was assessed under a light microscope 24 h after scratching.

The invasion of OC cells was analyzed using a transwell invasion assay. Briefly, 6000 OC cells were plated in the upper chamber coated with matrigel in serum‐free medium. After 48 h of culture, the invaded cells were fixed with 4% paraformaldehyde and stained with crystal violet (0.1%). Invaded cells were imaged, and cell numbers were counted using ImageJ software.

### 5.12 Detections of Lipid Peroxidation and Fe^2+^

For the detection of lipid peroxidation, OC cells seeded in glass‐bottom confocal dishes (Corning, USA) were incubated with 2 µm BODIPY 581/591C11 dye (Thermo Fisher, 30 min, 37°C). After two PBS rinses, BODIPY 581/591C11 staining was imaged using a laser confocal microscope. Relative lipid peroxidation was indicated by the fluorescence staining intensity using Image J.

For the detection of Fe^2+^, cells were incubated with 1 µm FerroOrange dye (Dojindo, 30 min, 37°C). After two PBS rinses, FerroOrange staining was imaged using a laser confocal microscope. Relative Fe^2+^ levels were indicated by the fluorescence staining intensity using Image J.

### 5.13 Oxygen Species (ROS) Detection

OC cells plated in glass‐bottom confocal dishes (Corning, USA) were incubated with 10 µm DCFDA (Sigma) for 20 min at 37°C. After two PBS rinses, DCFDA staining was imaged under a laser confocal microscope. Relative ROS levels were indicated by the fluorescence staining intensity quantified using Image J.

### 5.14 Measurement of Malondialdehyde (MDA)

The MDA content in OC cells was quantified using a lipid peroxidation MDA kit purchased from Beyotime Biotech in China, according to the manufacturer's protocols. The MDA content was normalized to BCA‐quantified protein concentration.

### 5.15 Cell Death Detection Assay

Cell death was quantified by Annexin V‐FITC/PI (Beyotime) staining assay (20 min, 28°C, dark). After two PBS rinses, the flow cytometer was used for evaluating cells positive for both Annexin V and PI, which were considered as dead.

### 5.16 Mitochondrial Isolation

Ovarian cancer cells were PBS‐washed and homogenized in mitochondrial isolation buffer (Beyotime). The homogenate was then centrifuged for 30 min at 1000 × g at 4°C. Subsequently, the supernatant containing the mitochondrial fraction was transferred to a new tube and centrifuged again for 10 min at 12 000 × g at 4°C. After that, lysis buffer was added to the resulting pellet containing mitochondria, which was then resuspended and centrifuged for 20 min at 12 000 × g at 4°C. The supernatants containing mitochondrial proteins were used for immunoblotting analysis.

### 5.17 Proteinase K Protection Assay

Purified mitochondria were re‐suspended in cold mitochondria isolation solution and treated with proteinase K for 25 min with or without Triton X‐100 (1%). After that, the samples were analyzed by immunoblotting assay as described above. MFN1, COX4, and TFAM served as indicators for the outer or inner mitochondrial membrane (OMM or IMM), or mitochondrial matrix (MM).

### 5.18 Co‐Immunoprecipitation (co‐IP) Assay

OC cells were solubilized in RIPA–protease blocker mix (Roche) and then incubated with specific antibodies or protein G beads to enrich target proteins for 1.5 h at 4°C. After washing the beads using the 0.5% NP‐40 buffer three times, the results were analyzed by immunoblotting assay.

### 5.19 LC‐MS Analysis

Liquid chromatography–tandem mass spectrometry (LC–MS/MS) analysis of the co‐immunoprecipitation (co‐IP) samples was performed by Applied Protein Technology (APTBIO, Shanghai, China). Briefly, eluted proteins were reduced with dithiothreitol (DTT), alkylated with iodoacetamide (IAA), and digested with trypsin overnight at 37°C. Peptides were separated on an Easy‐nLC 1200 system (Thermo Fisher) coupled to a Q Exactive HF‐X Orbitrap mass spectrometer (Thermo Fisher). Data were acquired in data‐dependent acquisition (DDA) mode. Raw data were processed using MaxQuant (version 1.6.14.0) against the human UniProt database. Search parameters included trypsin/P specificity, a maximum of 2 missed cleavages, fixed carbamidomethylation of cysteine, and variable modifications of methionine oxidation and protein N‐terminal acetylation. The false discovery rate (FDR) was set to 1% at both peptide‐spectrum match (PSM) and protein levels.

### 5.20 DNA Extraction and Quantification of mtDNA

Relative mitochondrial DNA (mtDNA) levels were quantified by qRT‐PCR assay using genomic DNA extracted from OC cells. DNA was extracted with a commercial kit (TIANGEN) per the supplier's protocol. The results were expressed as the ratio of ND1 (a mitochondrial gene) to HGB (a nuclear gene) with primer sequences listed in Table S1.

### 5.21 Animal Experiments

Four‐week‐old female BALB/c nude mice (*n* = 6/group) were maintained under SPF conditions. All experimental procedures were approved by the Institutional Animal Care Committee of our Hospital and followed NIH guidelines.

For the xenograft tumor growth assay, 6 × 10^6^ OC cells were subcutaneously injected in the right flank of the animal. The animals were monitored every day, and volumes were measured weekly with a caliper (Tumor volume = (length × width^2^) / 2) by two independent researchers in a blinded manner. After four weeks, the animals were humanely euthanized according to institutional ethical guidelines, and xenograft tumors were harvested for further analysis.

For the lung metastasis assay, 4 × 10^6^ OC cells were delivered via lateral tail‐vein injection. The animals were monitored every day and humanely euthanized 30 days post cells injection. Lungs were excised and evaluated by H&E for metastatic foci.

### 5.22 Statistical Analysis

Data are mean ± SD (Standard Deviation) of 3 biologically independent experiments and analyzed using GraphPad Prism 9. Two‐group comparisons were conducted using two‐tailed unpaired *t* tests. Multiple‐group comparisons were evaluated by one‐way ANOVA with Tukey post‐test. Correlation was assessed using Spearman's rank correlation analysis. ^*^ *p* < 0.05 (statistical significance), ns = not significant.

## Author Contributions

YG, JH, and XZ contributed equally to this work. XL, XG, and SL designed, supervised, and funded the study. YG, JH, and XZ performed most in vitro and in vivo experiments. ML and YF contributed to the in vitro experiments and data analysis. XL and XL contributed to the in vivo experiments and data analysis. YG, JH, and XZ wrote the manuscript. XL, XG, and SL reviewed and edited the paper. All authors approved the final manuscript.

## Ethics

The current study was approved by the Medical Research Ethics Committee of the First Affiliated Hospital of Fourth Military Medical University (No. KY20243516‐1). All animal experiments were approved by the Ethics Committee of the Department of Laboratory Animals of Fourth Military Medical University (No. 20240566). Written informed consent was obtained from all participants in the study.

## Conflicts of Interest

The authors declare no conflicts of interest.

## Supporting information




**Supporting File 1**: advs76689‐sup‐0001‐SuppMat.docx.

**Supporting File 2**: advs76689‐sup‐0002‐original_ blots_ images.pdf.

## Data Availability

The data that support the findings of this study are available from the corresponding author upon reasonable request.
