# Supplementary material for: Mitochondrial SLC25A46 Rewires Fatty Acid Oxidation to Promote Cell Proliferation and Ferroptosis Evasion in Ovarian Cancer by Stabilizing CACT
Source: Adv Sci (Weinh). 2026 Jul 20;13(57):e23969. doi: 10.1002/advs.202523969 (PMC13383691; doi:10.1002/advs.202523969)
Supplement: Supplementary file 1 [file ADVS-13-e23969-s001.pdf]

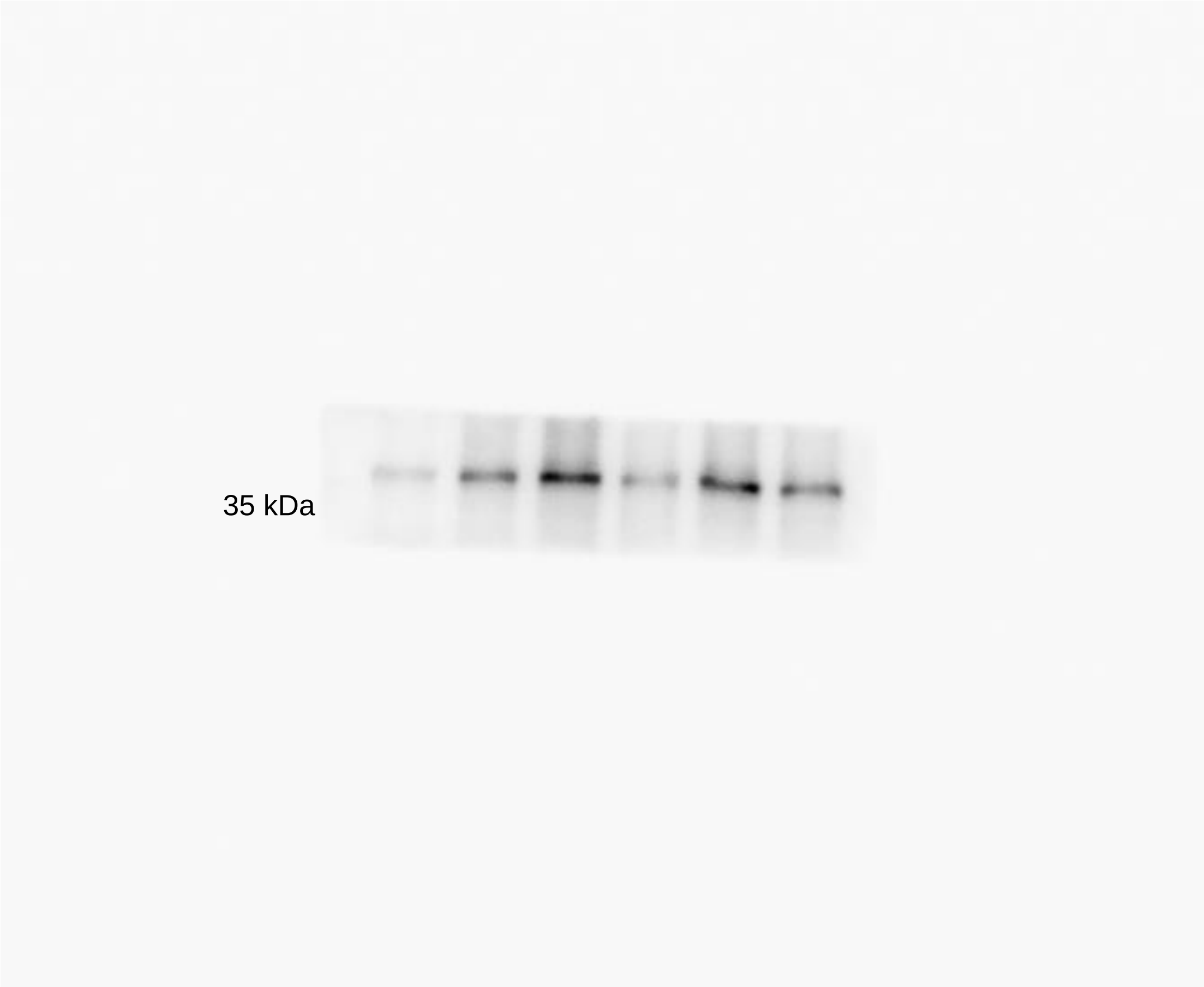


**Fig 1H**

**IB: SLC25A46**

**Groups**: IOSE80, A2780, ES2, HEY, OVCAR3, SKOV3 (whole cell lysate)

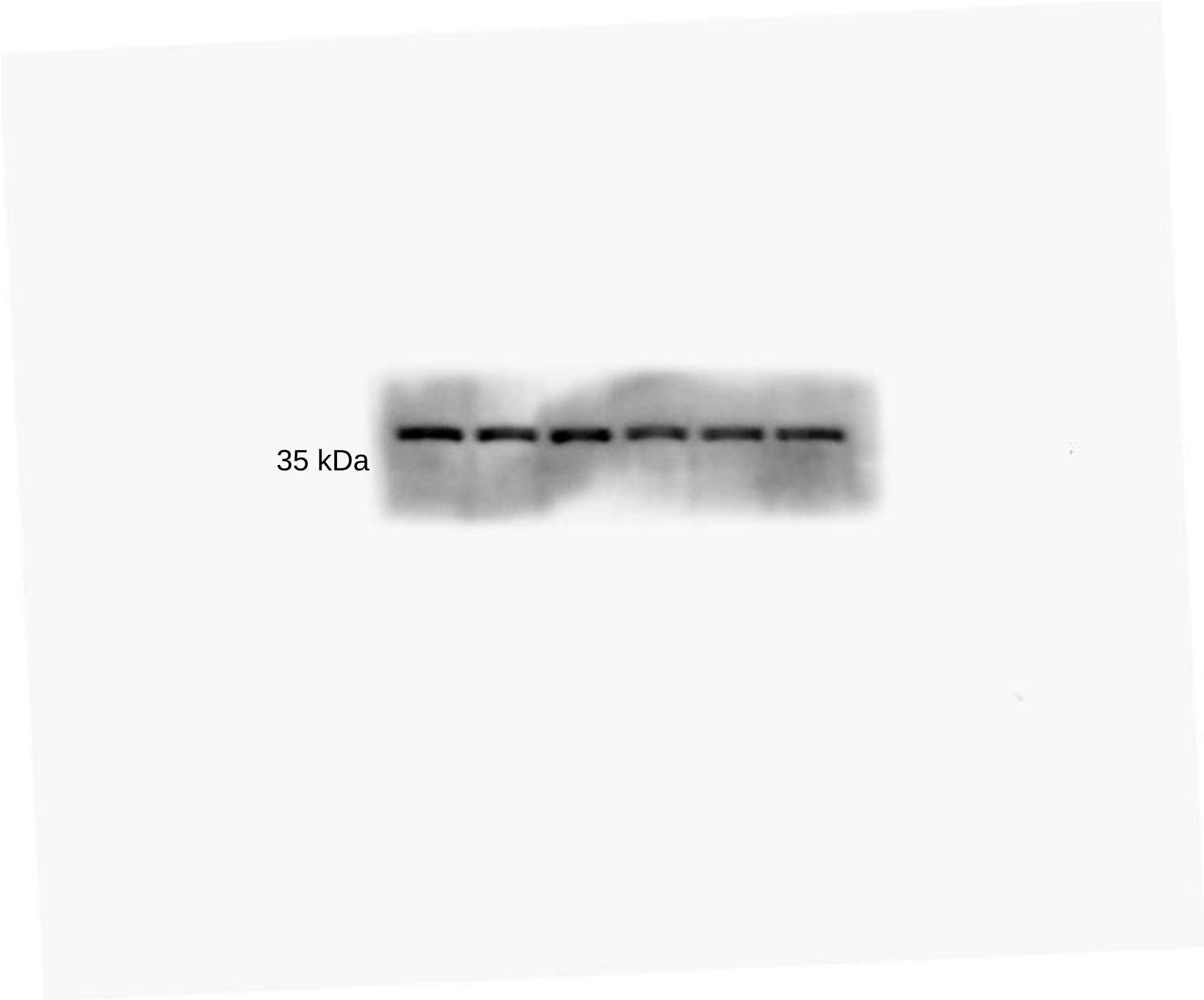


**Fig 1H**

**IB: β-actin**

**Groups**: IOSE80, A2780, ES2, HEY, OVCAR3, SKOV3 (whole cell lysate)

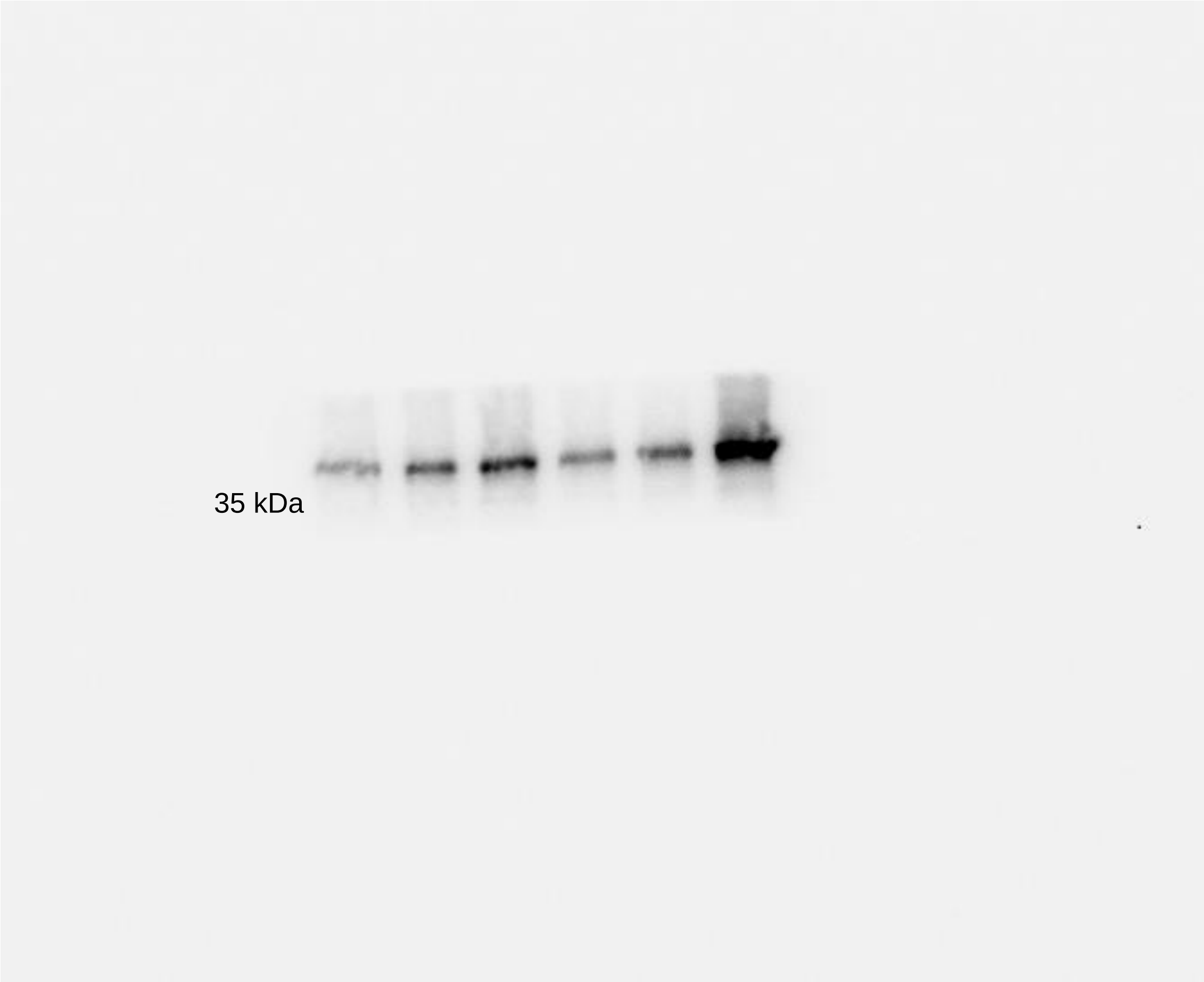


**Fig 1H**

**IB: SLC25A46**

**Groups**: IOSE80, A2780, ES2, HEY, OVCAR3, SKOV3 (mitochondrial fraction)

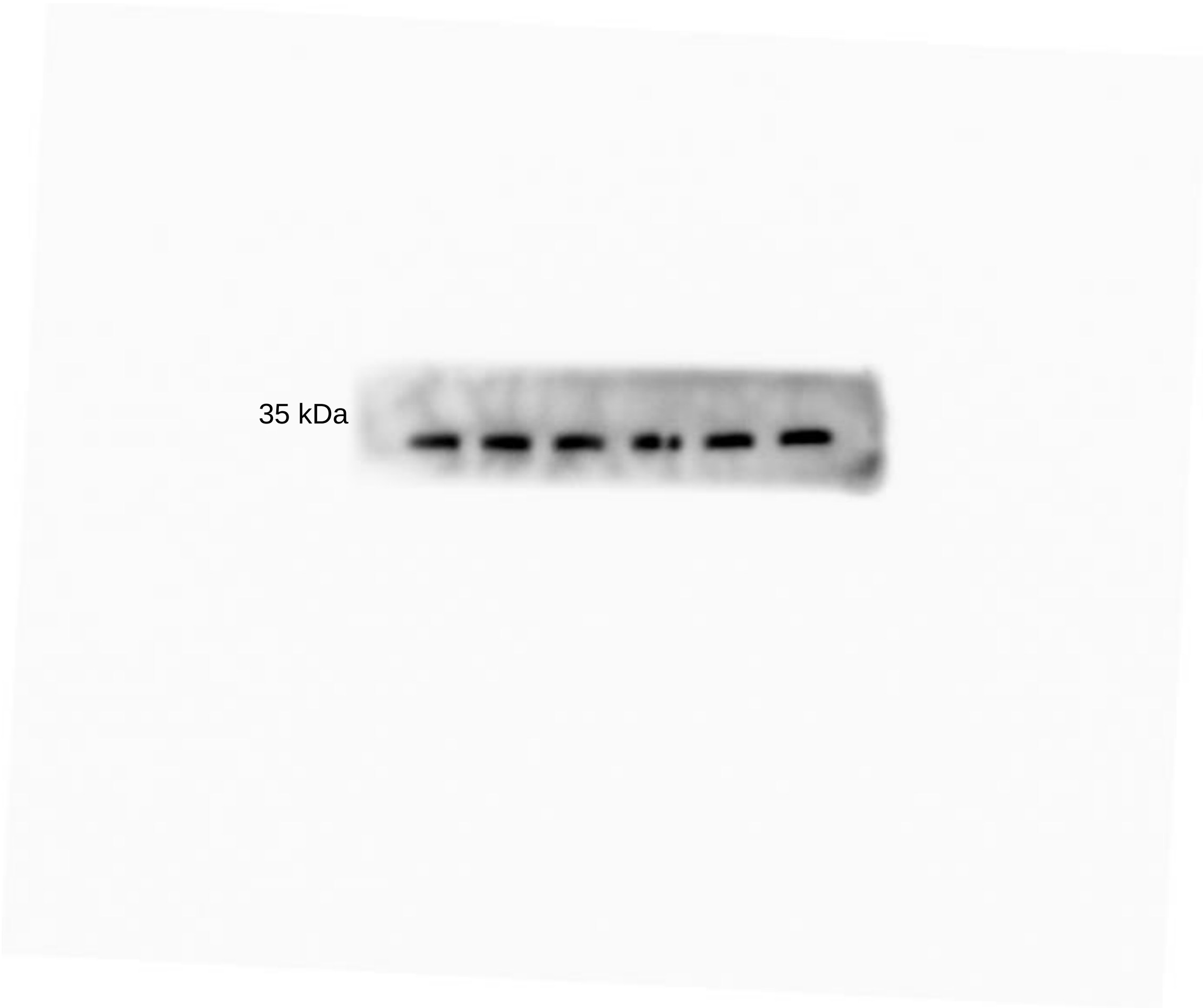


**Fig 1H**

**IB: VDAC**

**Groups**: IOSE80, A2780, ES2, HEY, OVCAR3, SKOV3 (mitochondrial fraction)

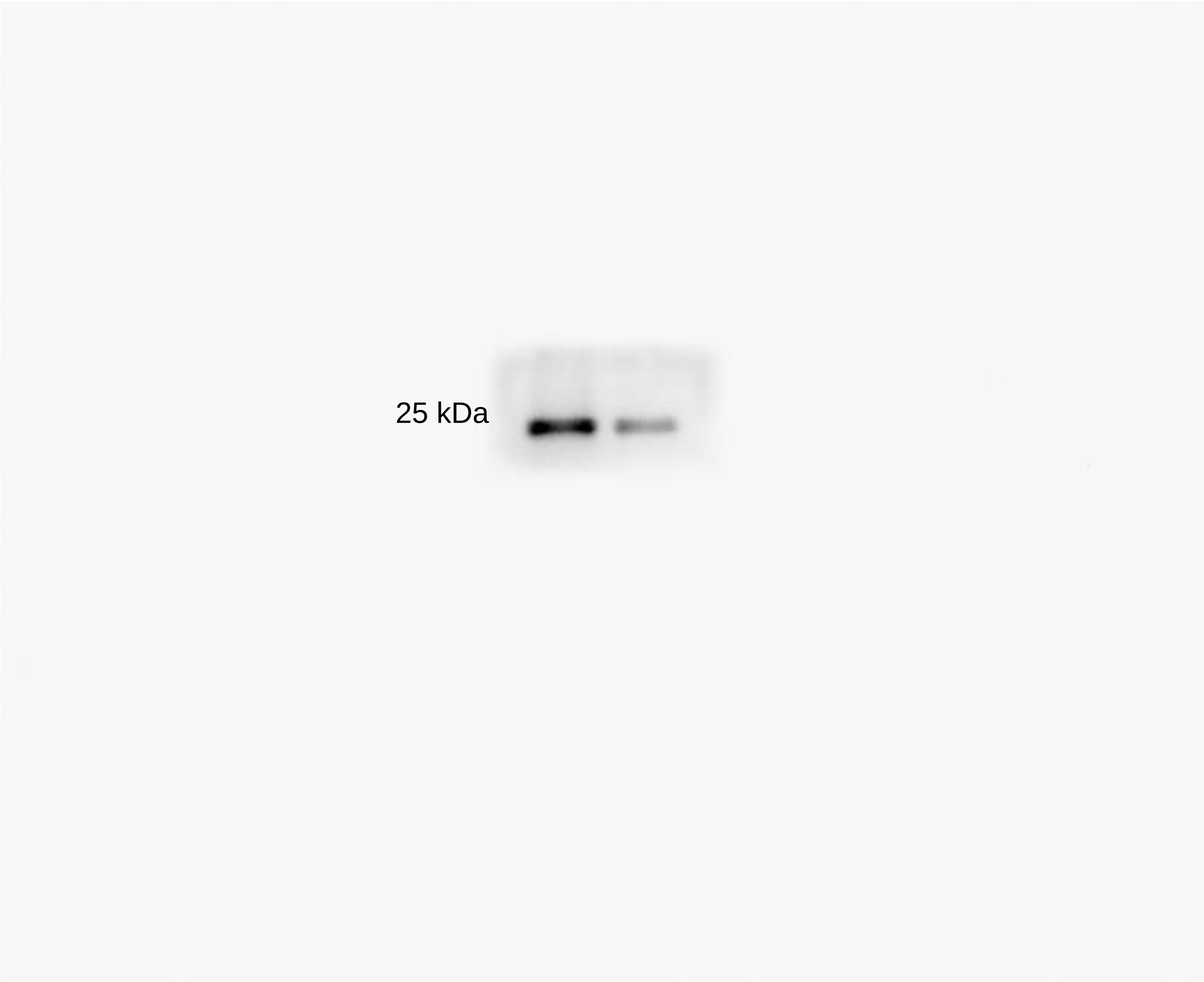


**Fig 3H**

**IB: GPX4**

**Groups (ES2 cells):** shCtrl, shSLC25A46

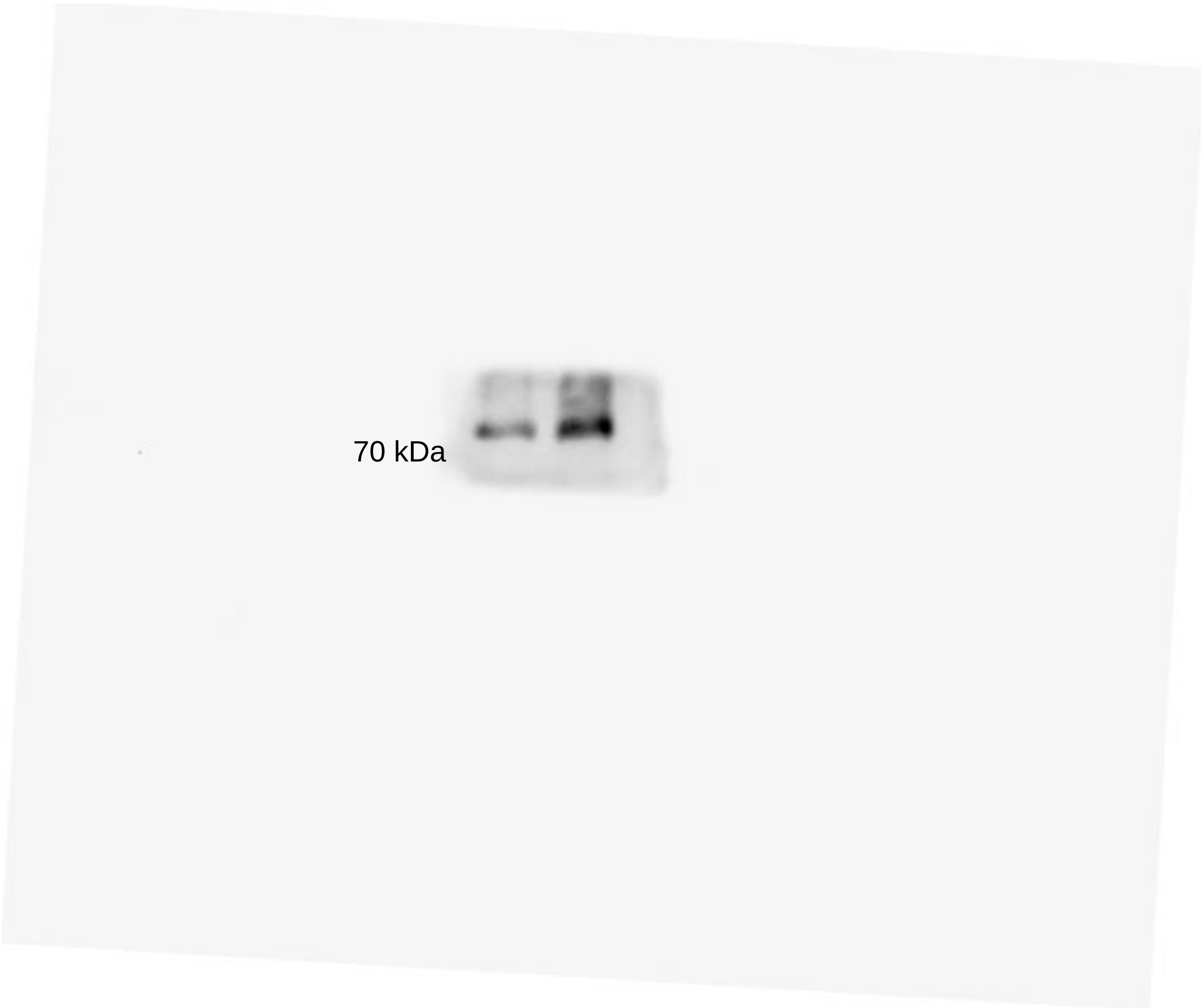


**Fig 3H**

**IB: ACSL4**

**Groups (ES2 cells):** shCtrl, shSLC25A46

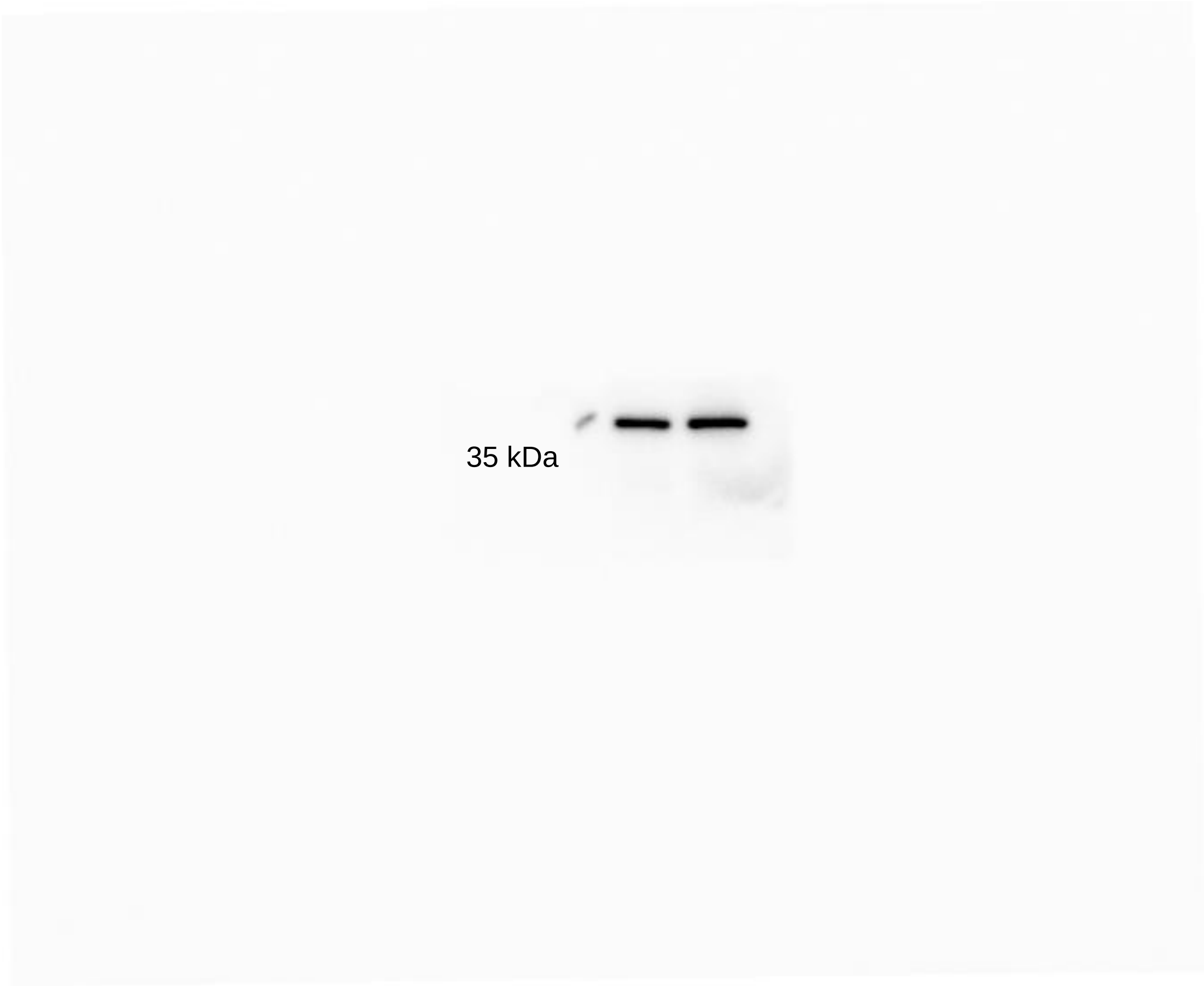


**Fig 3H**

**IB: β-actin**

**Groups (ES2 cells):** shCtrl, shSLC25A46

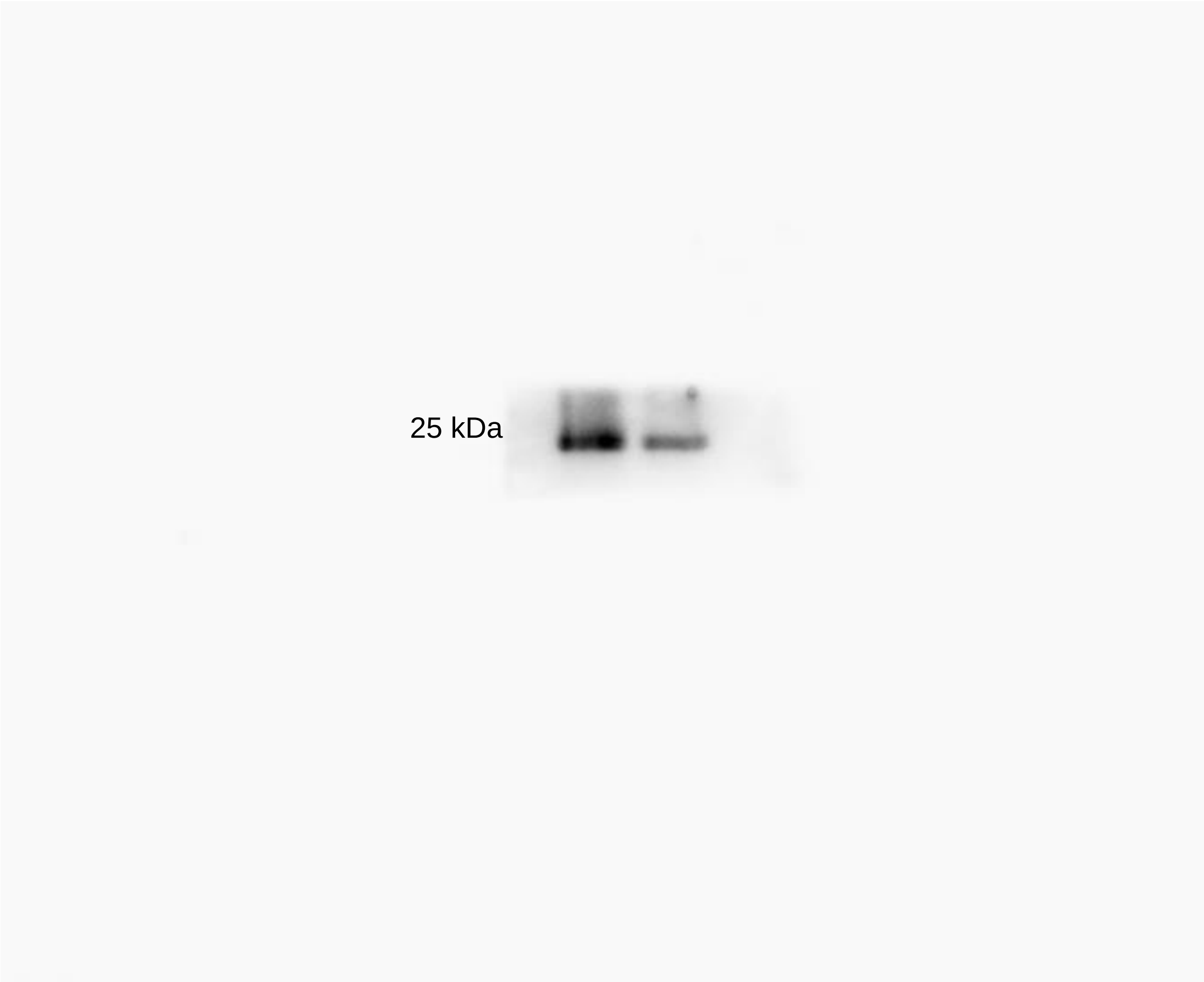


**Fig 3H**

**IB: GPX4**

**Groups (HEY cells):** EV, SLC25A46

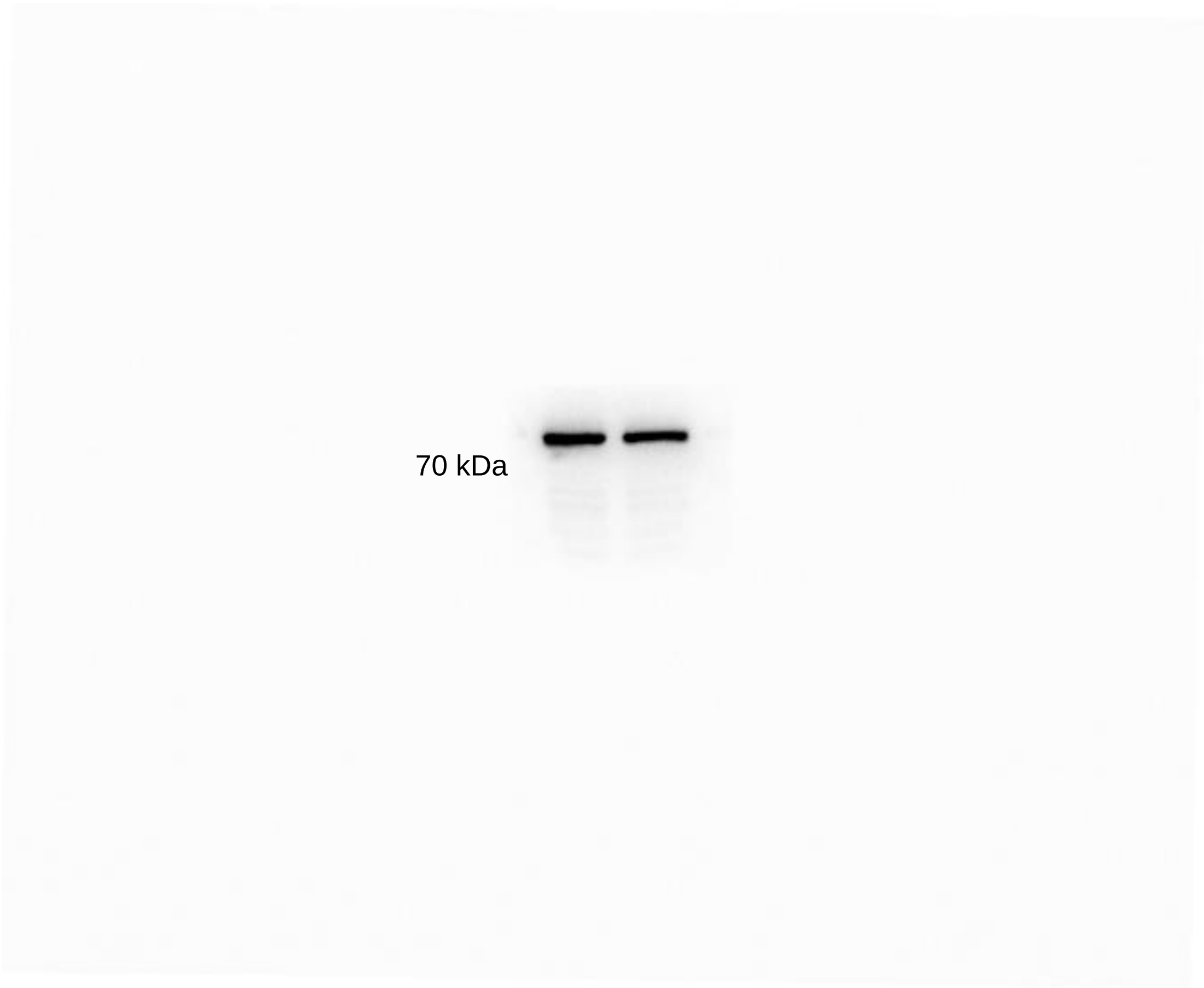


**Fig 3H**

**IB: ACSL4**

**Groups (HEY cells):** EV, SLC25A46

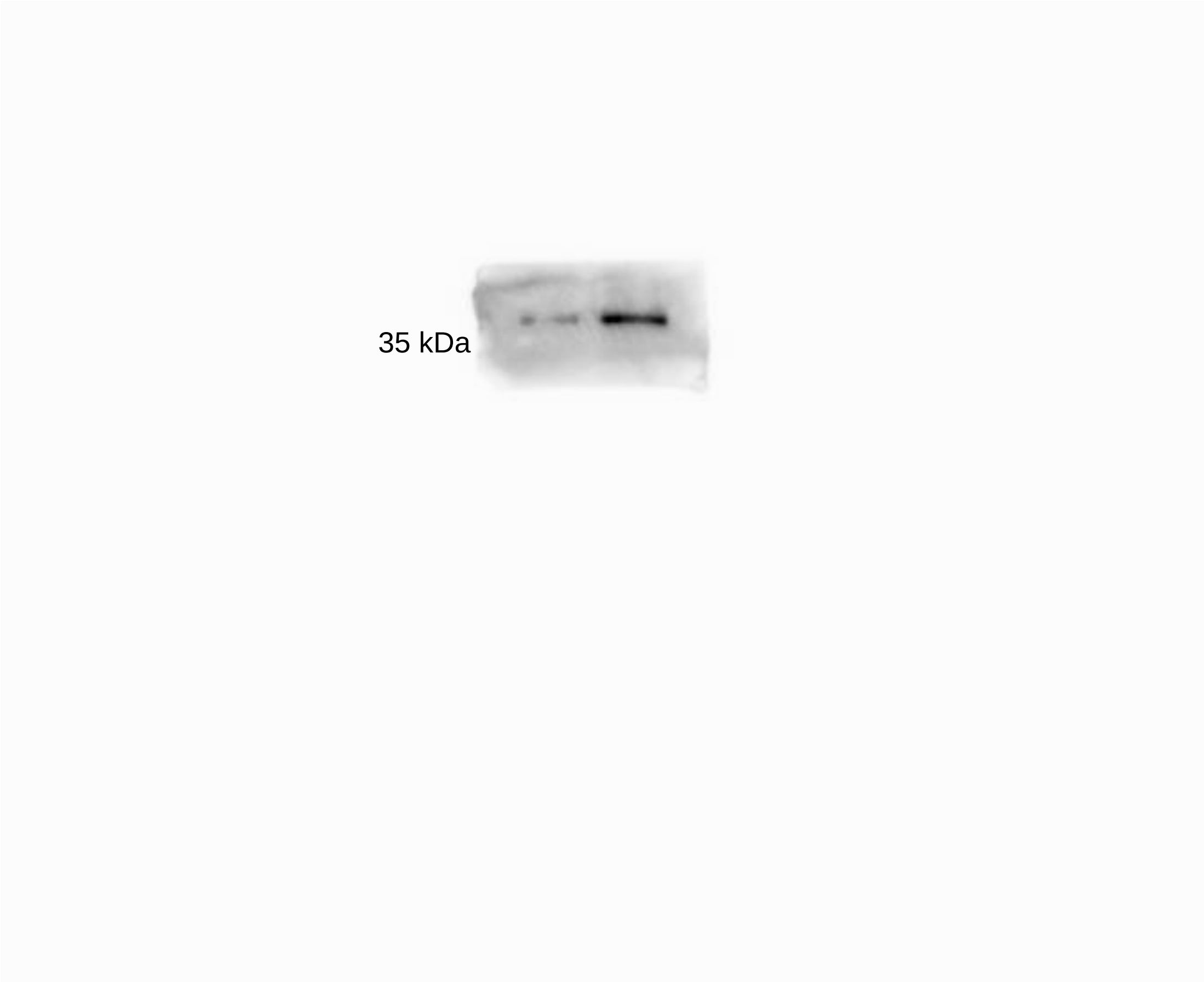


**Fig 3H**

**IB: β-actin**

**Groups (HEY cells):** EV, SLC25SA46

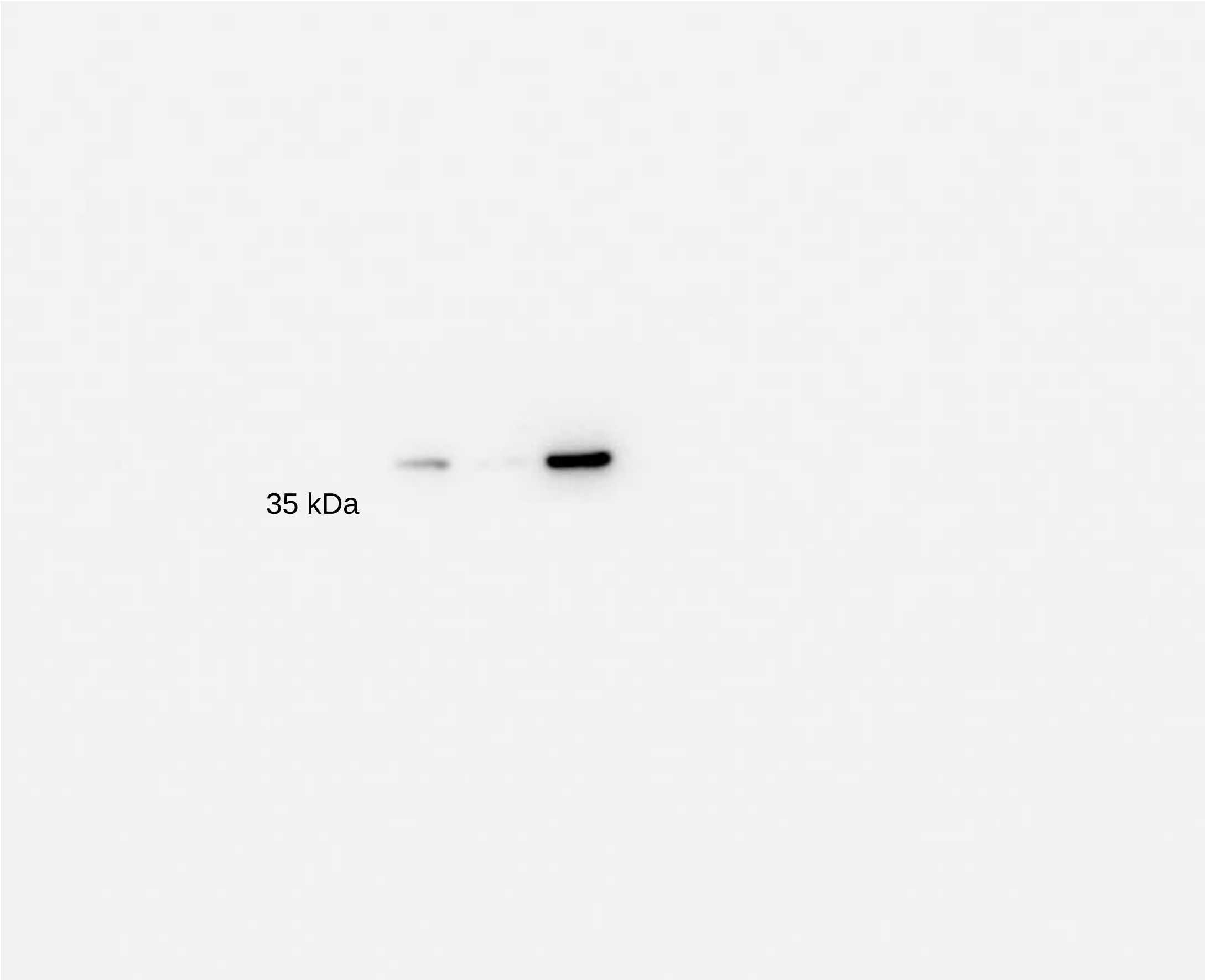


**Fig 5B**

**IB: SLC25A46**

Groups (ES2 cells): Input, IgG (IP), SLC25A46 (IP)

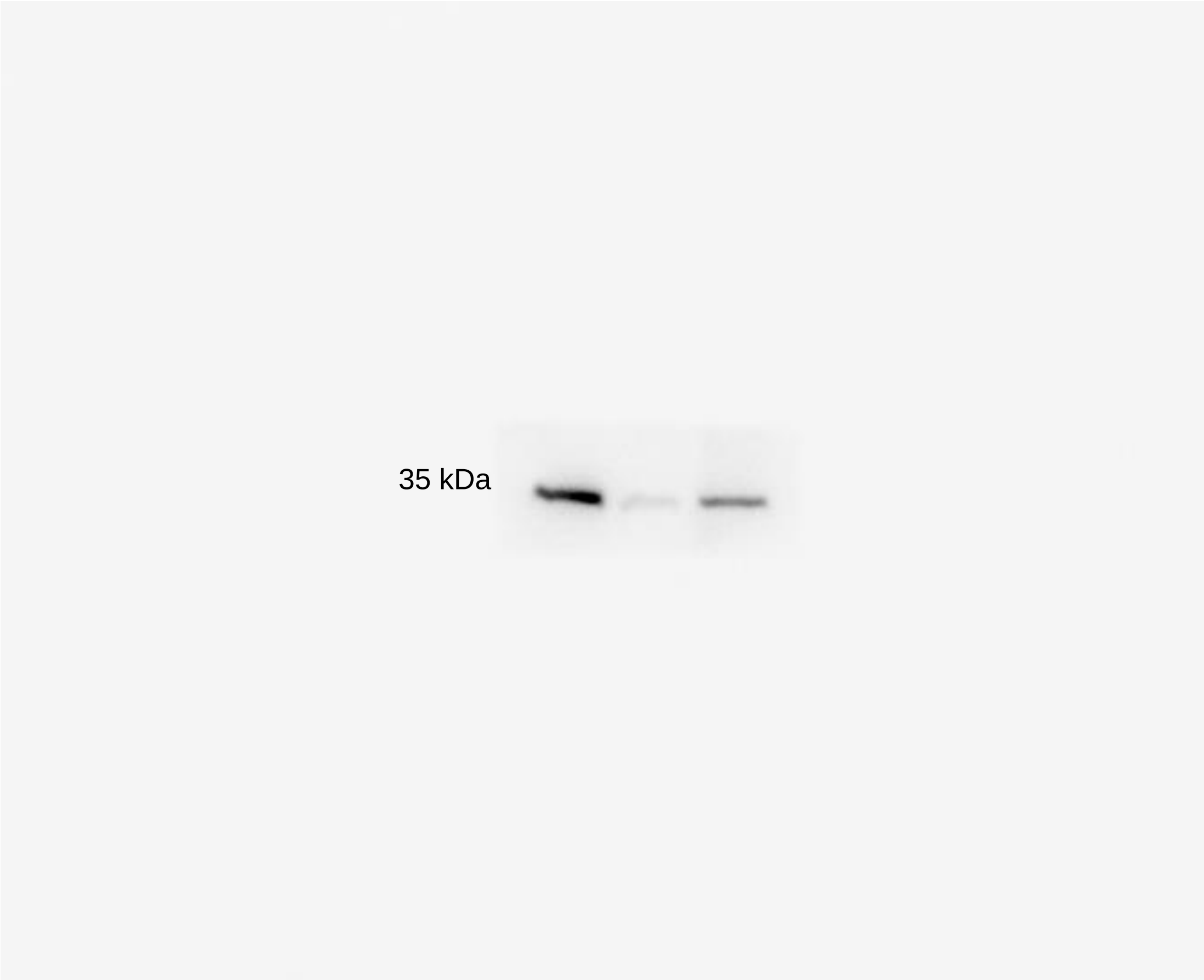


**Fig 5B**

**IB: CACT**

Groups (ES2 cells): Input, IgG (IP), SLC25A46 (IP)

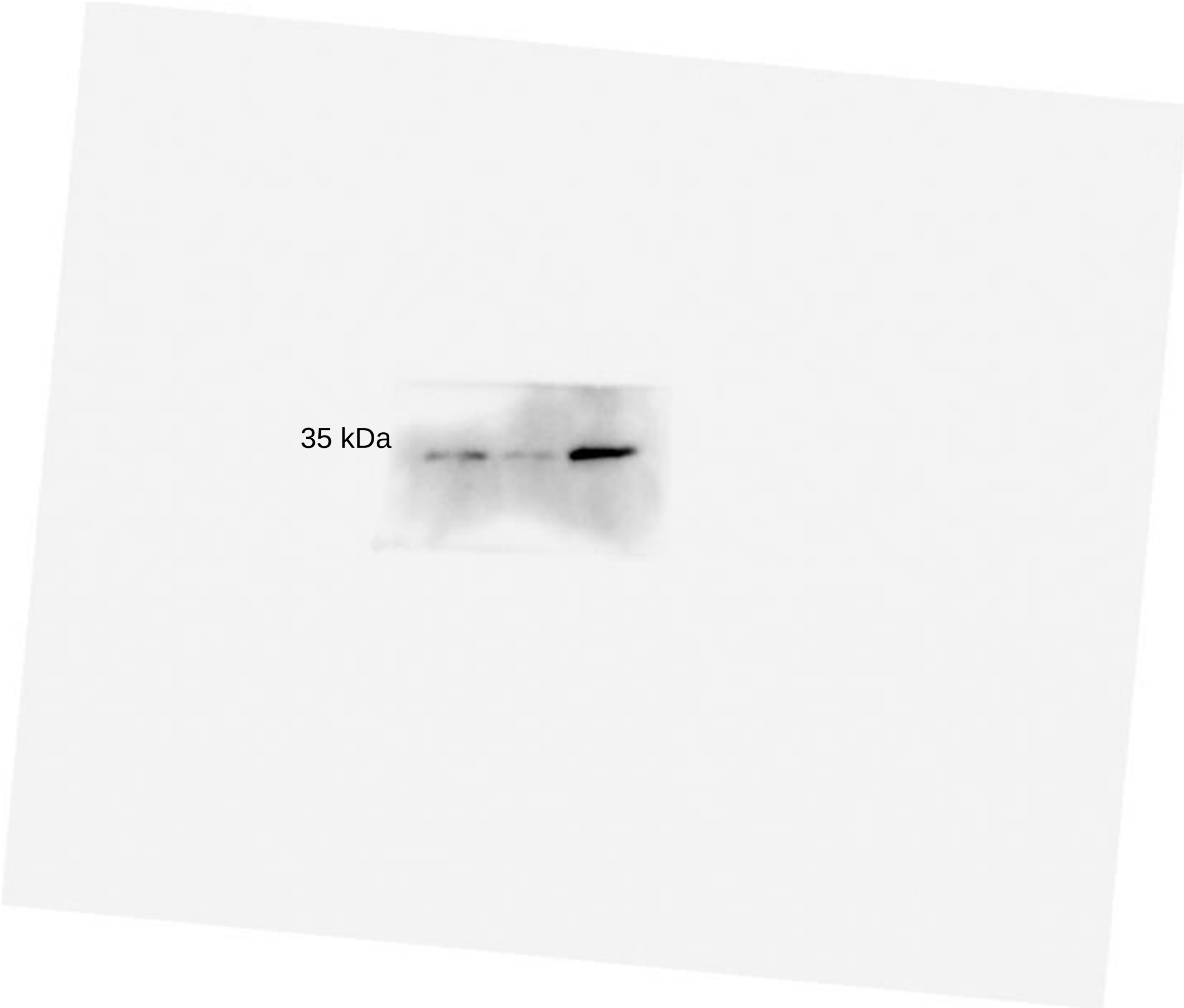


**Fig 5B**

**IB: CACT**

Groups (ES2 cells): Input, IgG (IP), CACT (IP)

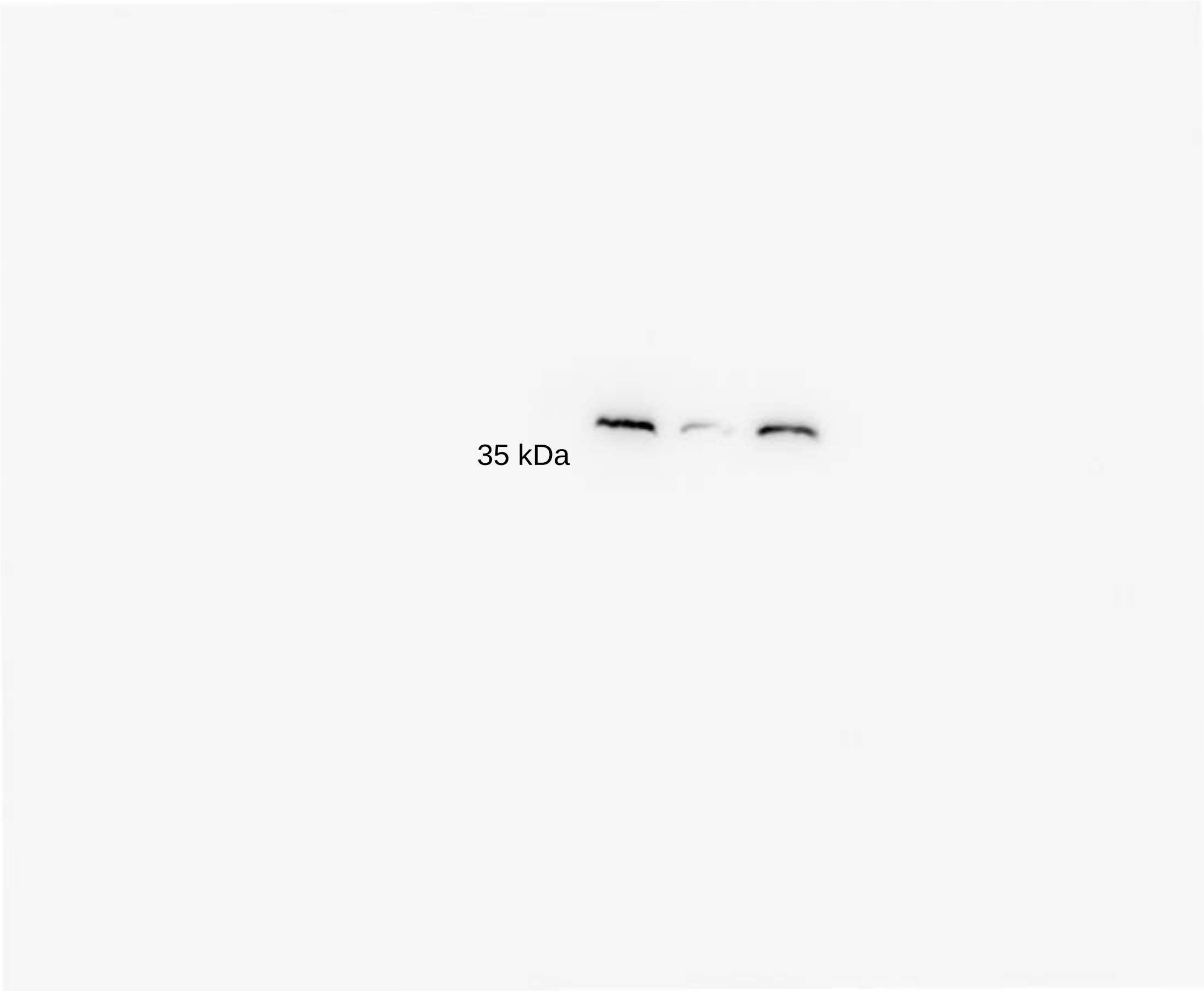


**Fig 5B**

**IB: SLC25A46**

Groups (ES2 cells): Input, IgG (IP), CACT (IP)

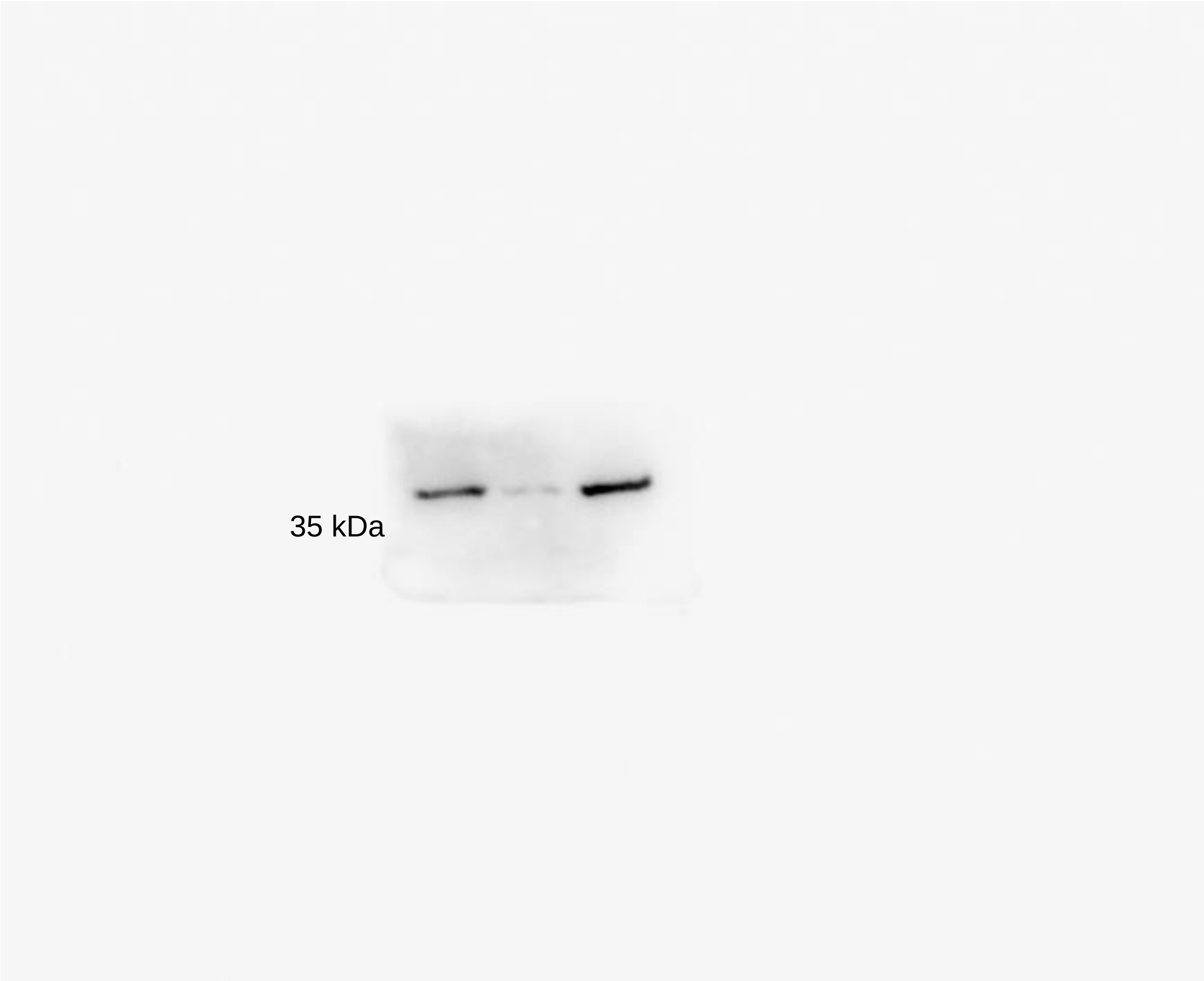


**Fig 5B**

**IB: SLC25A46**

Groups (SKOV3 cells): Input, IgG (IP), SLC25A46 (IP)

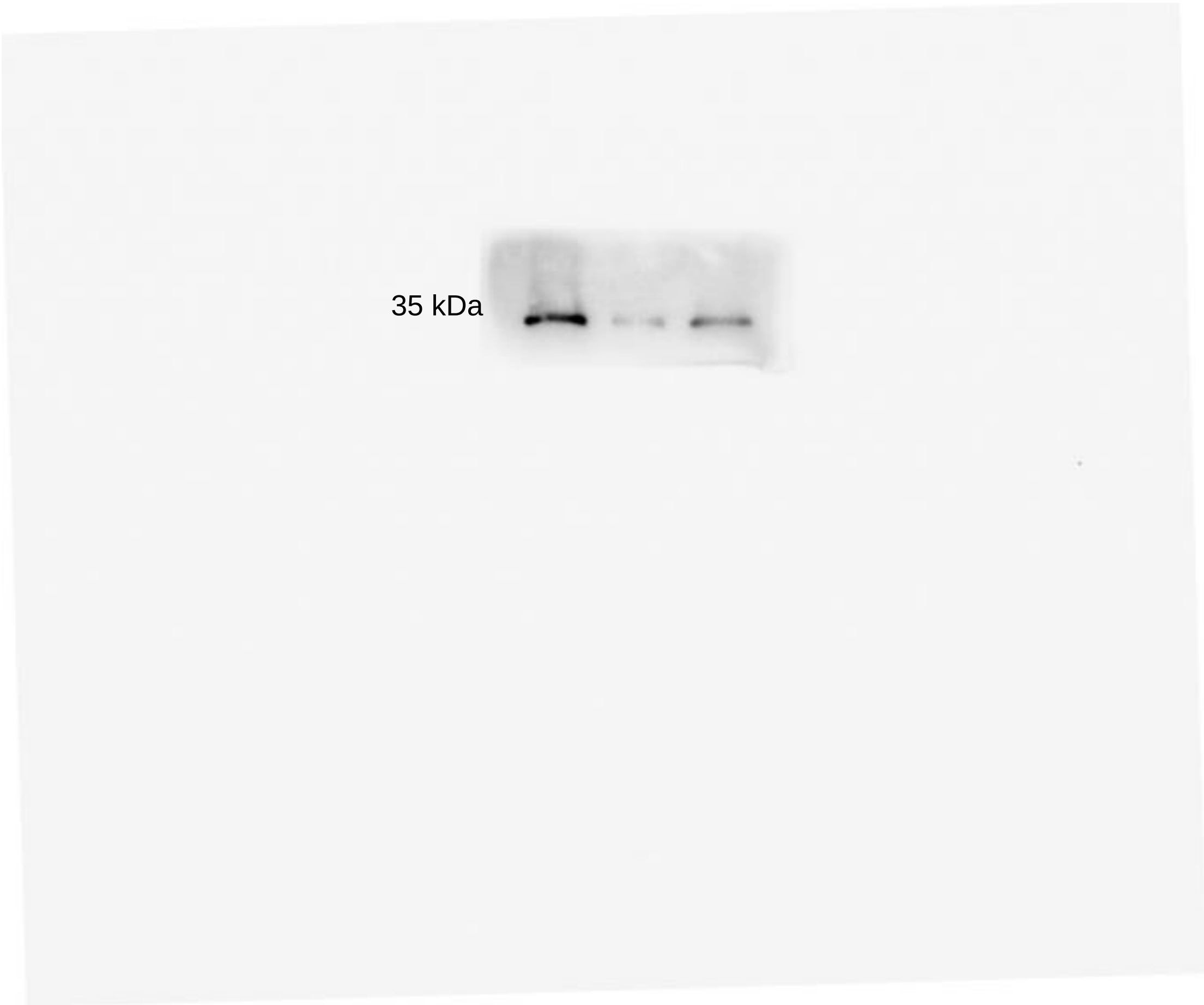


**Fig 5B**

**IB: CACT**

Groups (SKOV3 cells): Input, IgG (IP), SLC25A46 (IP)

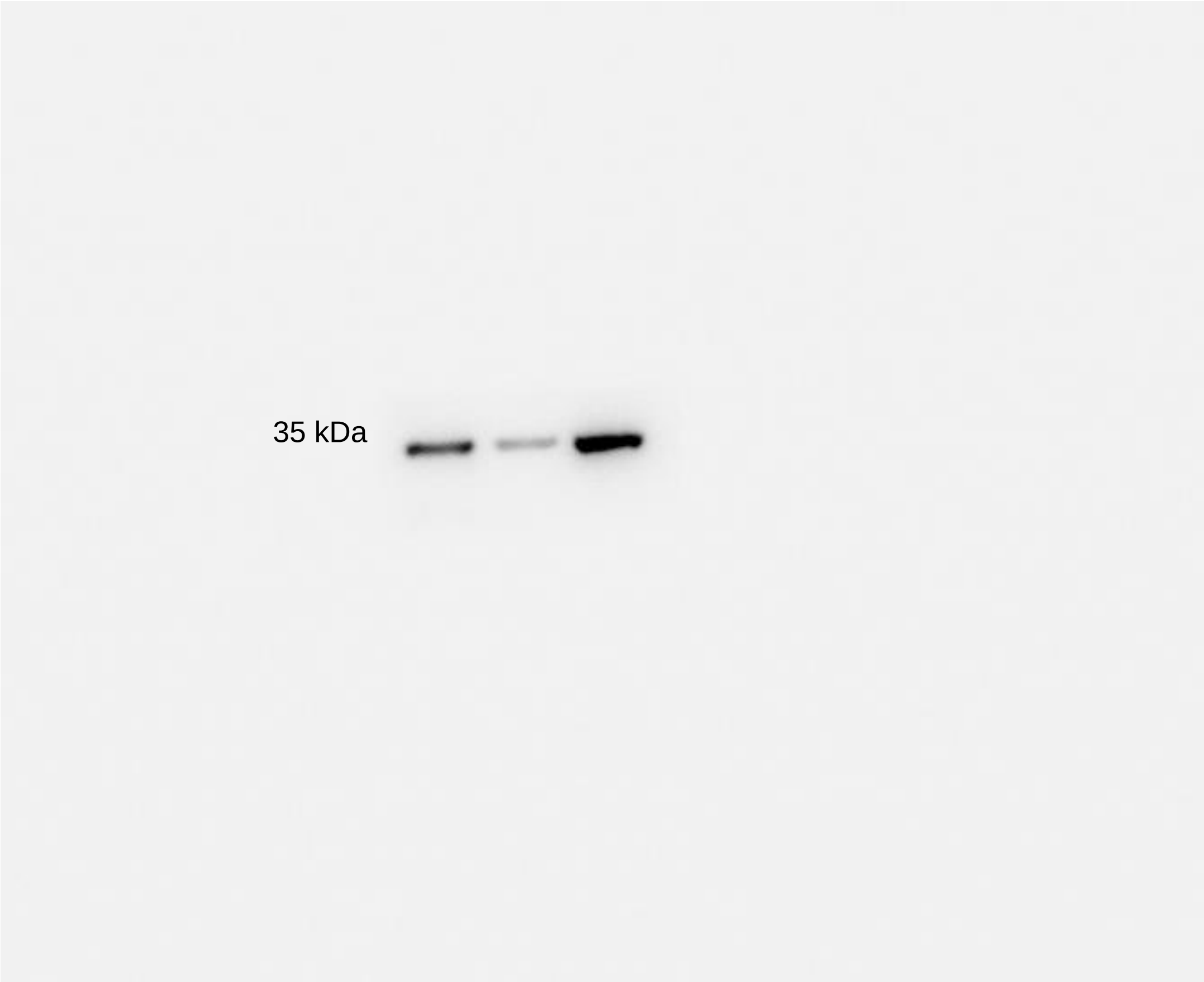


**Fig 5B**

**IB: CACT**

Groups (SKOV3 cells): Input, IgG (IP), CACT (IP)

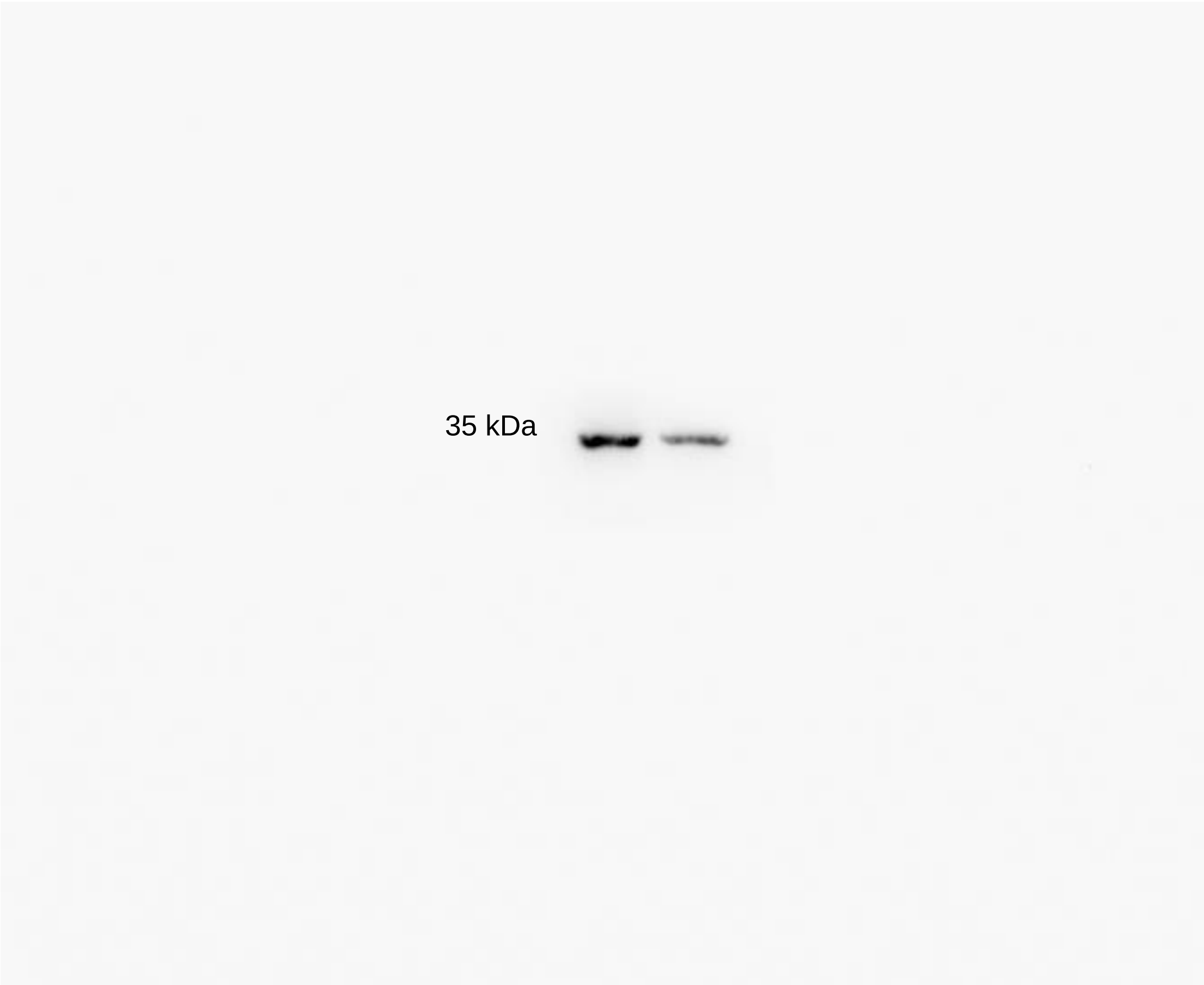


**Fig 5D**

**IB: CACT**

Groups (ES2 cells): shCtrl, shSLC25A46

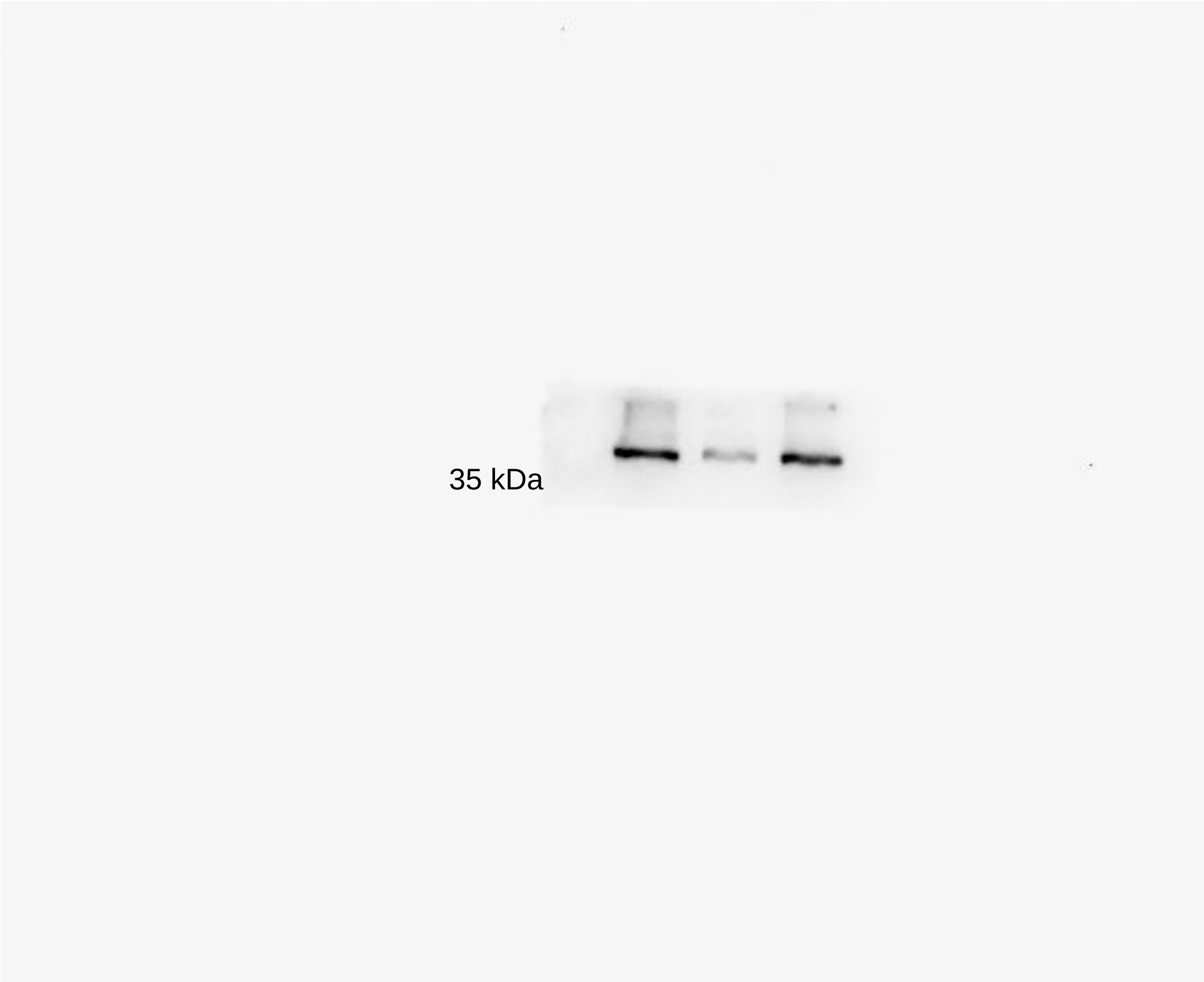


**Fig 5B**

**IB: SLC25A46**

Groups (SKOV3 cells): Input, IgG (IP), CACT (IP)

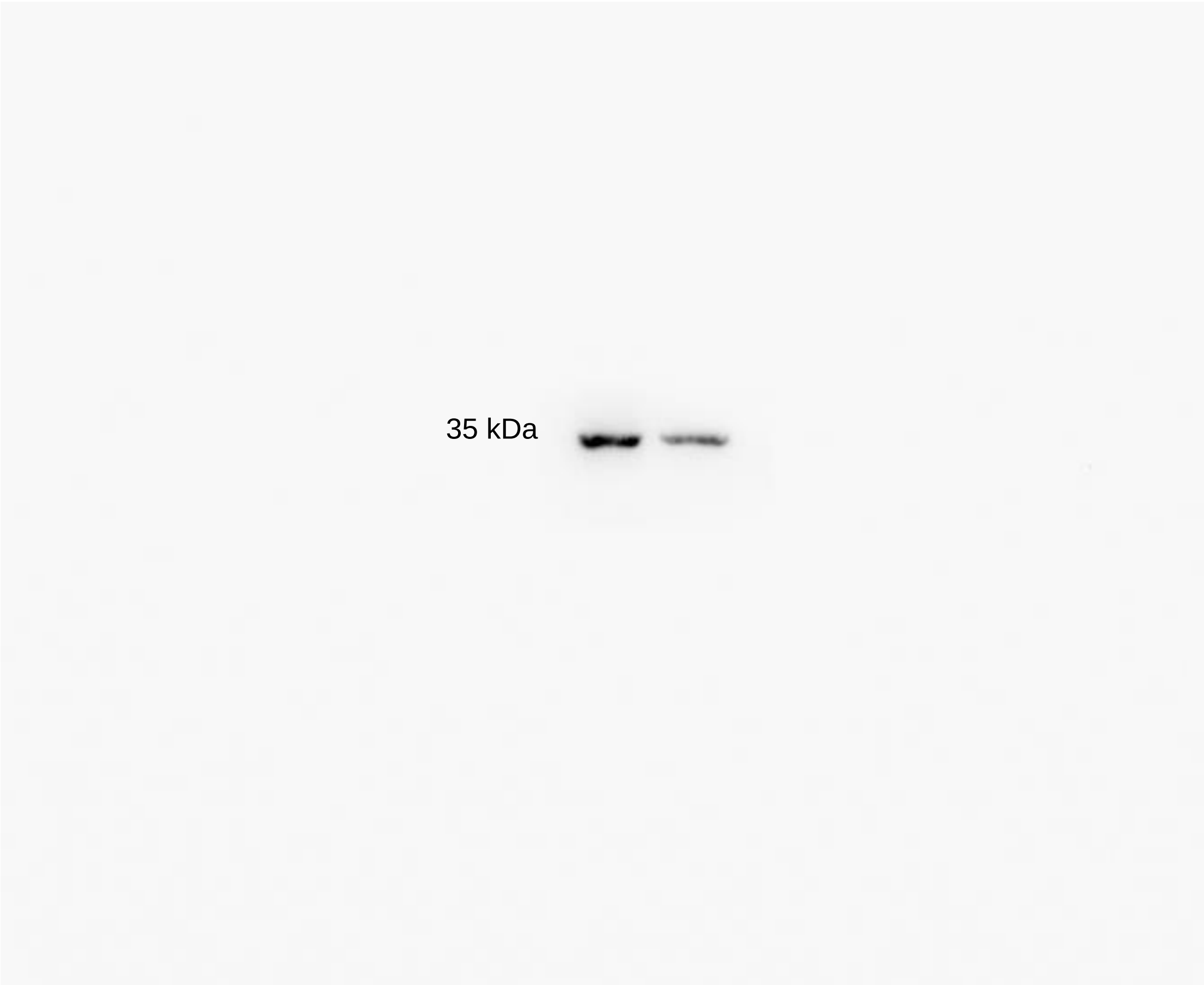


**Fig 5D**

**IB: CACT**

Groups (ES2 cells): shCtrl, shSLC25A46

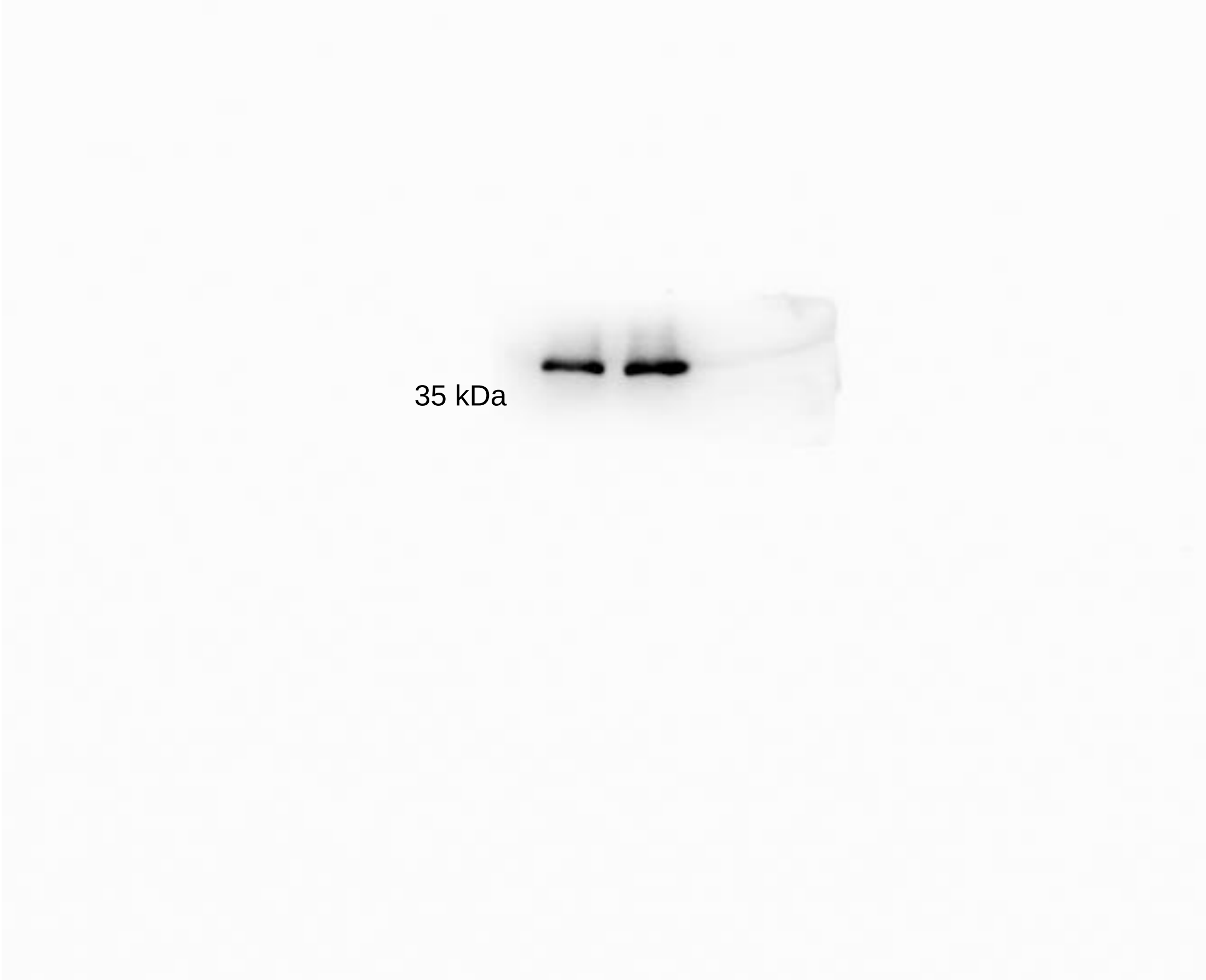


**Fig 5D**

**IB: β-actin**

Groups (ES2 cells): shCtrl, shSLC25A46

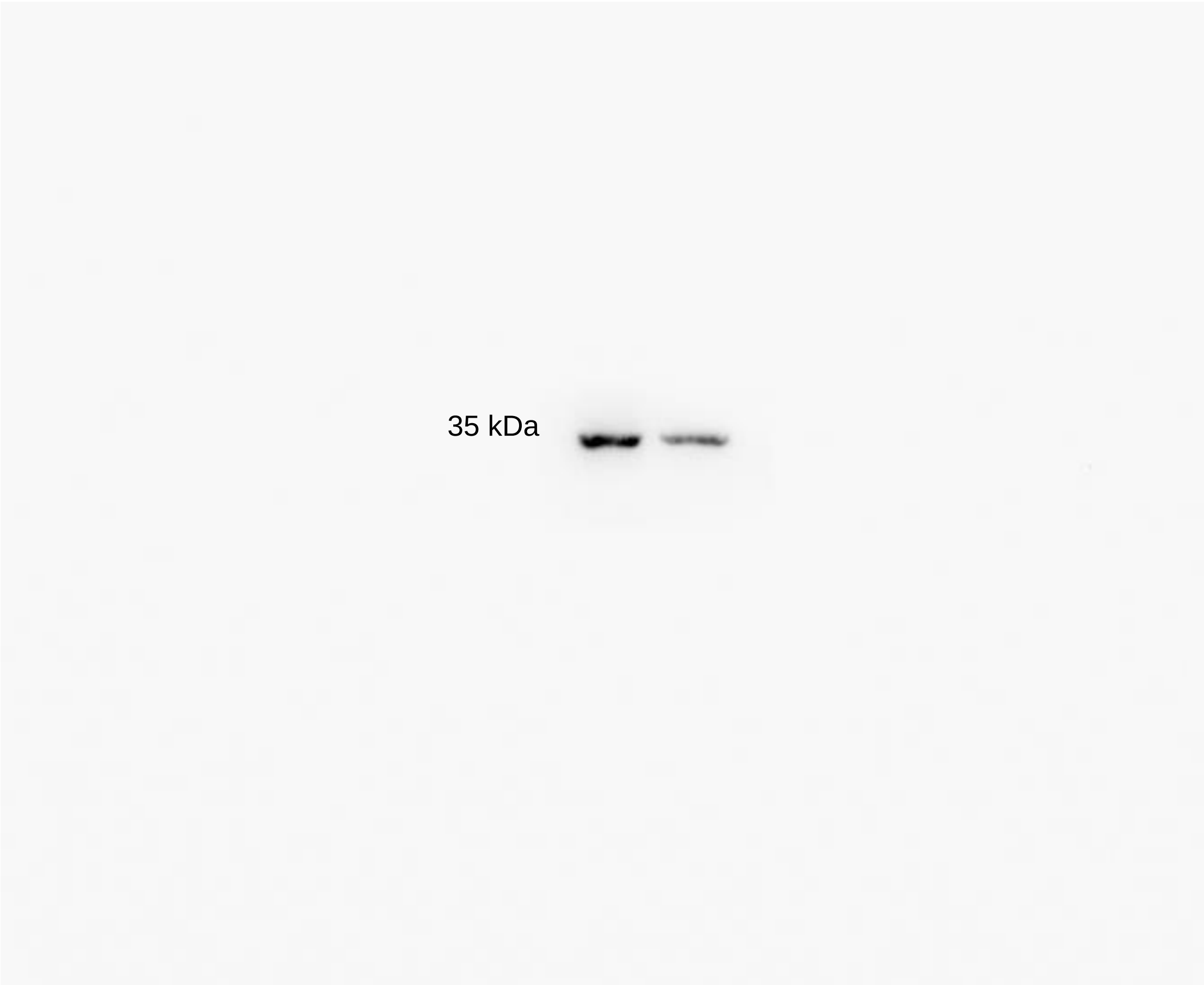


**Fig 5D**

**IB: CACT**

Groups (SKOV3 cells): shCtrl, shSLC25A46

35 kDa
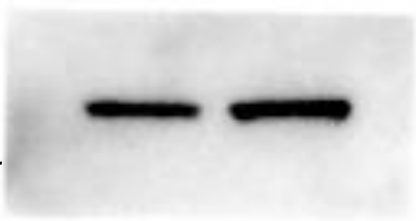

**Fig 5D**

**IB: β-actin**

Groups (SKOV3 cells): shCtrl, shSLC25A46

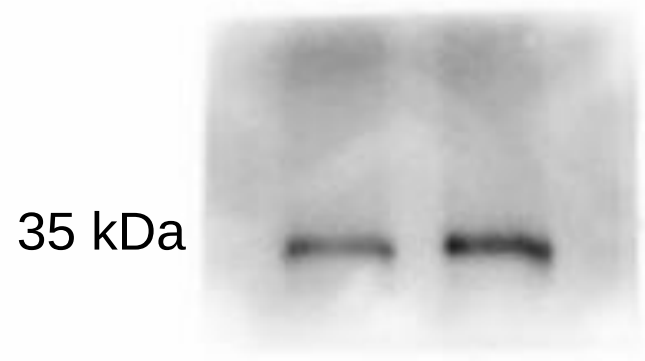


**Fig 5D**

**IB: CACT**

Groups (HEY cells): EV, SLC25A46

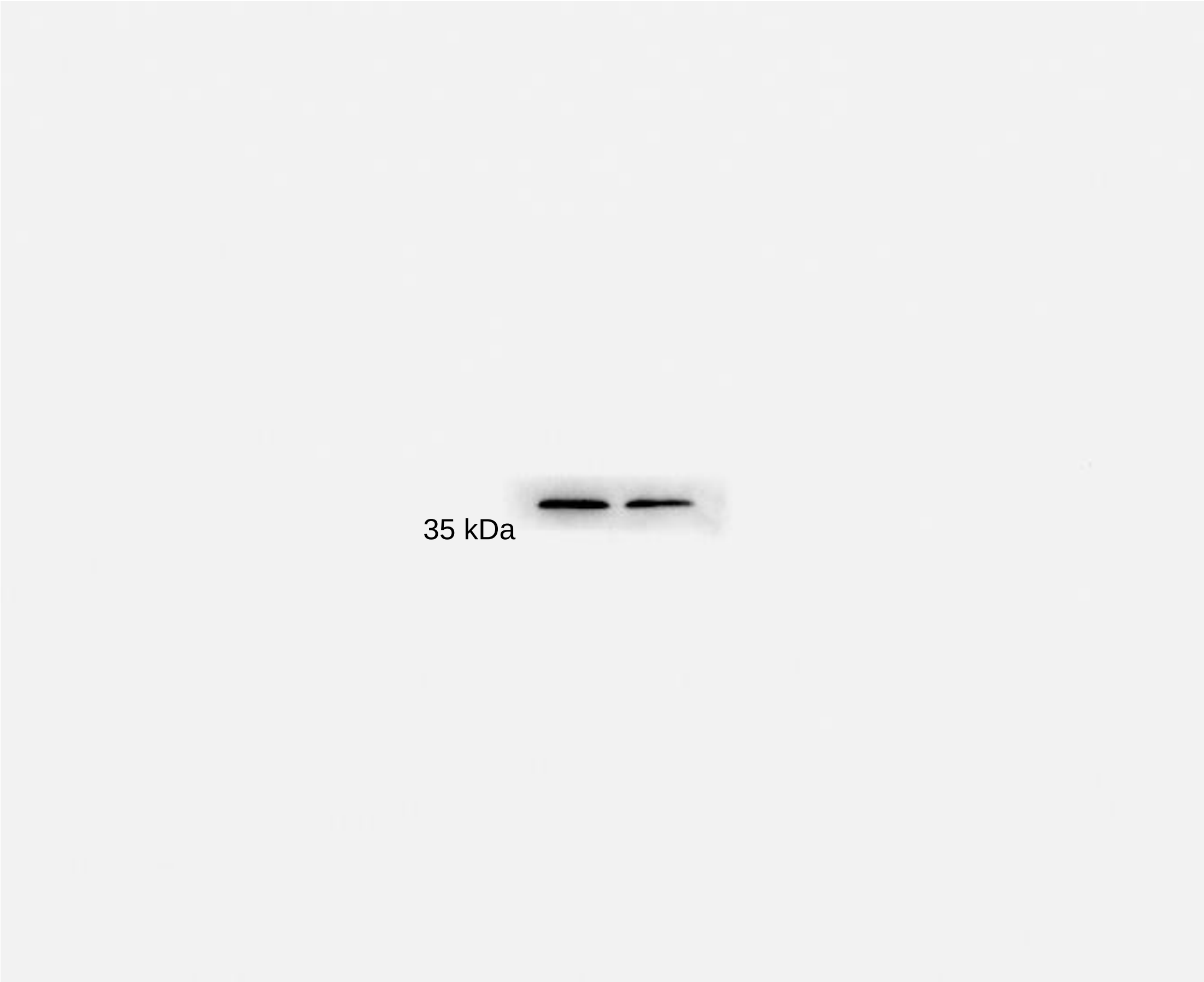


**Fig 5D**

**IB: β-actin**

Groups (HEY cells): EV, SLC25A46

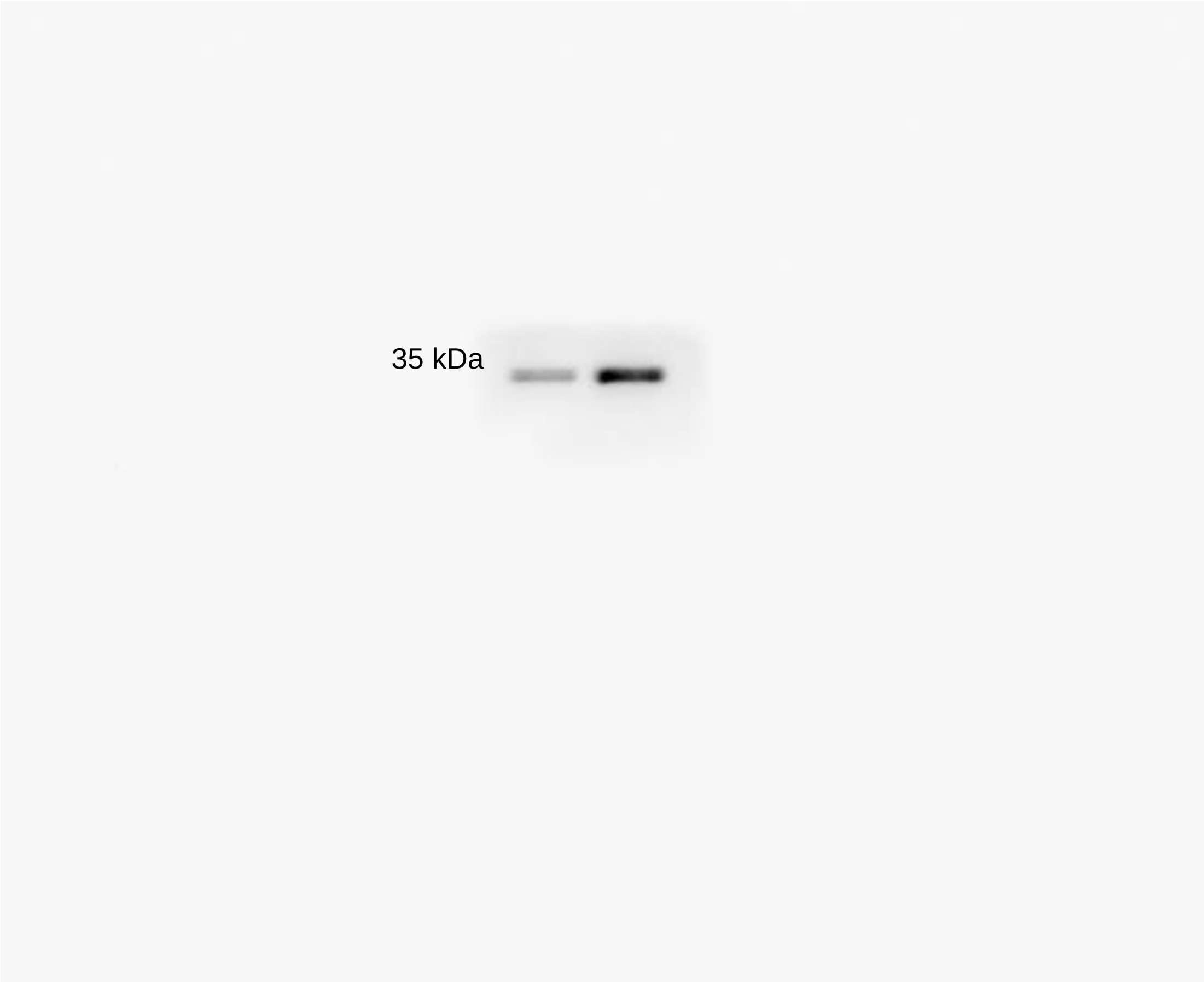


**Fig 5D**

**IB: CACT**

Groups (OVCAR3 cells): EV, SLC25A46

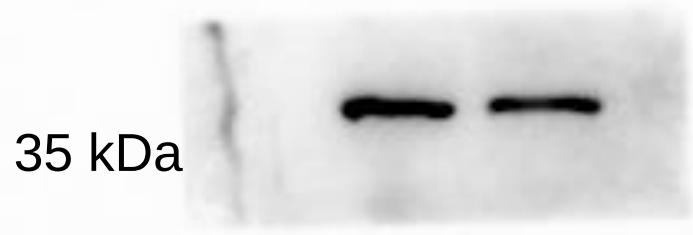


**Fig 5D**

**IB: β-actin**

Groups (OVCAR3 cells): EV, SLC25A46

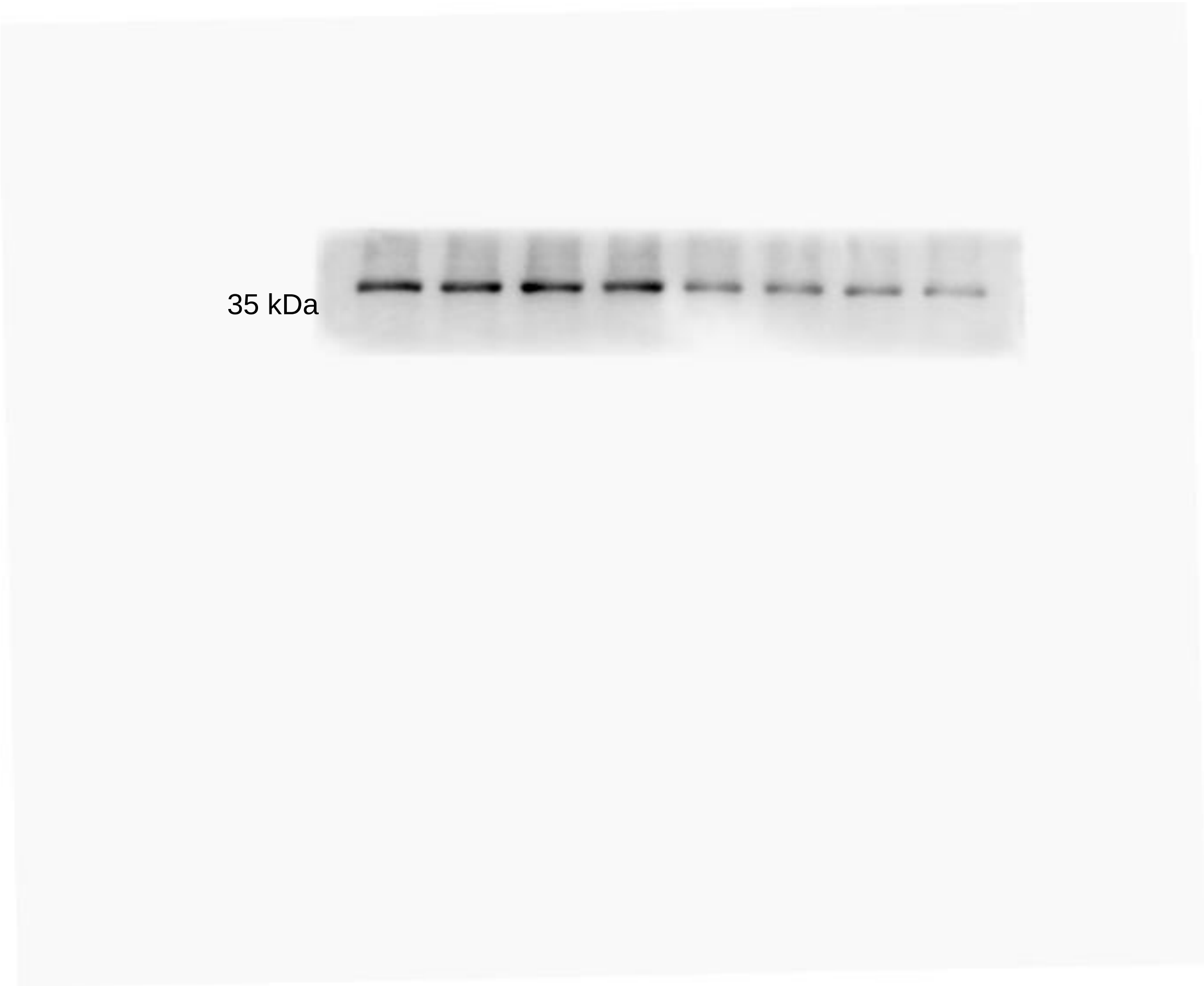


**Fig 6A**

**IB: SLC25A46**

Groups (ES2 cells): shCtrl (CHX: 0h, 1h, 2h, 4h), shSLC25A46 (CHX: 0h, 1h, 2h, 4h).

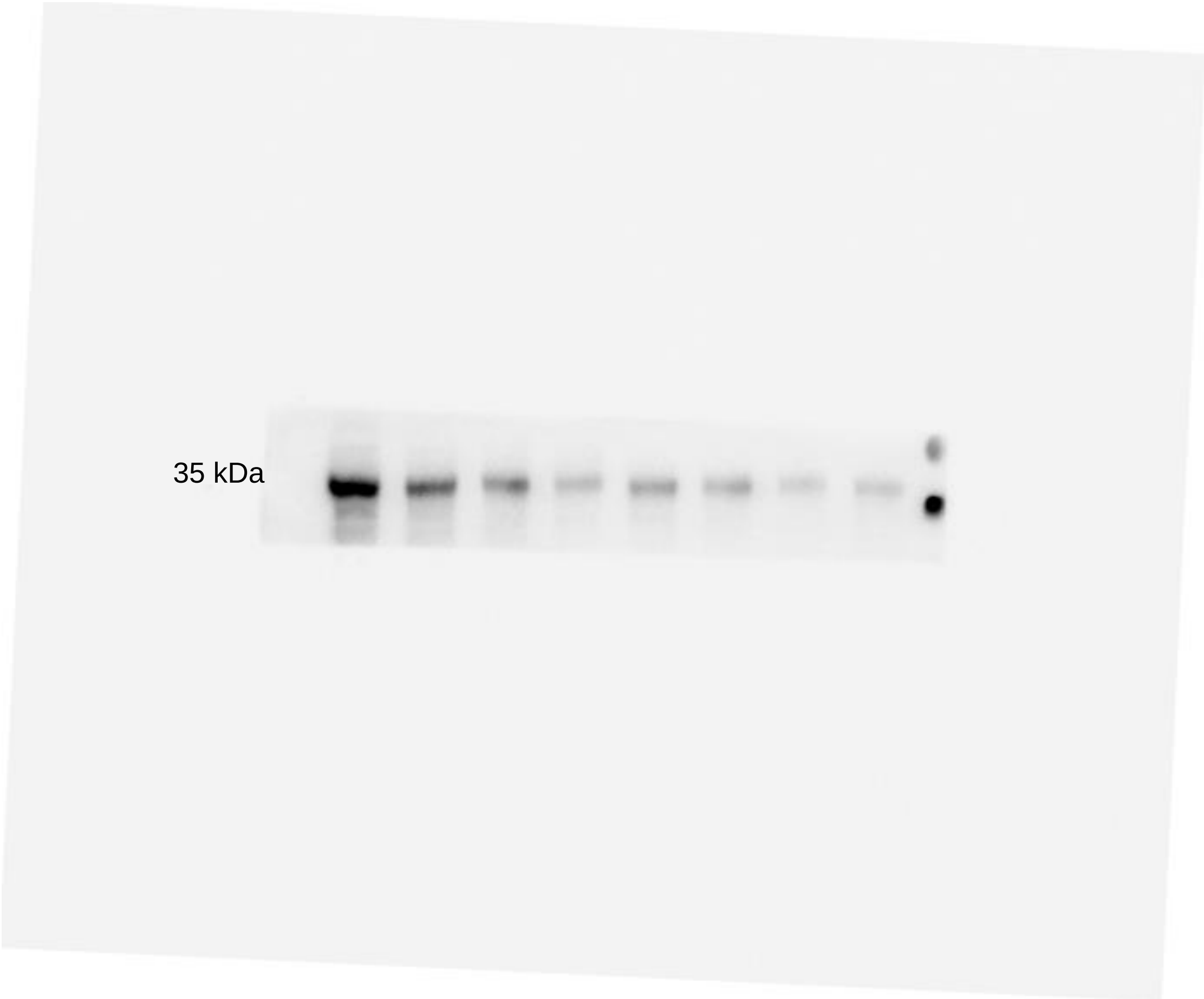


**Fig 6A**

**IB: CACT**

Groups (ES2 cells): shCtrl (CHX: 0h, 1h, 2h, 4h), shSLC25A46 (CHX: 0h, 1h, 2h, 4h)

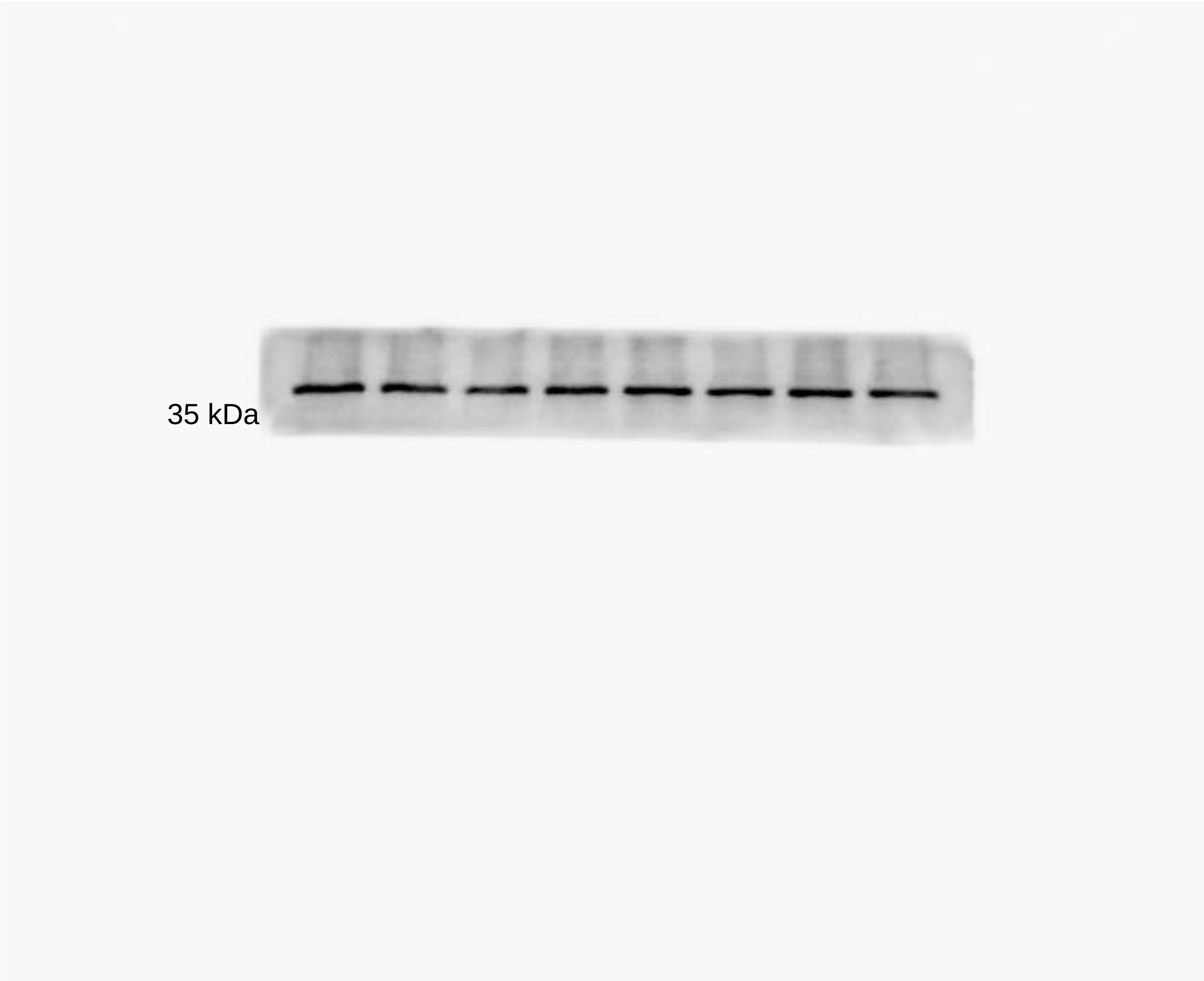


**Fig 6A**

**IB: β-actin**

Groups (ES2 cells): shCtrl (CHX: 0h, 1h, 2h, 4h), shSLC25A46 (CHX: 0h, 1h, 2h, 4h)

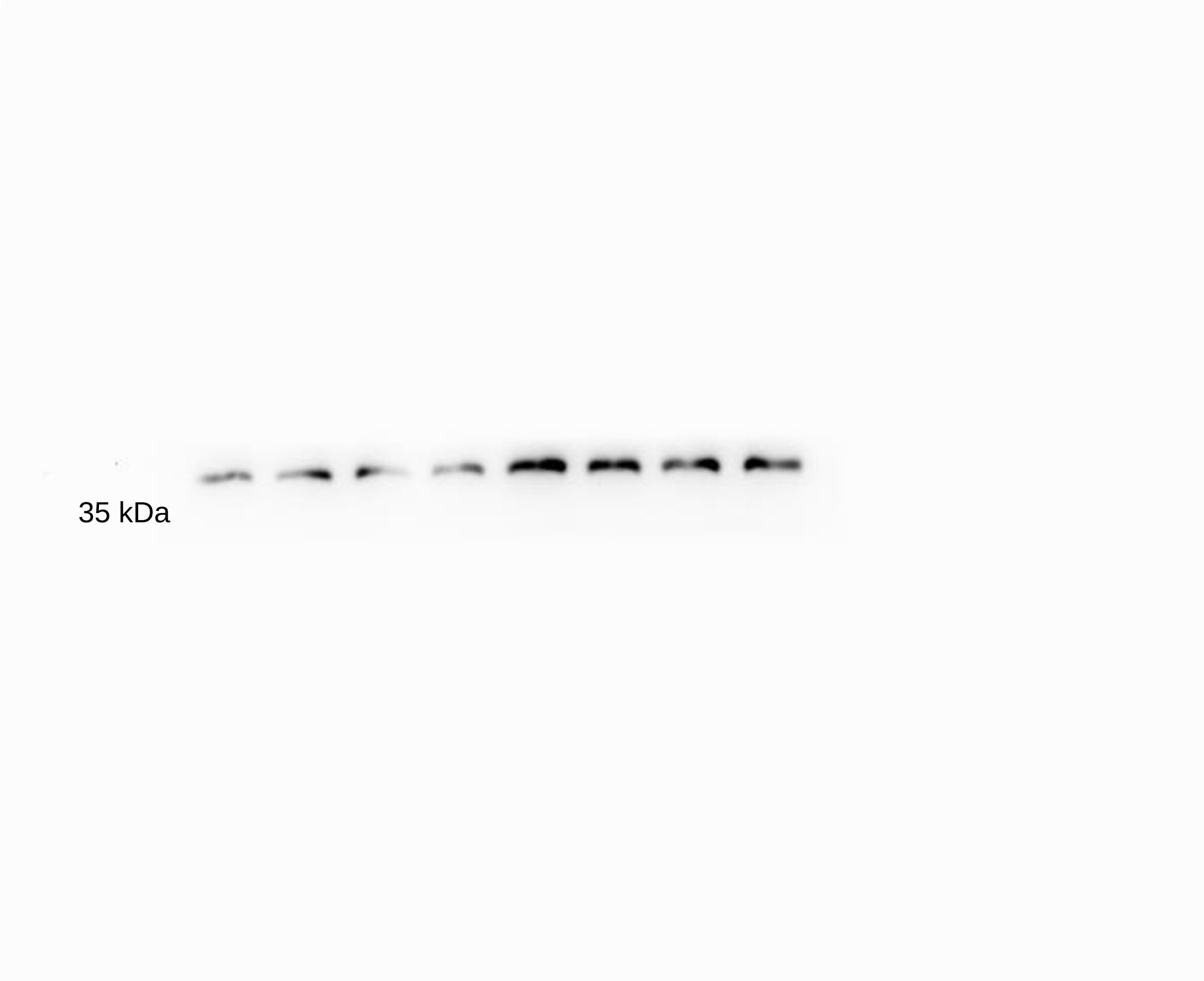


**Fig 6A**

**IB: SLC25A46**

Groups (HEY cells): EV (CHX: 0h, 1h, 2h, 4h), SLC25A46 (CHX: 0h, 1h, 2h, 4h).

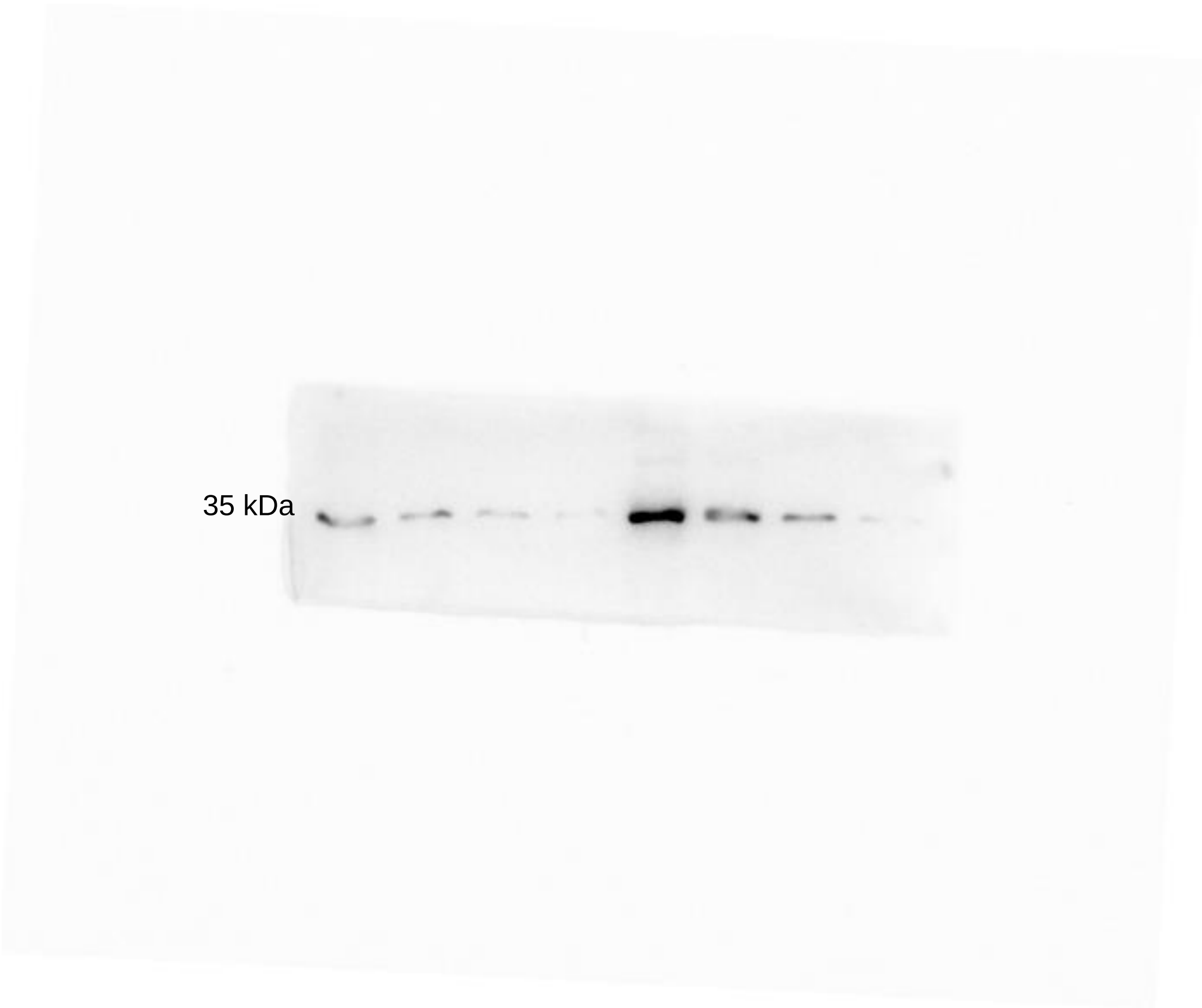


**Fig 6A**

**IB: CACT**

Groups (HEY cells): EV (CHX: 0h, 1h, 2h, 4h), SLC25A46 (CHX: 0h, 1h, 2h, 4h)

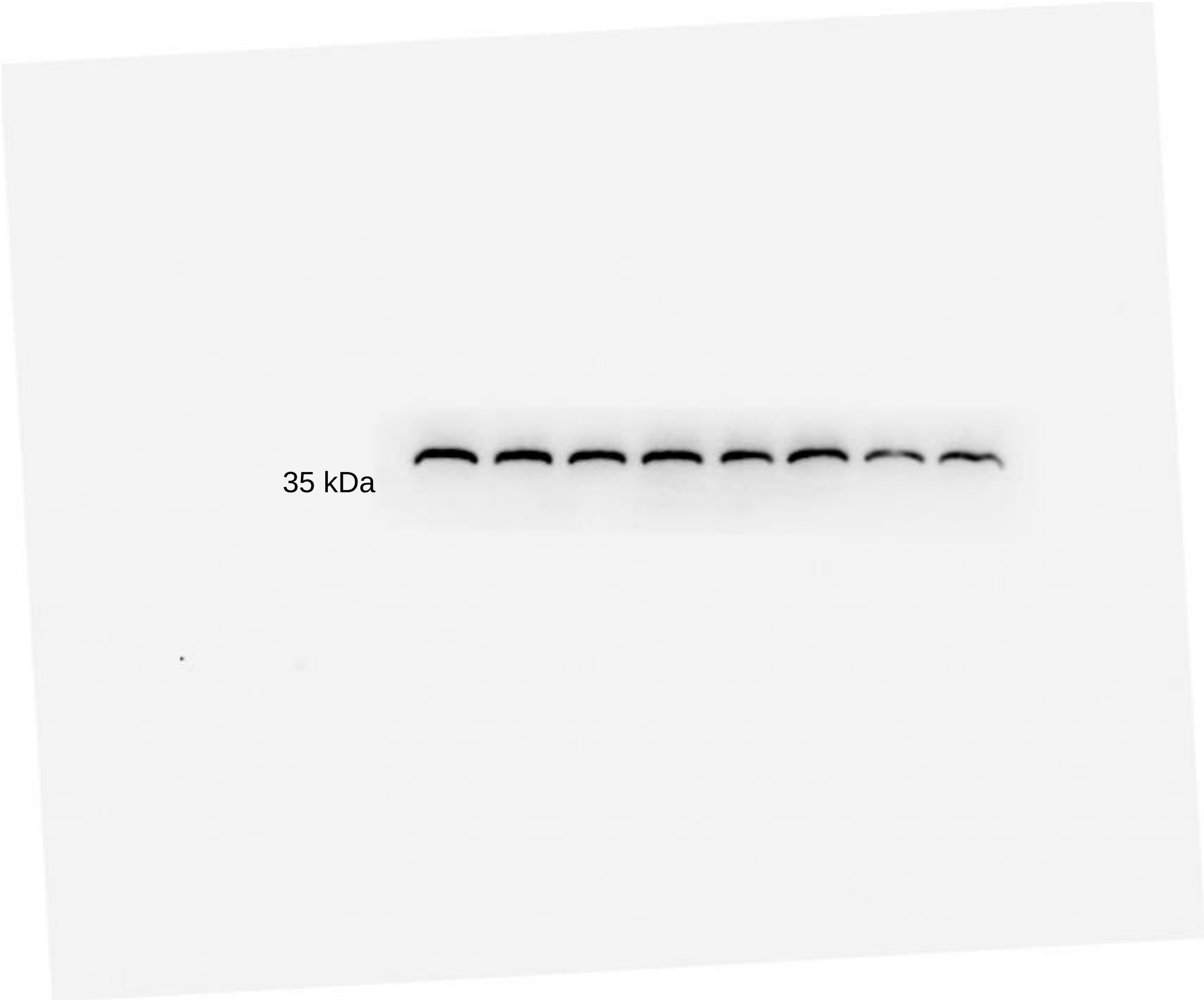


**Fig 6A**

**IB: β-actin**

Groups (HEY cells): EV (CHX: 0h, 1h, 2h, 4h), SLC25A46 (CHX: 0h, 1h, 2h, 4h)

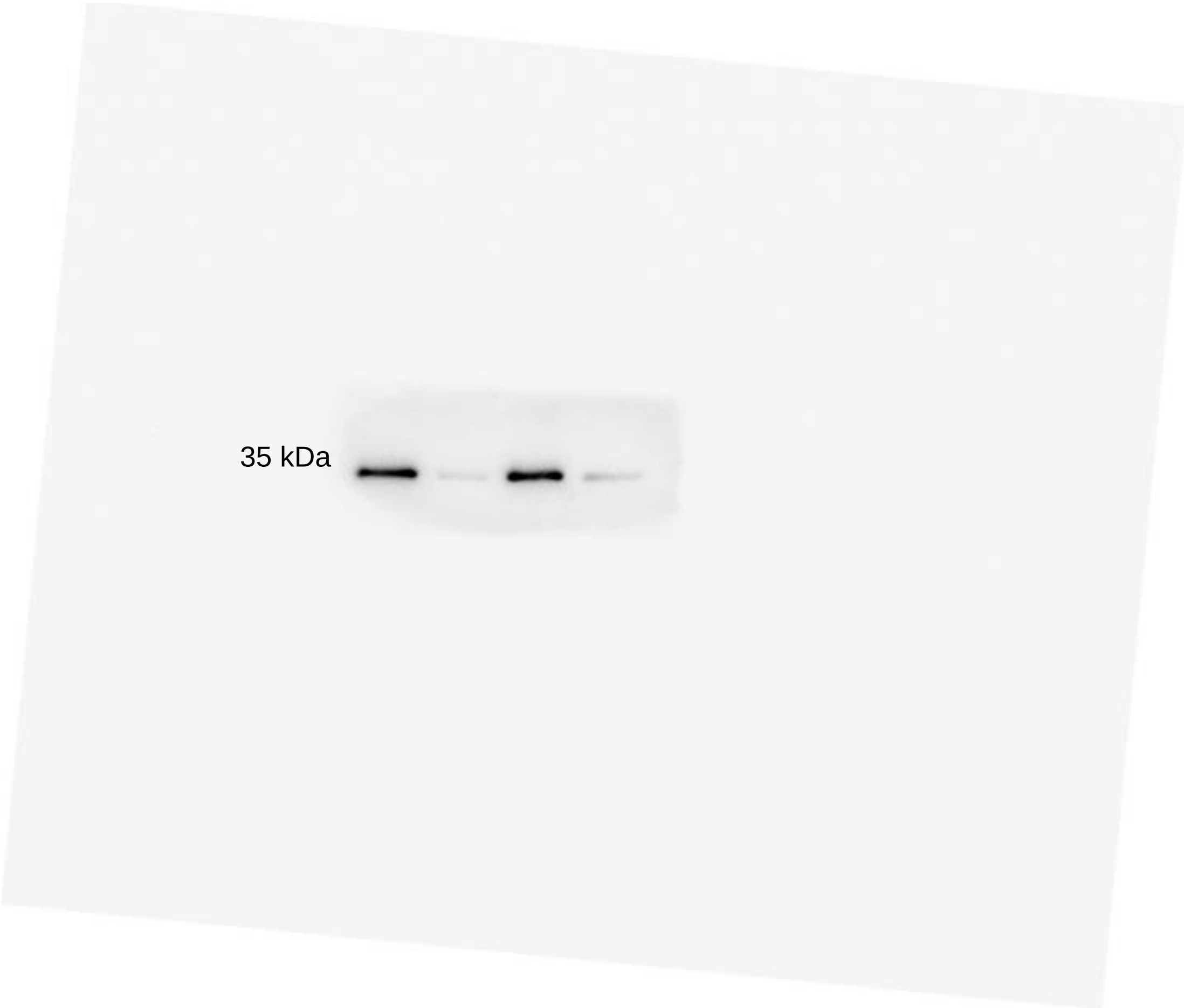


**Fig 6B**

**IB: CACT**

Groups (ES2 cells): shCtrl, SLC25A46 (DMSO, MG132, CQ)

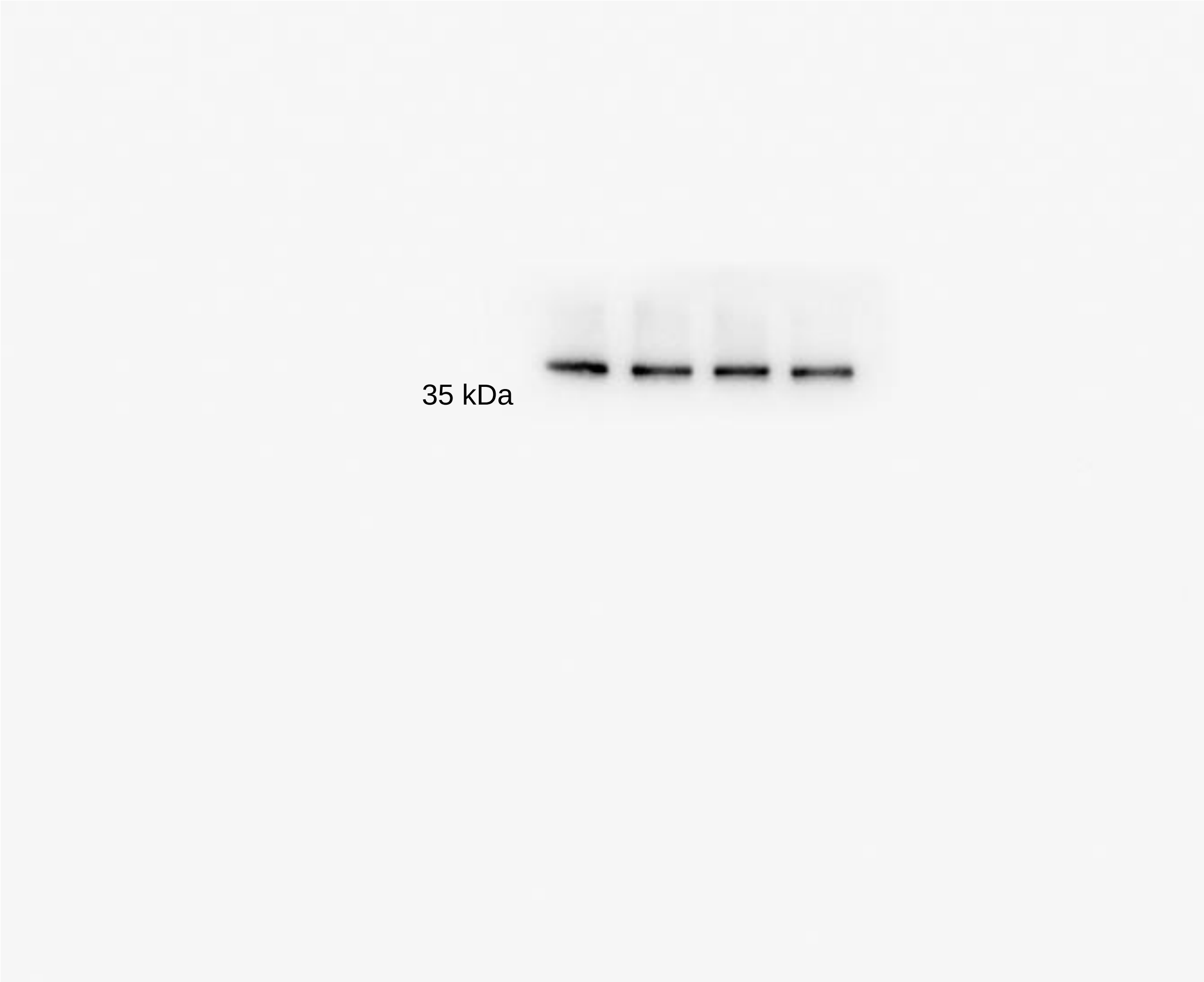


**Fig 6B**

**IB: β-actin**

Groups (ES2 cells): shCtrl, SLC25A46 (DMSO, MG132, CQ)

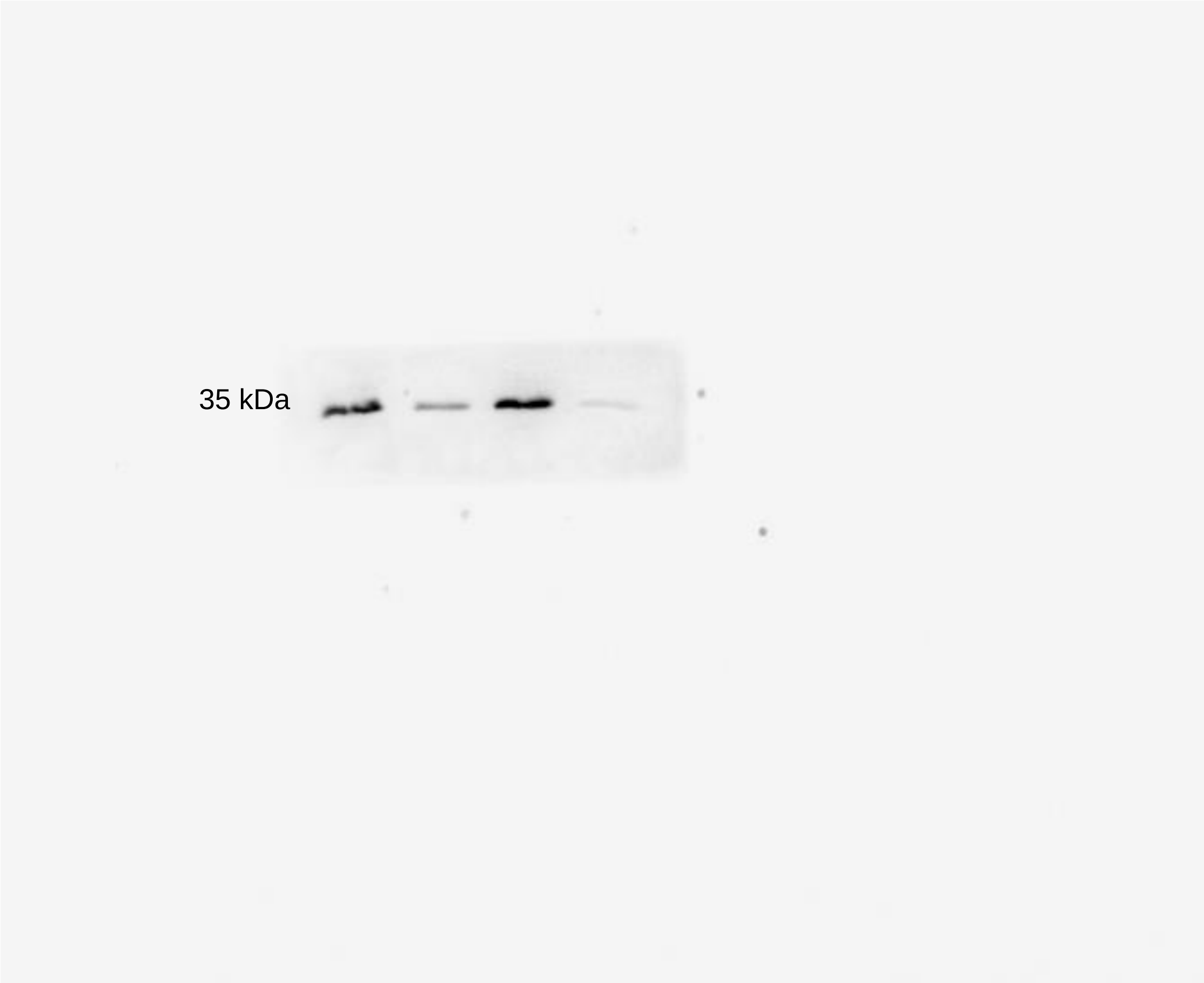


**Fig 6B**

**IB: CACT**

Groups (SKOV3 cells): shCtrl, SLC25A46 (DMSO, MG132, CQ)

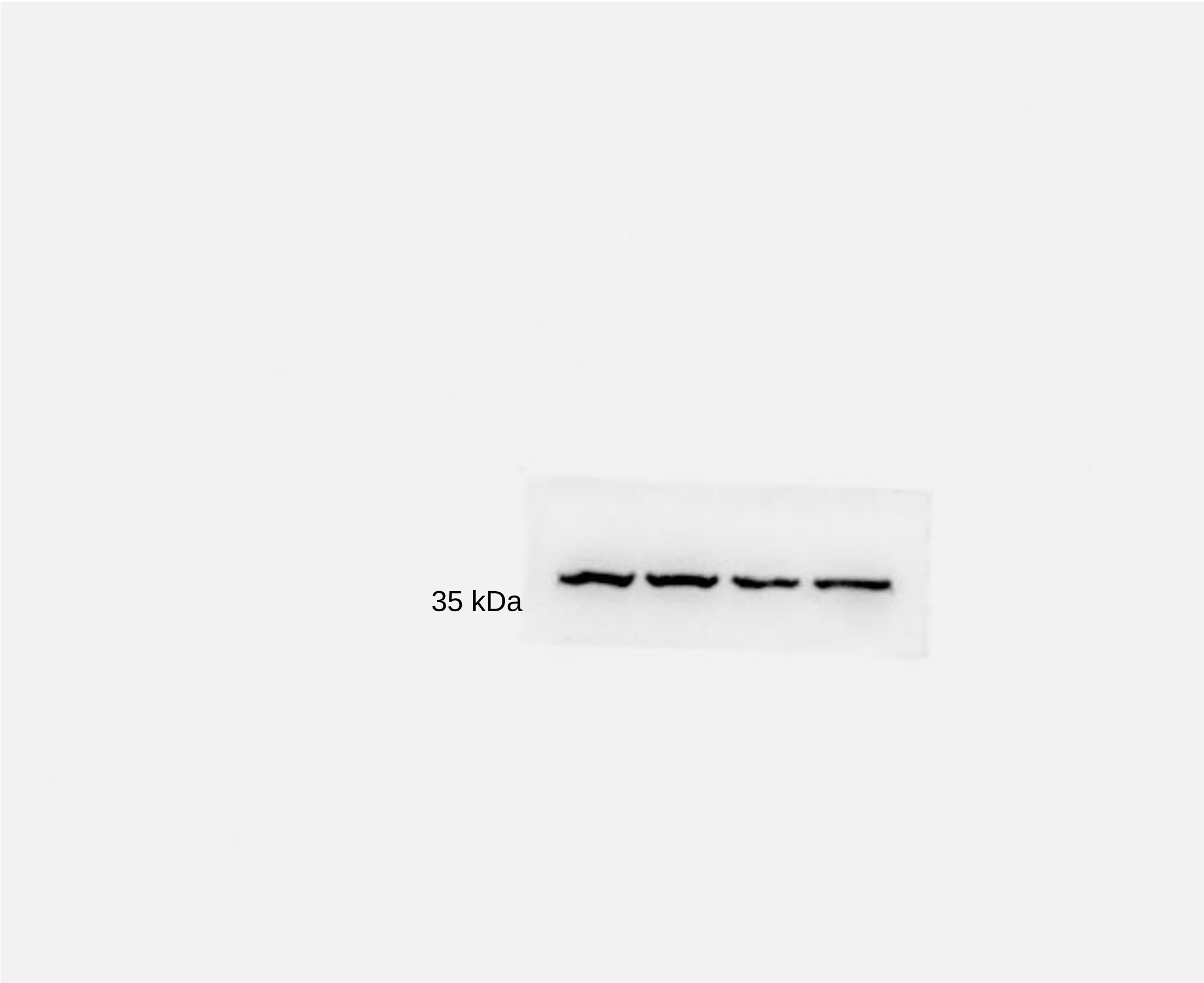


**Fig 6B**

**IB: β-actin**

Groups (SKOV3 cells): shCtrl, SLC25A46 (DMSO, MG132, CQ)

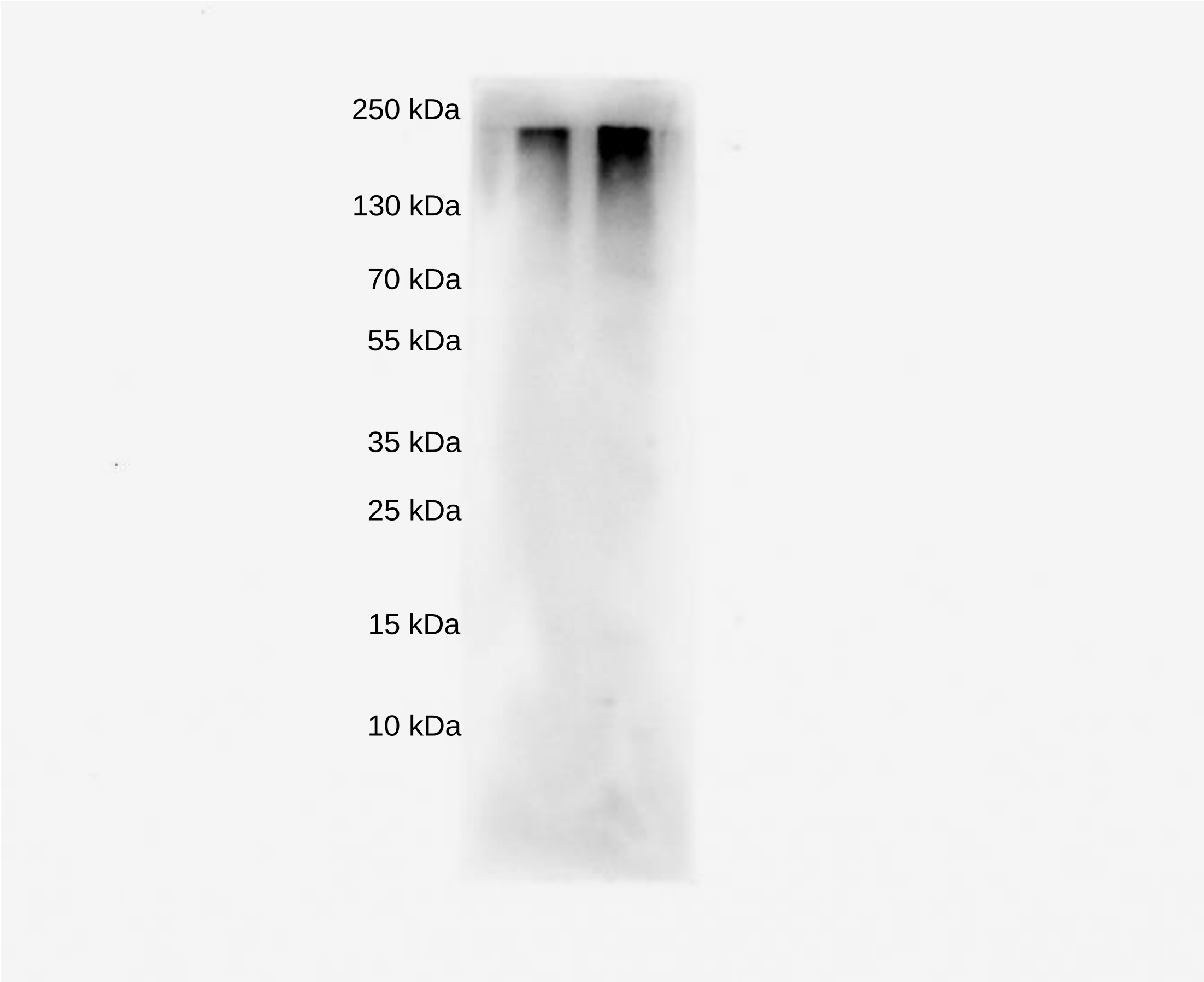


**Fig 6C**

**IB: Anti-Ub**

Groups (ES2 cells): shCtrl+MG132, SLC25A46+MG132

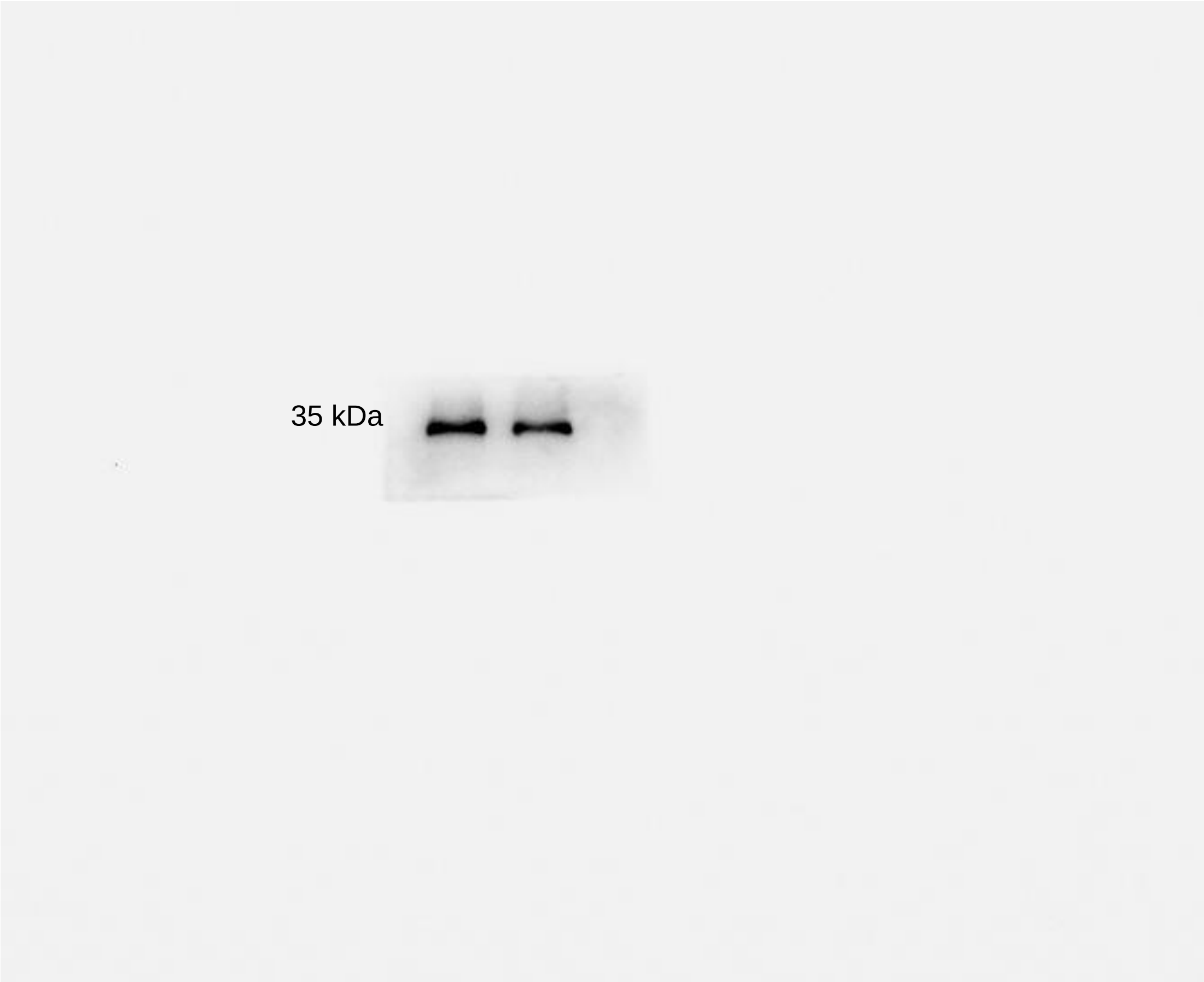


**Fig 6C**

**IB: CACT**

Groups (ES2 cells): shCtrl+MG132, SLC25A46+MG132

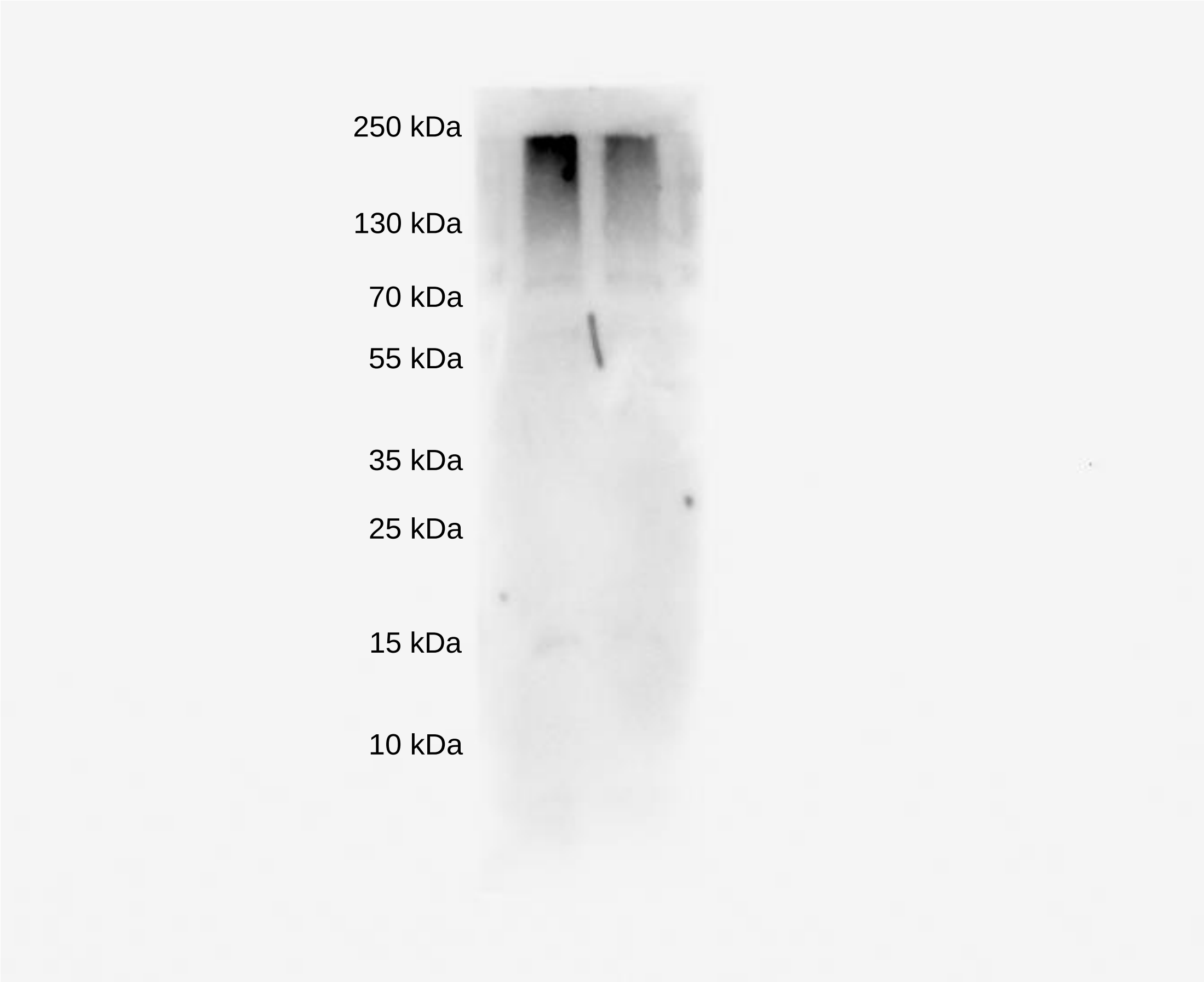


**Fig 6C**

**IB: Anti-Ub**

Groups (HEY cells): shCtrl+MG132, SLC25A46+MG132

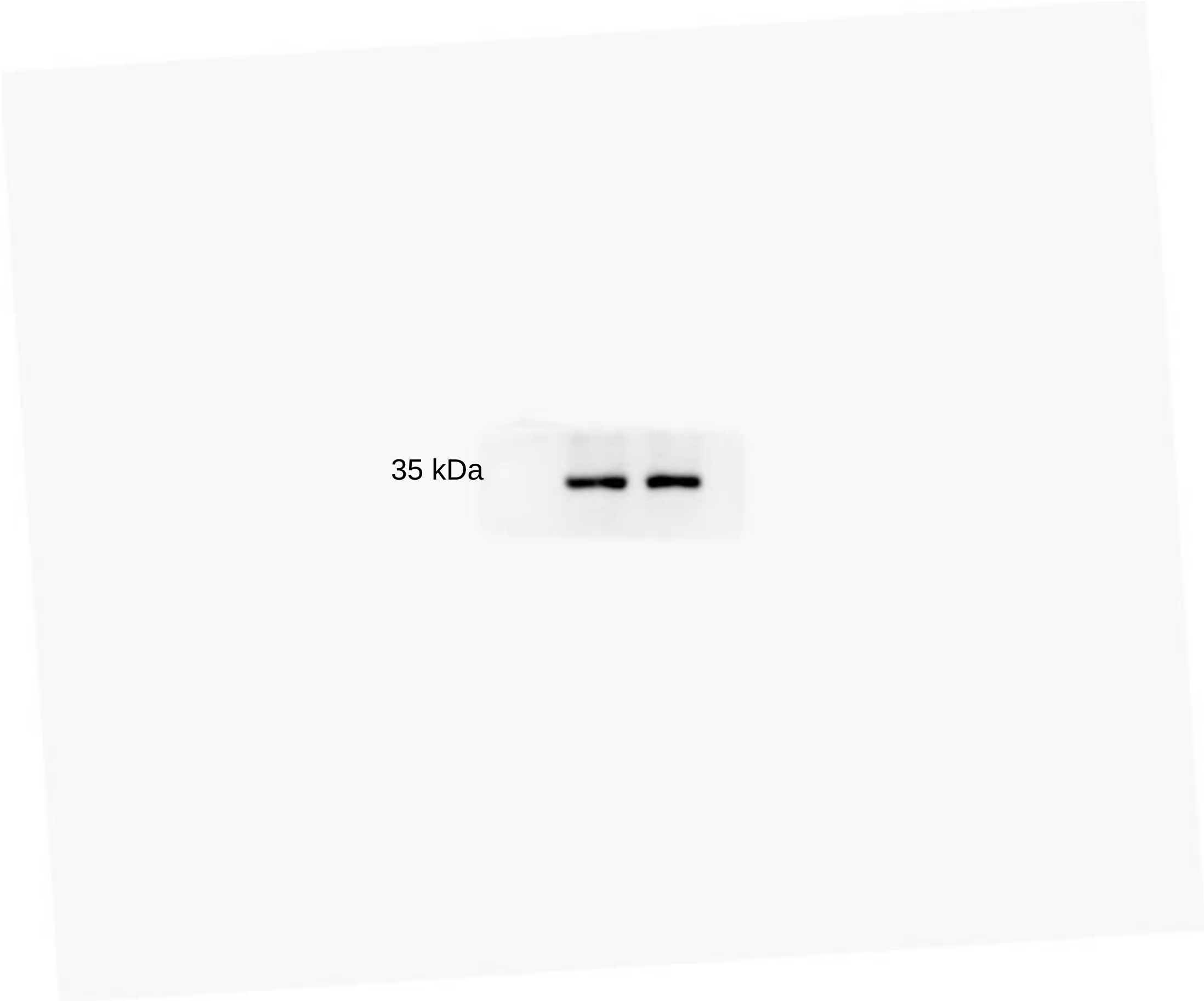


**Fig 6C**

**IB: CACT**

Groups (HEY cells): shCtrl+MG132, SLC25A46+MG132

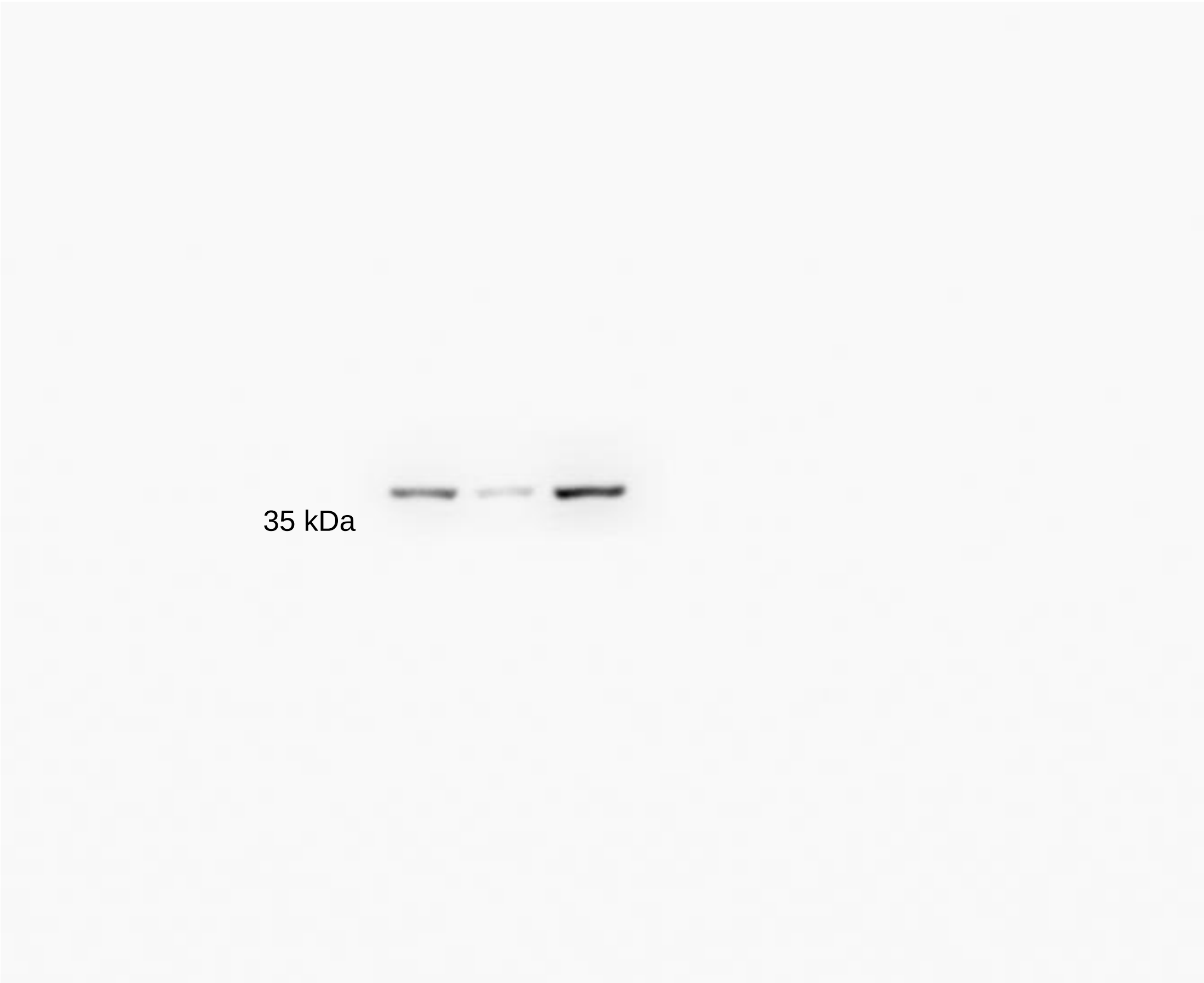


**Fig 6D**

**IB: SLC25A46**

Groups (ES2 cells): Input, IgG (IP), SLC25A46 (IP)

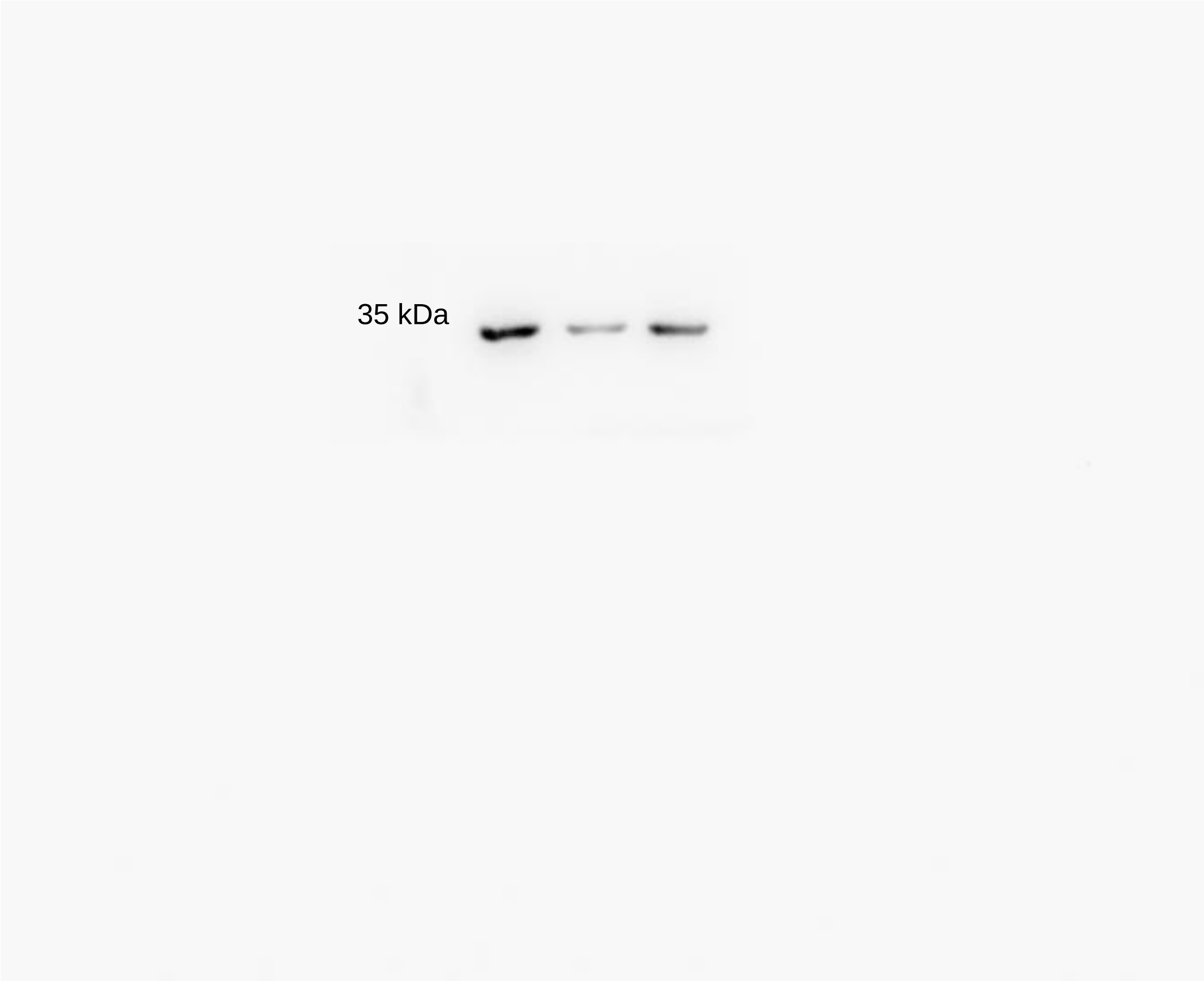


**Fig 6D**

**IB: MARCHF5**

Groups (ES2 cells): Input, IgG (IP), SLC25A46 (IP)

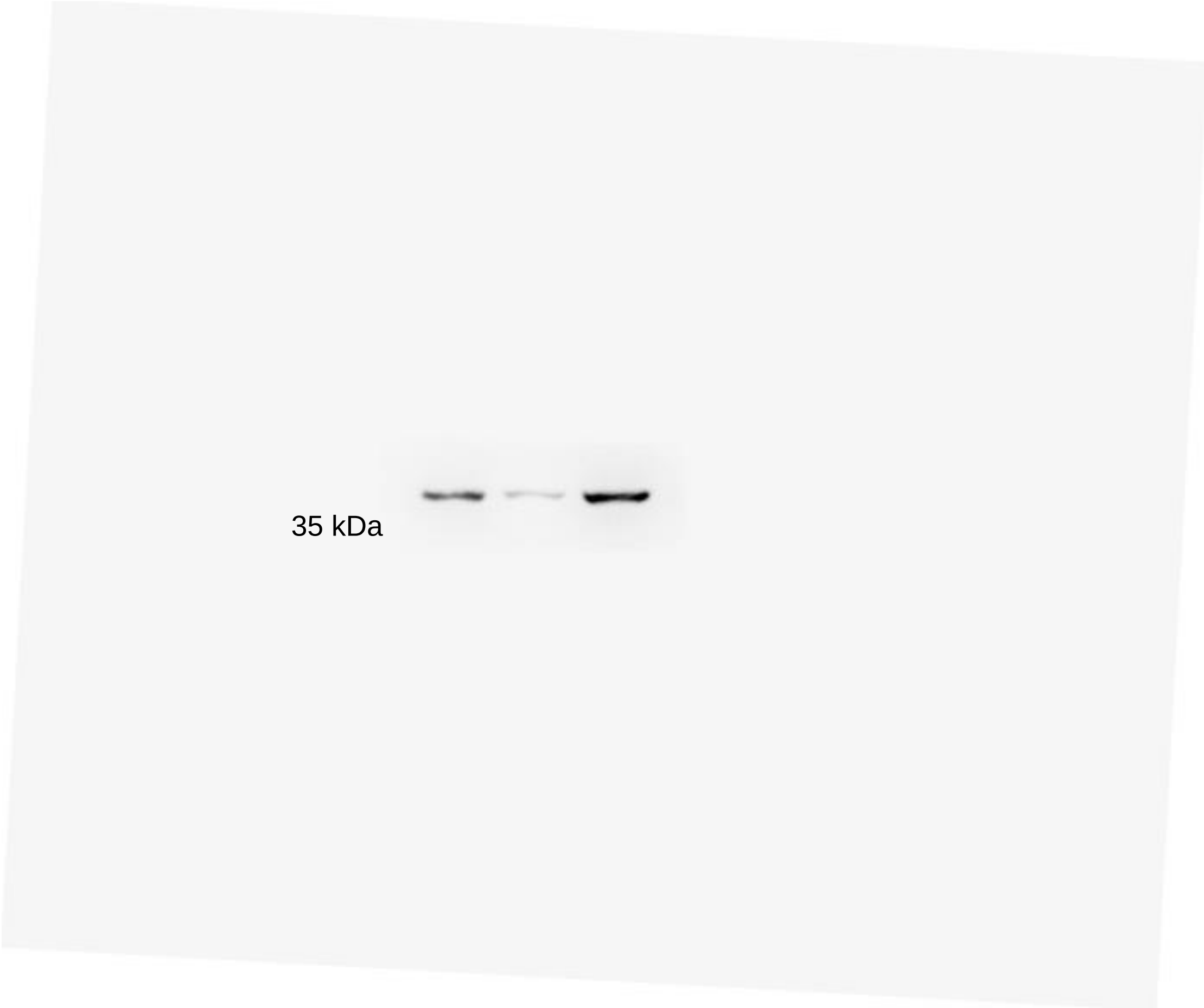


**Fig 6D**

**IB: SLC25A46**

Groups (SKOV3 cells): Input, IgG (IP), SLC25A46 (IP)

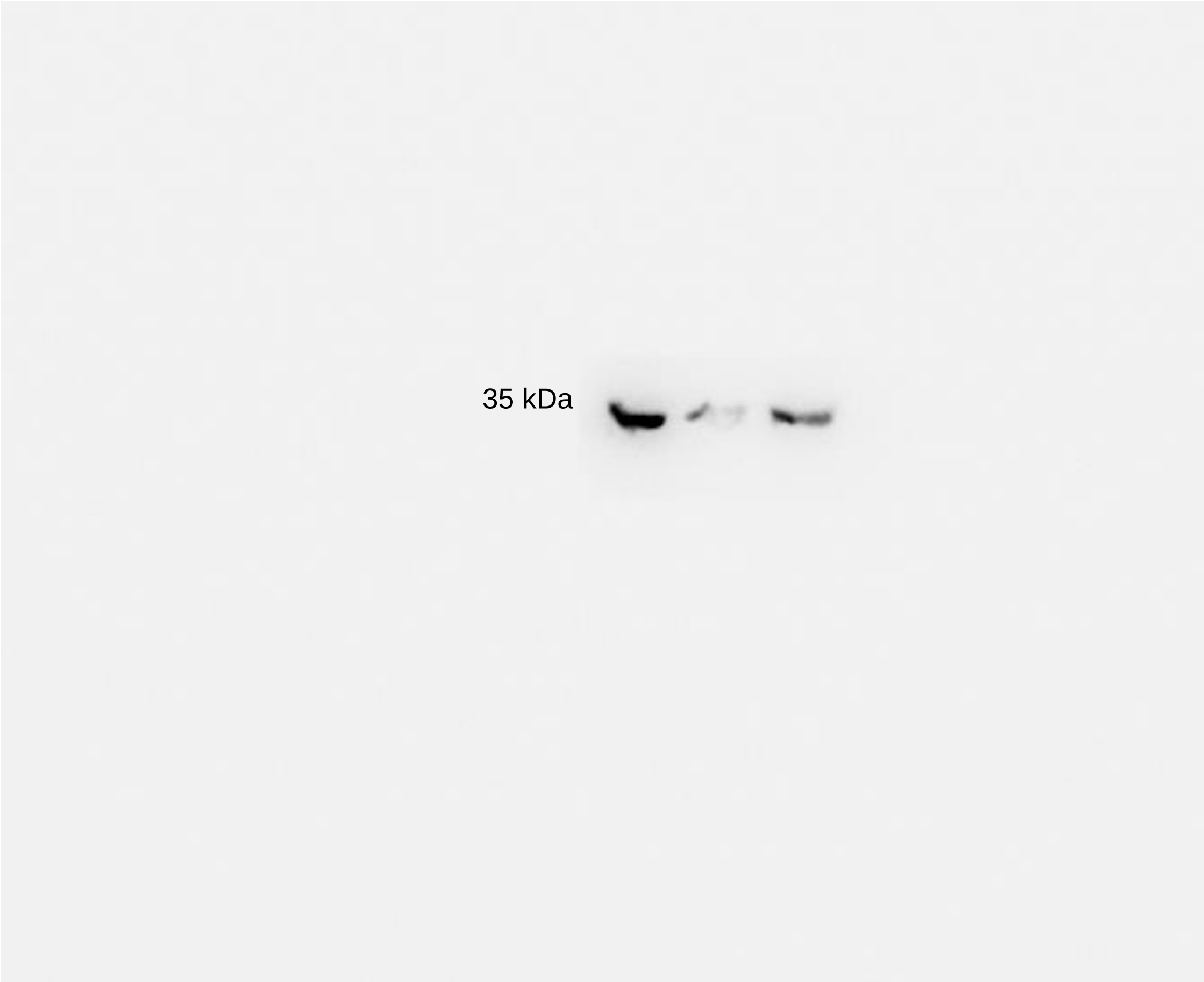


**Fig 6D**

**IB: MARCHF5**

Groups (SKOV3 cells): Input, IgG (IP), SLC25A46 (IP)

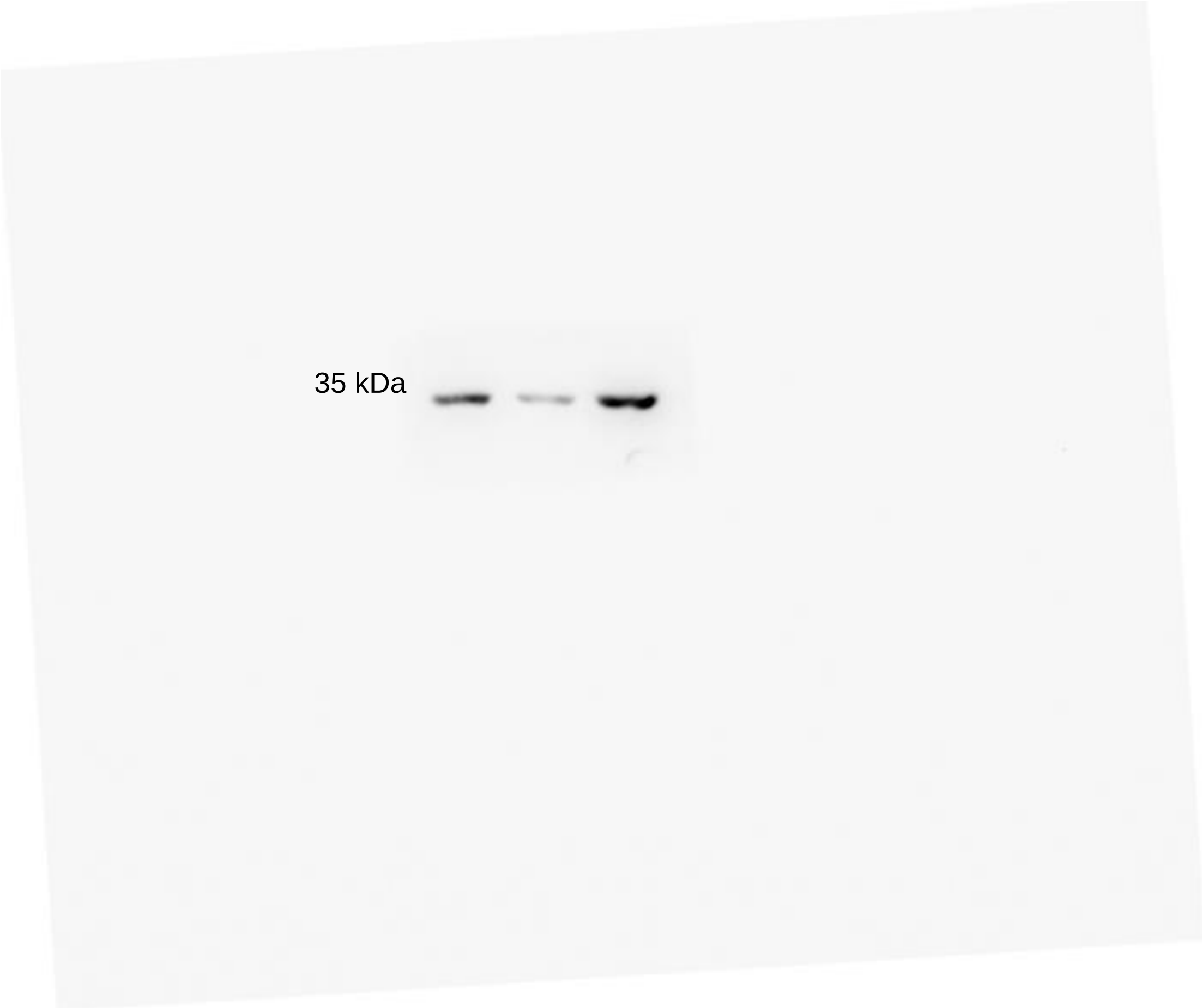


**Fig 6E**

**IB: MARCHF5**

Groups (ES2 cells): Input, IgG (IP), MARCHF5 (IP)

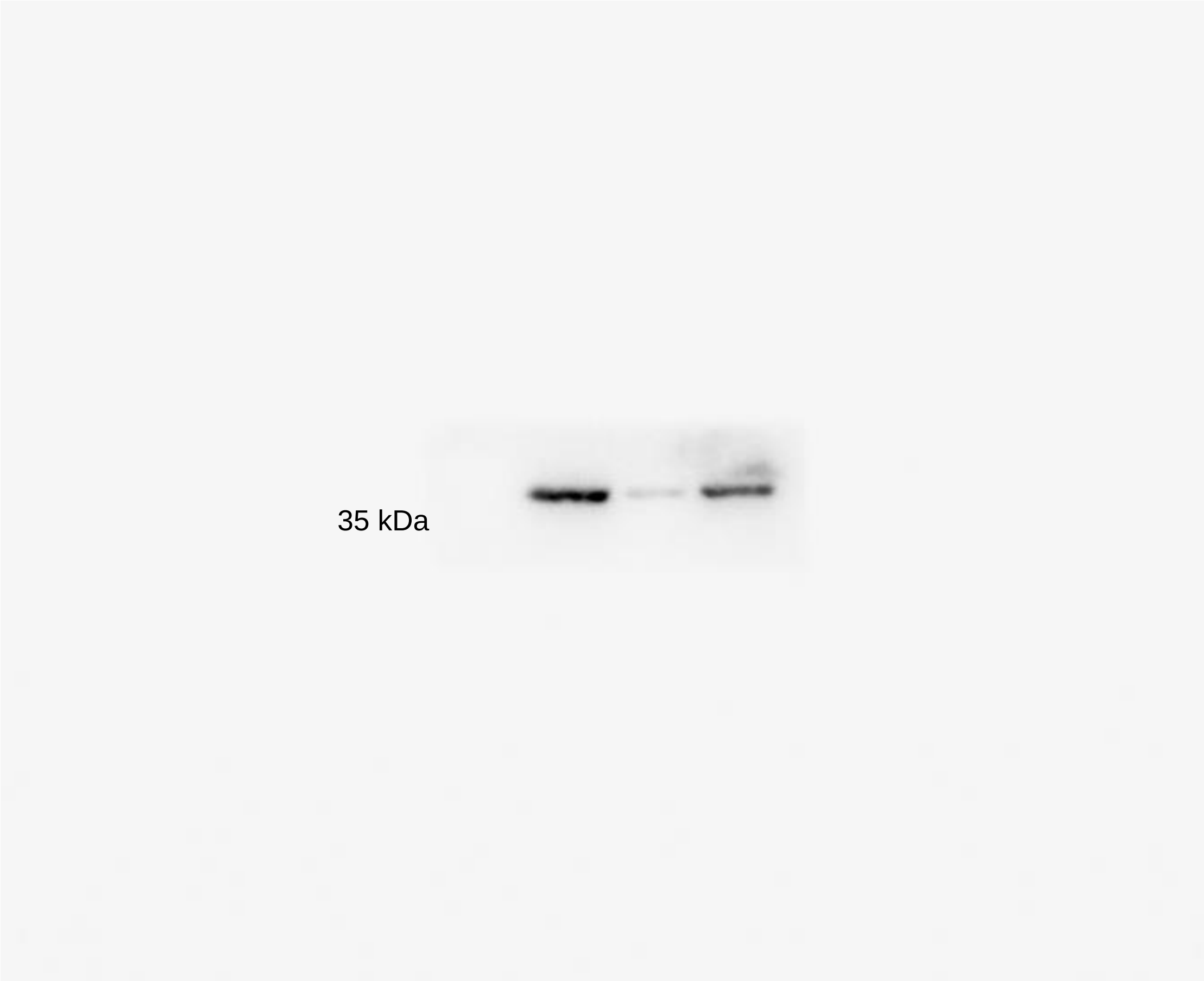


**Fig 6E**

**IB: SLC25A46**

Groups (ES2 cells): Input, IgG (IP), MARCHF5 (IP)

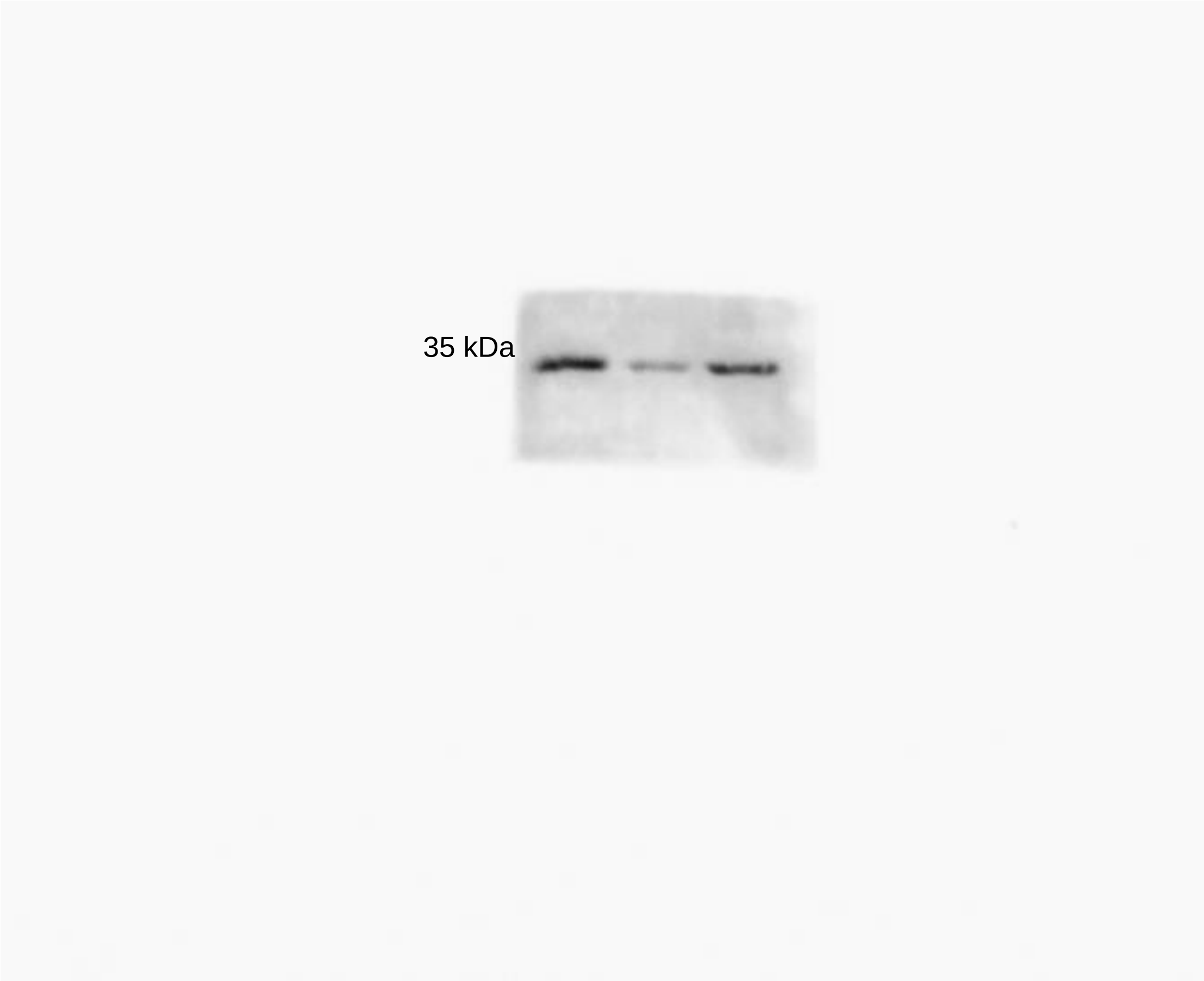


**Fig 6E**

**IB: CACT**

Groups (ES2 cells): Input, IgG (IP), MARCHF5 (IP)

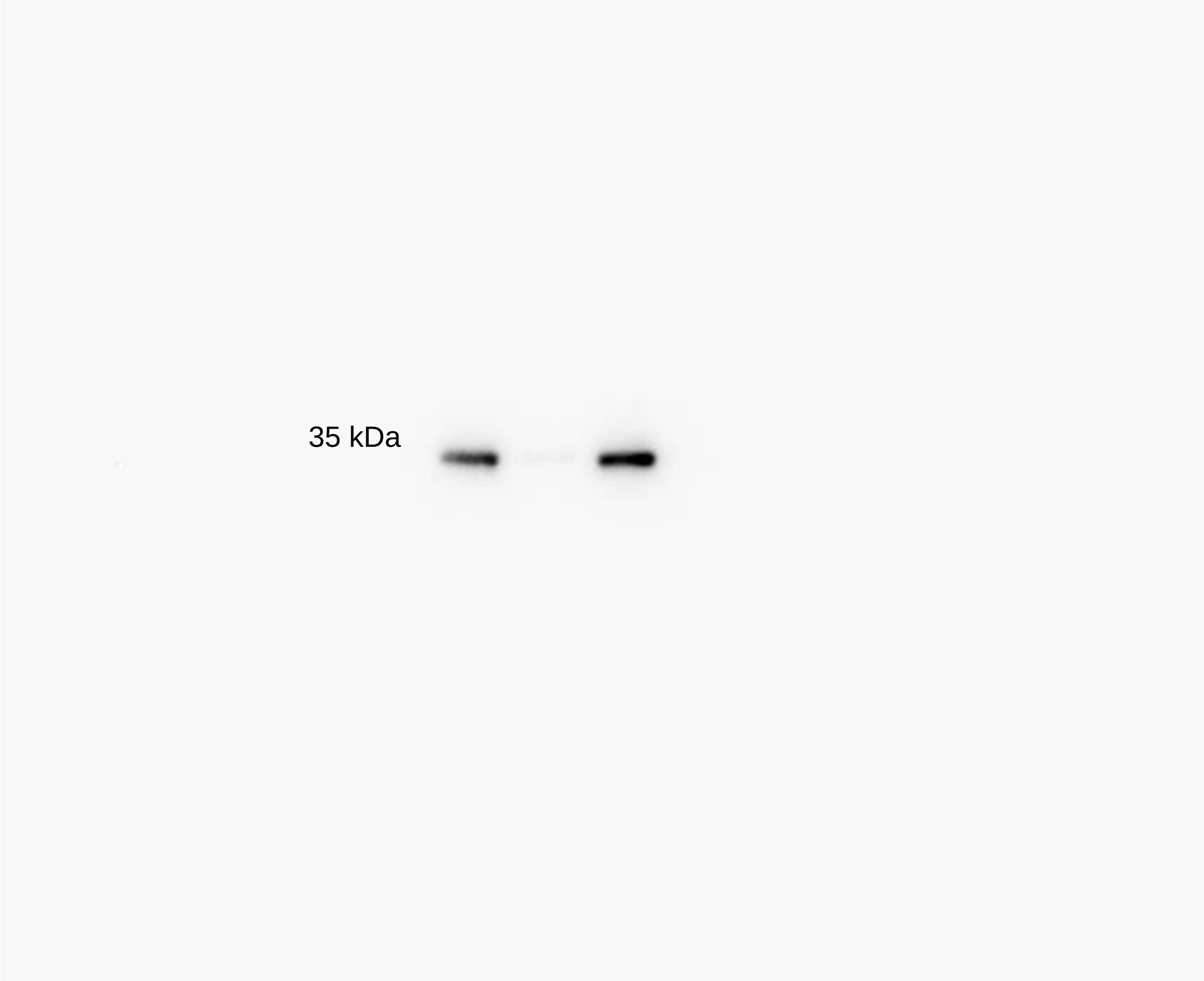


**Fig 6E**

**IB: MARCHF5**

Groups (SKOV3 cells): Input, IgG (IP), MARCHF5 (IP)

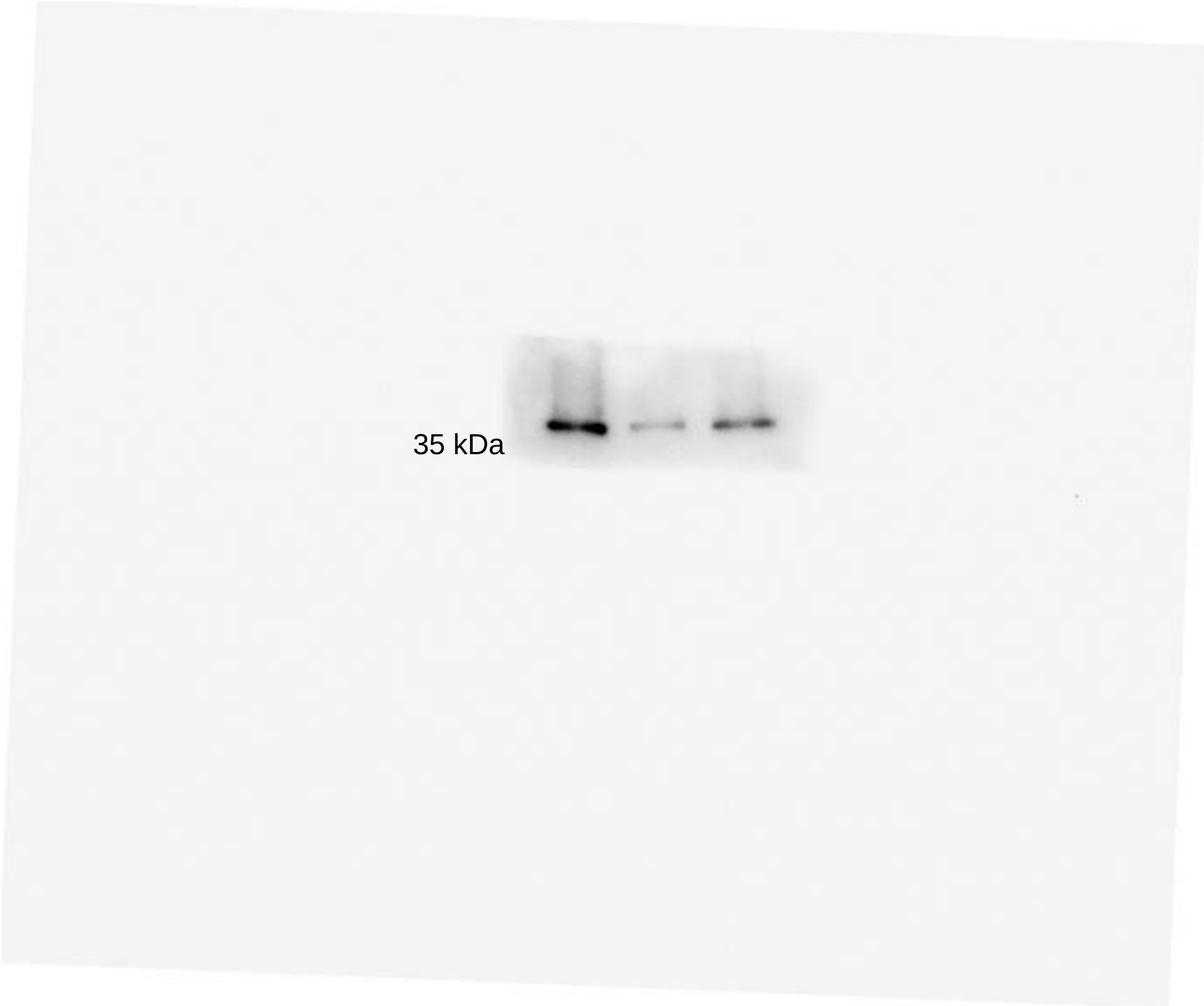


**Fig 6E**

**IB: SLC25A46**

Groups (SKOV3 cells): Input, IgG (IP), MARCHF5 (IP)

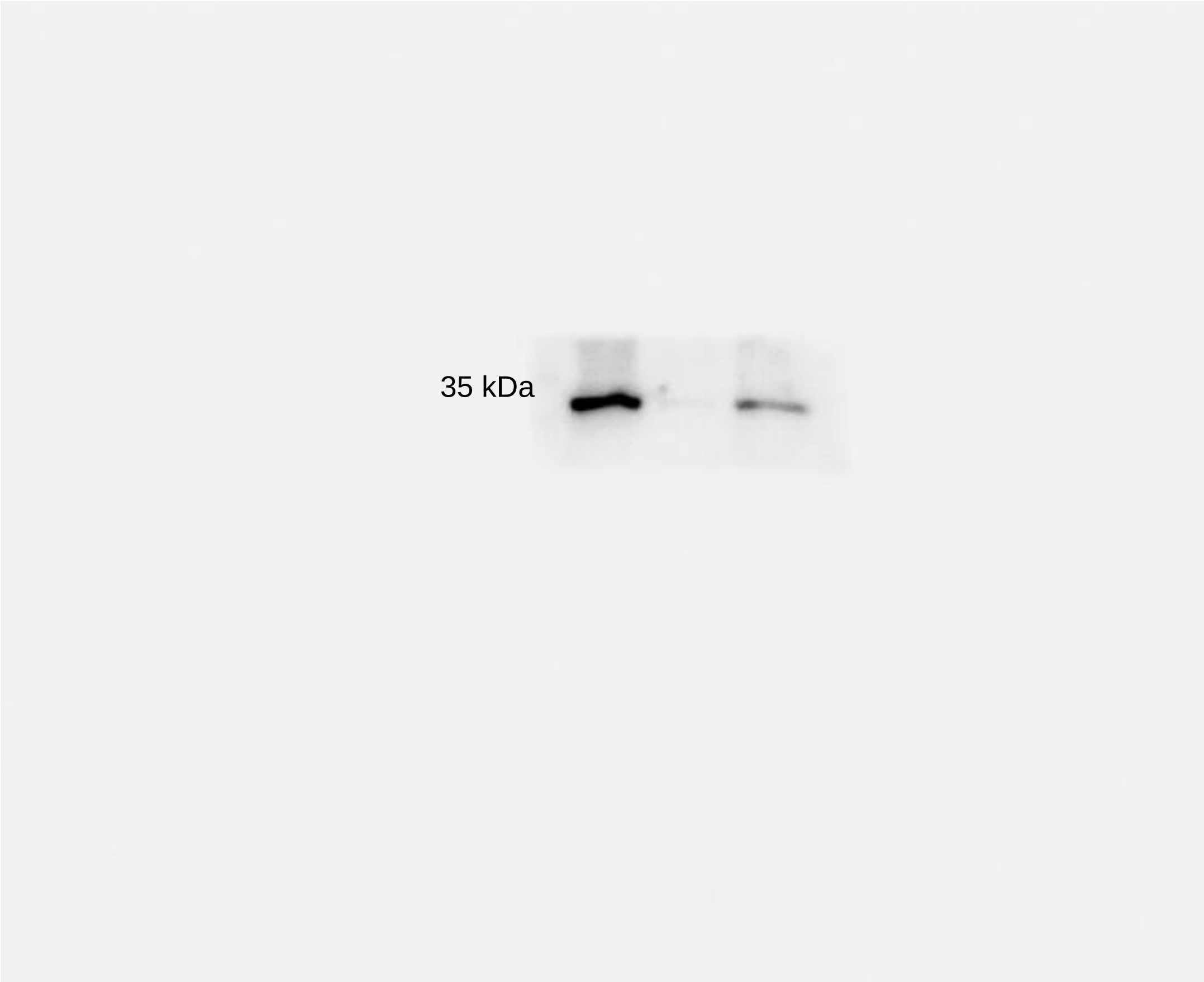


**Fig 6E**

**IB: CACT**

Groups (SKOV3 cells): Input, IgG (IP), MARCHF5 (IP)

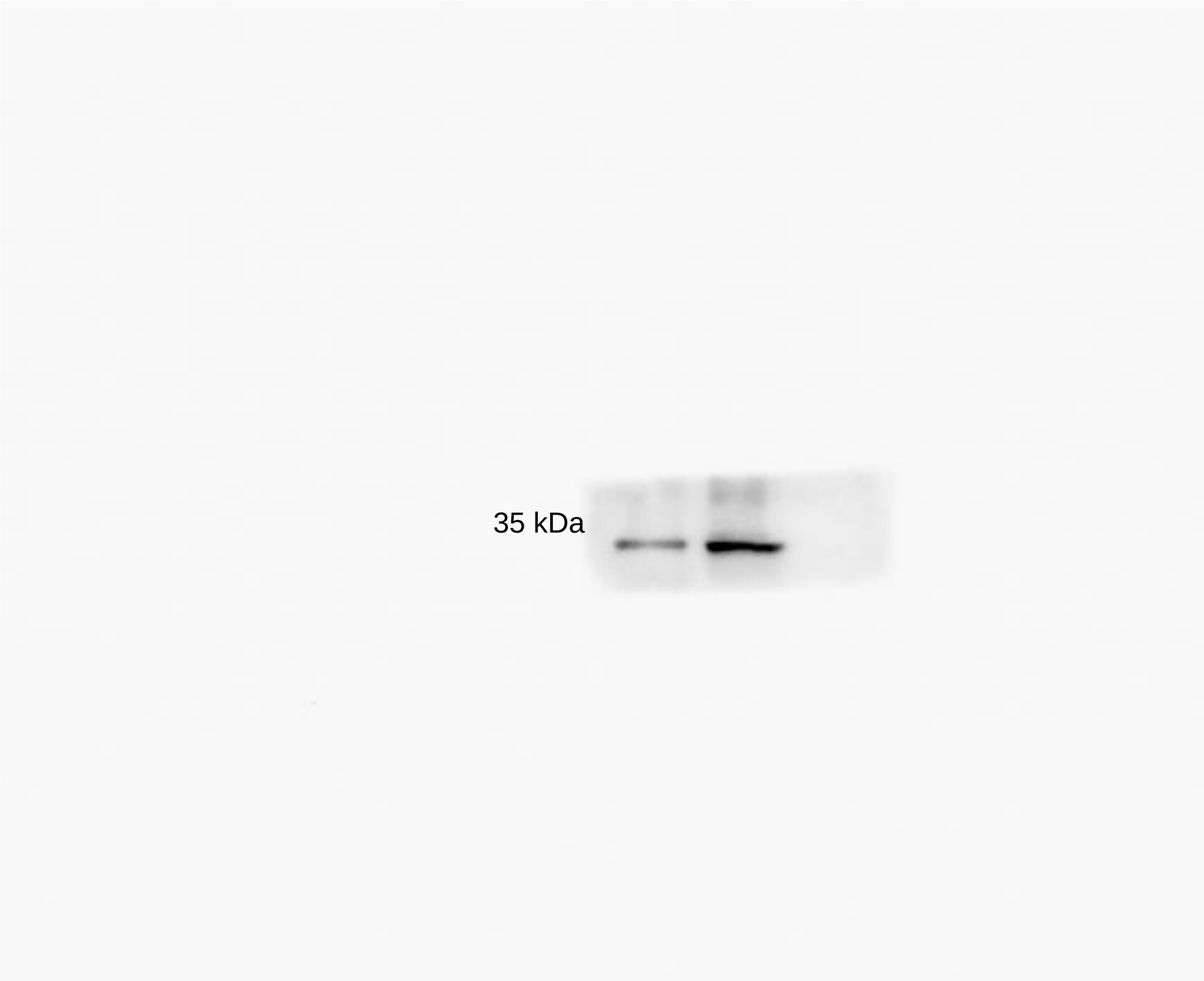


**Fig 6F**

**IB: CACT**

Groups (ES2 cells): shCtrl, shMARCHF5

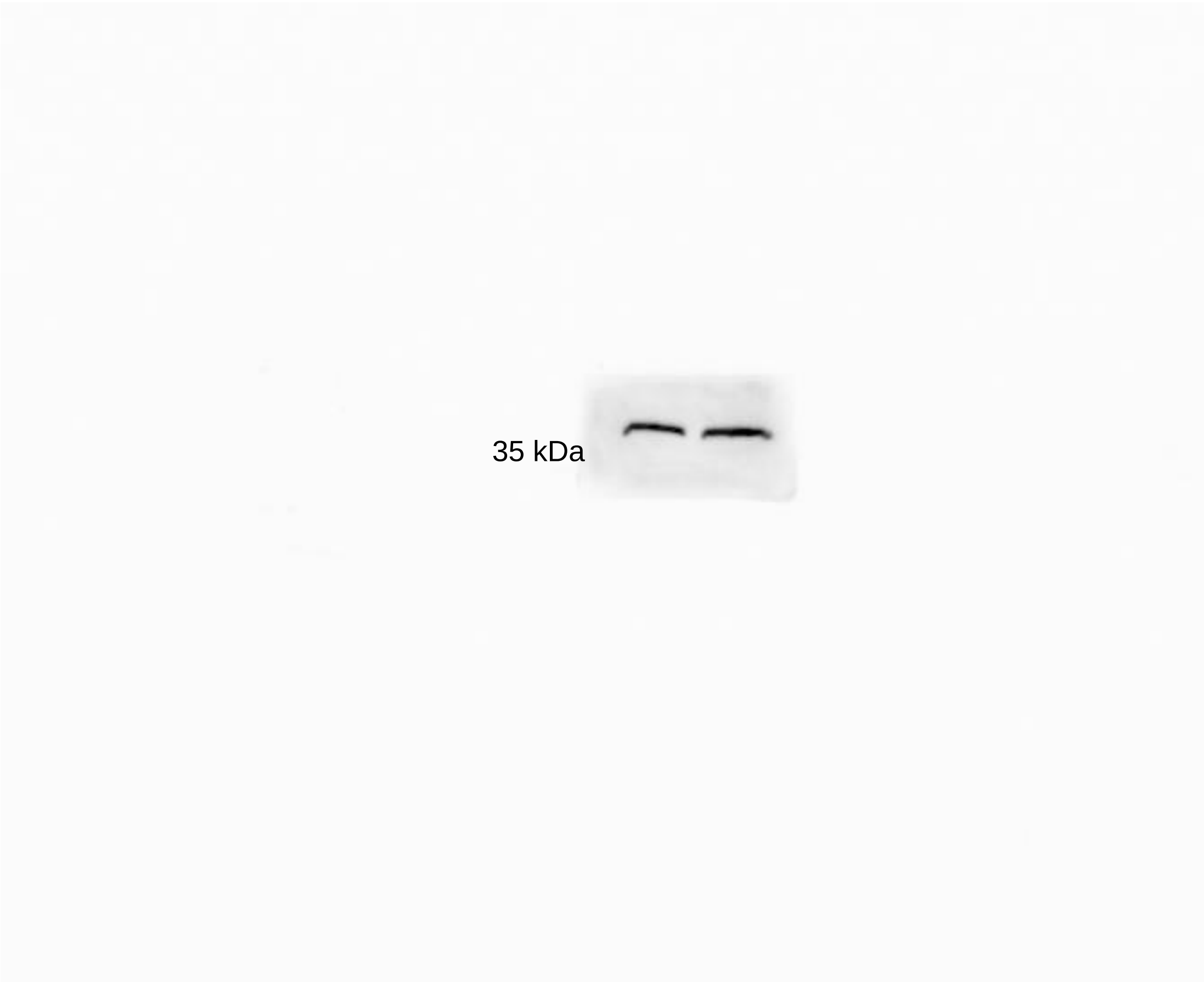


**Fig 6F**

**IB: β-actin**

Groups (ES2 cells): shCtrl, shMARCHF5

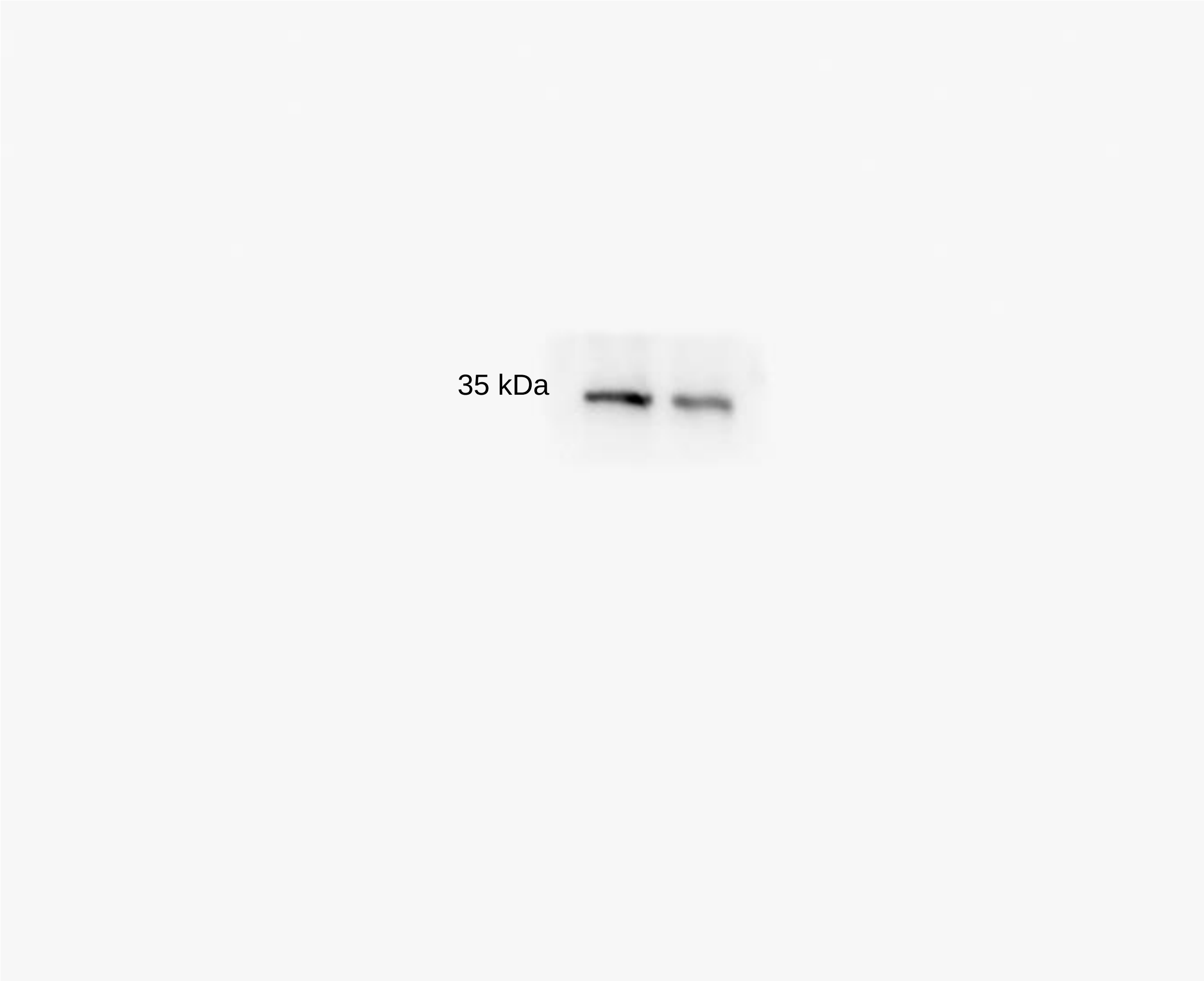


**Fig 6F**

**IB: CACT**

Groups (HEY cells): EV, MARCHF5

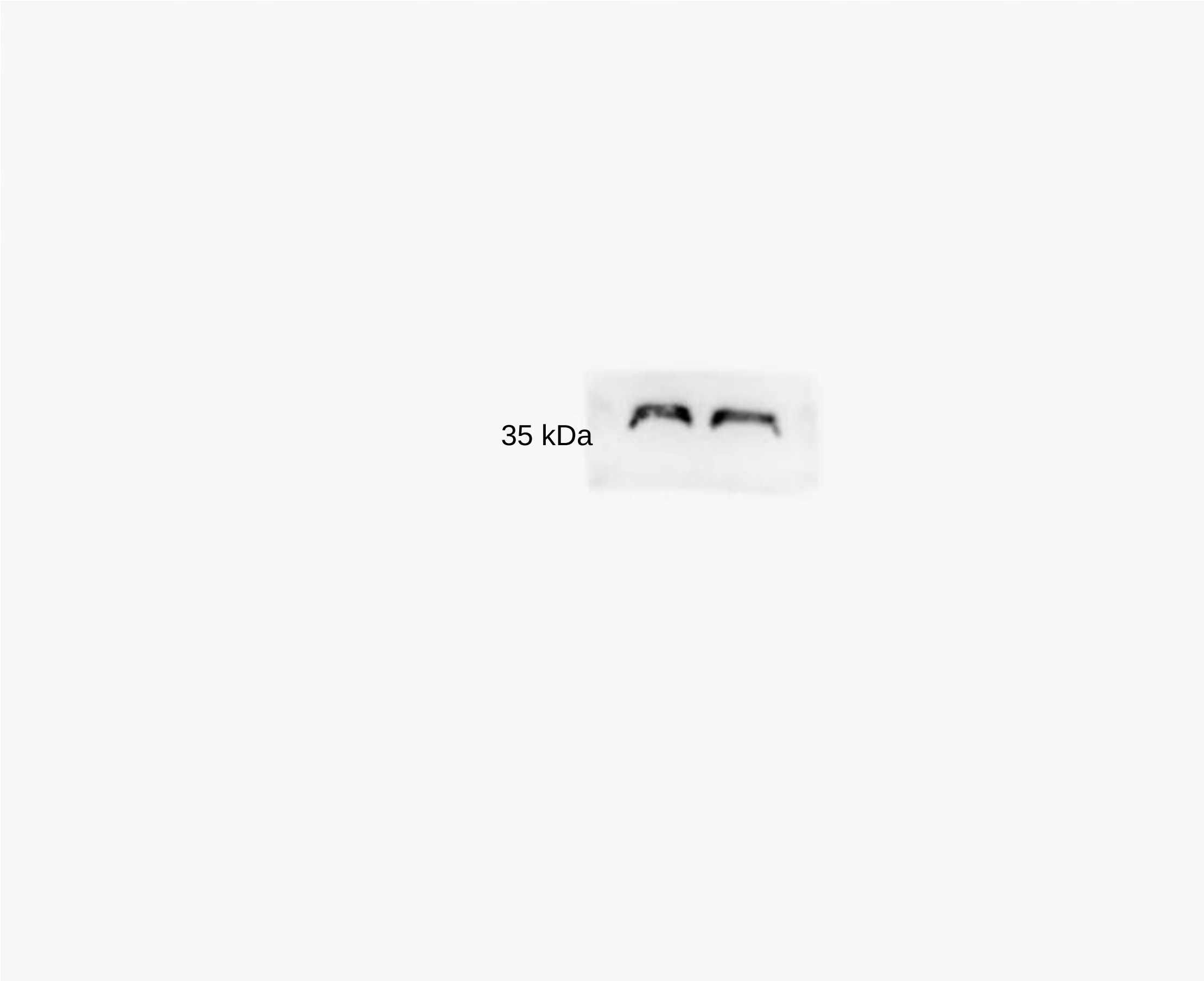


**Fig 6F**

**IB: β-actin**

Groups (HEY cells): EV, MARCHF5

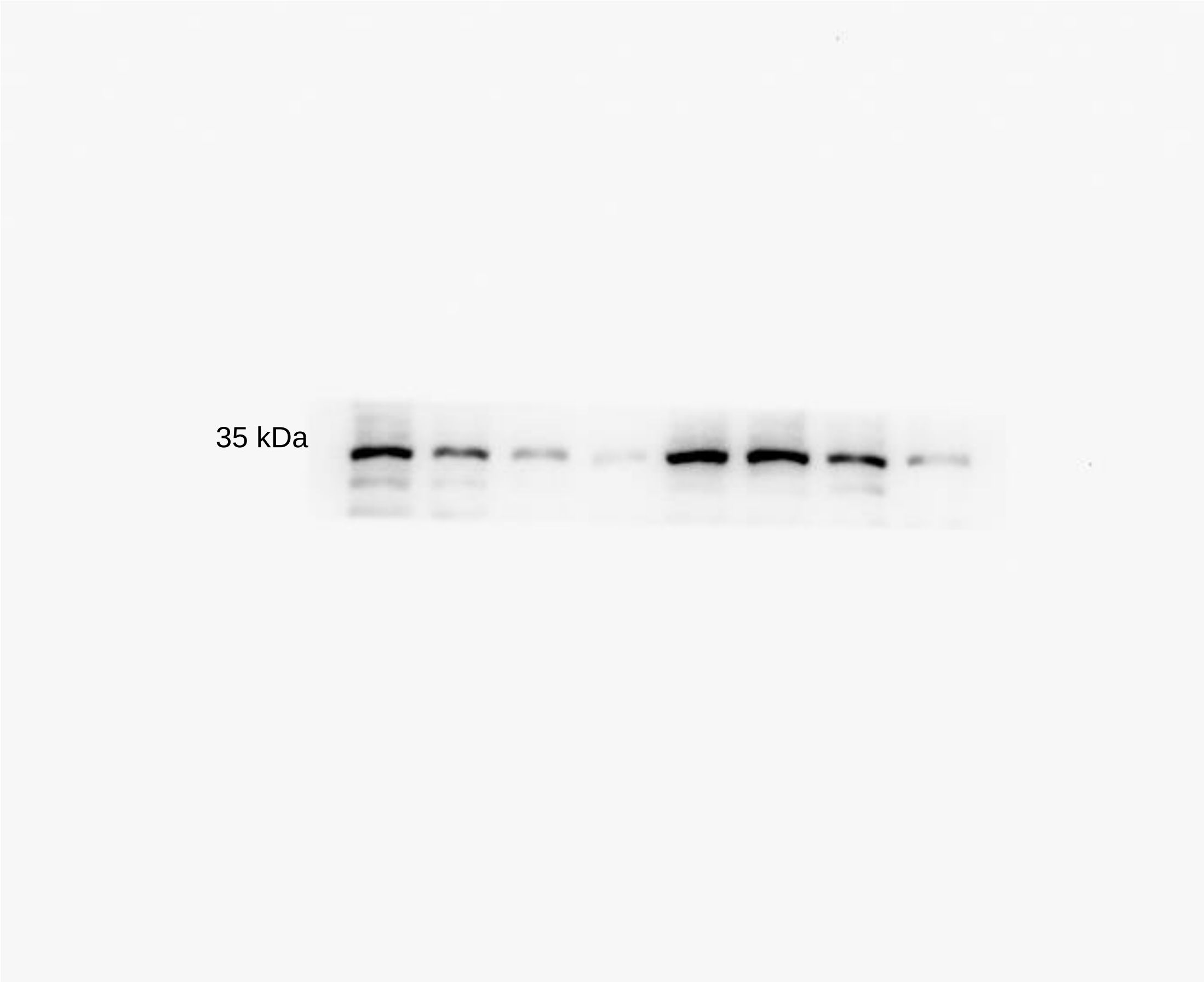


**Fig 6G**

**IB: CACT**

Groups (ES2 cells): shCtrl (CHX: 0h, 1h, 2h, 4h), shMARCHF5 (CHX: 0h, 1h, 2h, 4h)

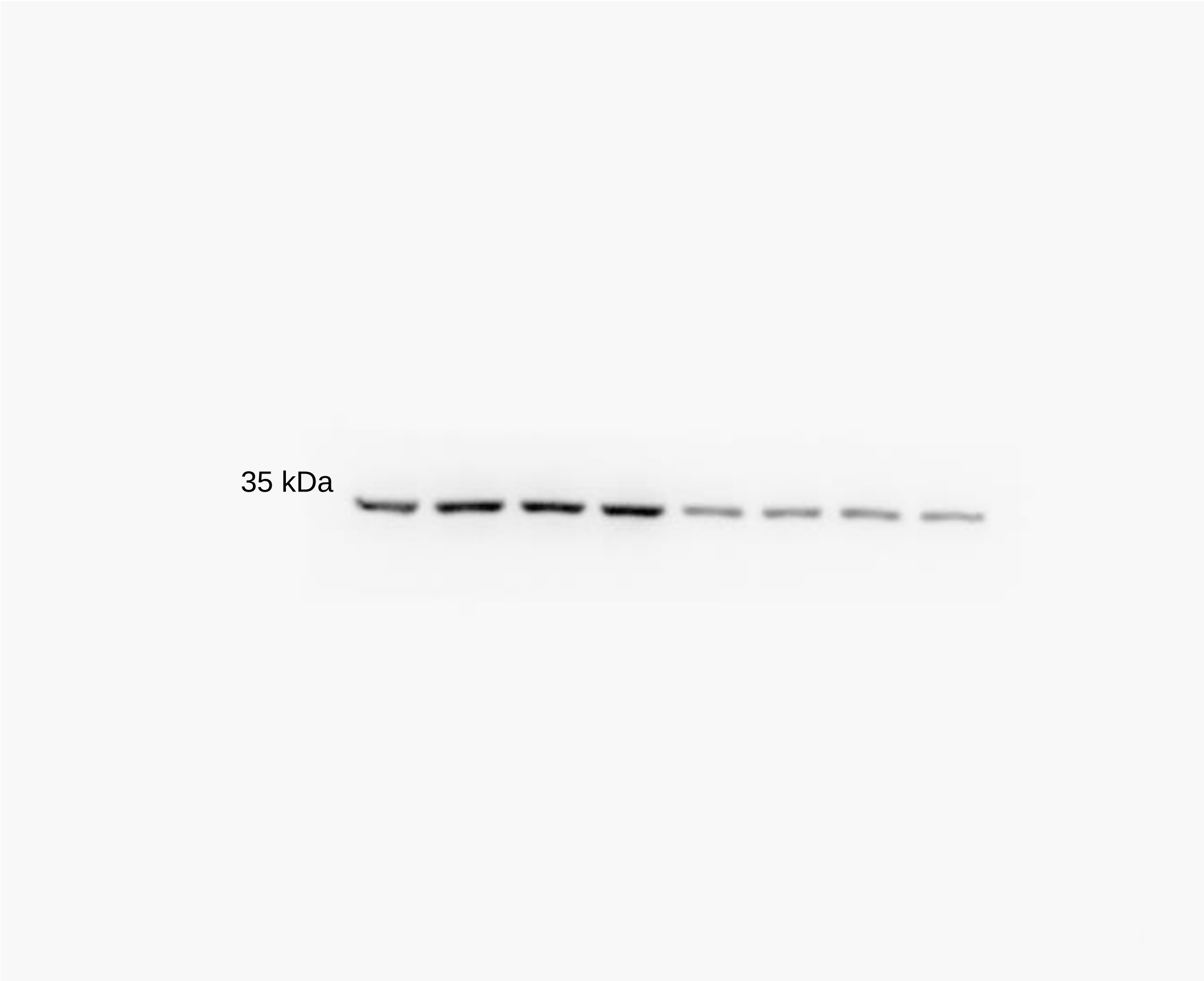


**Fig 6G**

**IB: MARCHF5**

Groups (ES2 cells): shCtrl (CHX: 0h, 1h, 2h, 4h), shMARCHF5 (CHX: 0h, 1h, 2h, 4h)

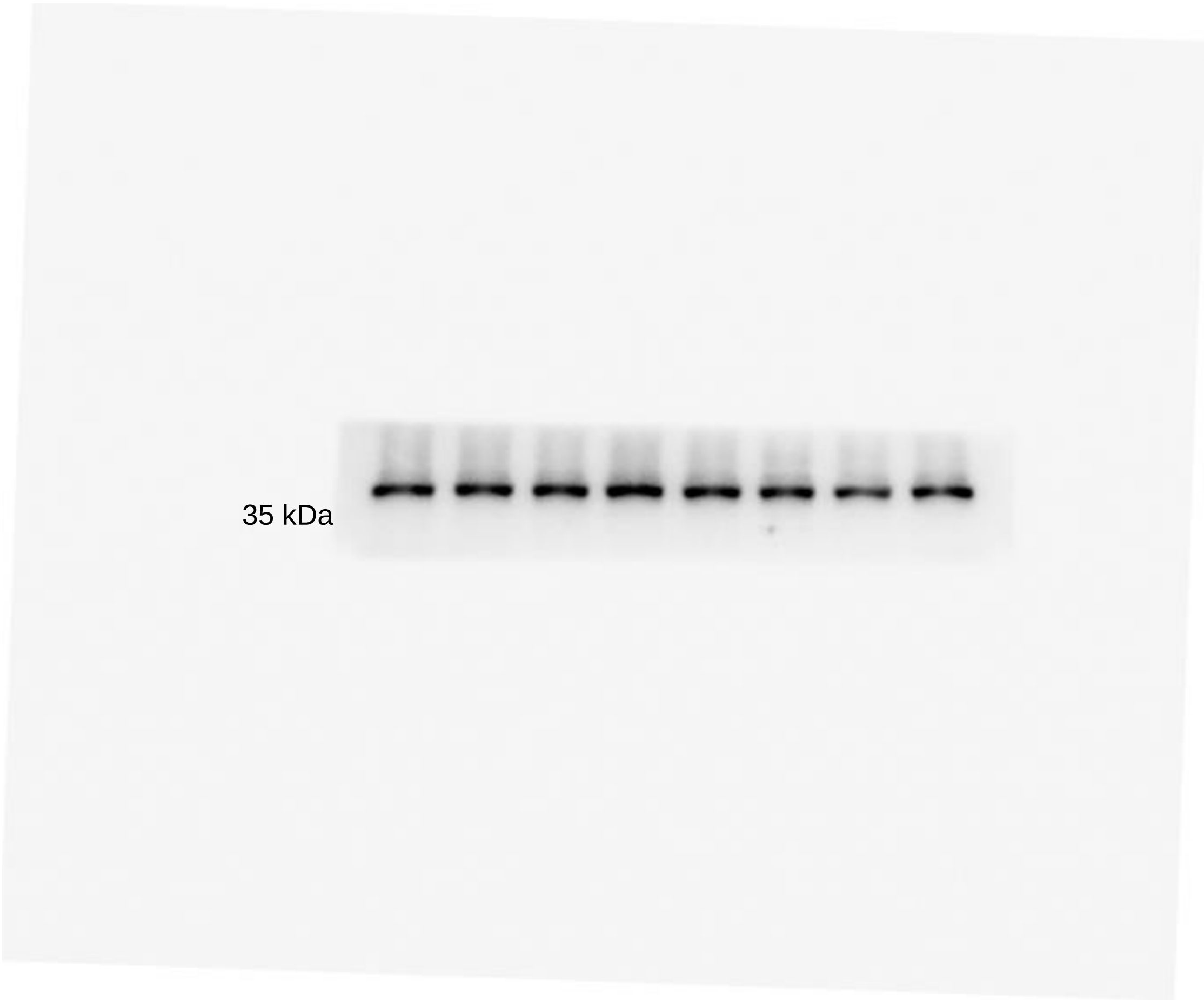


**Fig 6G**

**IB: β-actin**

Groups (ES2 cells): shCtrl (CHX: 0h, 1h, 2h, 4h), shMARCHF5 (CHX: 0h, 1h, 2h, 4h)

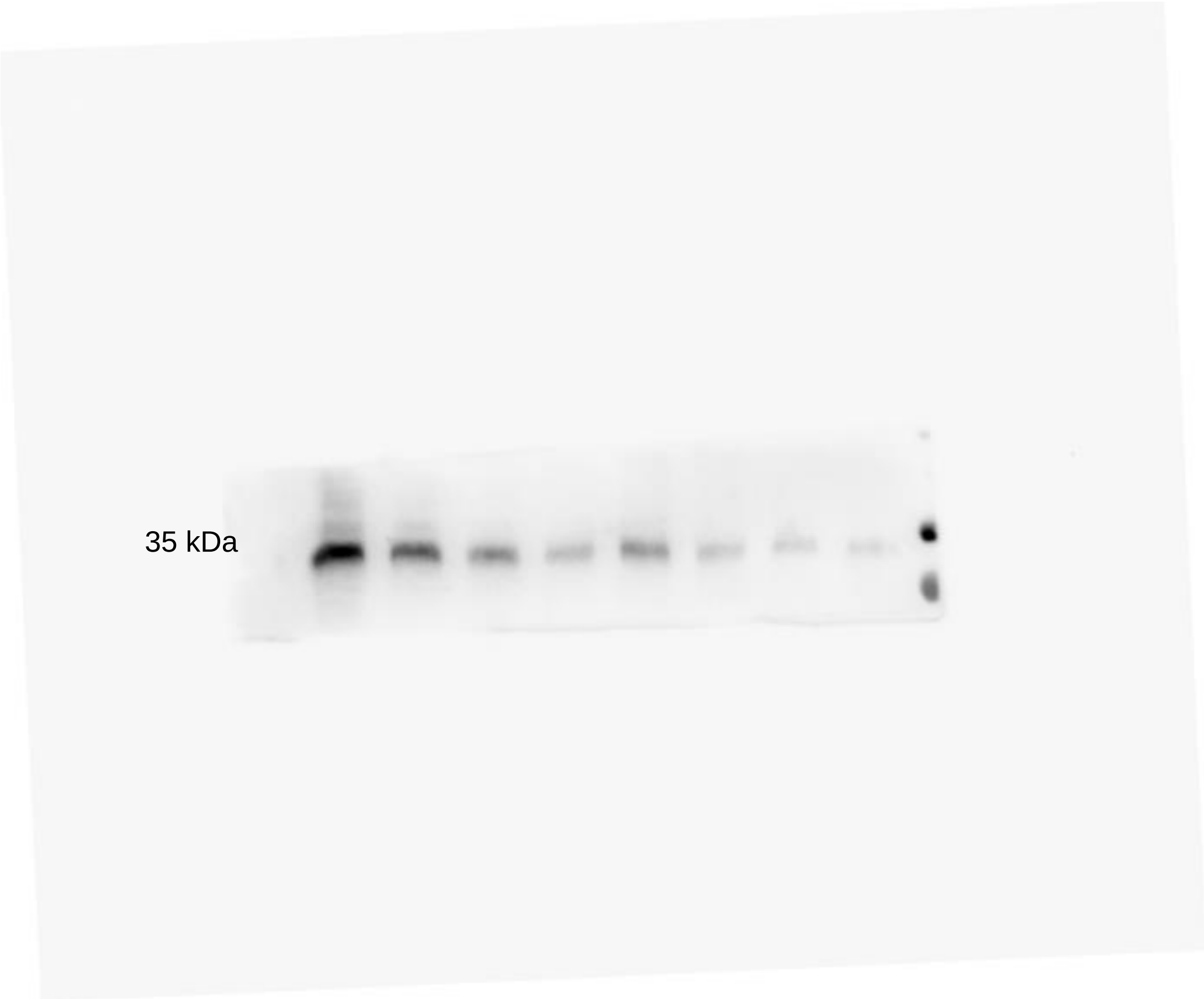


**Fig 6G**

**IB: CACT**

Groups (HEY cells): EV (CHX: 0h, 1h, 2h, 4h), MARCHF5 (CHX: 0h, 1h, 2h, 4h)

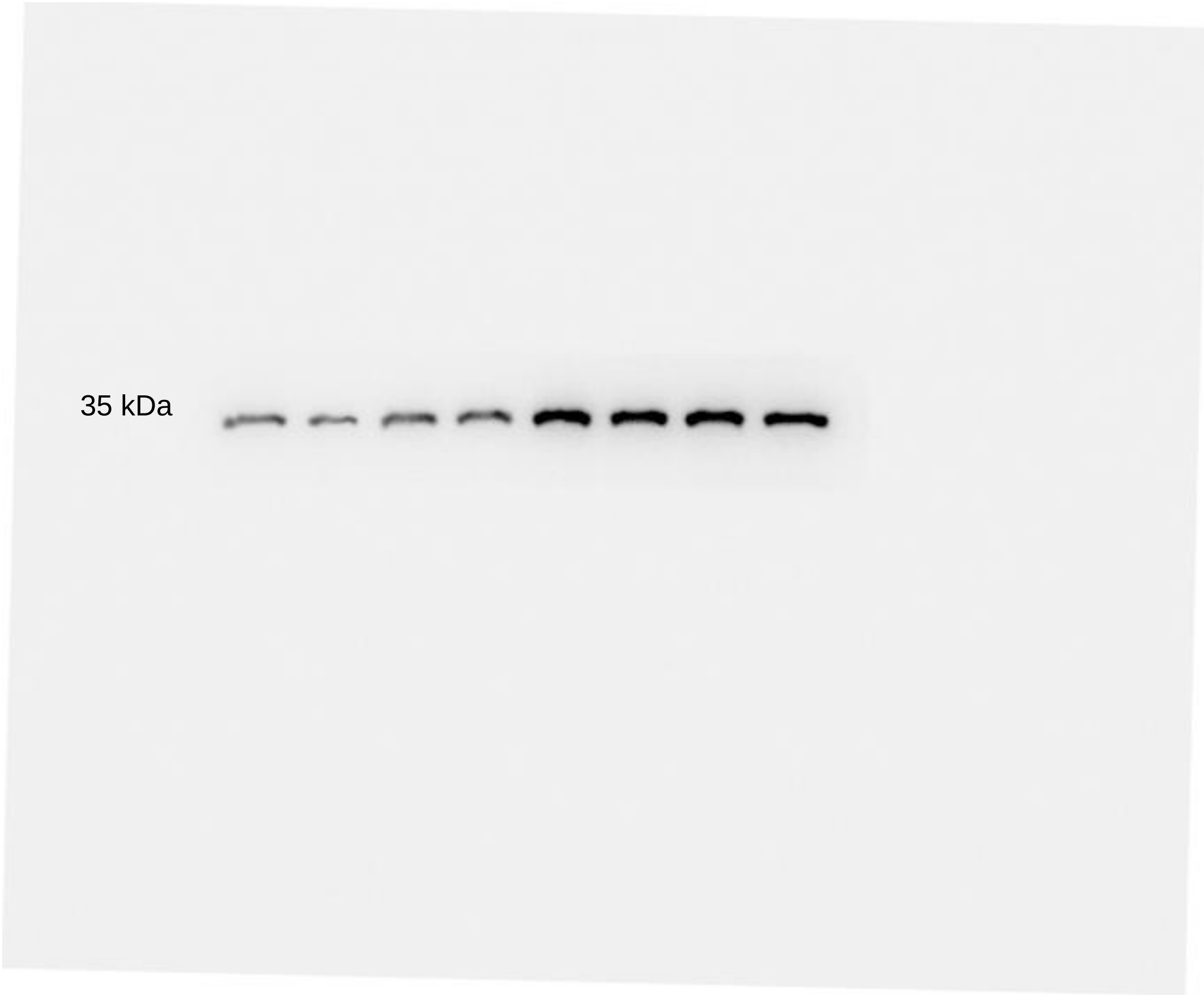


**Fig 6G**

**IB: MARCHF5**

Groups (HEY cells): EV (CHX: 0h, 1h, 2h, 4h), MARCHF5 (CHX: 0h, 1h, 2h, 4h)

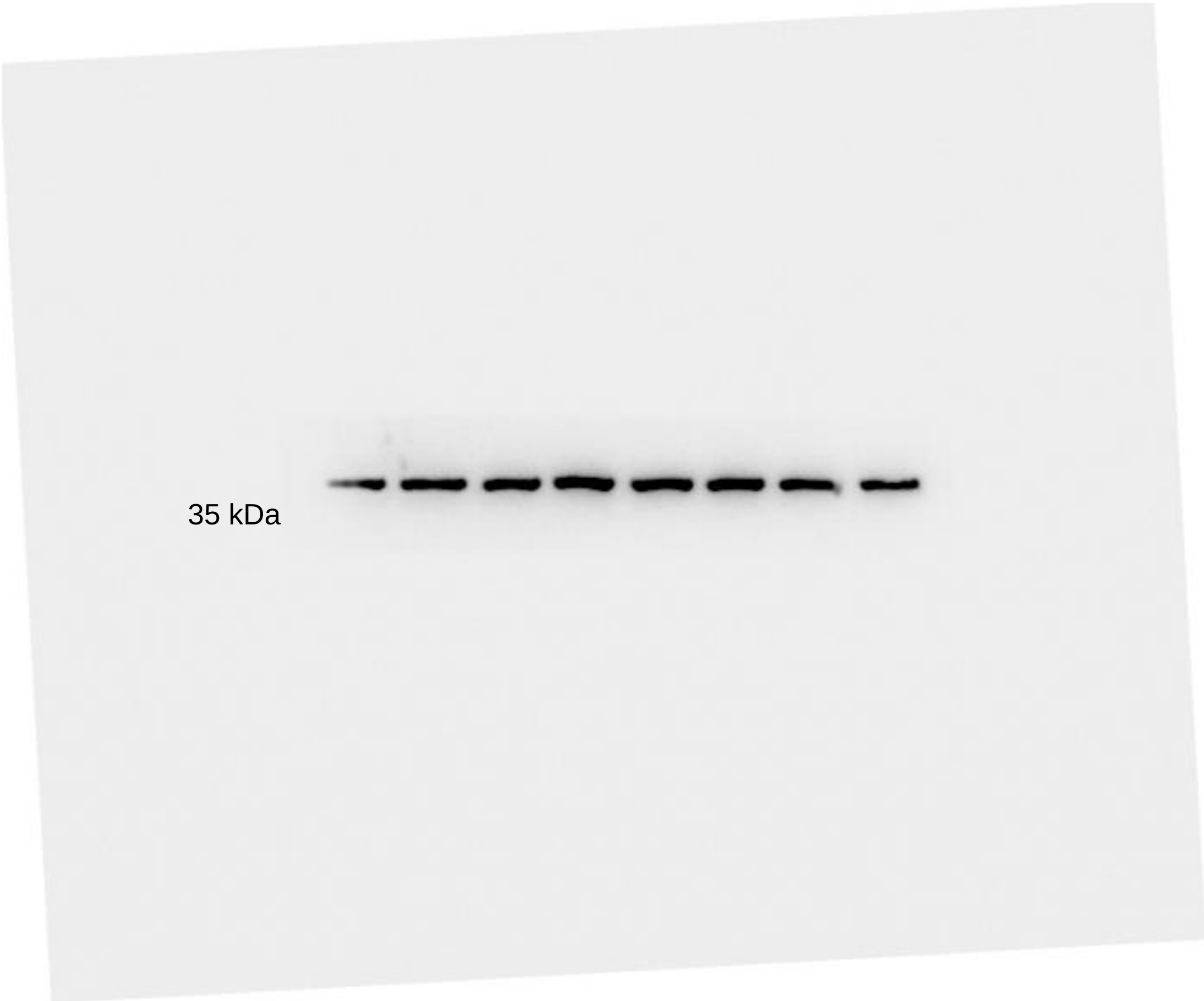


**Fig 6G**

**IB: β-actin**

Groups (HEY cells): EV (CHX: 0h, 1h, 2h, 4h), MARCHF5 (CHX: 0h, 1h, 2h, 4h)

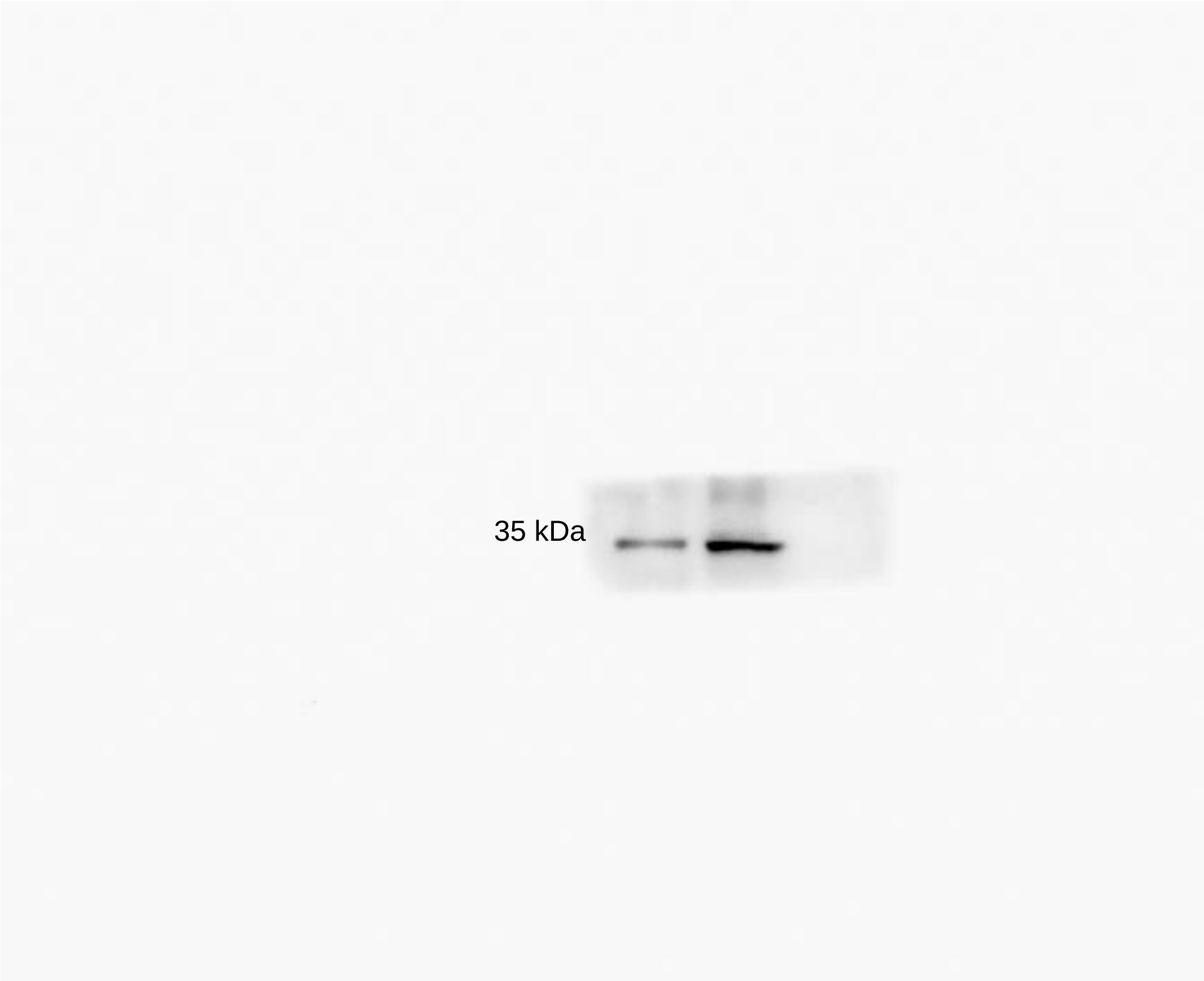


**Fig 6H**

**IB: CACT**

Groups (ES2 cells): shCtrl, shMARCH5

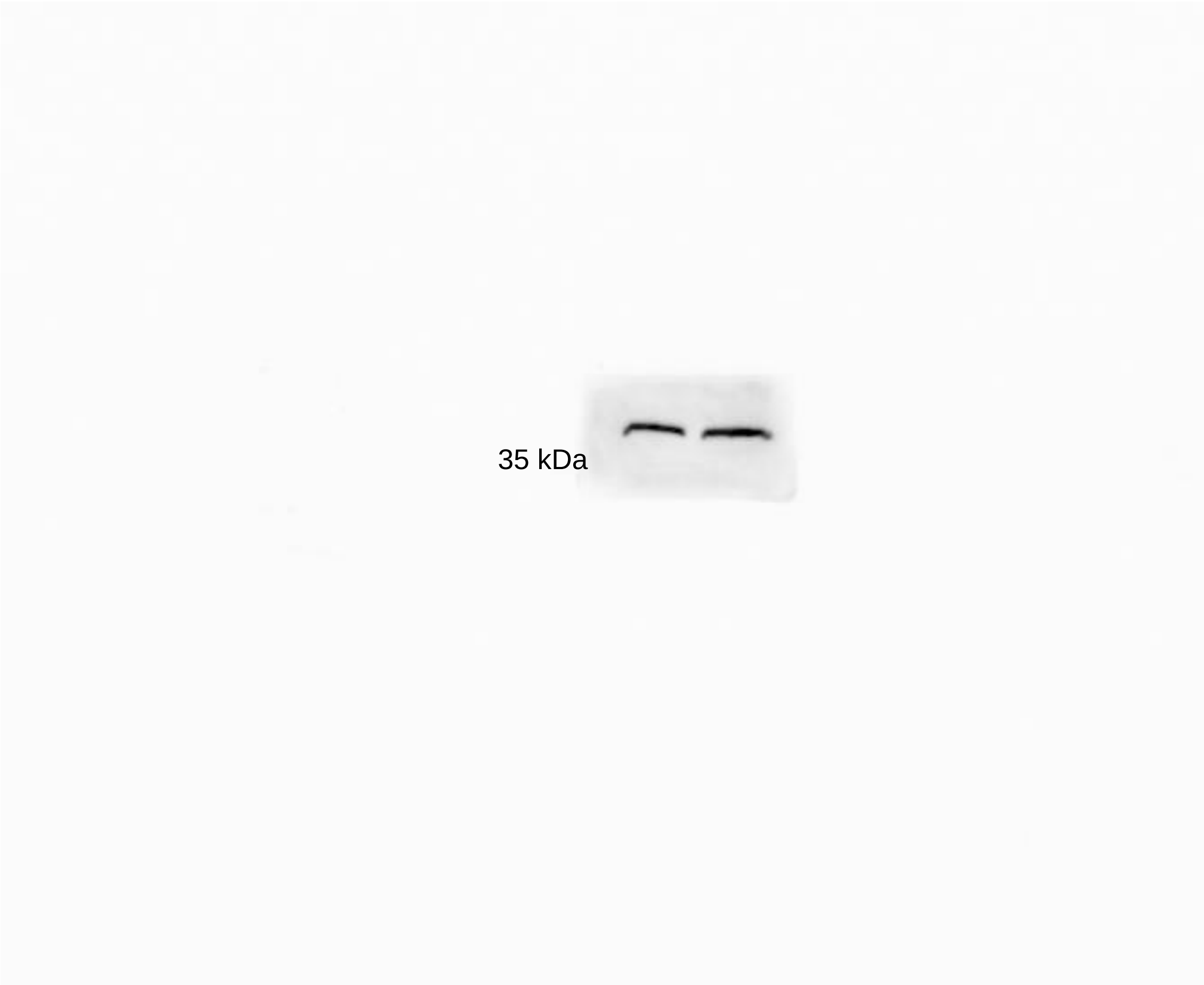


**Fig 6H**

**IB: β-actin**

Groups (ES2 cells): shCtrl, shMARCH5

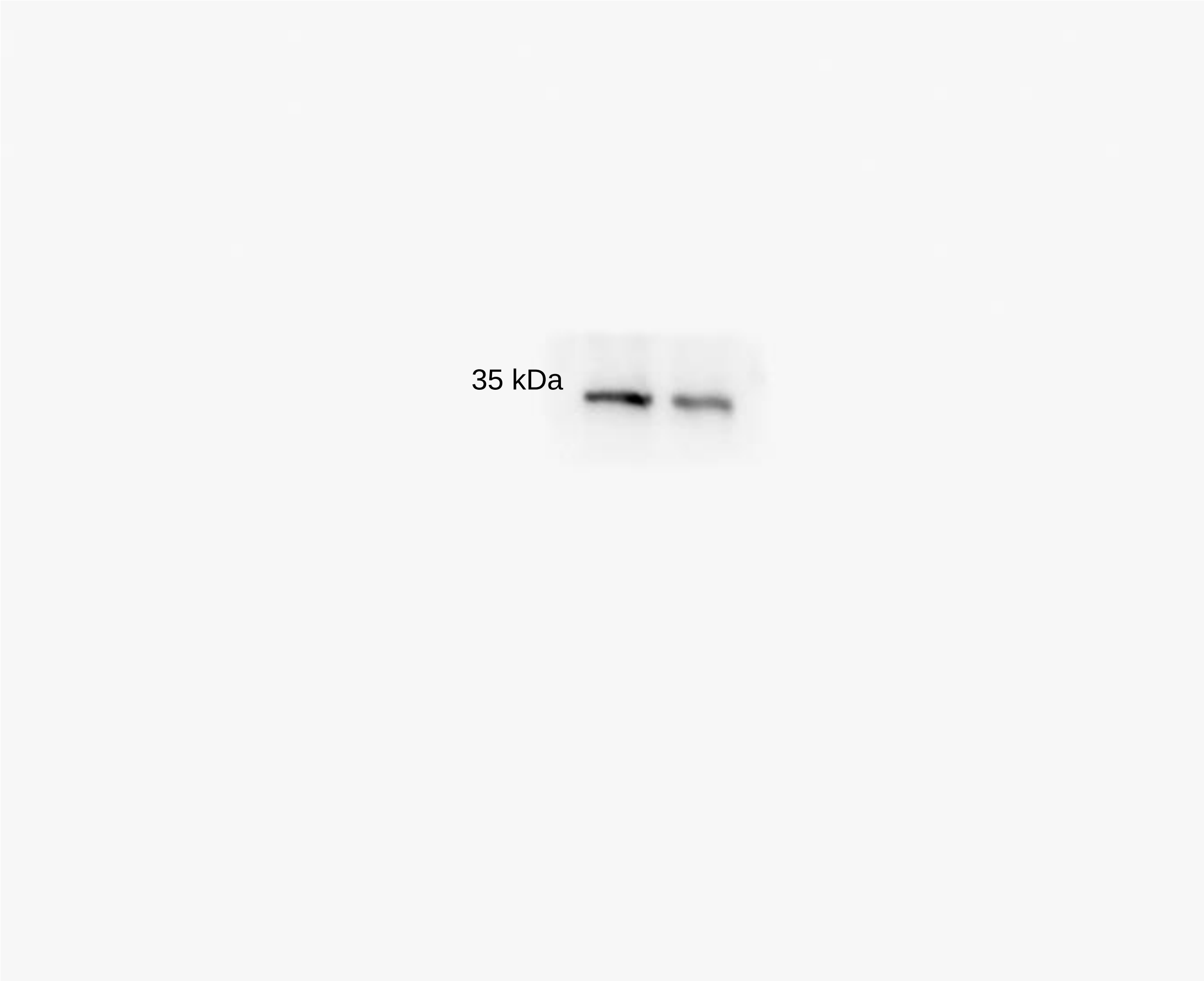


**Fig 6H**

**IB: CACT**

Groups (HEY cells): shCtrl, shMARCH5

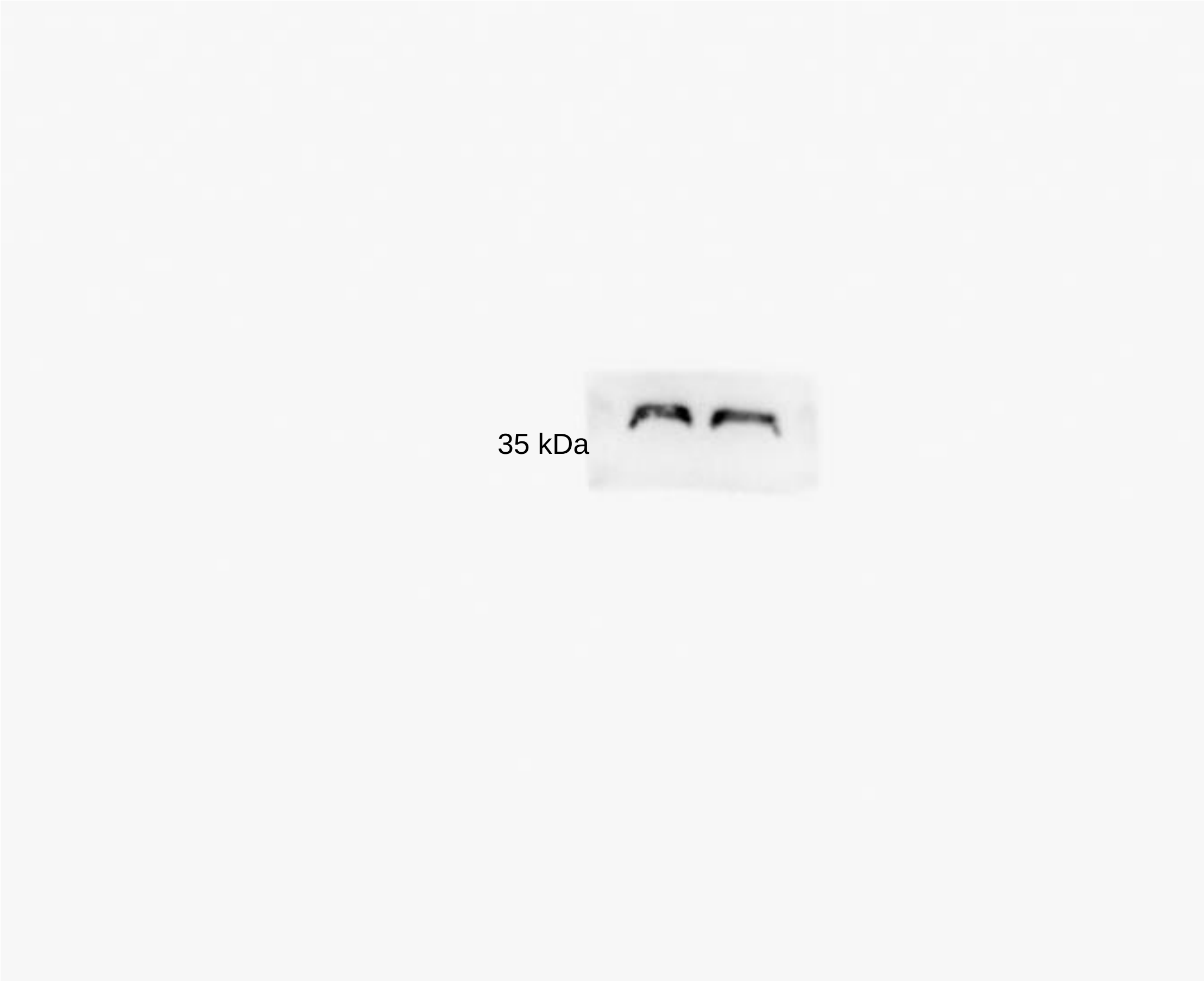


**Fig 6H**

**IB: β-actin**

Groups (HEY cells): shCtrl, shMARCH5

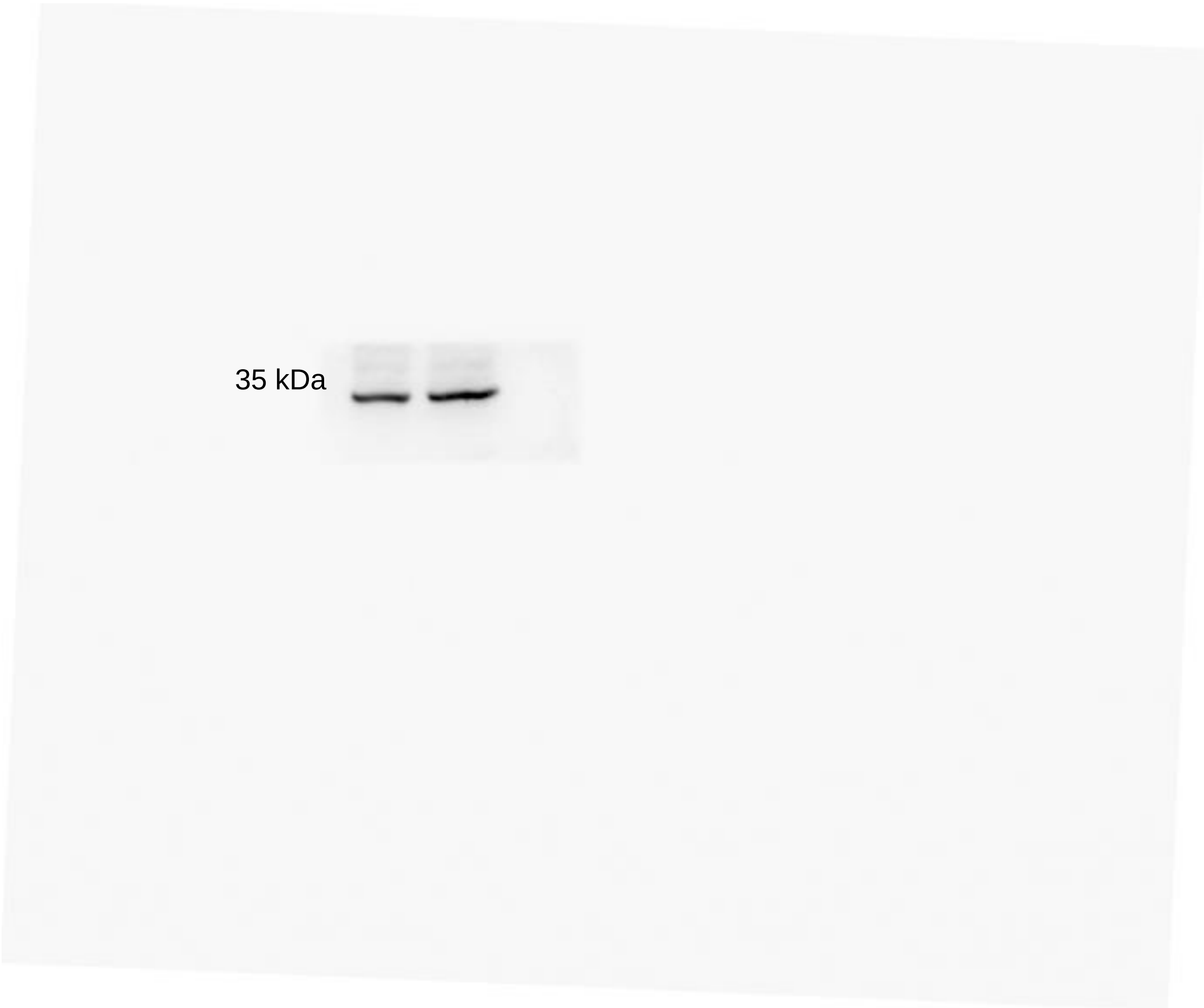


**Fig 6I**

**IB: MARCHF5**

Groups (ES2 cells): shCtrl, shSLC25A46

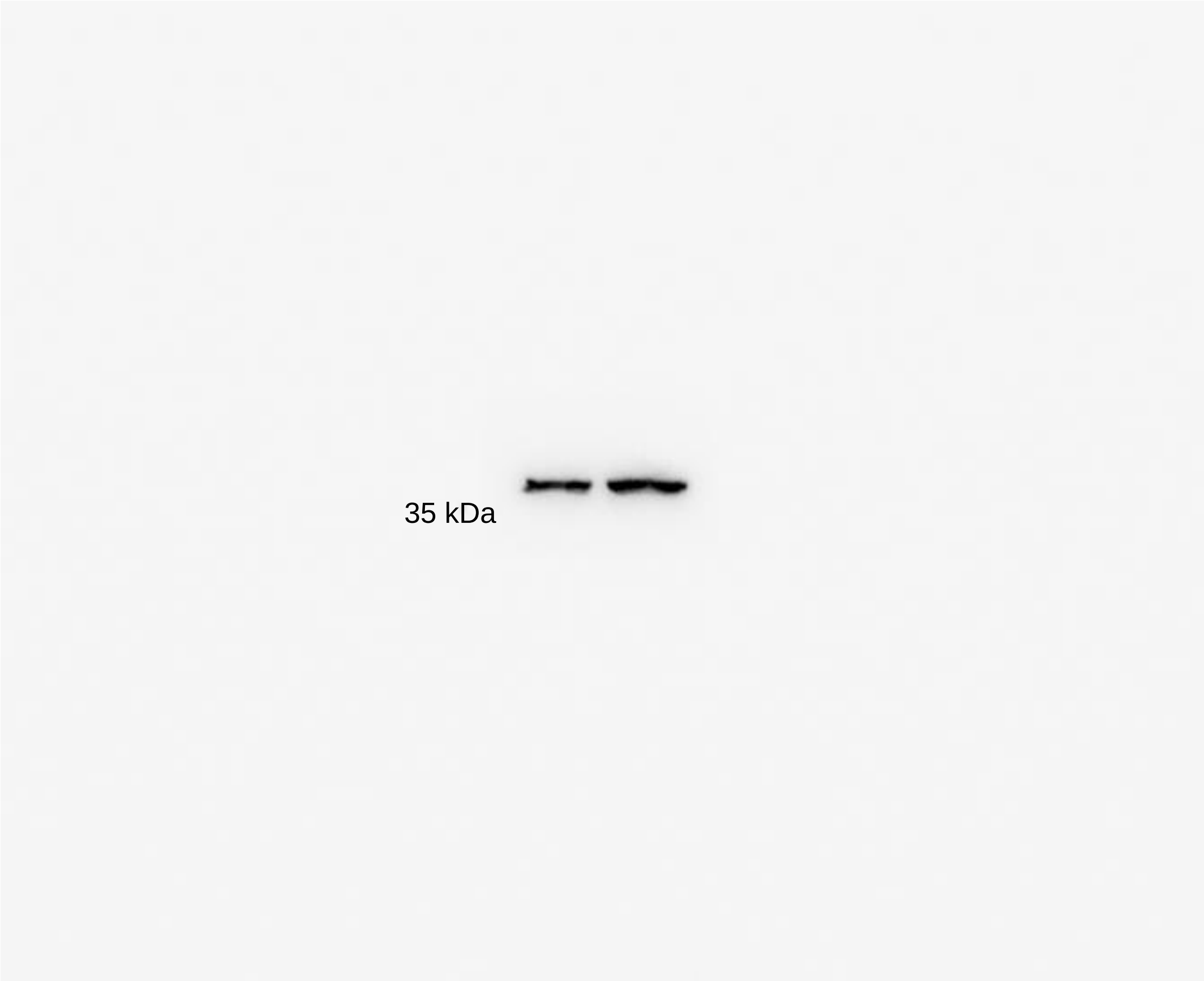


**Fig 6I**

**IB: β-actin**

Groups (ES2 cells): shCtrl, shSLC25A46

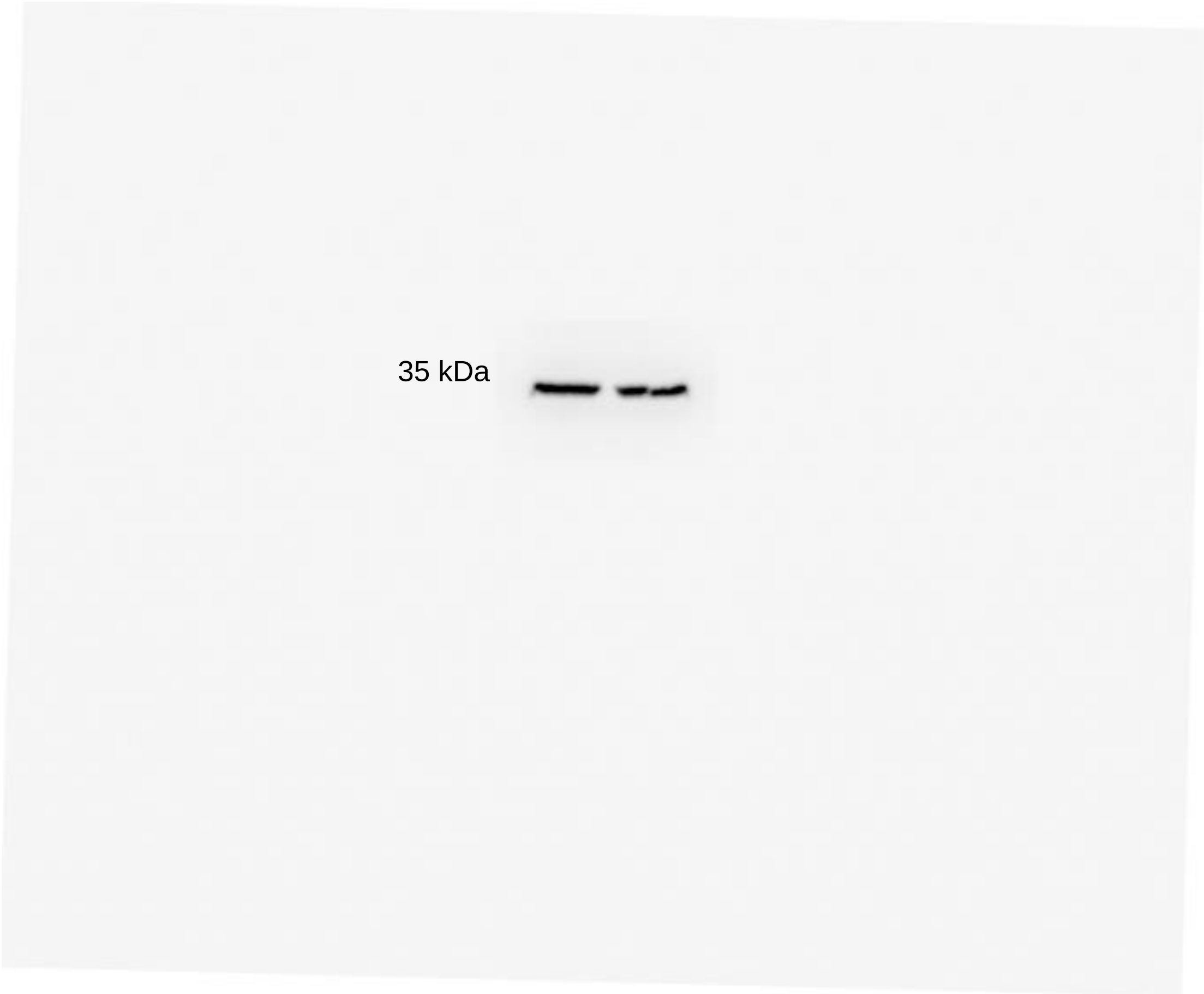


**Fig 6I**

**IB: MARCHF5**

Groups (HEY cells): EV, SLC25A46

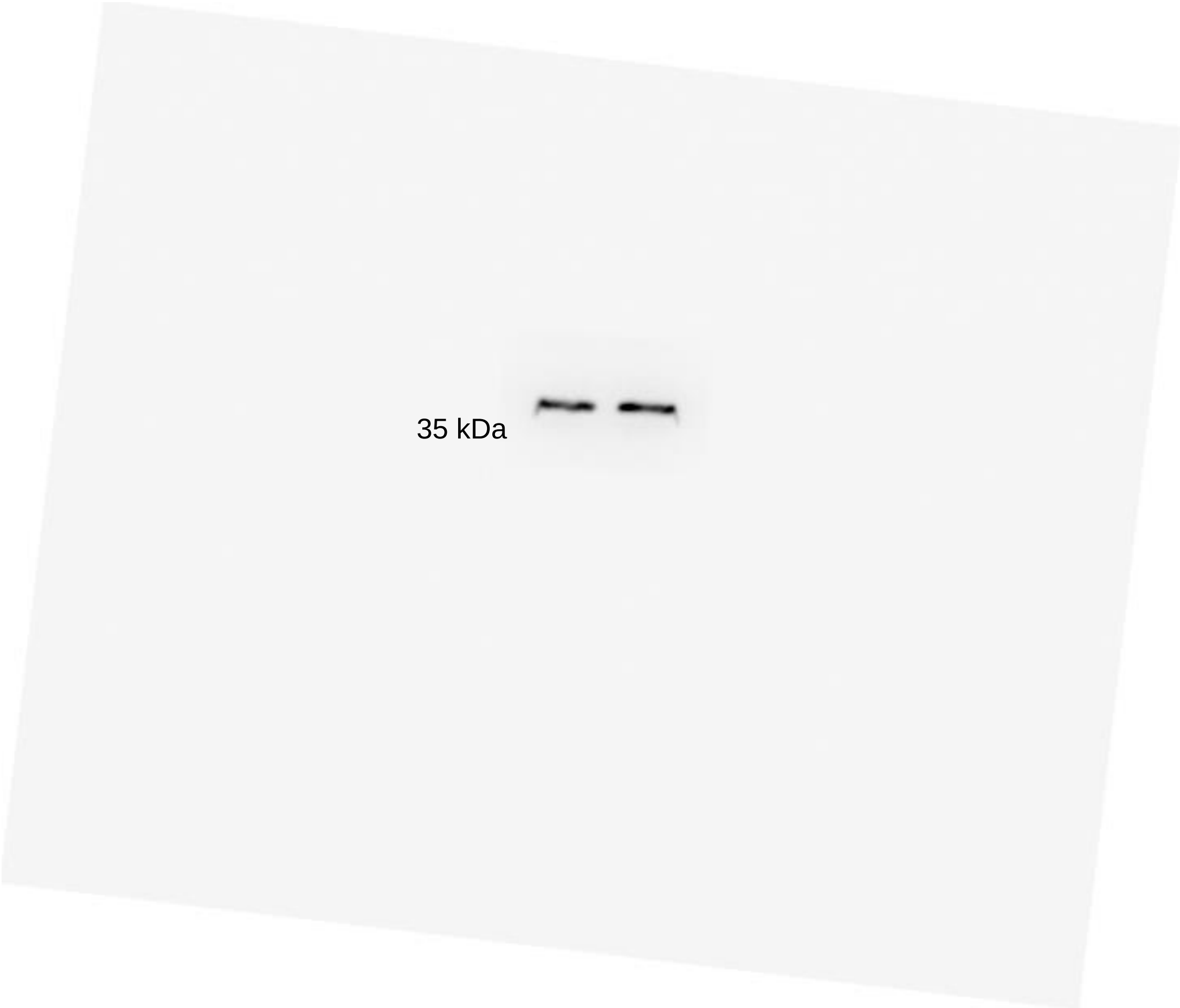


**Fig 6I**

**IB: β-actin**

Groups (HEY cells): EV, SLC25A46

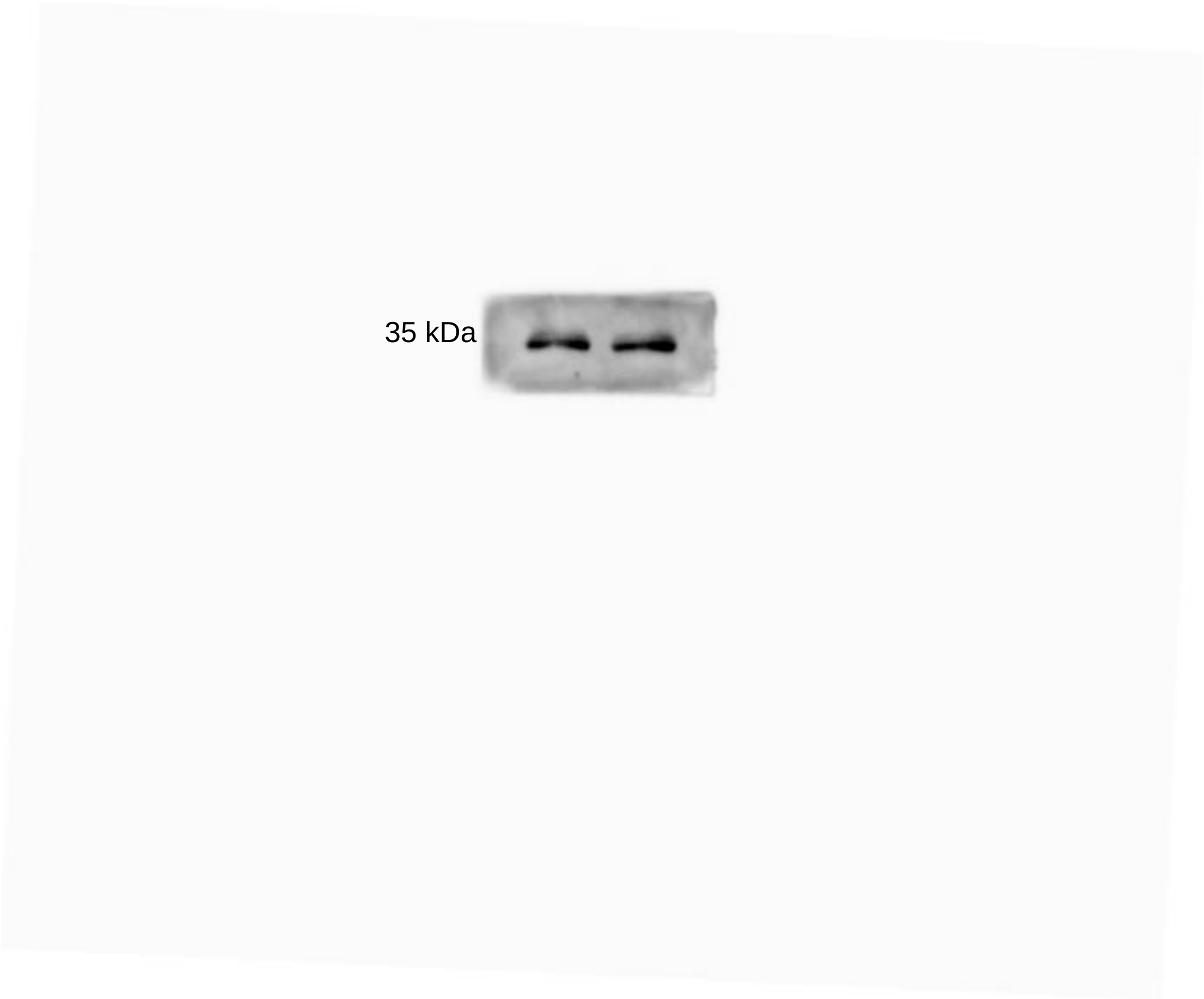


**Fig 6J**

**IB: MARCHF5 (mito)**

Groups (ES2 cells): shCtrl, shSLC25A46

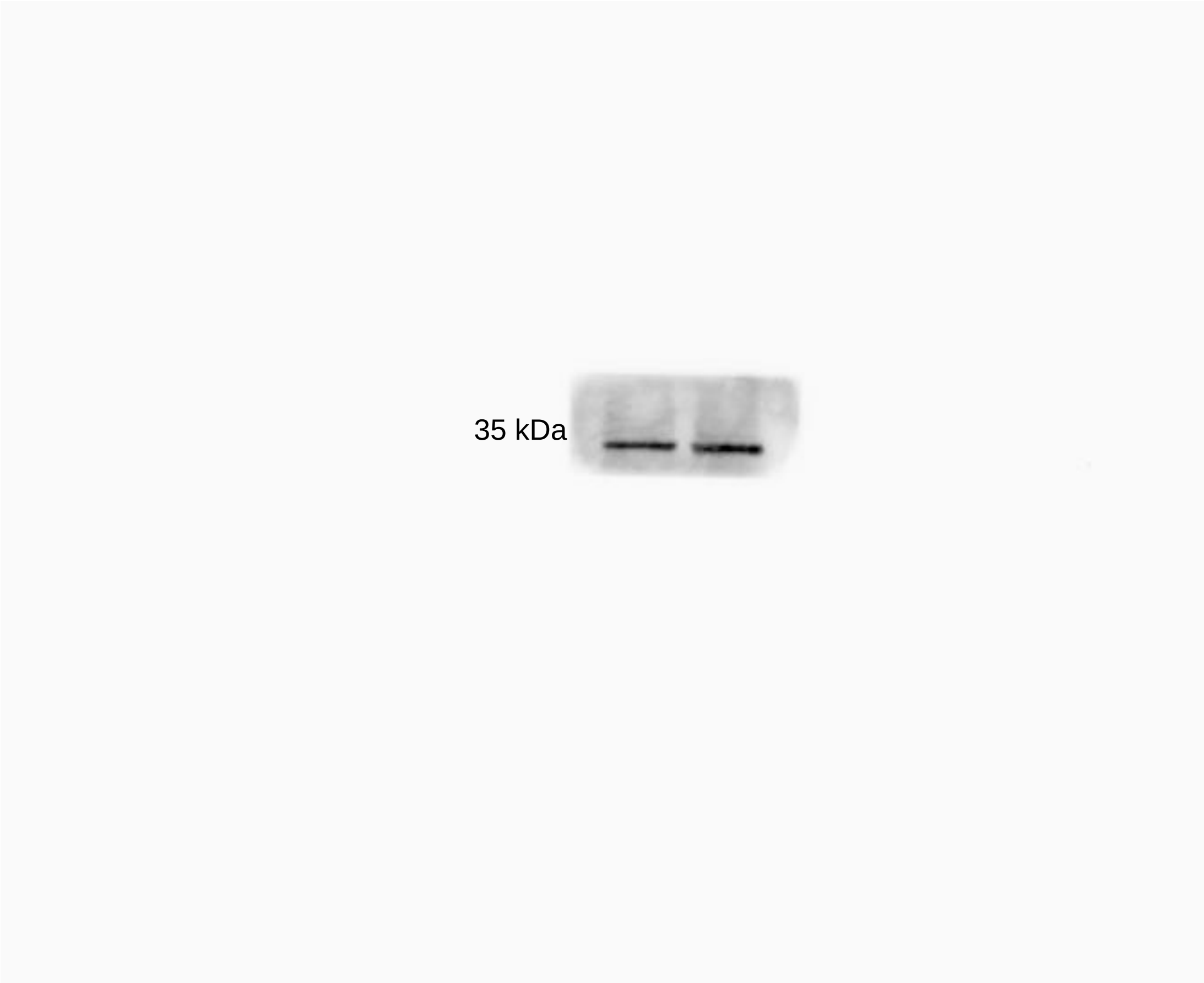


**Fig 6J**

**IB: VDAC (mito)**

Groups (ES2 cells): shCtrl, shSLC25A46

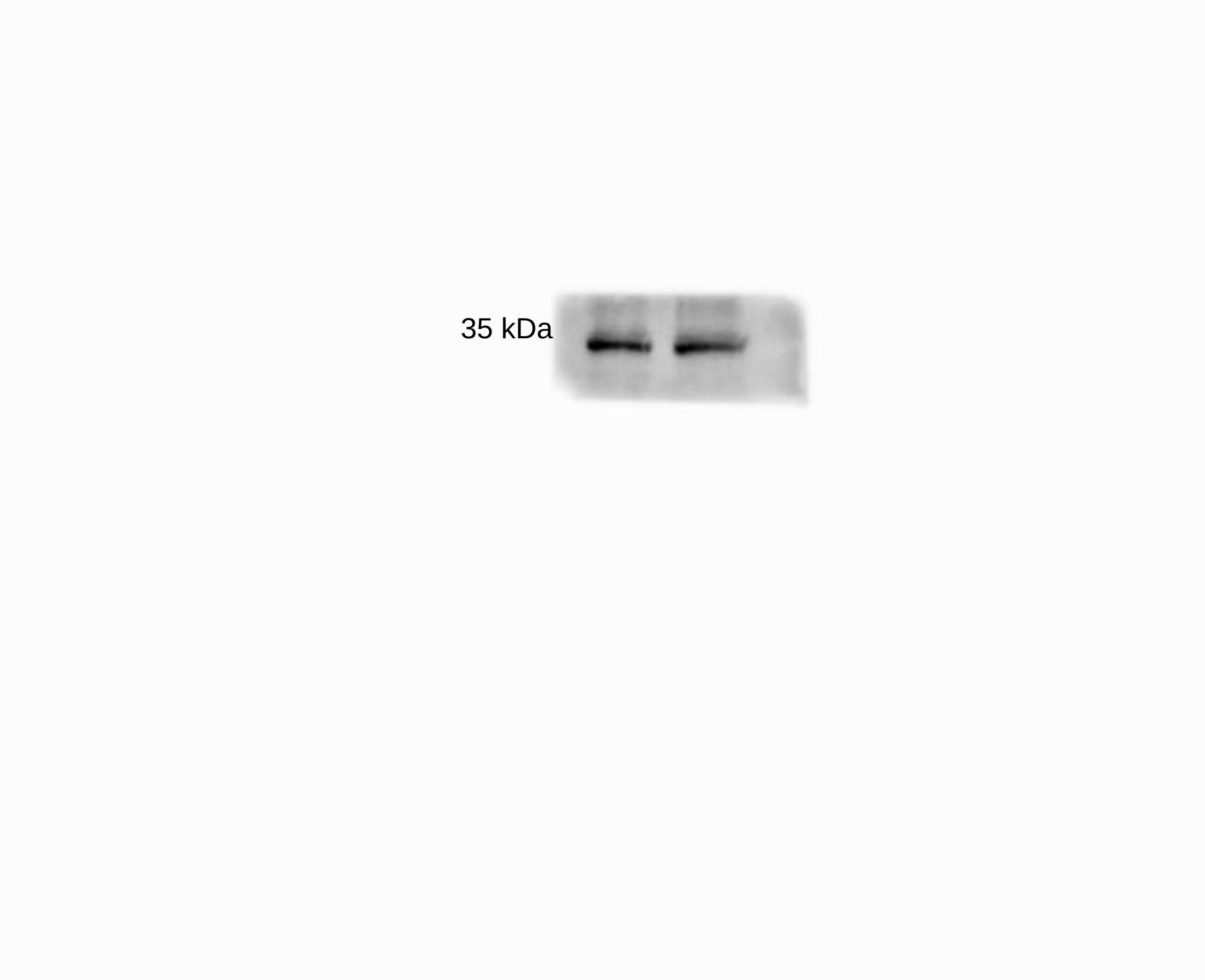


**Fig 6J**

**IB: MARCHF5(mito)**

Groups (HEY cells): EV, SLC25A46

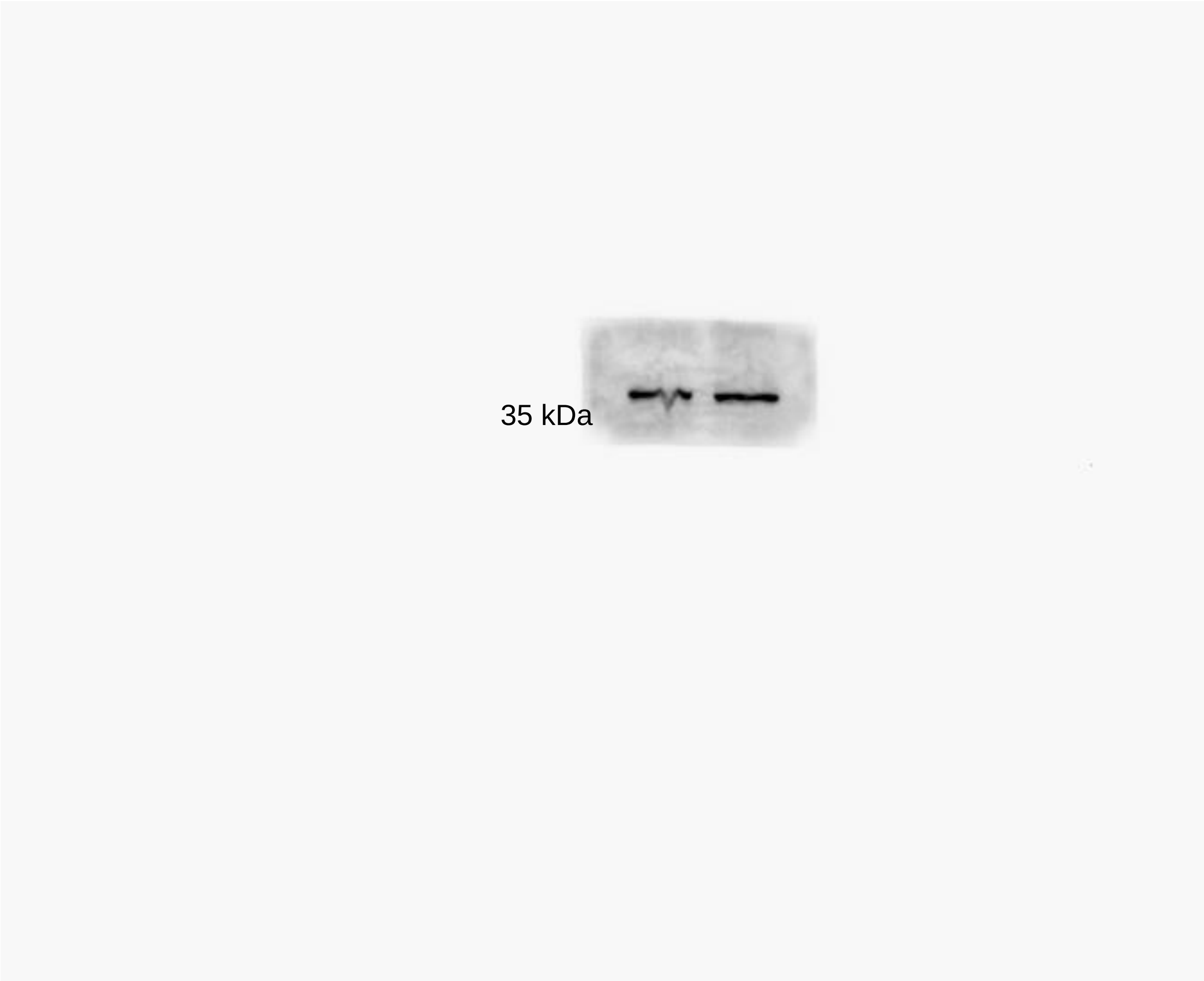


**Fig 6J**

**IB: β-actin (mito)**

Groups (HEY cells): EV, SLC25A46

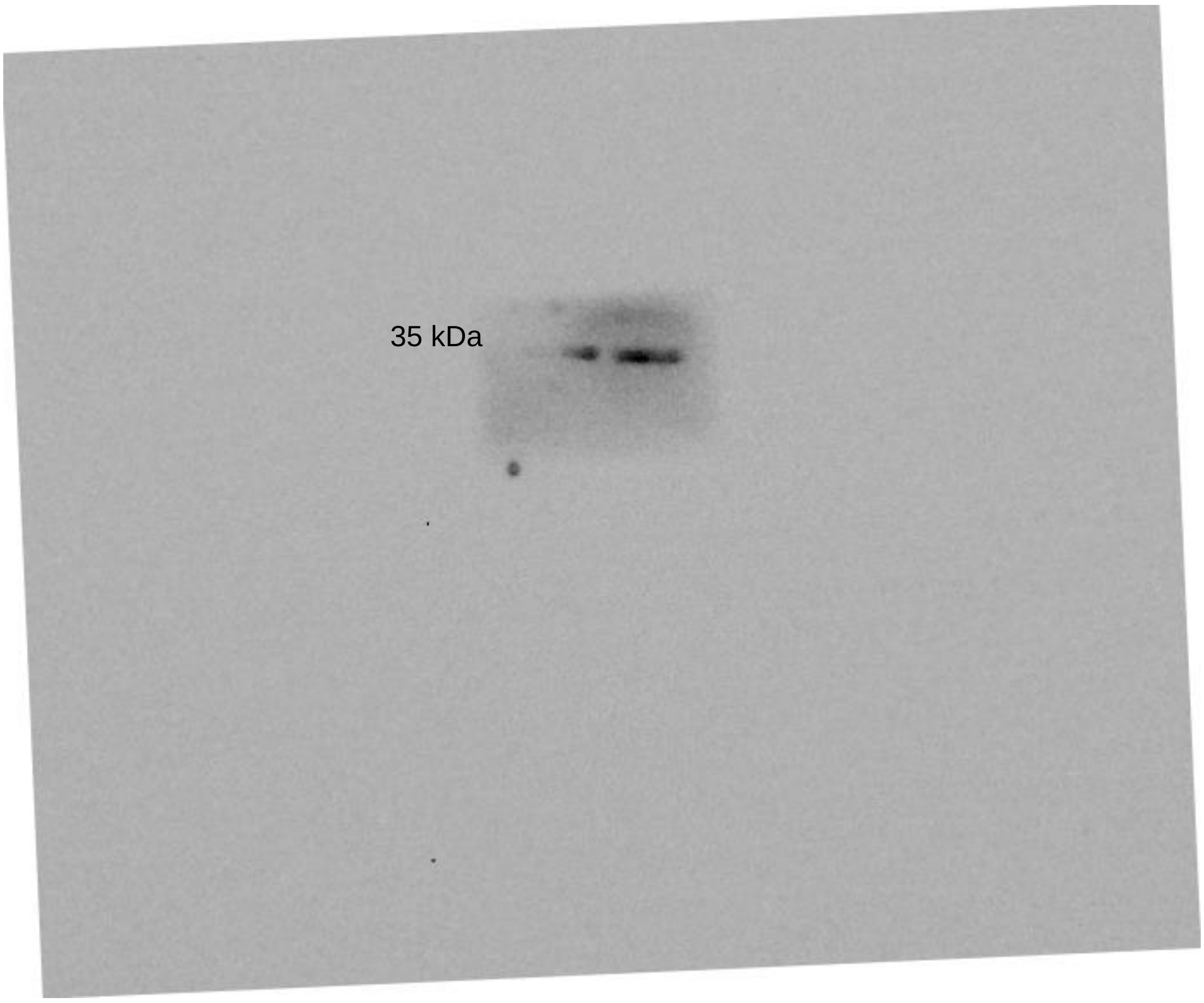


**Fig 6K**

**IB: MARCHF5**

Groups (ES2 cells): shCtrl+MG132, shSLC25A46+MG132

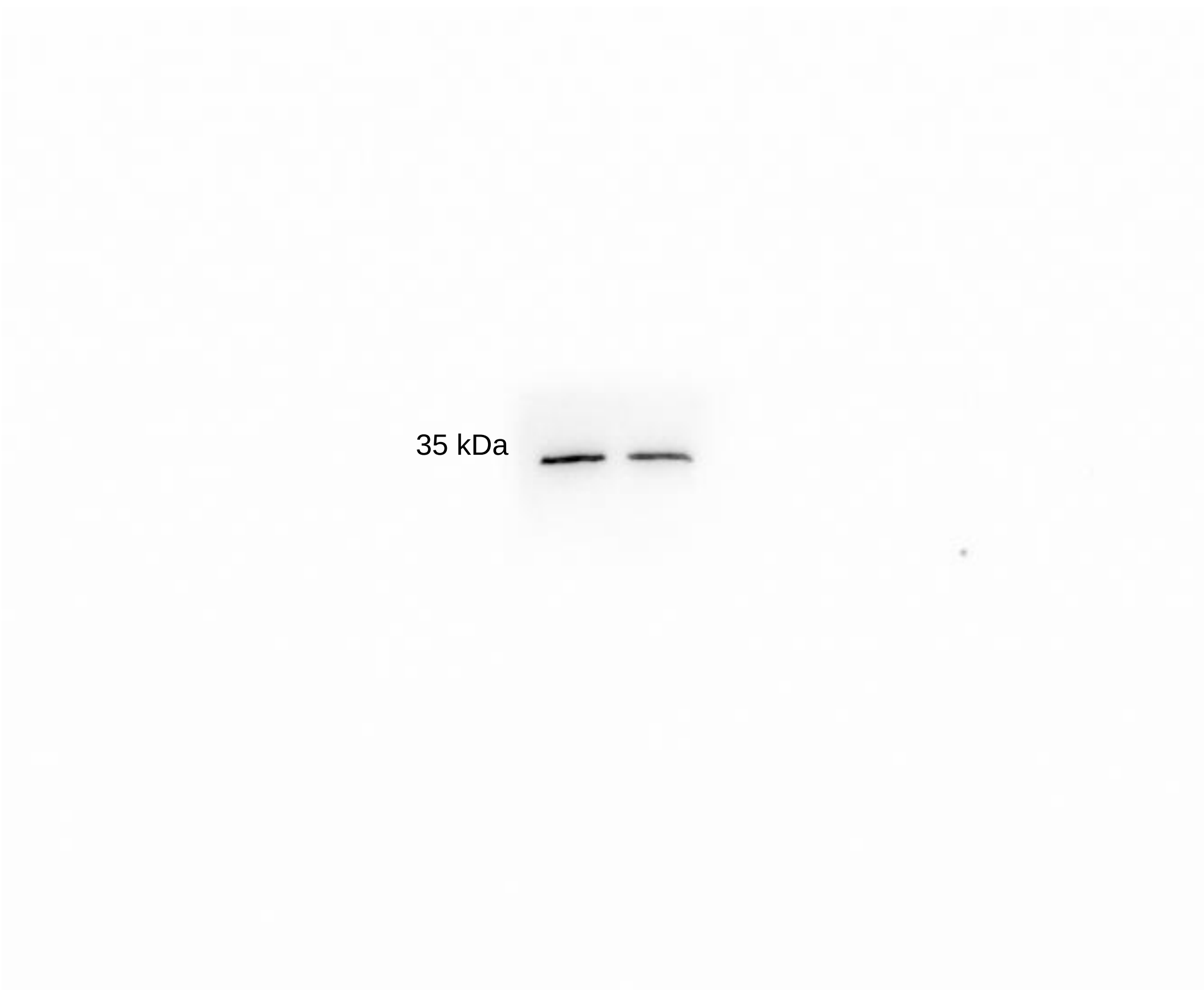


**Fig 6J**

**IB: CACT**

Groups (ES2 cells): shCtrl+MG132, shSLC25A46+MG132

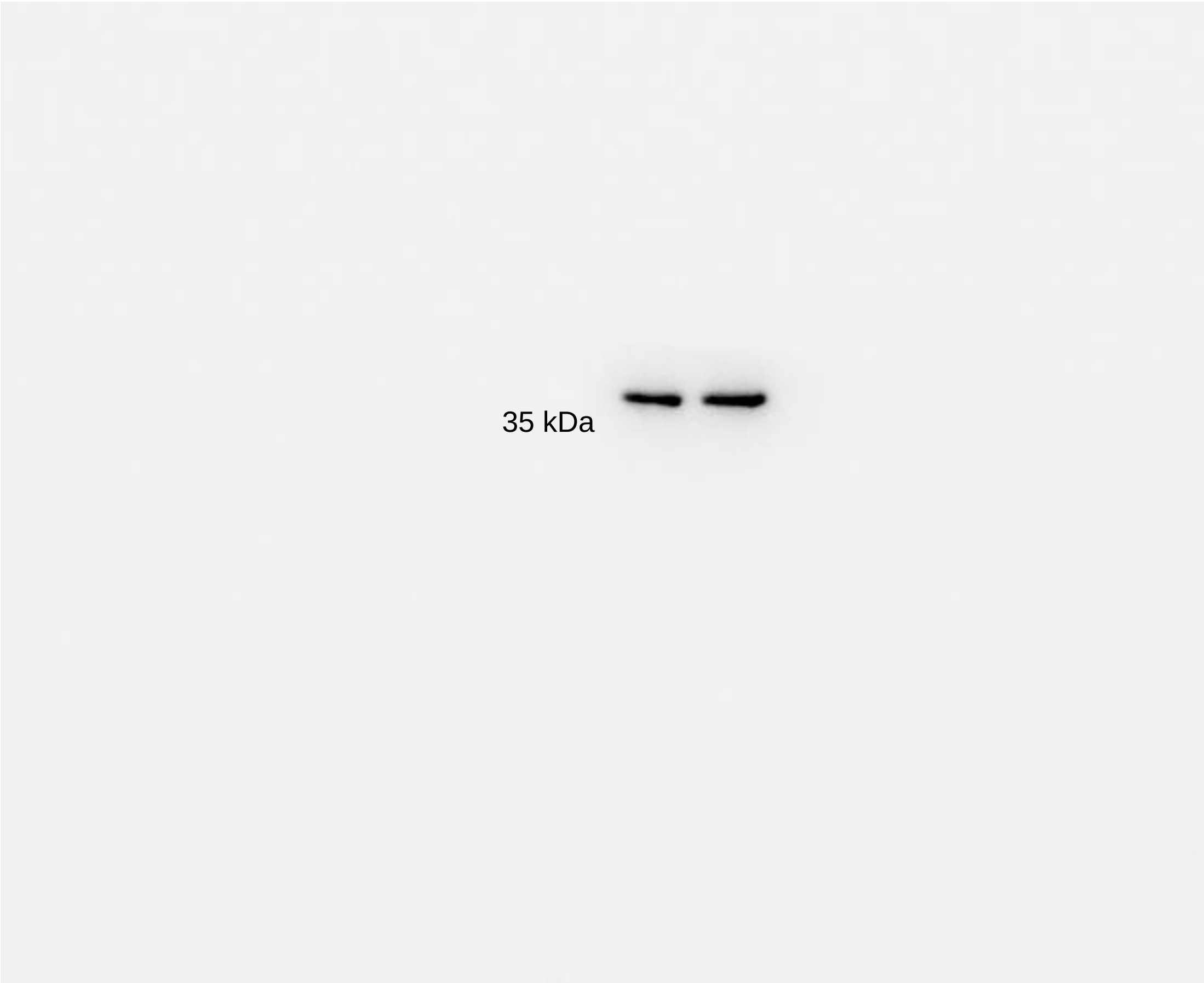


**Fig 6K**

**IB: β-actin**

Groups (ES2 cells): shCtrl+MG132, shSLC25A46+MG132

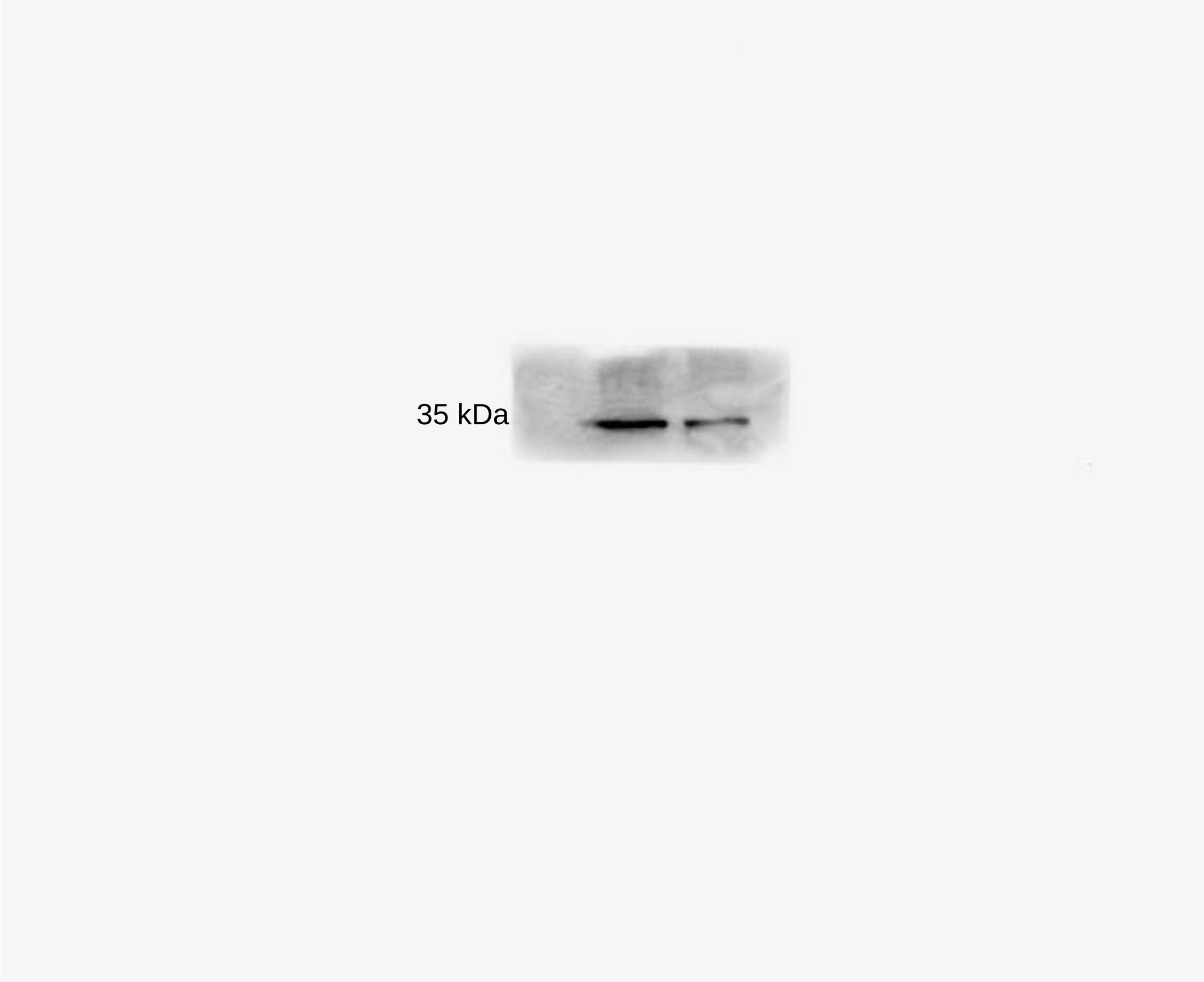


**Fig 6K**

**IB: MARCHF5**

Groups (HEY cells): EV+MG132, EV+MG132

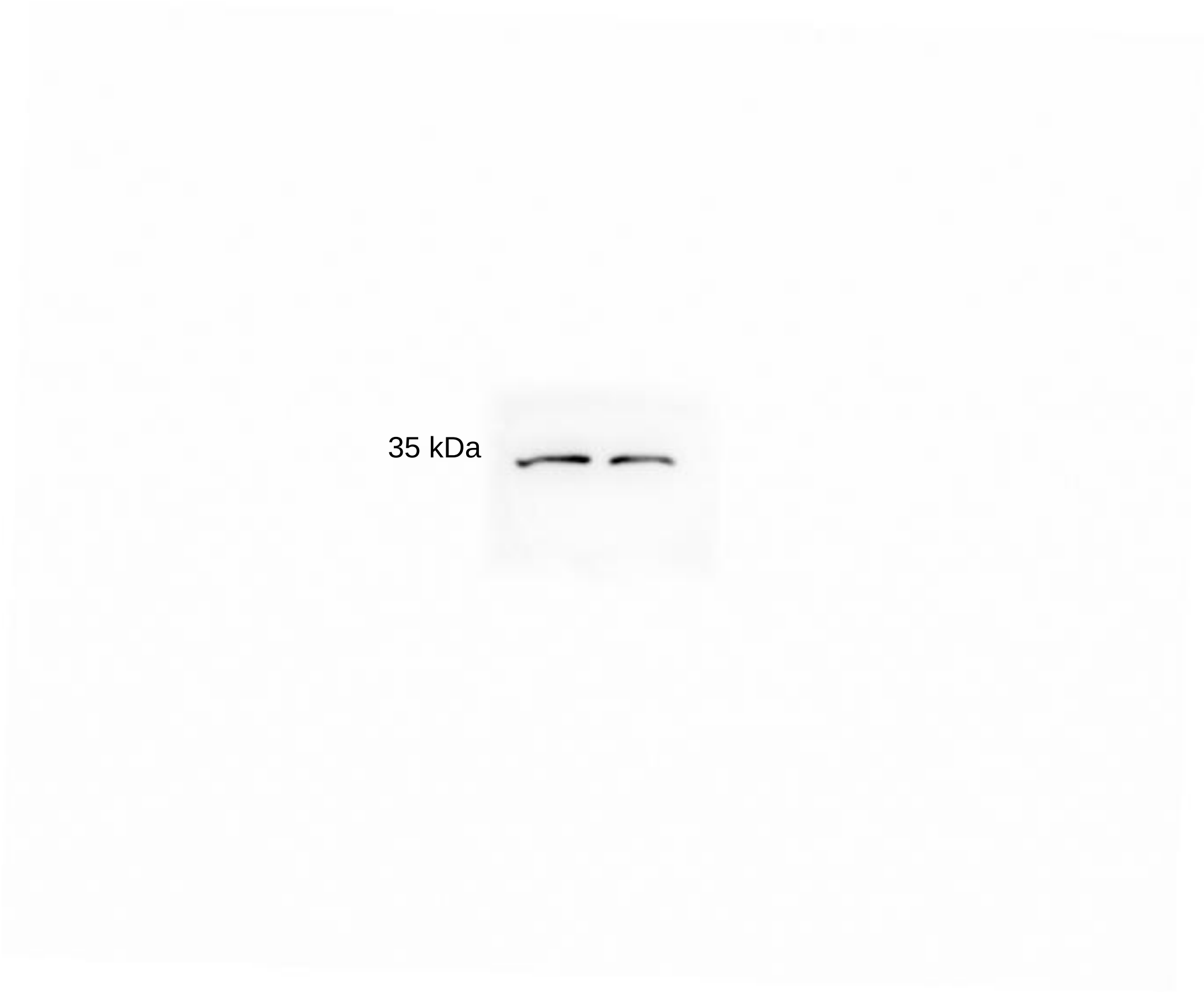


**Fig 6K**

**IB: CACT**

Groups (HEY cells): EV+MG132, EV+MG132**Fig 6J**

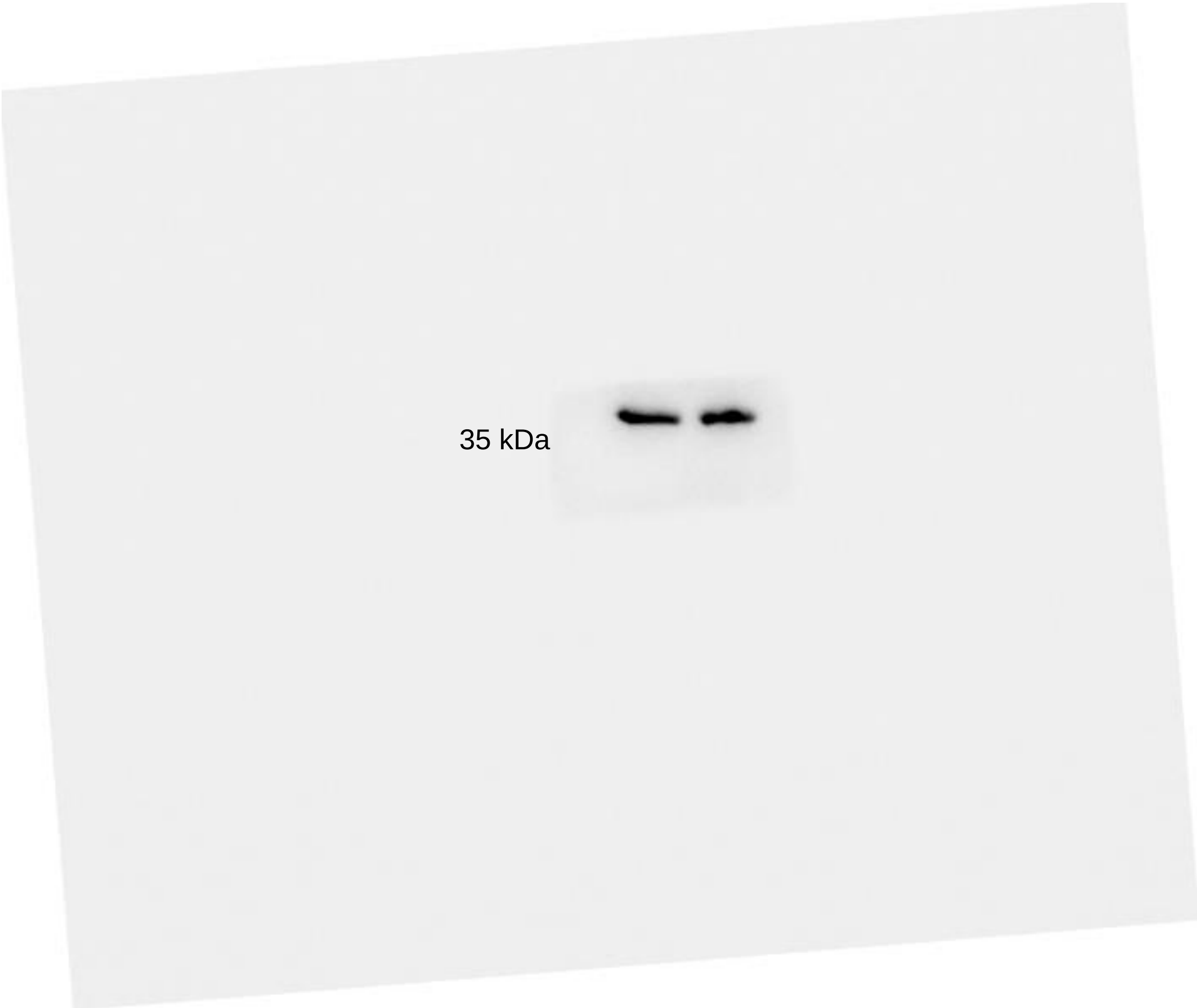


**Fig 6K**

**IB: β-actin**

Groups (HEY cells): EV+MG132, EV+MG132

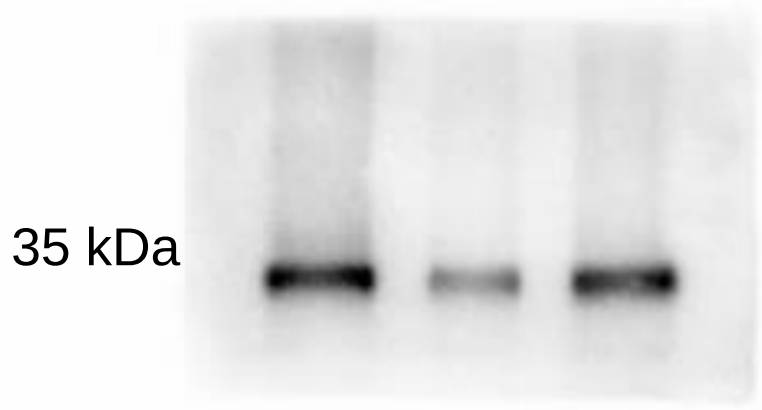


**Fig 6L**

**IB: CACT**

Groups (ES2 cells): shCtrl, shSLC25A46, shSLC25A46+shMARCHF5

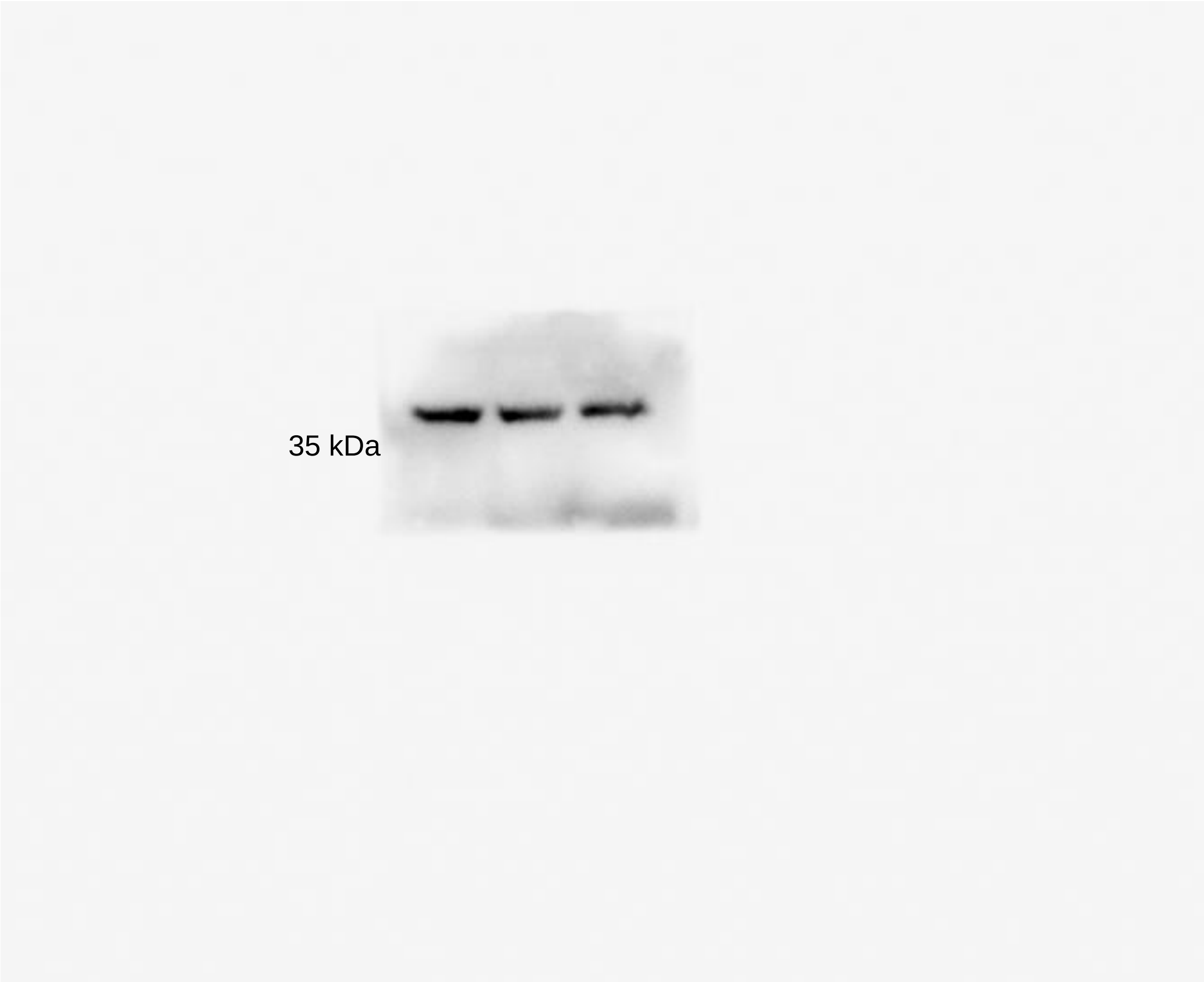


**Fig 6L**

**IB: β-actin**

Groups (ES2 cells): shCtrl, shSLC25A46, shSLC25A46+shMARCHF5

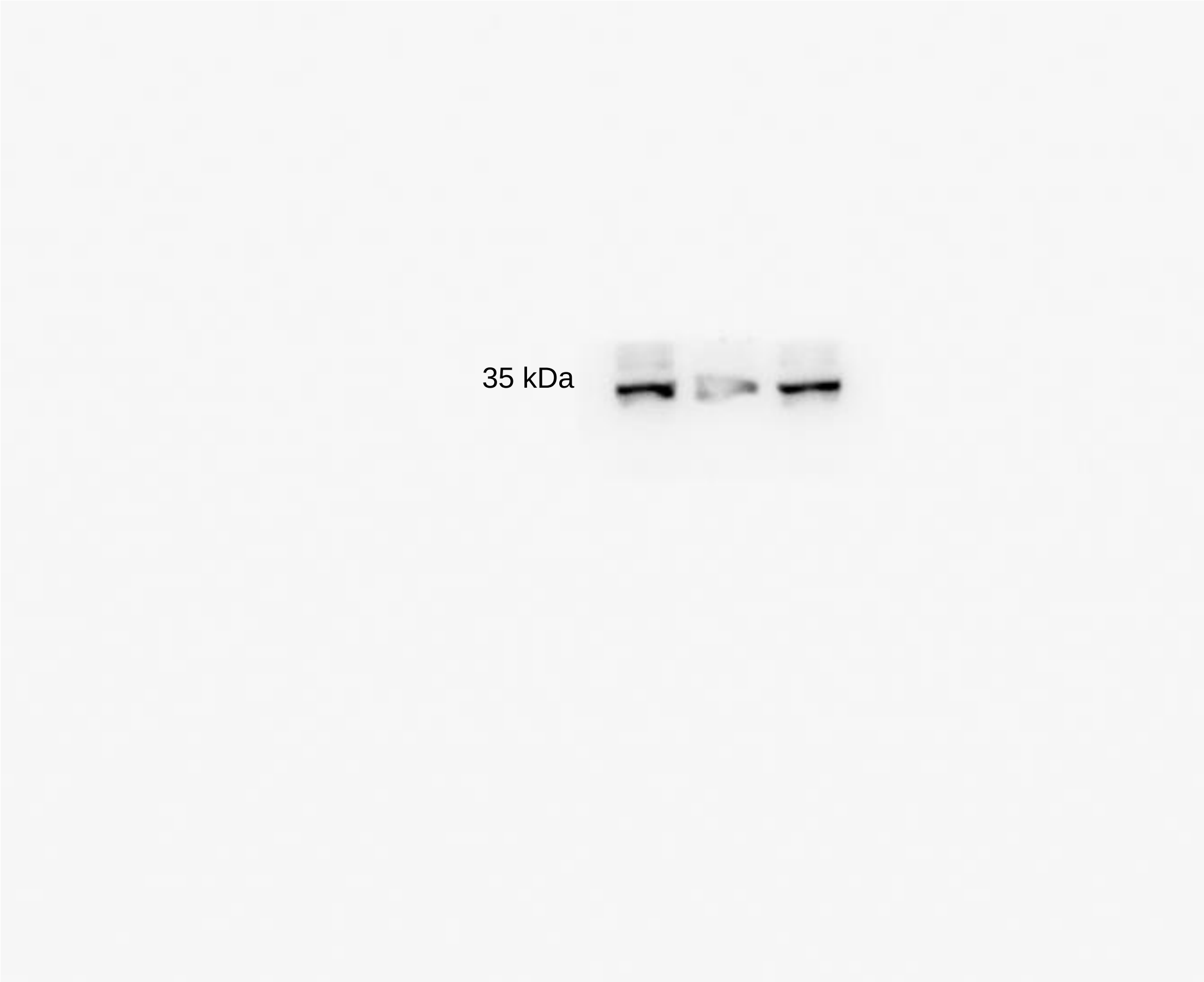


**Fig 6L**

**IB: CACT**

Groups (SKOV3 cells): shCtrl, shSLC25A46, shSLC25A46+shMARCHF5

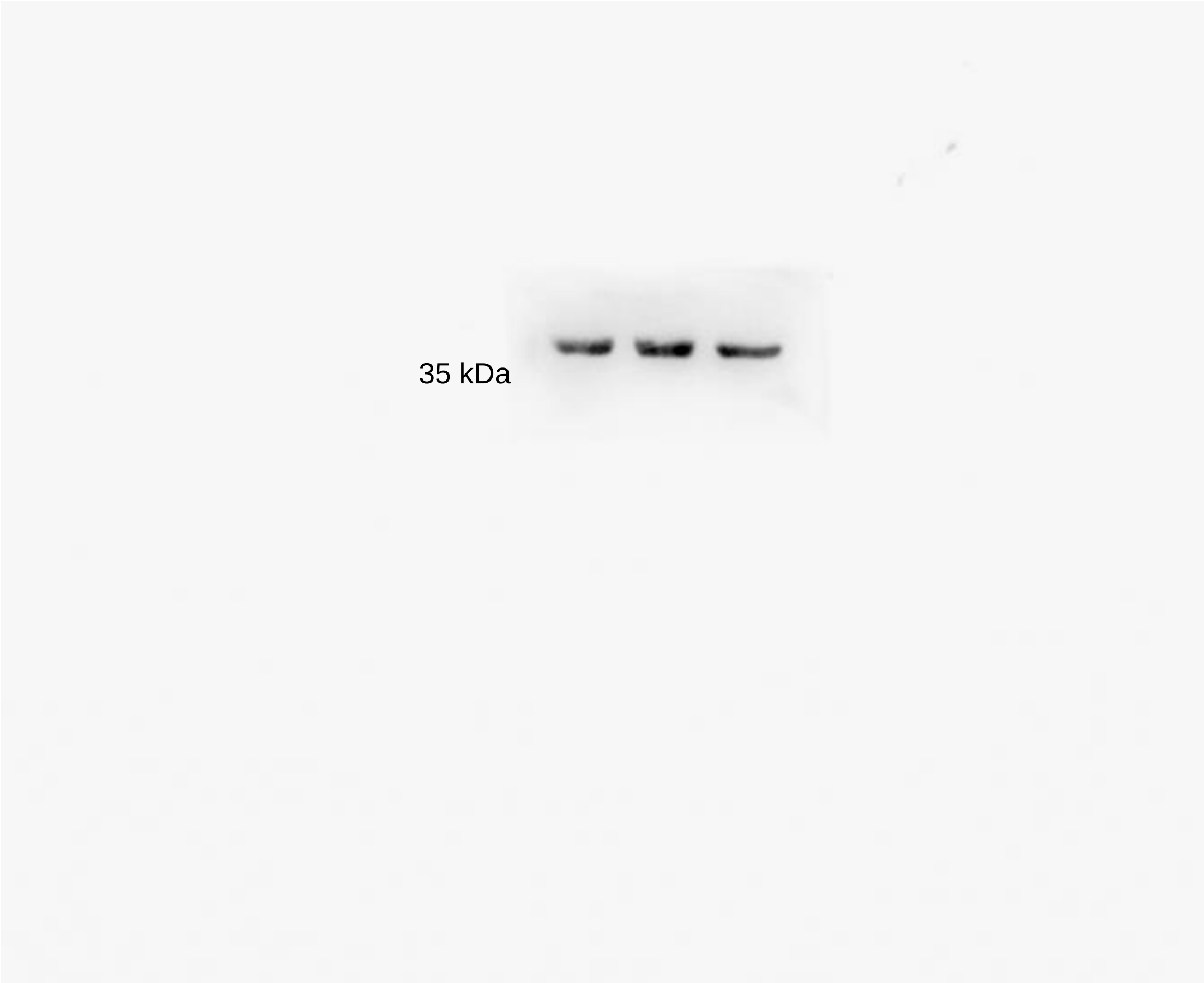


**Fig 6L**

**IB: β-actin**

Groups (SKOV3 cells): shCtrl, shSLC25A46, shSLC25A46+shMARCHF5

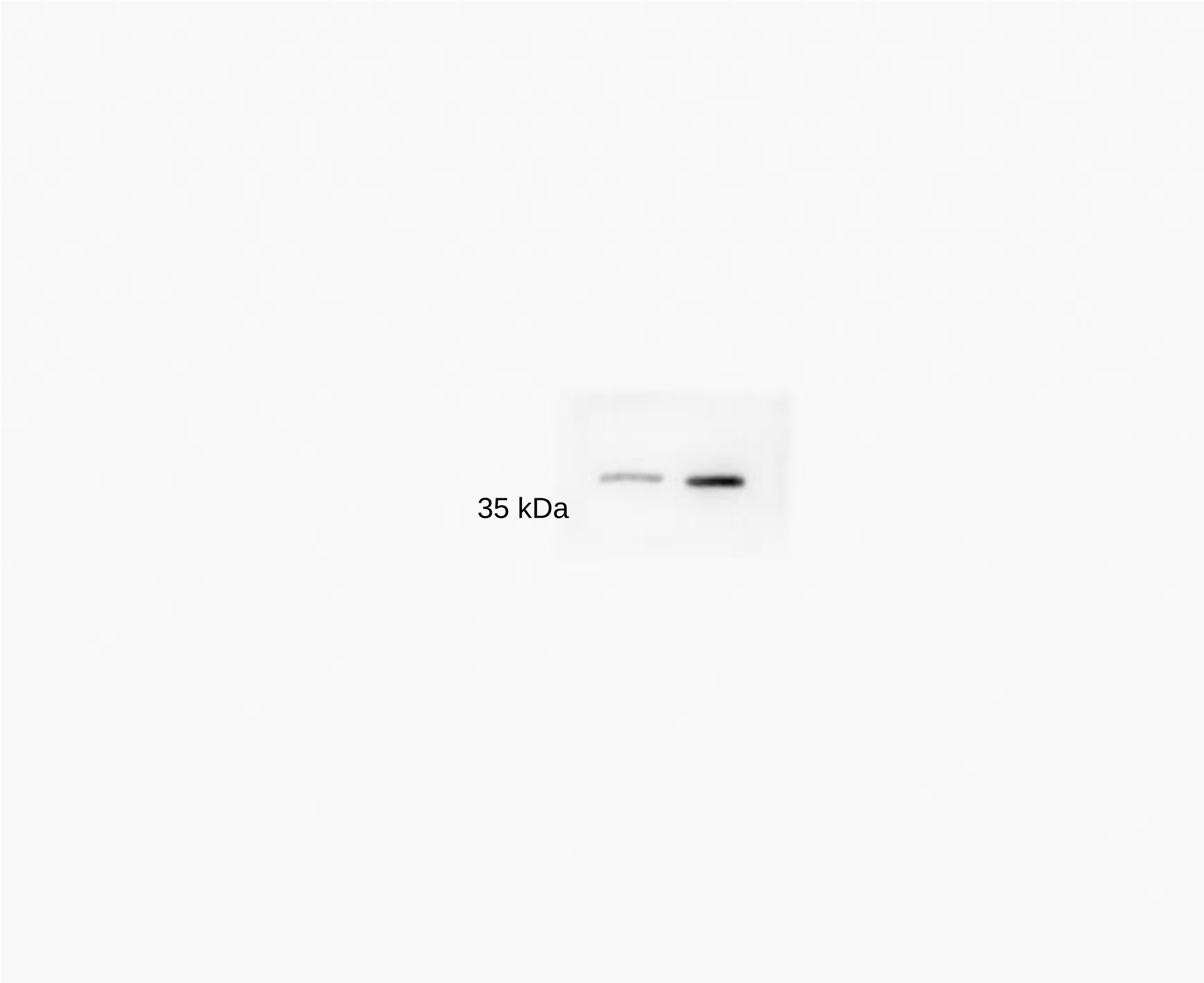


**Fig 8K**

**IB: SLC25A46**

Groups (HEY cells): Parental, Resistant

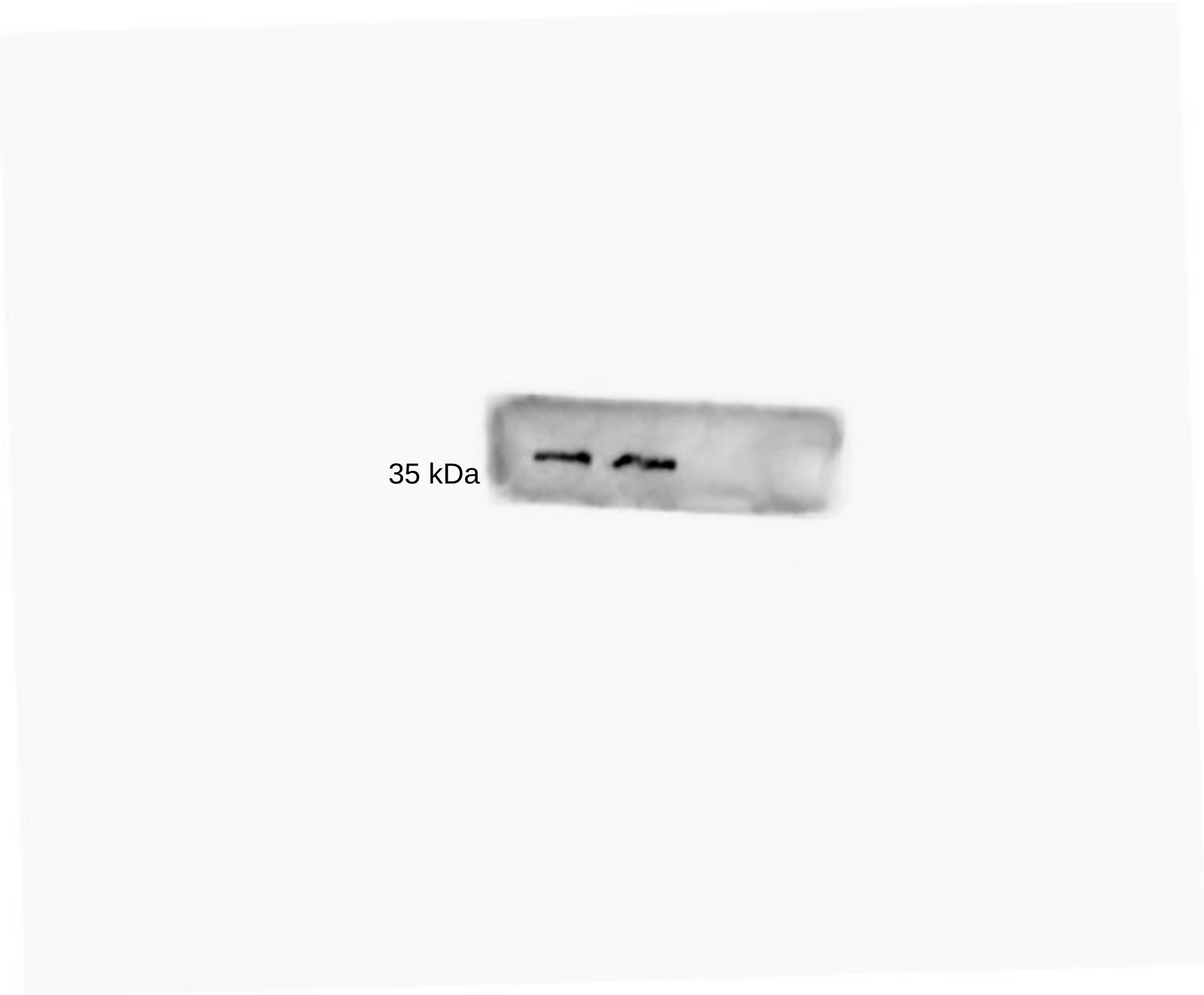


**Fig 8K**

**IB: β-actin**

Groups (HEY cells): Parental, Resistant

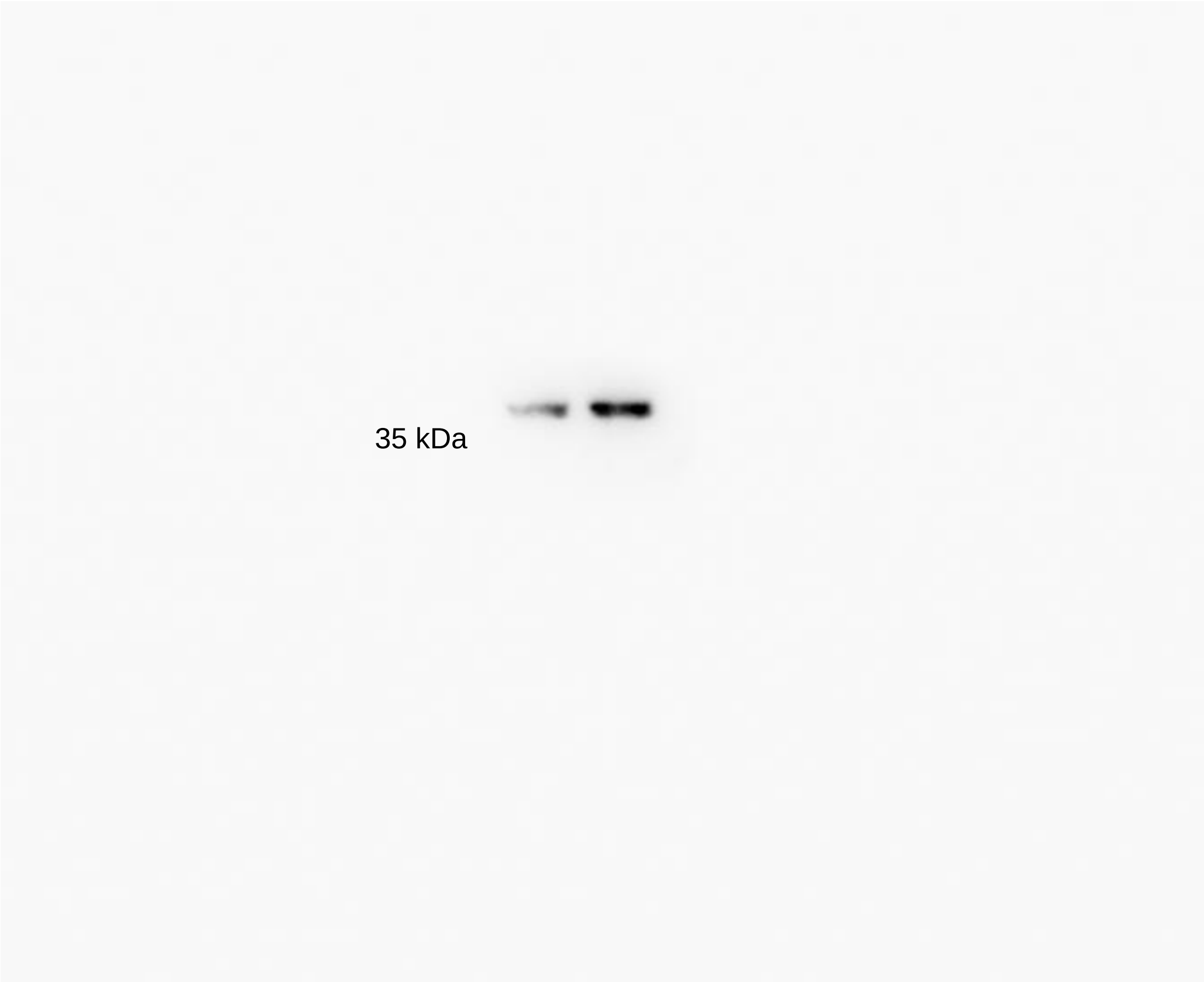


**Fig 8K**

**IB: SLC25A46**

Groups (SKOV3 cells): Parental, Resistant

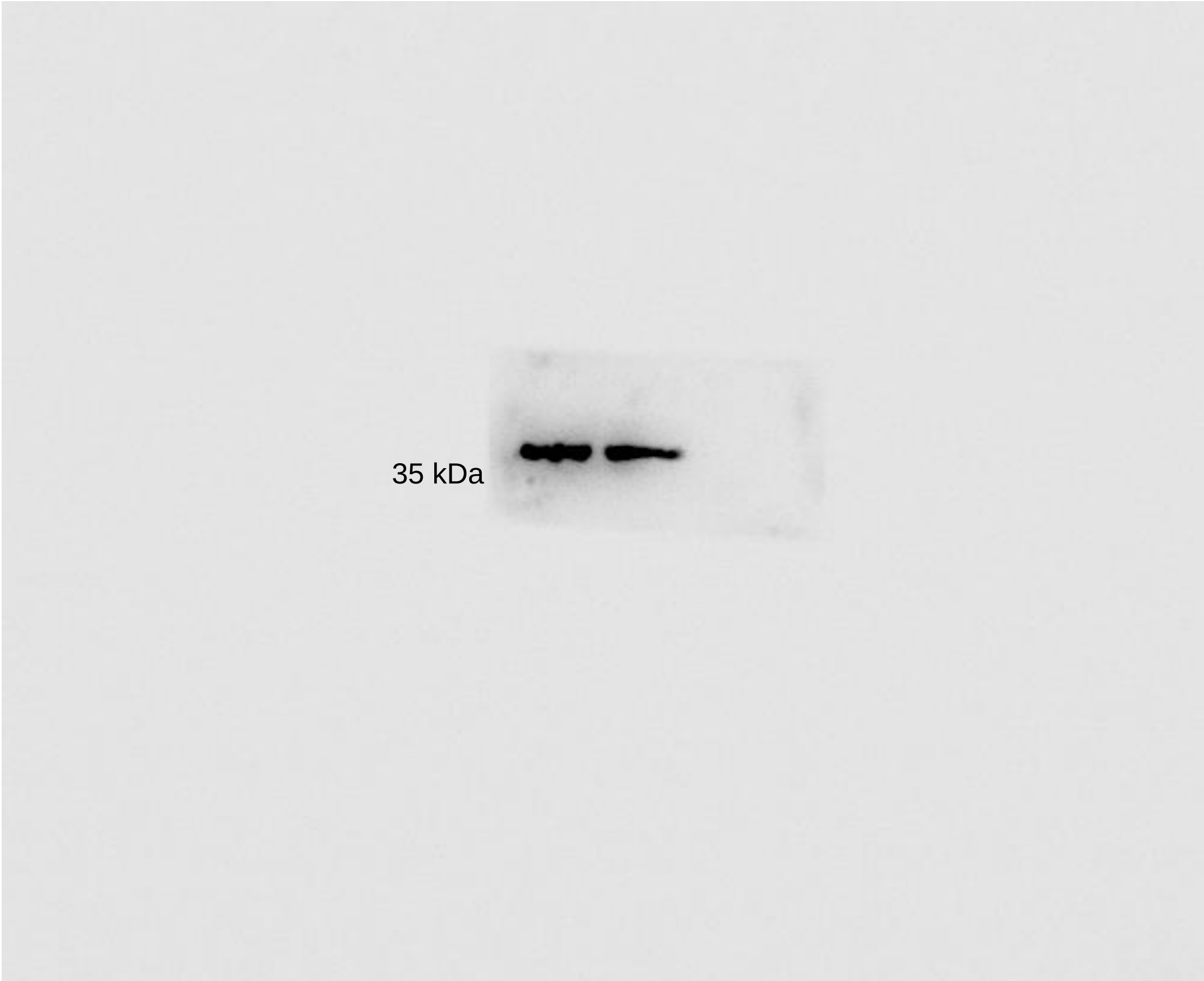


**Fig 8K**

**IB: β-actin**

Groups (SKOV3 cells): Parental, Resistant

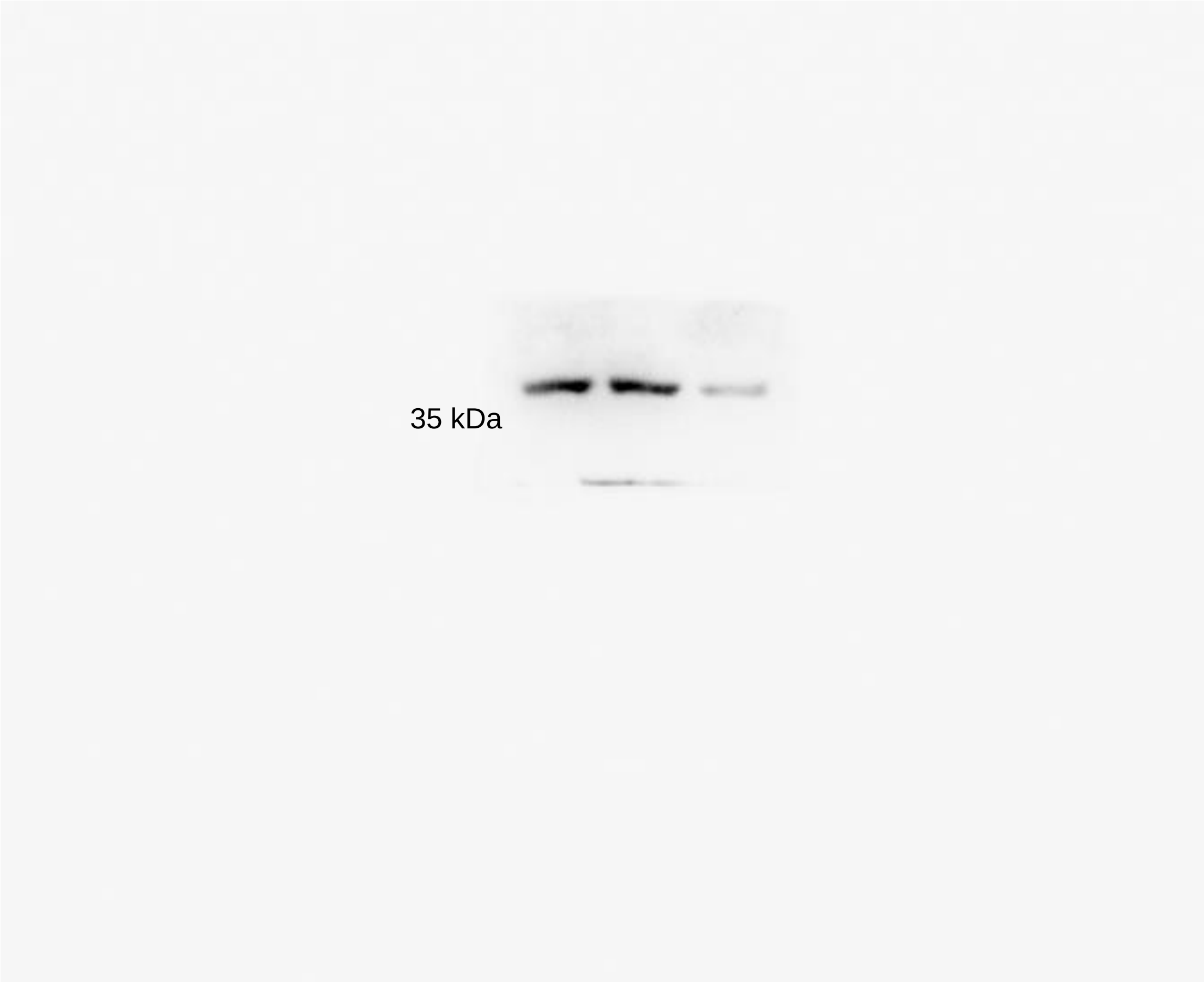


**Fig 9D**

**IB: SLC25A46**

Groups (ES2 cells): shCtrl, shHOXB13, shPBX1

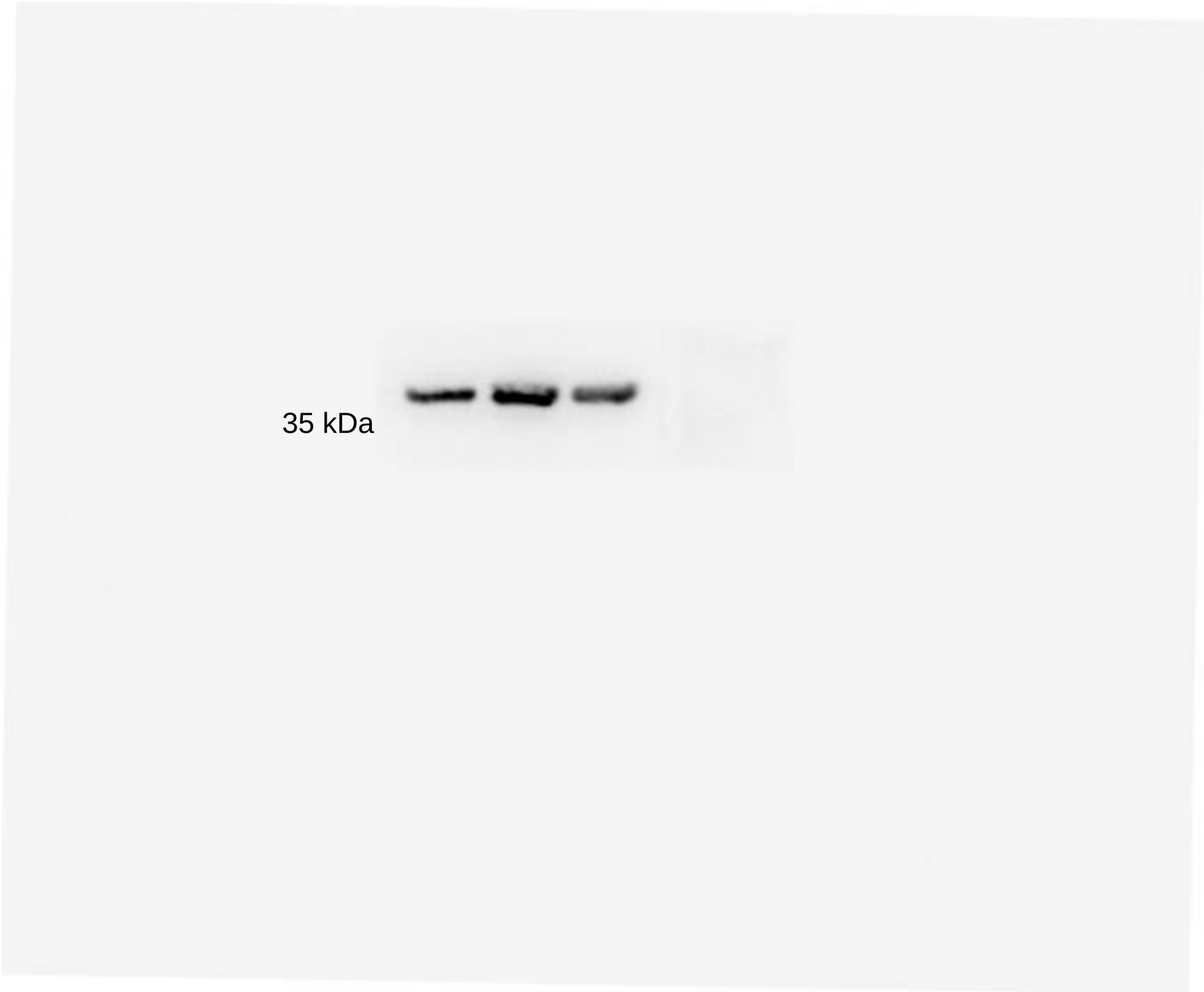


**Fig 9D**

**IB: β-actin**

Groups (ES2 cells): shCtrl, shHOXB13, shPBX1

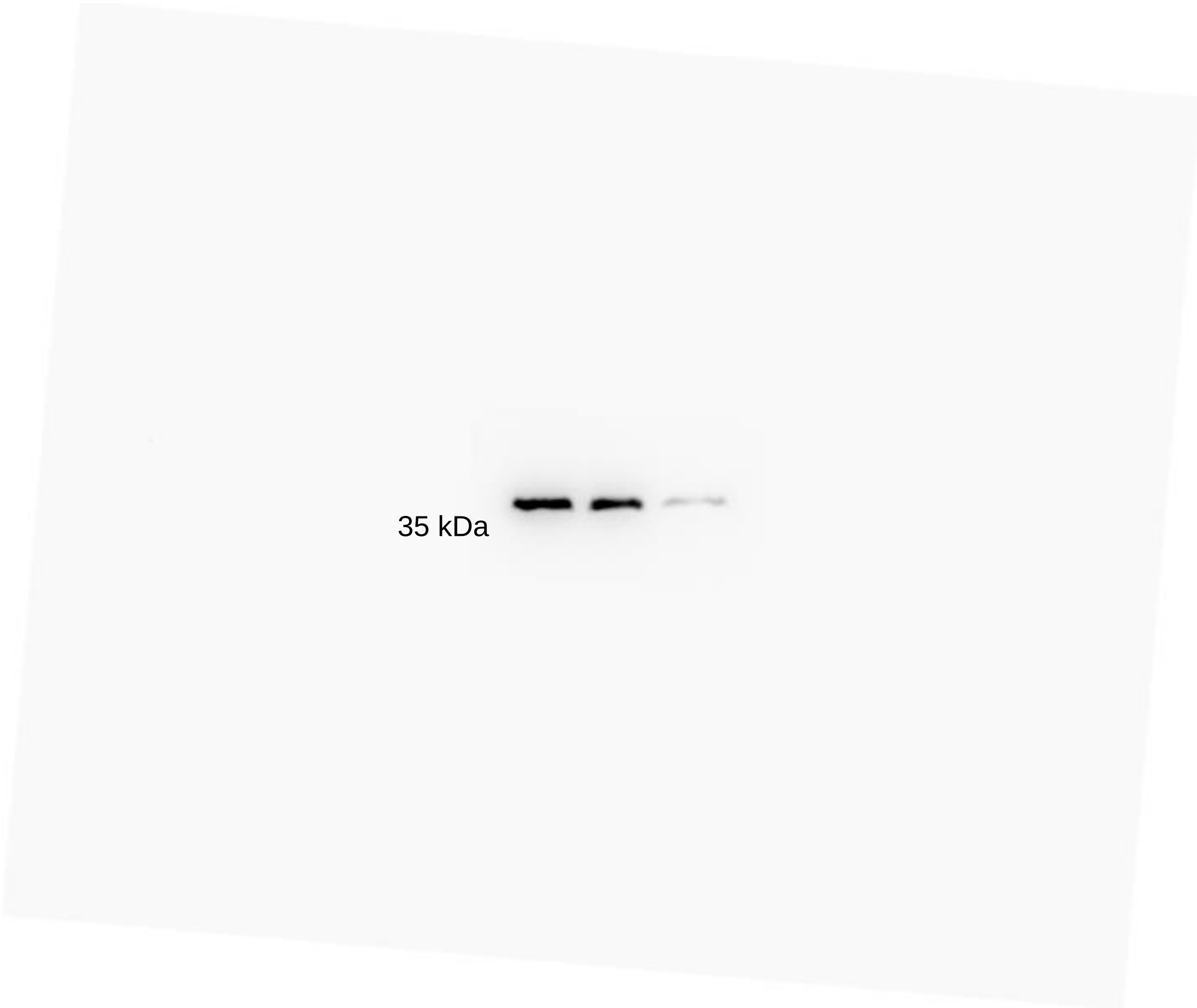


**Fig 9D**

**IB: SLC25A46**

Groups (SKOV3 cells): shCtrl, shHOXB13, shPBX1

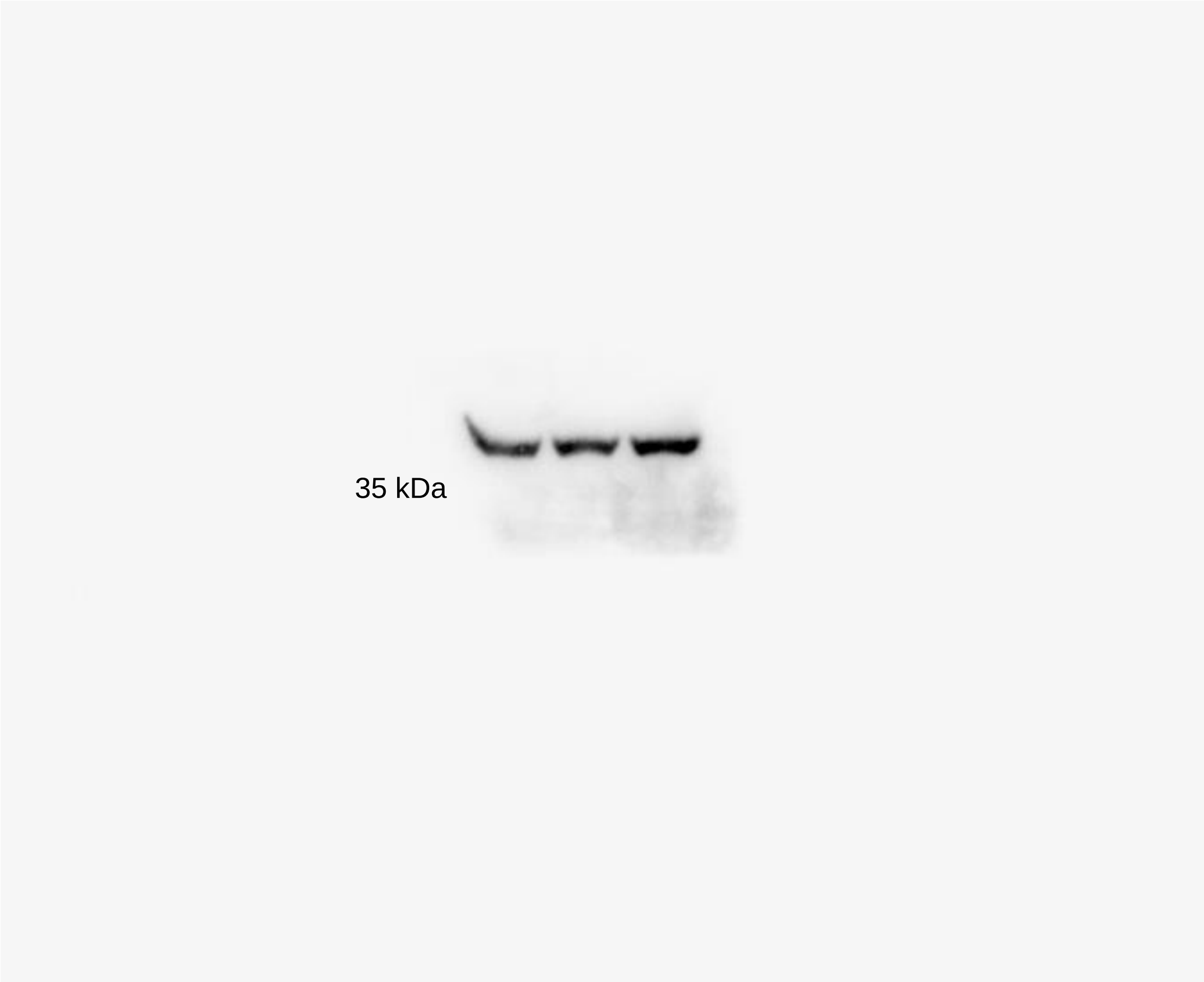


**Fig 9D**

**IB: β-actin**

Groups (SKOV3 cells): shCtrl, shHOXB13, shPBX1
