# Supplementary material for: Mitochondrial SLC25A46 Rewires Fatty Acid Oxidation to Promote Cell Proliferation and Ferroptosis Evasion in Ovarian Cancer by Stabilizing CACT
Source: Adv Sci (Weinh). 2026 Jul 20:e23969. Online ahead of print. doi: 10.1002/advs.202523969 (PMC13383691; doi:10.1002/advs.202523969)
Supplement: Supplementary file 1 — Supporting File 1: advs76689‐sup‐0001‐SuppMat.docx. [file ADVS-9999-e23969-s002.docx]

**Supplemental information**

**Mitochondrial SLC25A46 rewires fatty acid oxidation to promote cell proliferation and ferroptosis evasion in ovarian cancer by stabilizing CACT**

**Supplemental figures**

**Figure S1.** **(A)** Immunofluorescence staining (IF) analysis for co-localization of SLC25A46 with VDAC in ES2 and SKOV3 cells (Scale bar = 5 µm). **(B)** Upregulations of SLC25A46 expression were also observed in a list of other cancer types using the online Sangerbox database (GBM, Glioblastoma multiforme; GBMLGG, Glioma; LGG, Brain Lower Grade Glioma; ESCA, Esophageal carcinoma; STES, Stomach and Esophageal carcinoma; COAD, Colon adenocarcinoma; COADREAD, Colon adenocarcinoma/Rectum adenocarcinoma Esophageal carcinoma; STAD, Stomach adenocarcinoma; LIHC, Liver hepatocellular carcinoma; PAAD, Pancreatic adenocarcinoma; ALL, Acute Lymphoblastic Leukemia; LAML, Acute Myeloid Leukemia; CHOL, Cholangiocarcinoma).

**
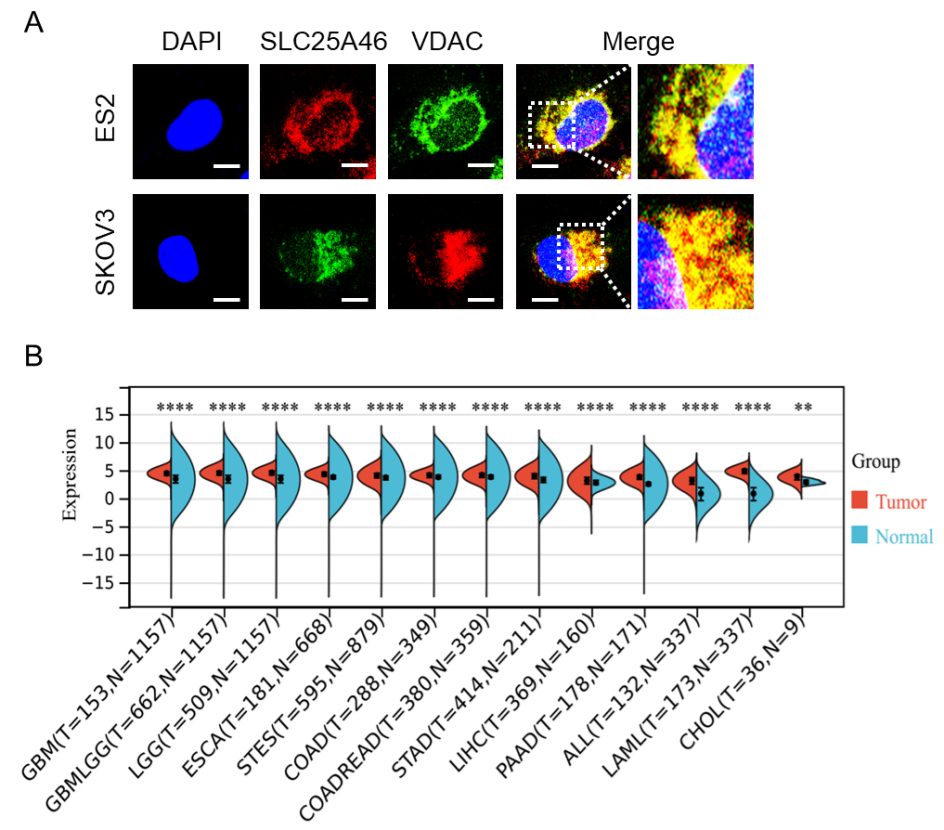
**

**Figure S2. (A-B)** SLC25A46 knockdown efficiencies were validated by qRT-PCR (A) and Western blotting (B, the left panel shows the blots; the right panel shows the densitometric analysis results). (C) Correlations between the expression of SLC25A46 and cell cycle regulators were analyzed using the public GEPIA database. **(D-E)** Wound healing (D) and transwell (E) assays were used for evaluating migration and invasion of ES2 and SKOV3 cells when SLC25A46 was knocked-down.


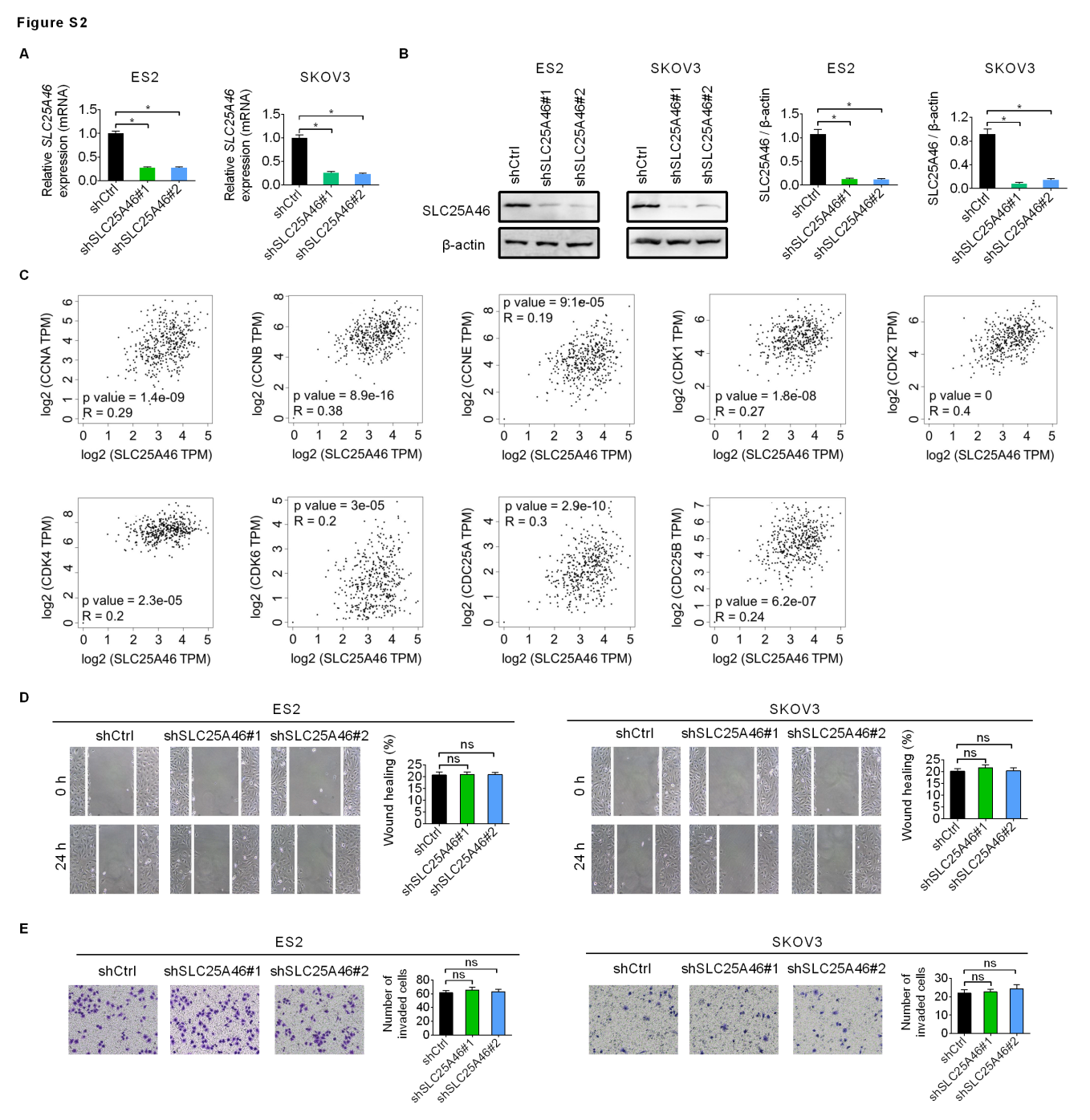


**Figure S3. (A)** SLC25A46 knockdown efficiency in ES2 cells prior to their injection into mice was tested by Western blot analysis. **(B)** Pulmonary metastasis nude mouse model was used for evaluating the effect of SLC25A46 knockdown on metastasis abilities of OC cells *in vivo* (Scale bar = 20 µm). **(C-D)** IHC staining assay was used for evaluating expressions of SLC25A46 (C), Ki-67 (D) and PCNA (E) in SLC25A46-silencing or control xenografts (Scale bar = 10 µm).

**
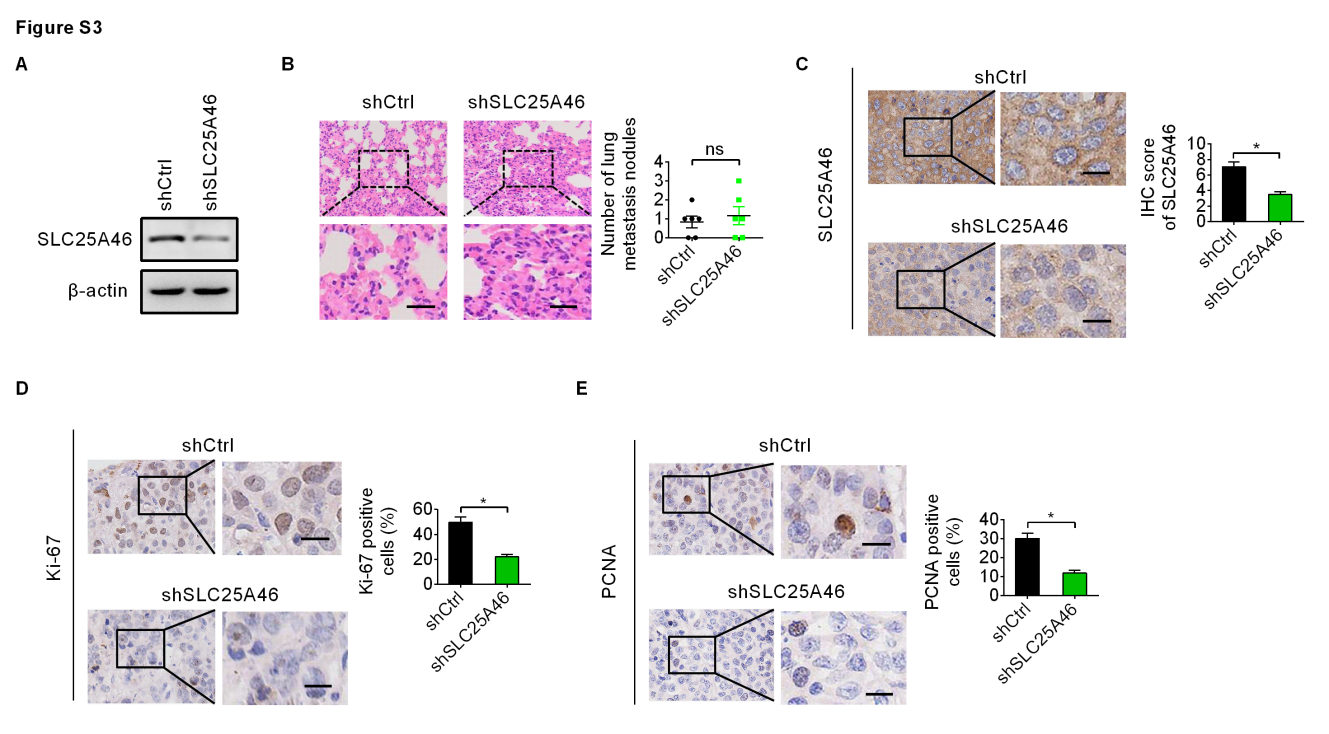
**

**Figure S4. Forced SLC25A46 expression promoted cell proliferation and suppressed cell death in OC cells. (A-B)** SLC25A46 upregulation efficiencies were validated by qRT-PCR (panel A) and Western blotting (B) assays. **(C-D)** CCK-8 cell viability (panel C) and colony formation (panel D) assays were used to examine the impact of SLC25A46 upregulation on the proliferation of OC cells. **(E-F)** EdU incorporation (panel E, Scale bar = 20 µm) and Annexin V/PI staining (panel F) assays were used to examine the impact of SLC25A46 upregulation on cell cycle progression and cell death in OC cells. **(G-H)** The effect of forced SLC25A46 expression on in vivo OC growth was evaluated by injecting stable SLC25A46-overexpressing and control HEY cells into nude mice (Panel G shows tumor growth curves and Panel H shows xenograft tumors and their weights). **(I-J)** Wound healing (panel I) and transwell (panel J) assays were used to examine the impact of SLC25A46 upregulation on migration and invasion of OC cells. **(K)** Pulmonary metastasis nude mouse model was used for evaluating the effect of forced SLC25A46 expression on metastasis abilities of OC cells *in vivo* (Scale bar = 20 µm).

**
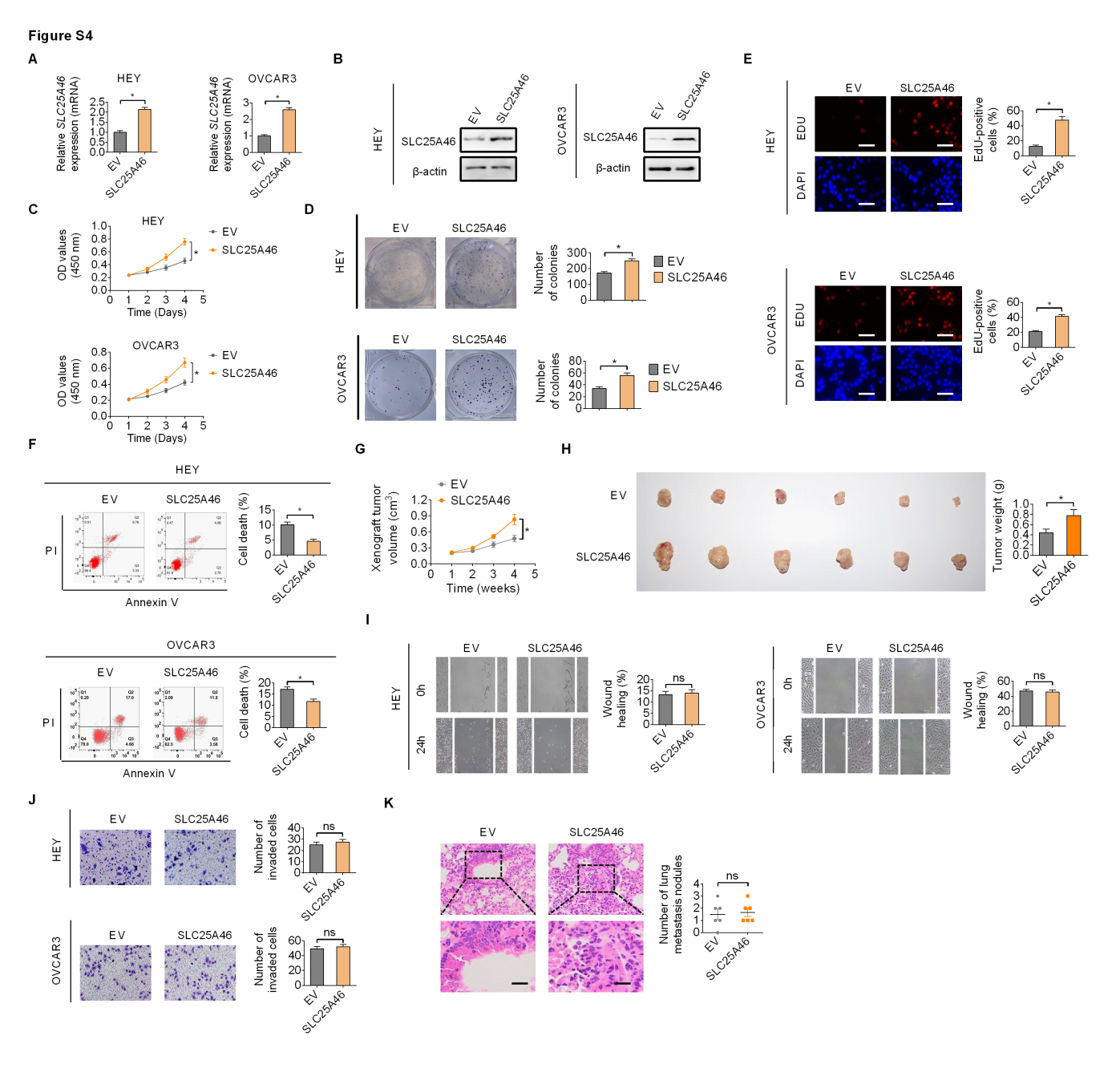
**

**Figure S5.** **(A)** Cell viability was determined by CCK-8 assay in ES2 and SKOV3 cells upon the treatment of a range of concentrations of inhibitors targeting different PCD pathways. **(B)** The impact of SLC25A46 knockdown or overexpression on the expressions of SOD2, CAT and GPX1 were assessed by Western blotting. **(C)** The impact of SLC25A46 knockdown or overexpression on GSSG/GSH ratio was analyzed. **(D)** BODIPY 493/503 was used to stain lipid droplets in SLC25A46 knockdown or overexpression OC cells (Scale bar = 10 µm)**. (E)** ATP production was assessed in SLC25A46 overexpression OC cells upon treatment with sulfo-N-succinimidyl oleate (SSO, 100 μM, 6 hours) or 5-(Tetradecyloxy)-2-furoic acid (TOFA, 20 μM, 6 hours).

**
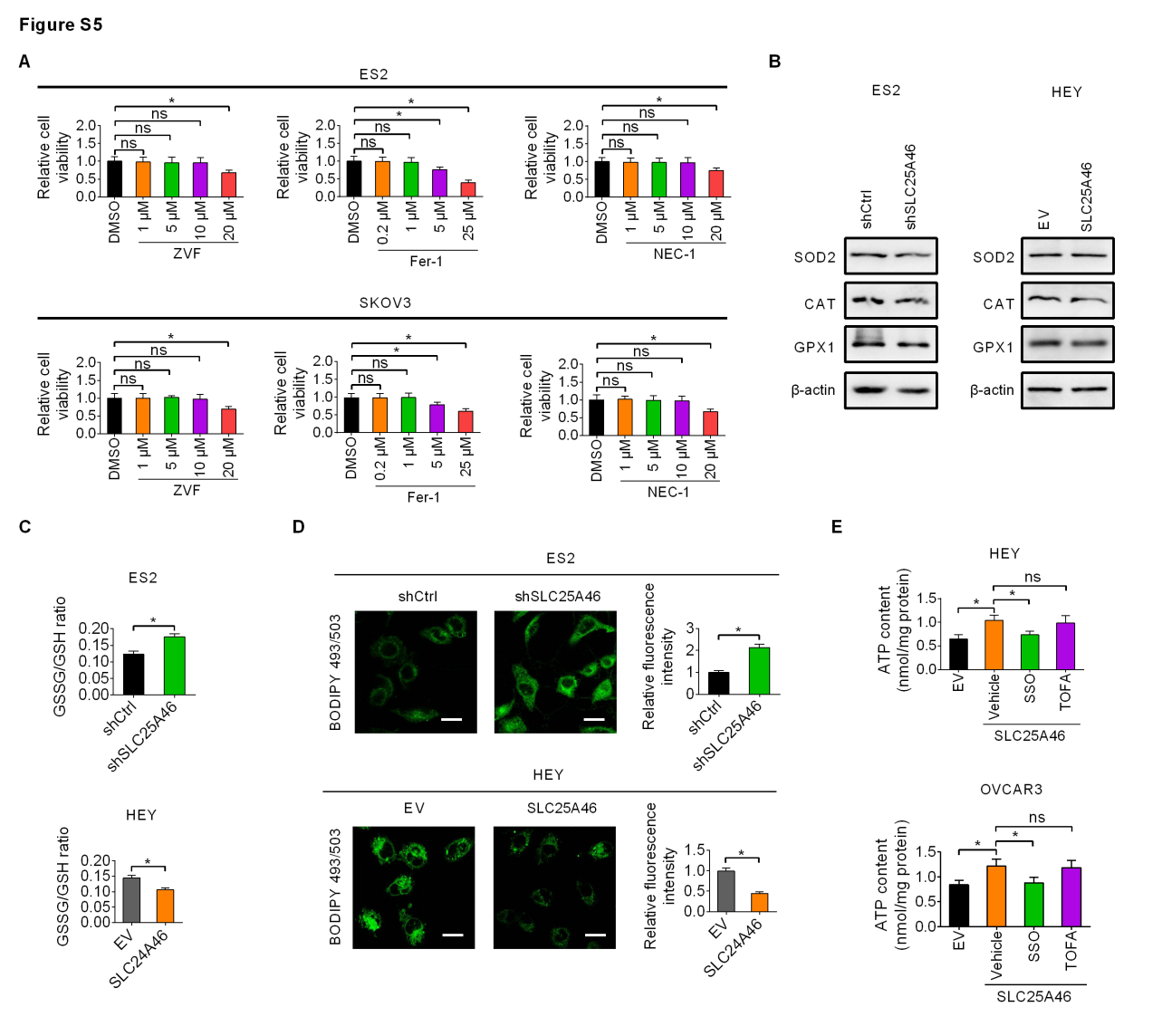
**

**Figure S6. (A)** The impact of SLC25A46 on CACT expression was determined by qRT-PCR assay in OC cells. **(B)** CO-IP assay was carried out to determine the interaction between MARCHF5 and CACT. **(C)** The effect of SLC25A46 silencing or overexpression on interaction between MARCHF5 and CACT was evaluated by CO-IP assay in OC cells. **(D)** Upregulation of CACT was observed in six cancer types using the online Sangerbox database (GBM, Glioblastoma multiforme; GBMLGG, Glioma; LGG, Brain Lower Grade Glioma; OV, Ovarian serous cystadenocarcinoma; PAAD, Pancreatic adenocarcinoma; LAML, Acute Myeloid Leukemia). **(E)** Immunofluorescence staining (IF) analysis for co-localization of CACT with VDAC in ES2 and SKOV3 cells (Scale bar = 5 µm). **(F)** Correlation between MARCHF5 and CACT expressions was analyzed in tumor tissue samples from OC patients (n=214).

**
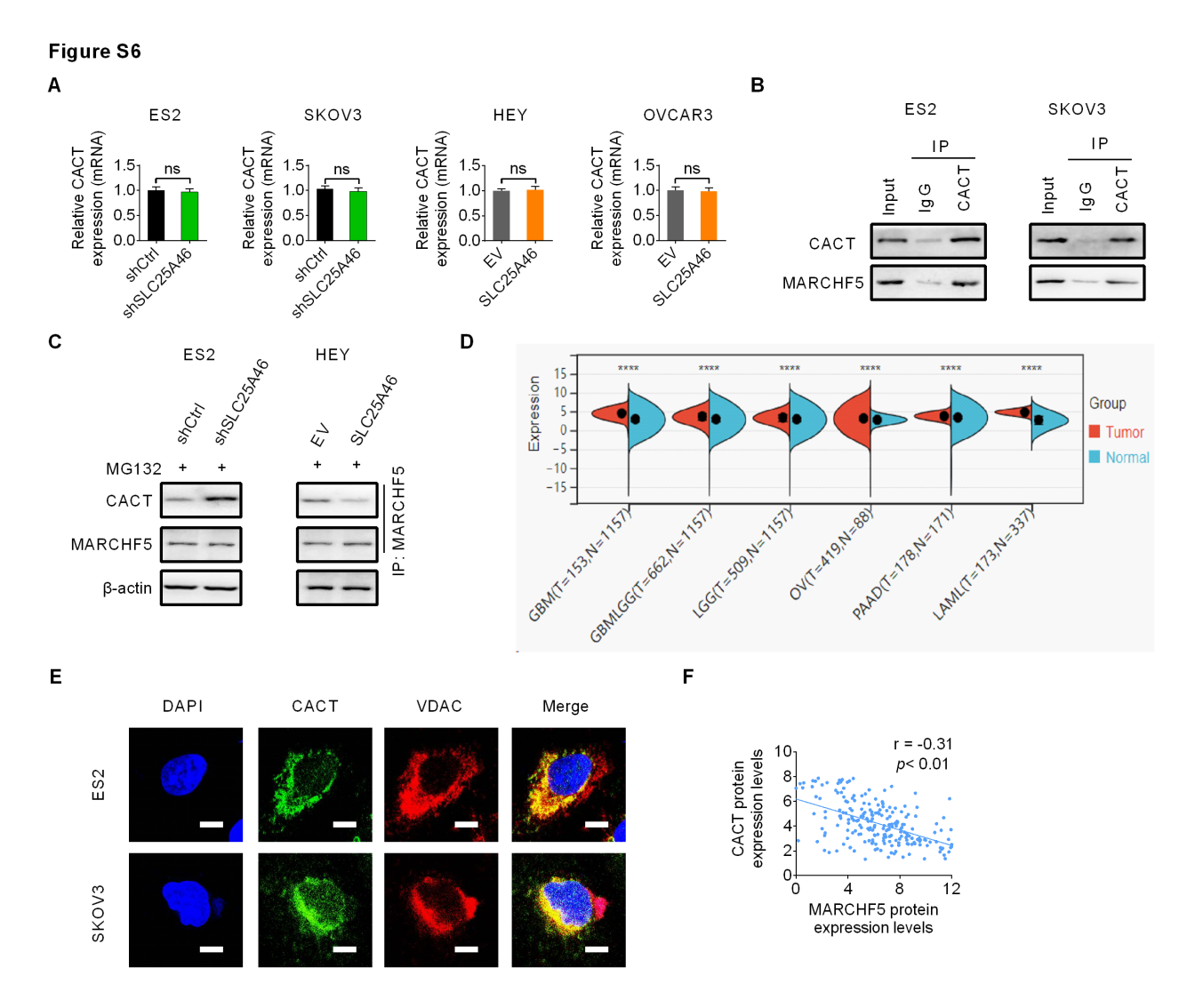
**

**Figure S7. (A-B)** Efficiencies of CACT silencing or overexpression were validated by qRT-PCR (A) and Western blotting (B) assays. **(C)** The levels of Fe^2+^ were evaluated in SLC25A46 silenced OC cells treated with RSL3 (Scale bar = 5 µm). **(D)** The half-maximal inhibitory concentration (IC50) values for carboplatin in ES2 and OVCAR3 cells. **(E)** IC50 values for carboplatin in ES2 and SKOV3 cells with or without SLC25A46 knockdown. **(F)** The levels of Fe^2+^ were evaluated in SLC25A46 silenced OC cells treated with carboplatin (20 μM for 24 hours, Scale bar = 5 µm). **(G-H)** SLC25A46 expression was detected at mRNA level by qRT-PCR (G) and at protein level by western blotting (H) assays in established carboplatin-resistant and -sensitive OC cells.


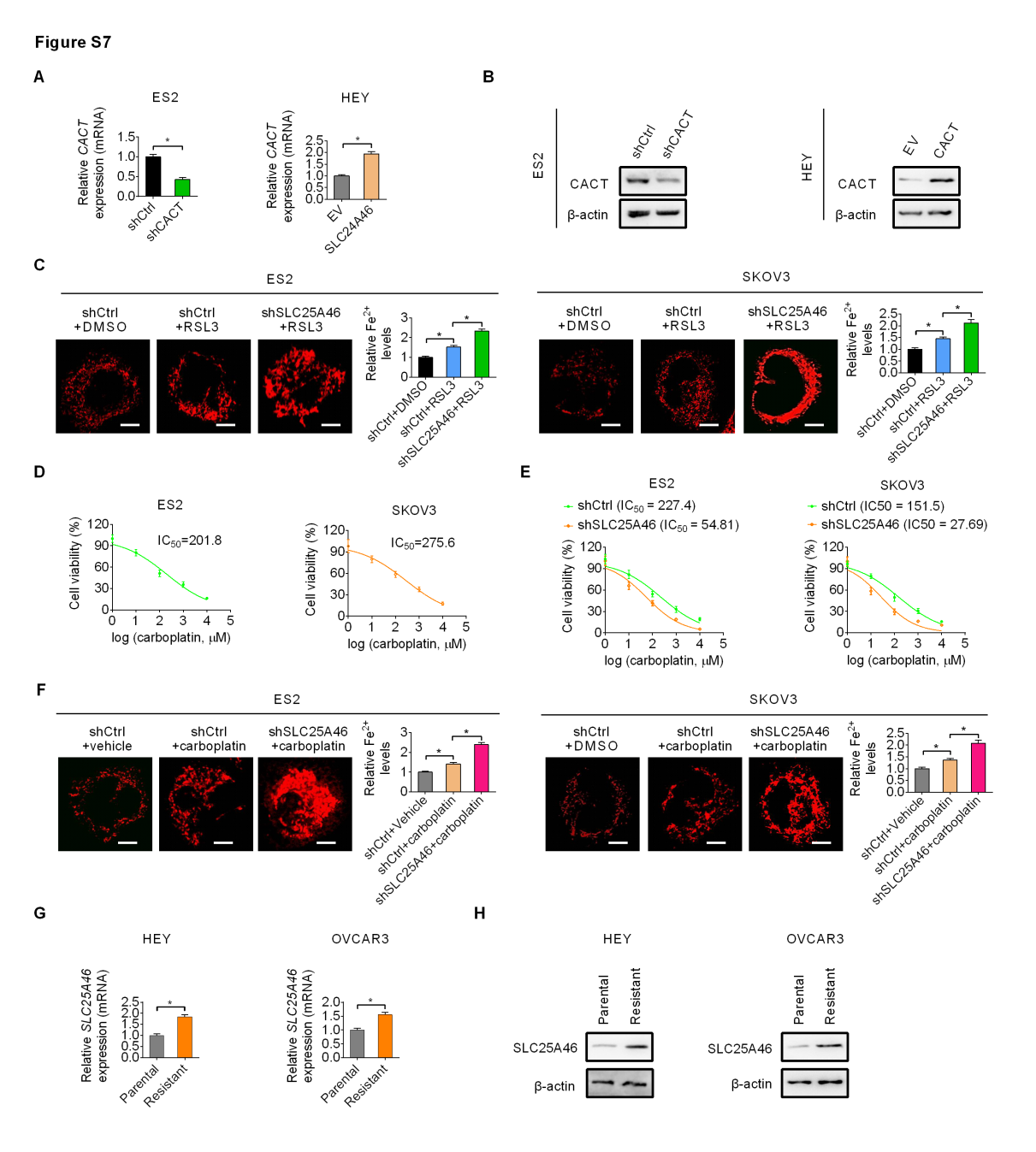


**Figure S8.** Correlations between the expressions of SLC25A46 and indicated transcription factors were analyzed using the online GEPIA database.


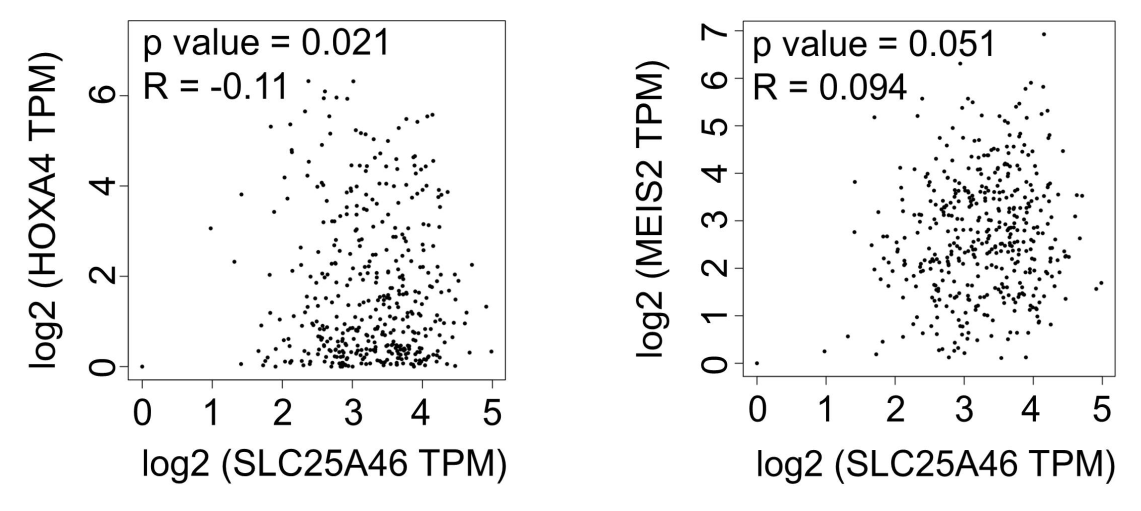


**Supplemental Tables**

**Table S1. Correlation between SLC25A46 expression and clinicopathologic features of OC patients.**

| Clincopathological features | Number  (%) | SLC25A46 expression | | *P* value |
| --- | --- | --- | --- | --- |
|  |  | Low | High |  |
| All | 214 (100%) | 107 | 107 |  |
| Age |  |  |  |  |
| <55 | 131 (61.2 %) | 63 | 68 | 0.575 |
| >=55 | 83 (38.8%) | 44 | 39 |  |
| FIGO |  |  |  |  |
| I-II | 55 | 32 | 23 | 0.159 |
| III-IV | 159 | 75 | 84 |  |
| Tumor size (cm) |  |  |  |  |
| <8 | 95 (44.4%) | 56 | 39 | **0.027** |
| >=8 | 119 (55.6% ) | 51 | 68 |  |
| Lymphatic invasion |  |  |  |  |
| No | 183 (85.5%) | 94 | 89 | 0.438 |
| Yes | 31 (14.5%) | 13 | 18 |  |
| Distant metastasis |  |  |  |  |
| No | 192 (89.7%) | 99 | 93 | 0.260 |
| Yes | 22 (10.3%) | 8 | 14 |  |

**Table S2. The genetic backgrounds of all cell lines used in this study**

| **Cell line** | **TP53** | **BRCA1/2** | **KRAS** | **PIK3CA** |
| --- | --- | --- | --- | --- |
| A2780 | wild-type | wild-type | wild-type | mutant (E365K) |
| ES2 | mutant (S241F) | wild-type | wild-type | wild-type |
| HEY | wild-type | wild-type | mutant (G12D) | wild-type |
| OVCAR3 | mutant (R248Q) | wild-type | wild-type | wild-type |
| SKOV3 | Null | wild-type | wild-type | mutant (H1047R) |
| IOSE-80 | wild-type | wild-type | wild-type | wild-type |

**Table S3. Primers used in this study**

| *SLC25A46* | forward primer | CGGAGGAACCCTTTTCCAGTG |
| --- | --- | --- |
|  | reverse primer | ATGCAAGGATGTGCCAGTACA |
| *MARCHF5* | forward primer | GTCGGCTCTATCTATTGGACAGC |
|  | reverse primer | GCTCTCTCCATAACATCCAGACC |
| *CACT* | forward primer | GGGGTCACTCCCATGTTTG |
|  | reverse primer | TGTGGTGAATACGCCAGATAAC |
| *PBX1* | forward primer | GACAACTCAGTGGAGCATTCA |
|  | reverse primer | CTCTCGCAGGAGATTCATCAC |
| *ChIP-PCR* | forward primer | AGCGGAGCCTAGTCCCTTCT |
|  | reverse primer | CGGCGTCTCGTAGGTGATTT |
| *β-actin* | forward primer | CACCATTGGCAATGAGCGGTTC |
|  | reverse primer | AGGTCTTTGCGGATGTCCACGT |

**Table S4. Primary antibodies used in this study.**

| **Antibody** | **Company (Cat. No.)** | **Working dilutions** |
| --- | --- | --- |
| SLC25A46 | Santa Cruz (sc-515823) | WB:1/1000; IF:1/200;  IP: 1/200 |
|  | Proteintech (12277-1-AP) | IHC: 1/500 |
| CACT | Proteintech (19363-1-AP) | WB: 1/1000; IHC: 1/500 |
|  | Bioma (CAU25538) | IF:1/200; IP: 1/200 |
| MARCH5 | Proteintech (12213-1-AP) | WB: 1/1000; IHC: 1/300 |
|  | Cell Signaling (19168S) | IP: 1/200; IF: 1/200 |
| 4-HNE | Proteintech (68538-1-Ig) | IHC:1/500 |
| VDAC | Proteintech (10866-1-AP) | WB: 1/1000 |
| MDA | Abcam (ab27642) | IHC, 1/300 |
| PBX1 | Proteintech (18204-1-Ig) | WB: 1/1000; IHC: 1/500 |
| ki-67 | Proteintech (27309-1-AP) | IHC:1/600 |
| SOD2 | Proteintech (24127-1-AP) | WB: 1/1000 |
| CAT | Proteintech (21260-1-AP) | WB: 1/1000 |
| GPX1 | Proteintech (29329-1-AP) | WB: 1/1000 |
| GPX4 | Proteintech (67763-1-ig) | WB: 1/1000 |
| ACSL4 | Proteintech (22401-1-AP) | WB: 1/1000 |
| β-actin | Proteintech (20536-1-AP) | WB: 1/2000 |
