# Supplementary material for: Mitochondrial SLC25A46 Rewires Fatty Acid Oxidation to Promote Cell Proliferation and Ferroptosis Evasion in Ovarian Cancer by Stabilizing CACT
Source: Adv Sci (Weinh). 2026 Jul 20:e23969. Online ahead of print. doi: 10.1002/advs.202523969 (PMC13383691; doi:10.1002/advs.202523969)
Supplement: Supplementary file 2 — Supporting File 2: advs76689‐sup‐0002‐original_ blots_ images.pdf. [file ADVS-9999-e23969-s001.pdf]

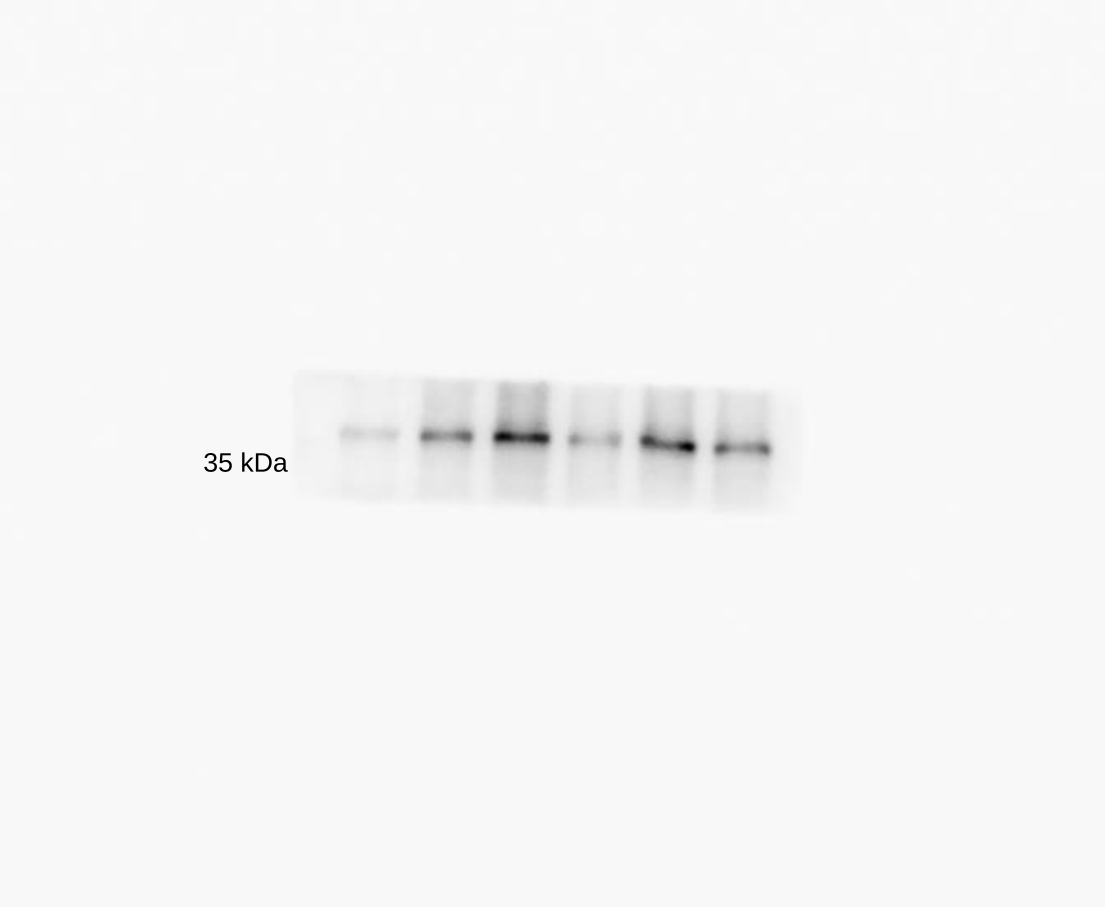

Fig 1H

IB: SLC25A46

Groups: IOSE80, A2780, ES2, HEY, OVCAR3, SKOV3 (whole cell lysate)

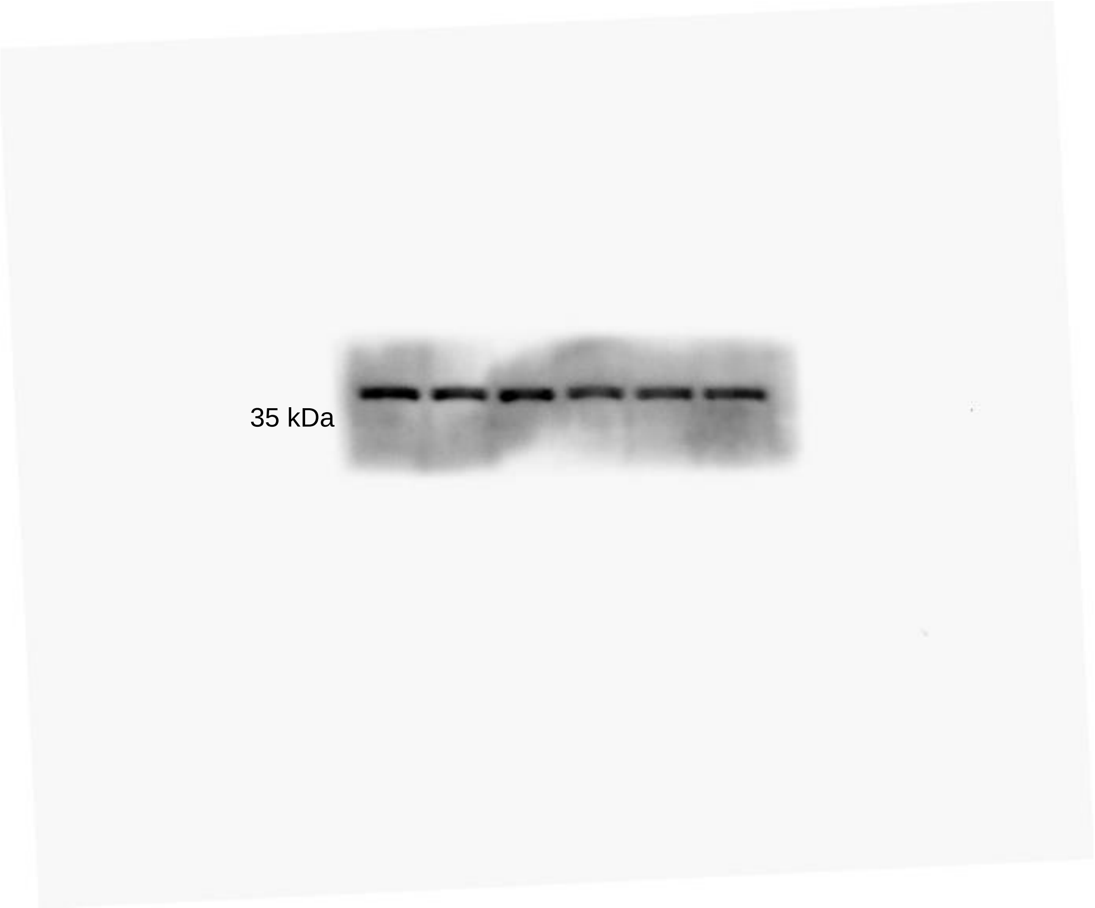

Fig 1H

IB: β-actin

Groups: IOSE80, A2780, ES2, HEY, OVCAR3, SKOV3 (whole cell lysate)

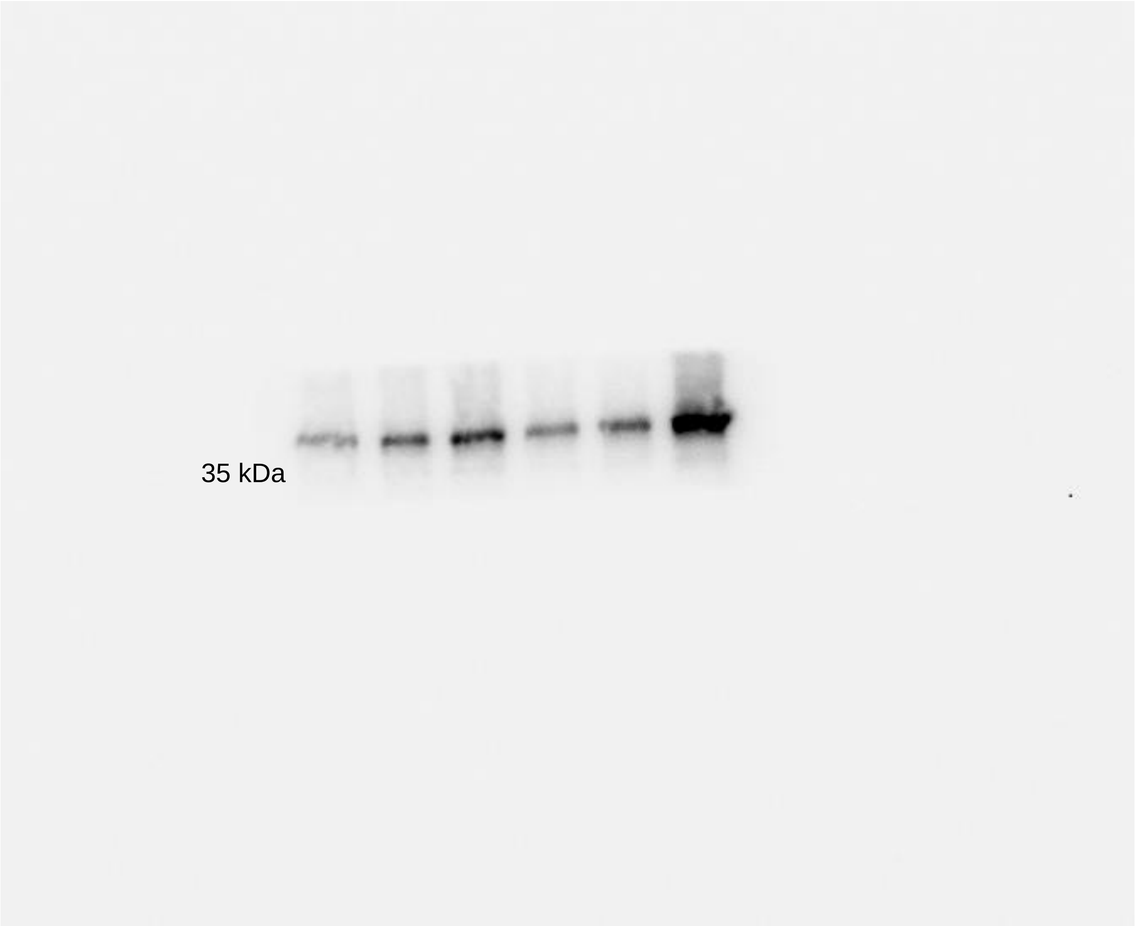

Fig 1H

IB: SLC25A46

Groups: IOSE80, A2780, ES2, HEY, OVCAR3, SKOV3 (mitochondrial fraction)

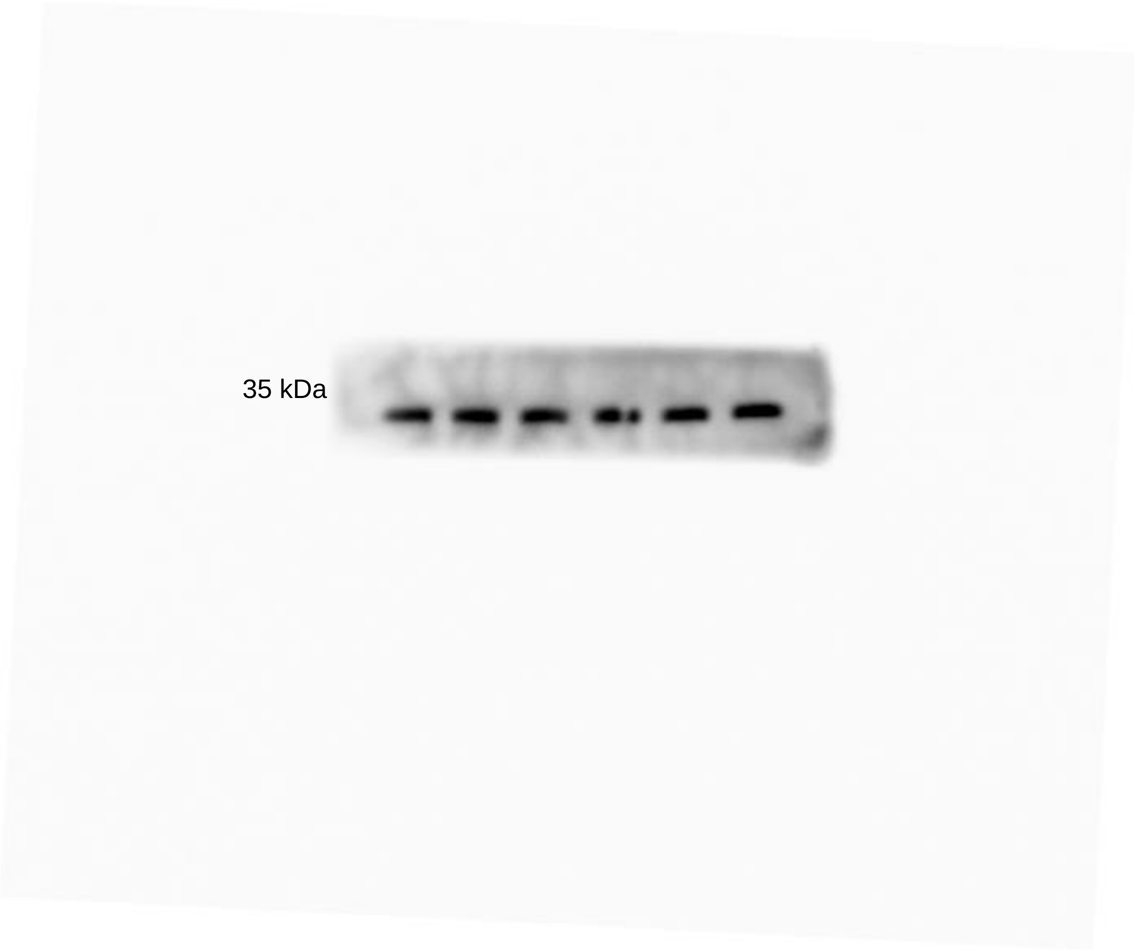

Fig 1H

IB: VDAC

Groups: IOSE80, A2780, ES2, HEY, OVCAR3, SKOV3 (mitochondrial fraction)

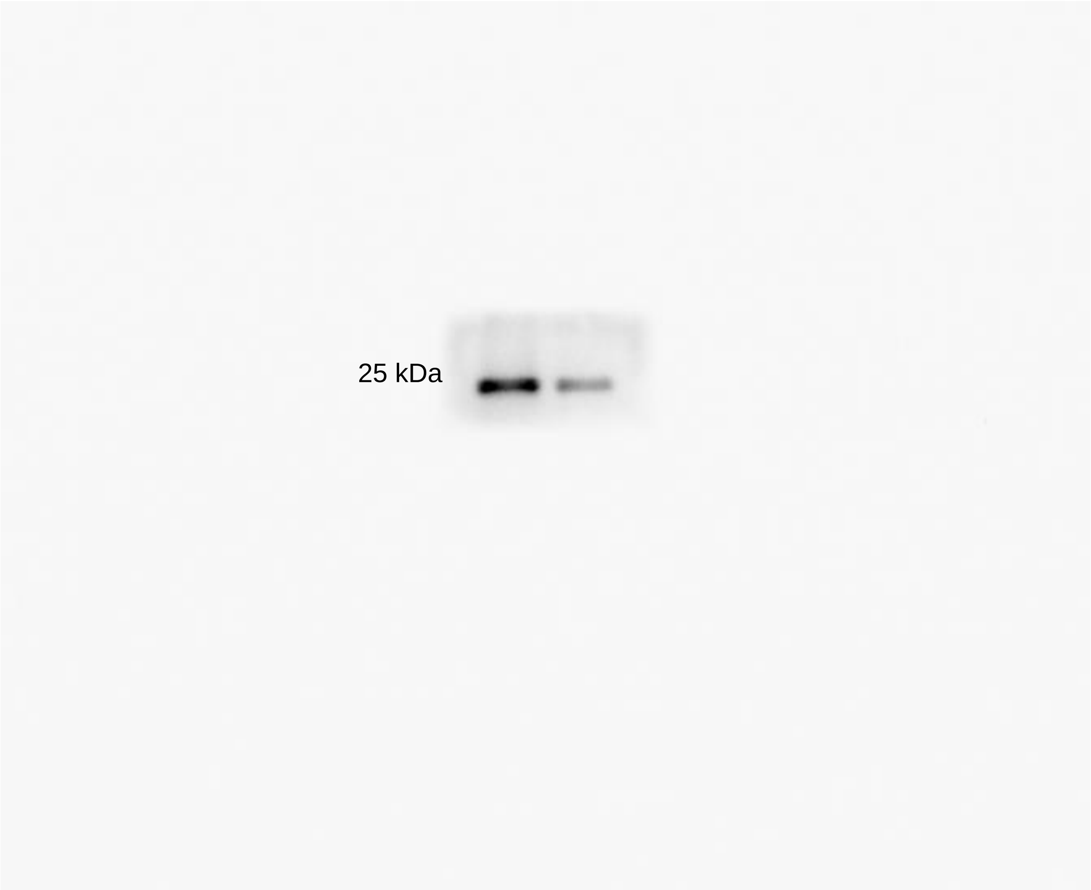

Fig 3H

IB: GPX4

Groups (ES2 cells): shCtrl, shSLC25A46

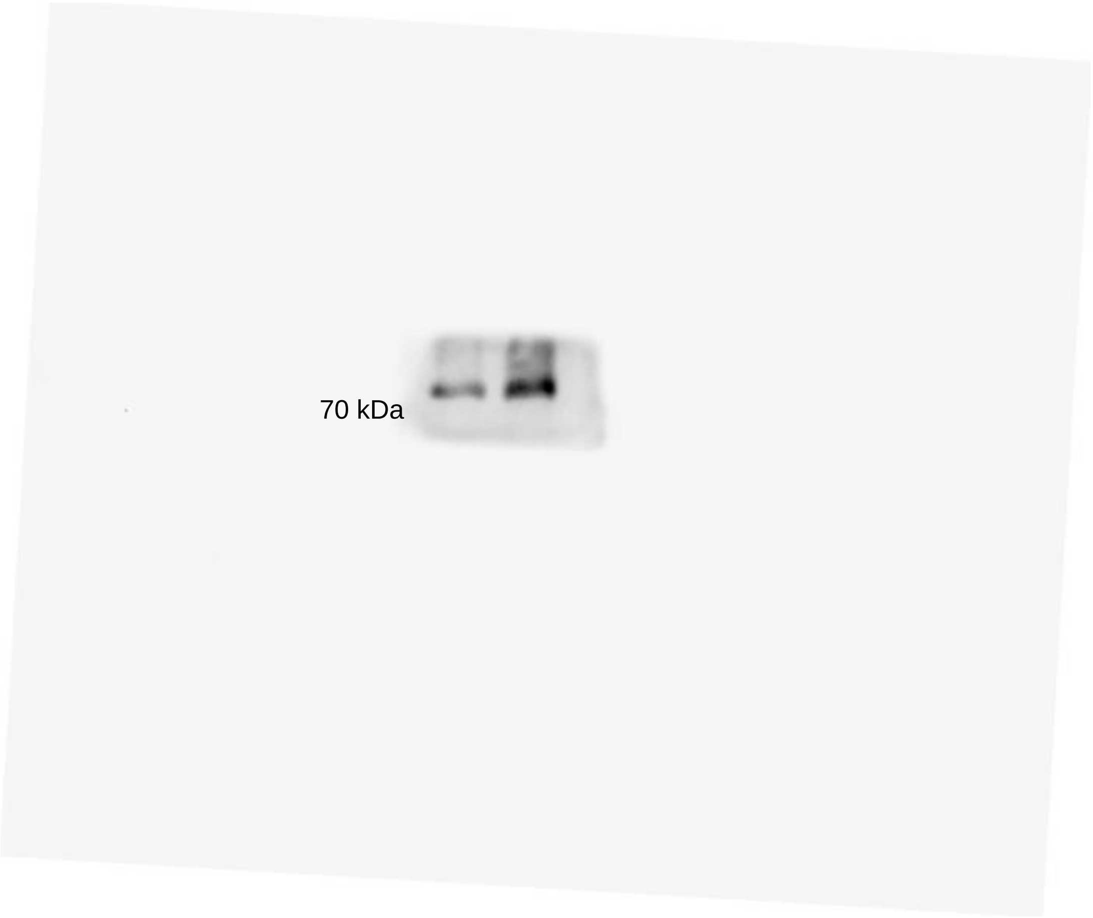

Fig 3H

IB: ACSL4

Groups (ES2 cells): shCtrl, shSLC25A46

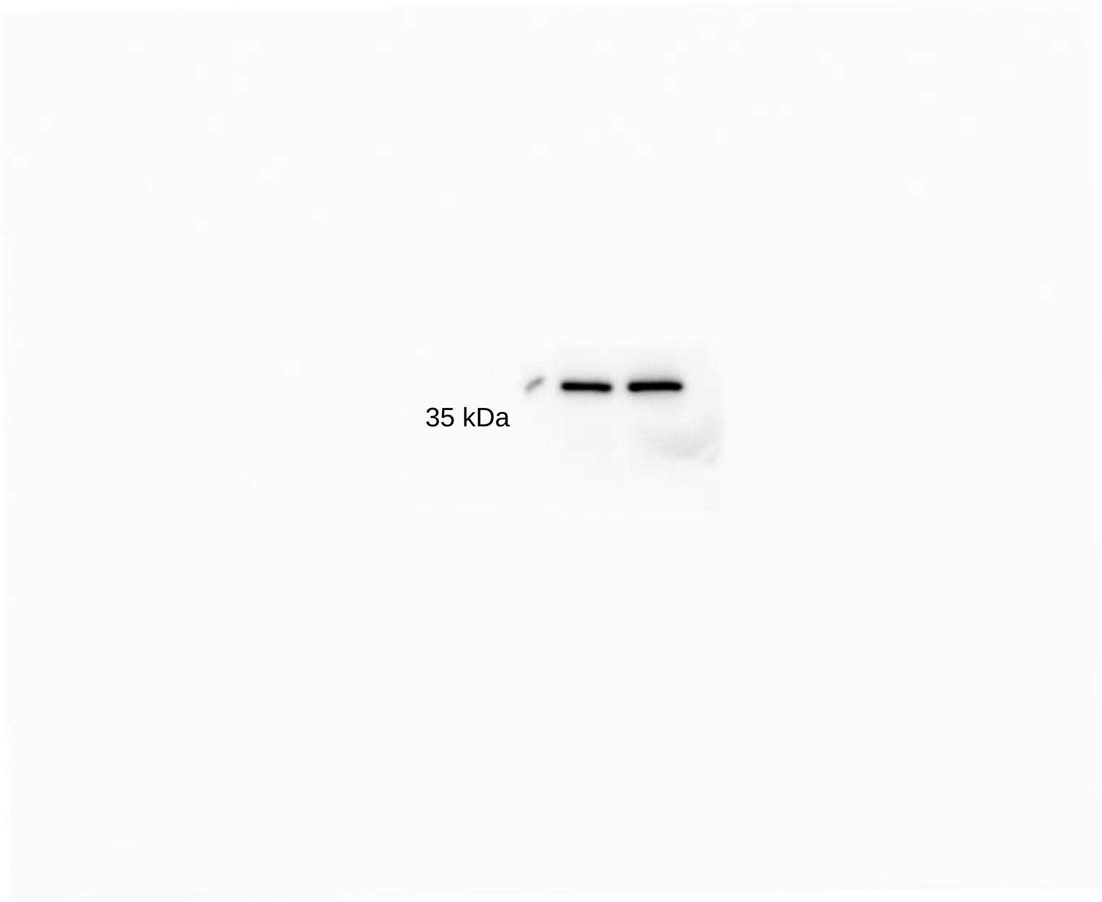

Fig 3H

IB: β-actin

Groups (ES2 cells): shCtrl, shSLC25A46

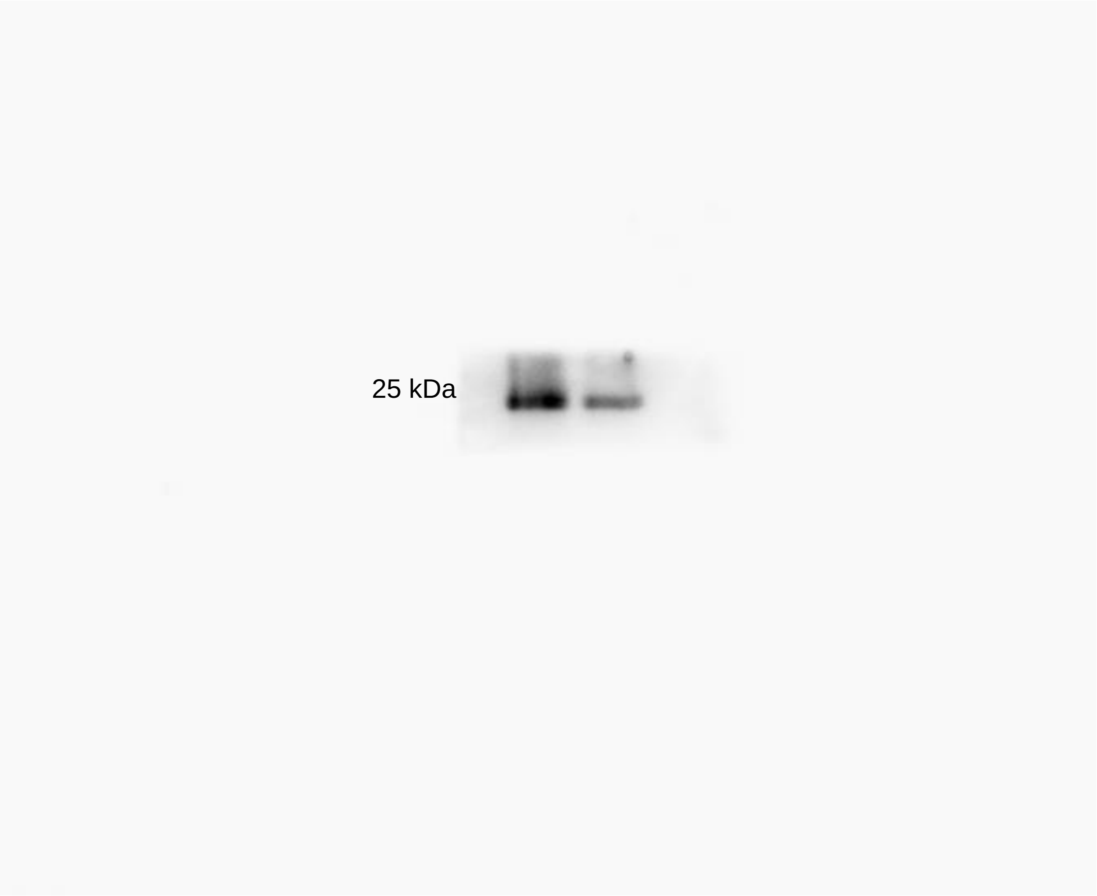

Fig 3H

IB: GPX4

Groups (HEY cells): EV, SLC25A46

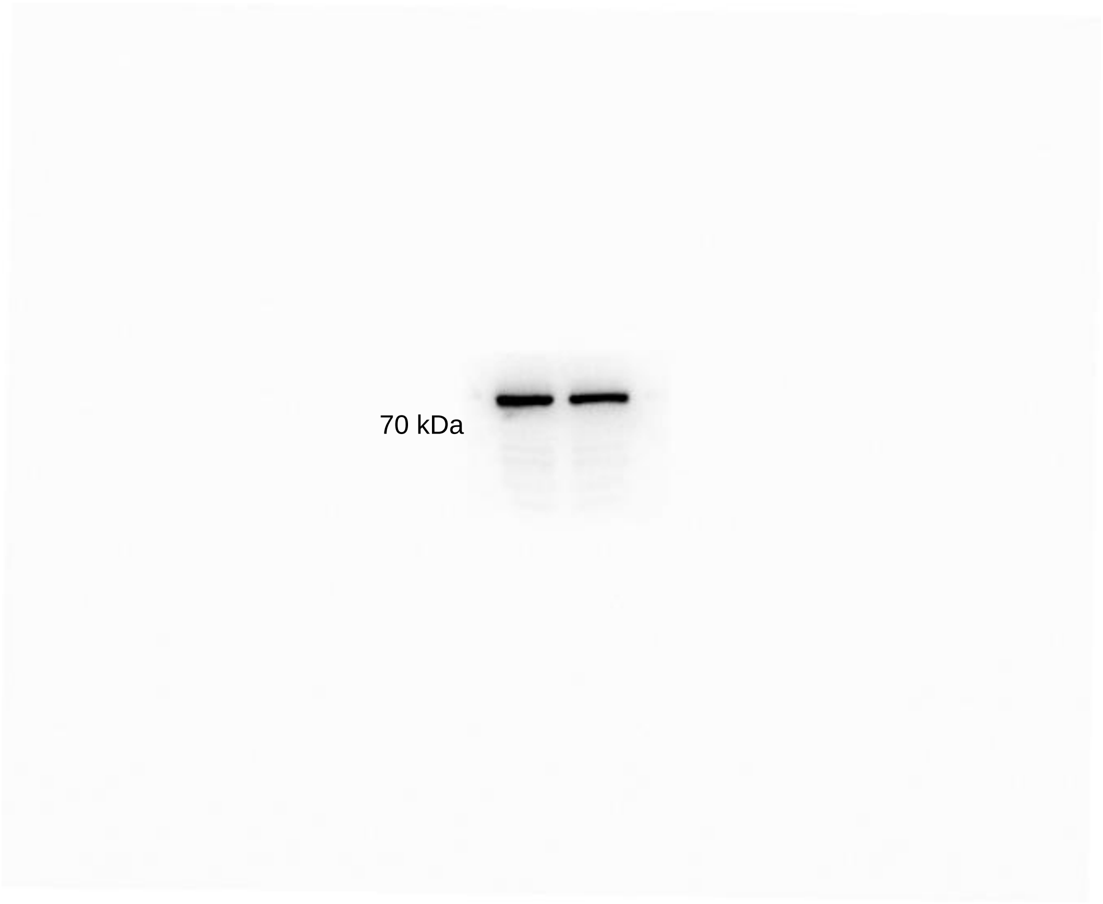

Fig 3H

IB: ACSL4

Groups (HEY cells): EV, SLC25A46

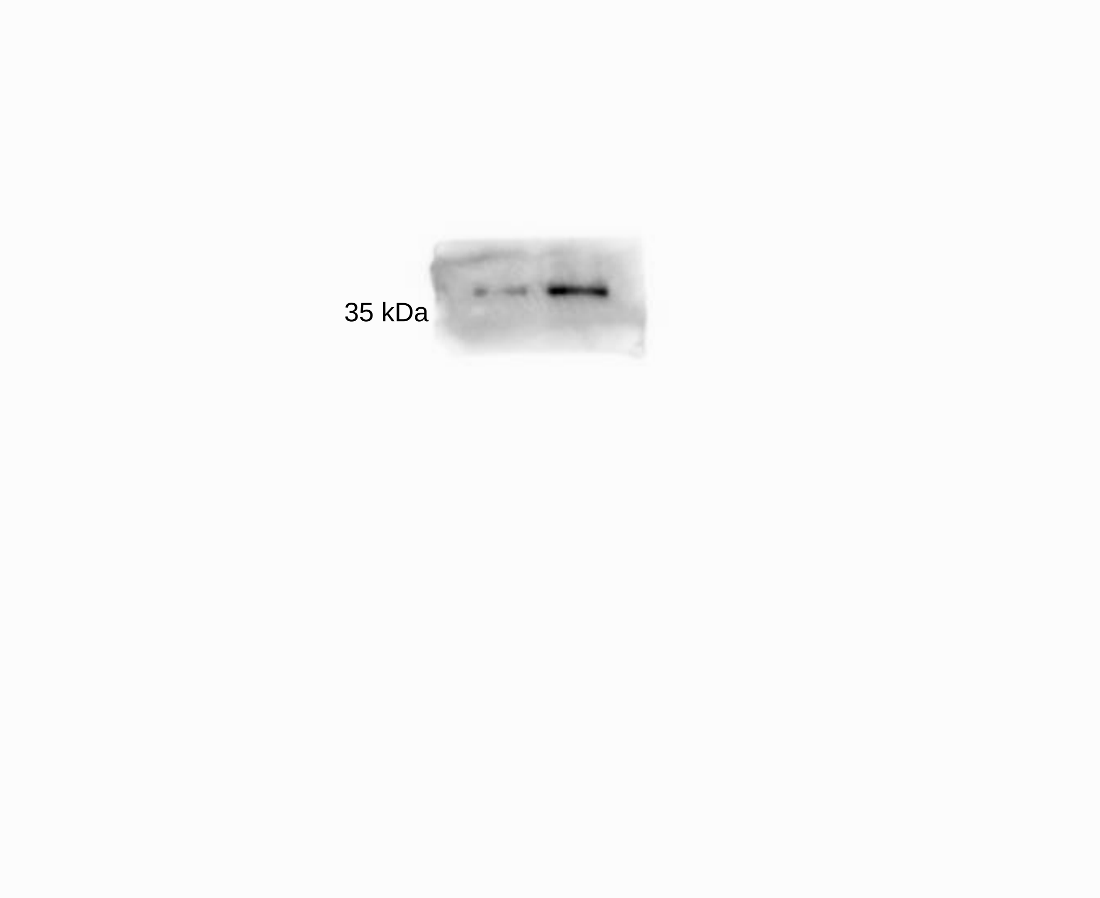

Fig 3H

IB: β-actin

Groups (HEY cells): EV, SLC25SA46

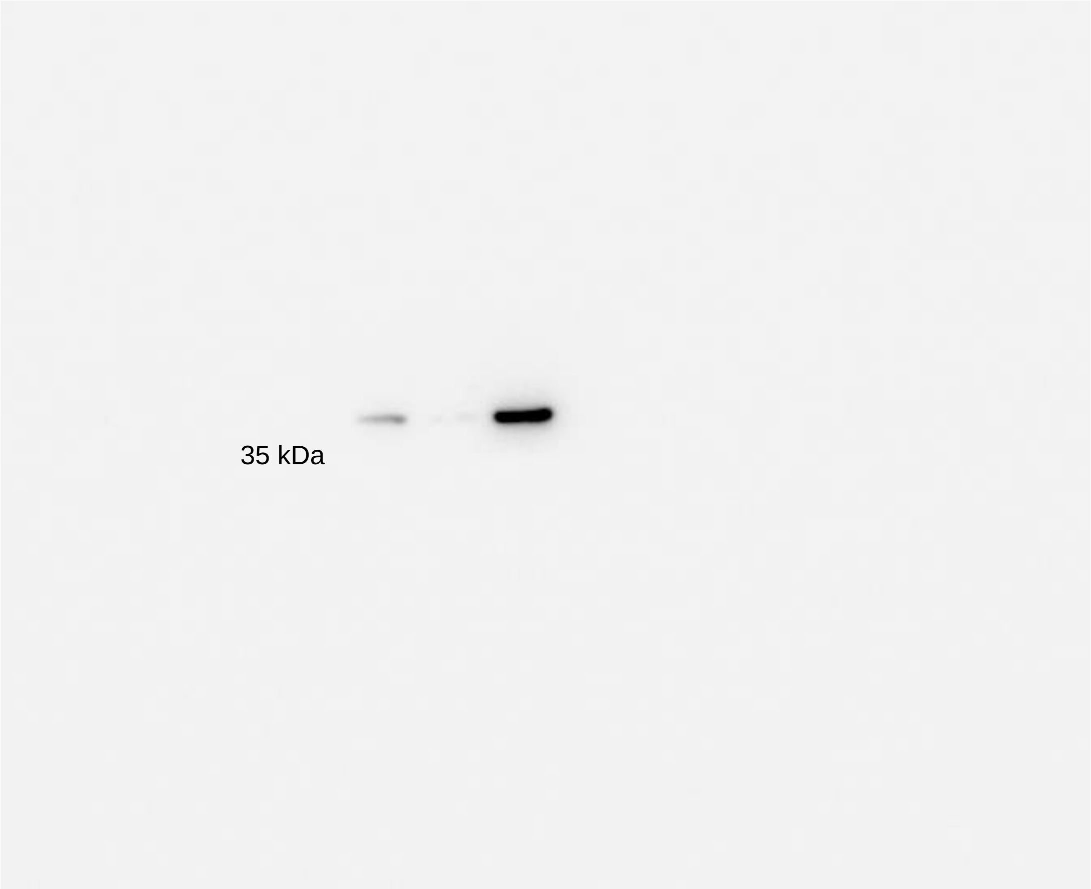

Fig 5B

IB: SLC25A46

Groups (ES2 cells): Input, IgG (IP), SLC25A46 (IP)

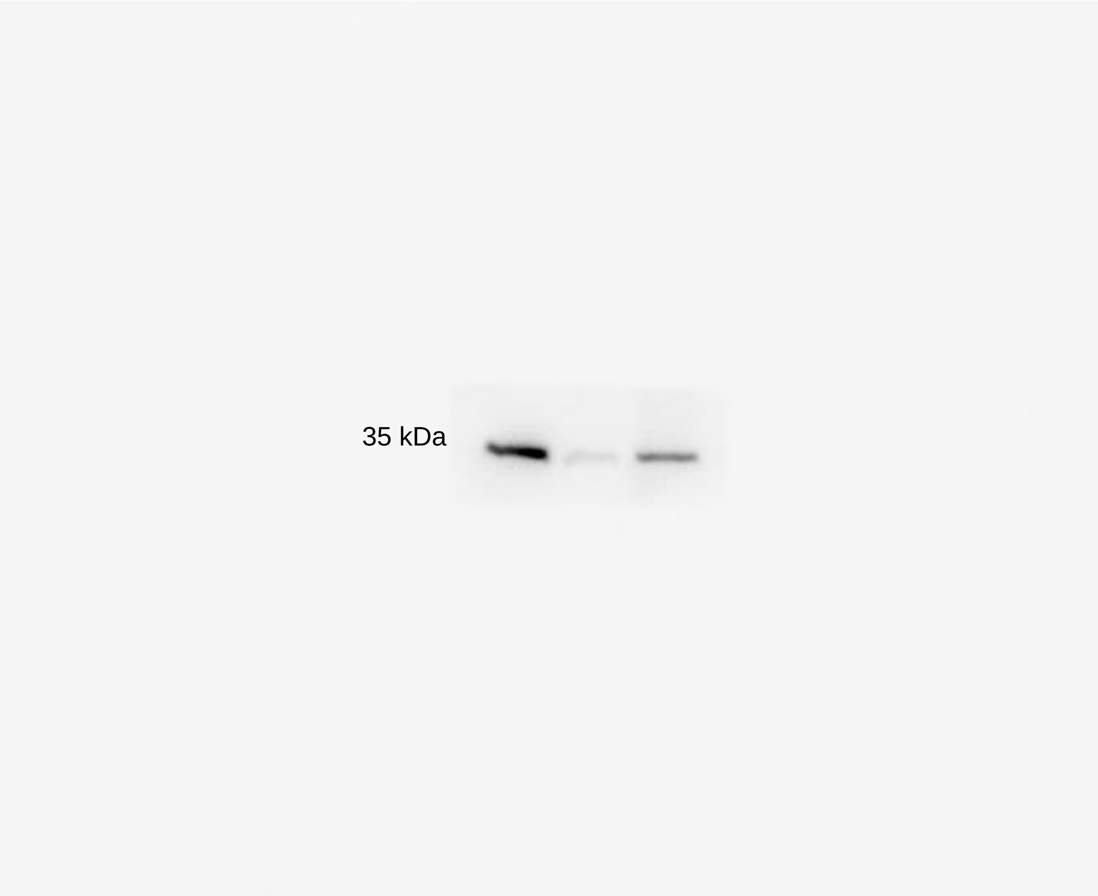

Fig 5B

IB: CACT

Groups (ES2 cells): Input, IgG (IP), SLC25A46 (IP)

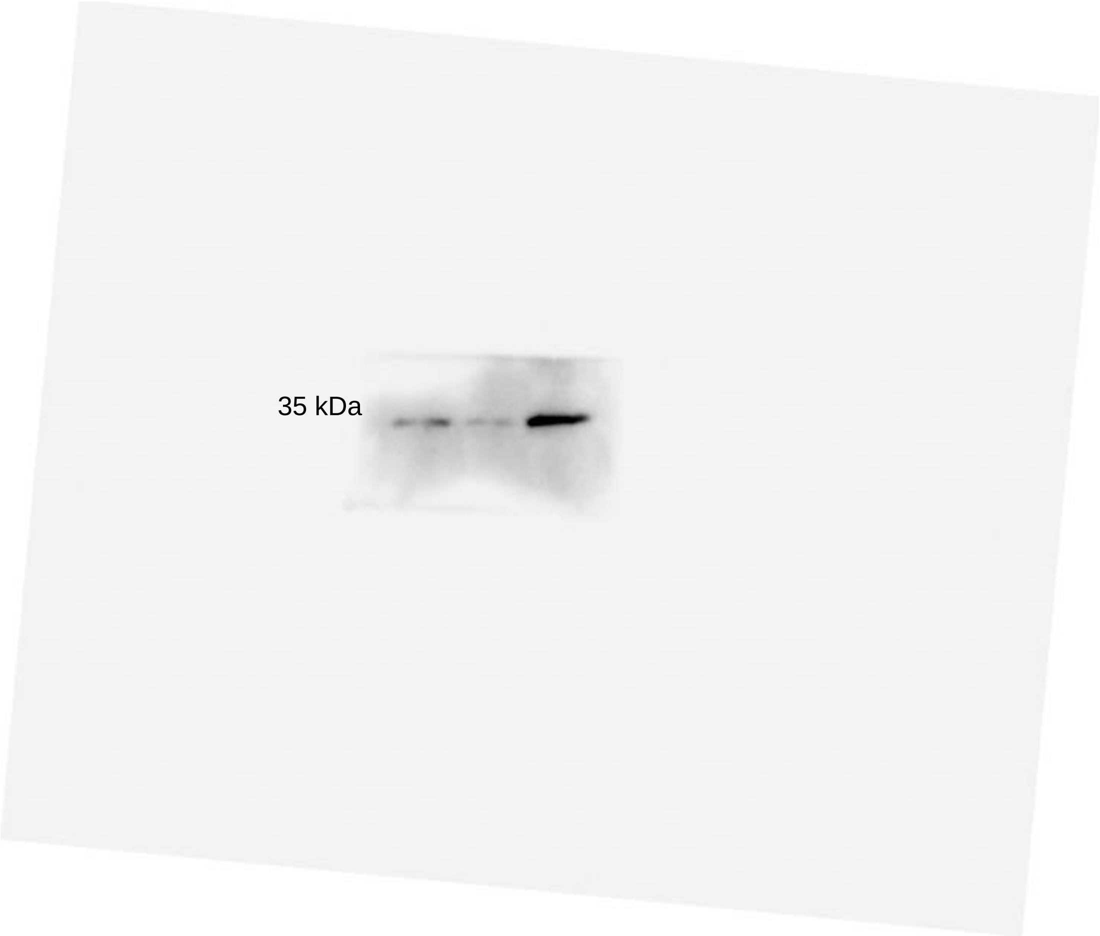

Fig 5B

IB: CACT

Groups (ES2 cells): Input, IgG (IP), CACT (IP)

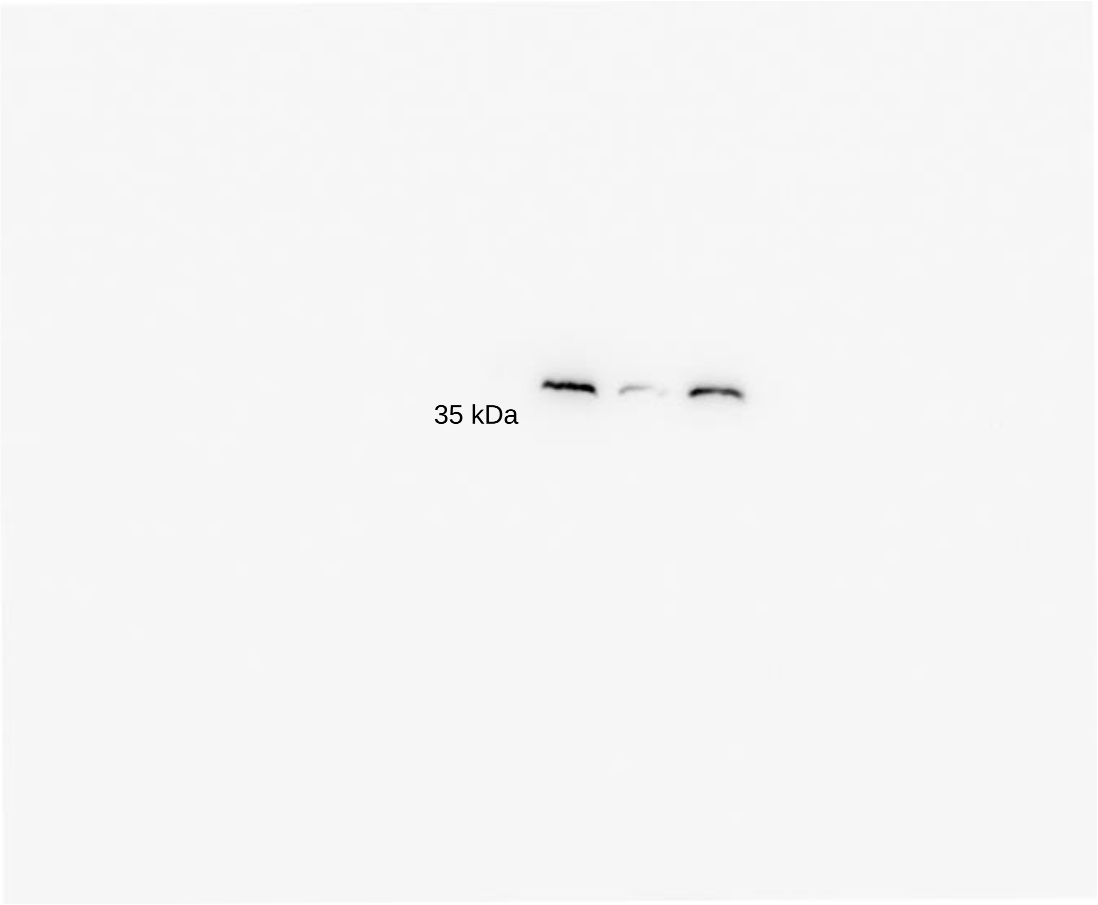

Fig 5B

IB: SLC25A46

Groups (ES2 cells): Input, IgG (IP), CACT (IP)

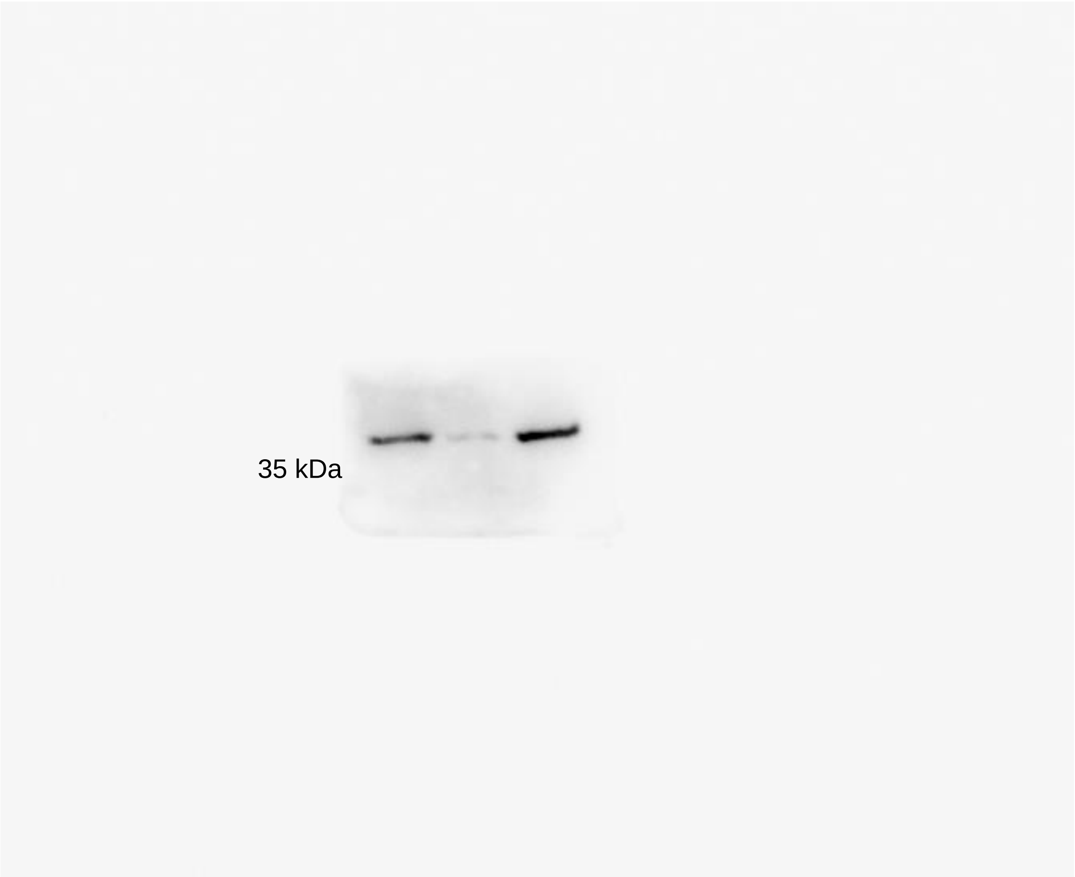

Fig 5B

IB: SLC25A46

Groups (SKOV3 cells): Input, IgG (IP), SLC25A46 (IP)

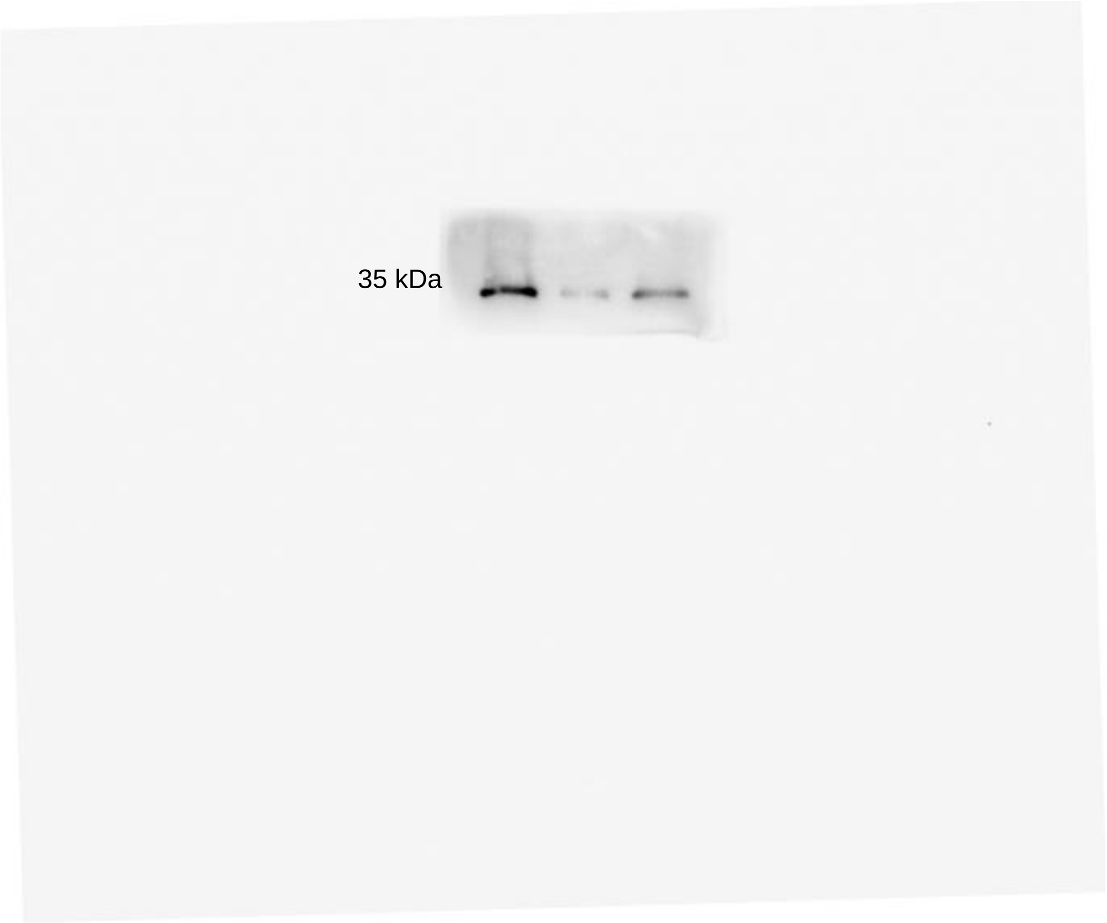

Fig 5B

IB: CACT

Groups (SKOV3 cells): Input, IgG (IP), SLC25A46 (IP)

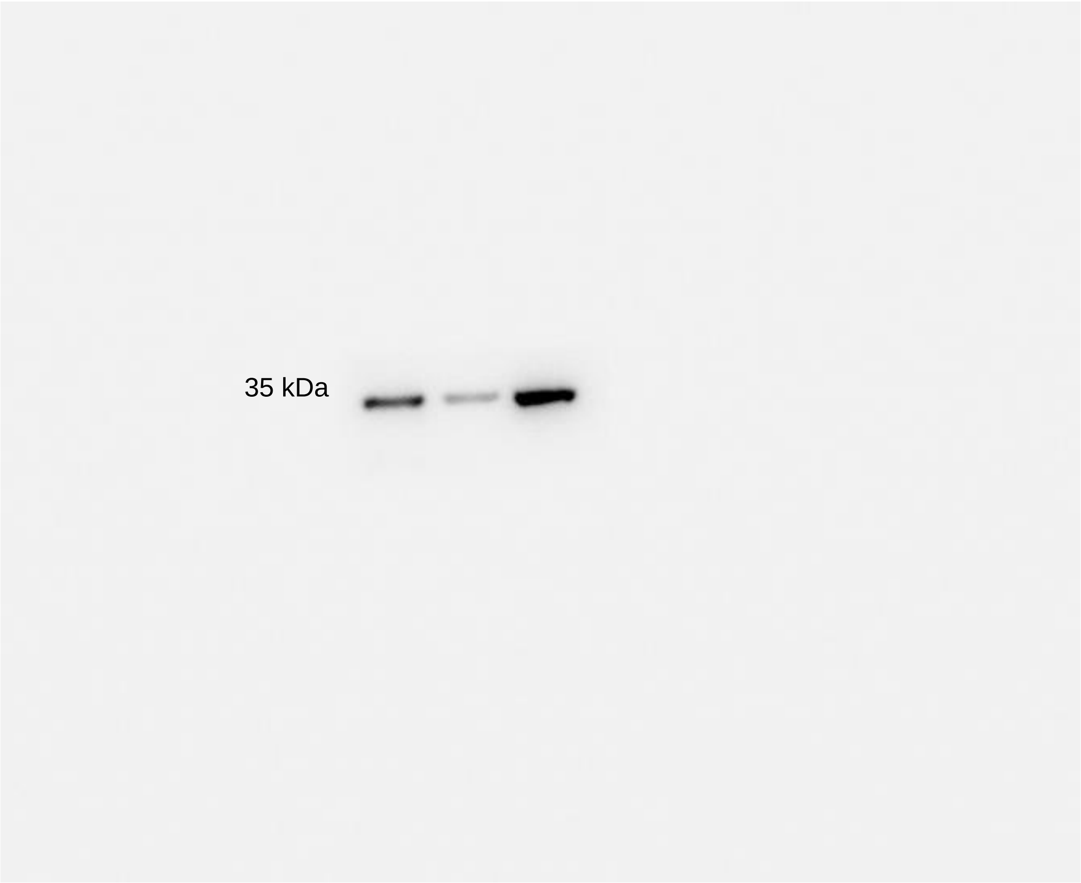

Fig 5B

IB: CACT

Groups (SKOV3 cells): Input, IgG (IP), CACT (IP)

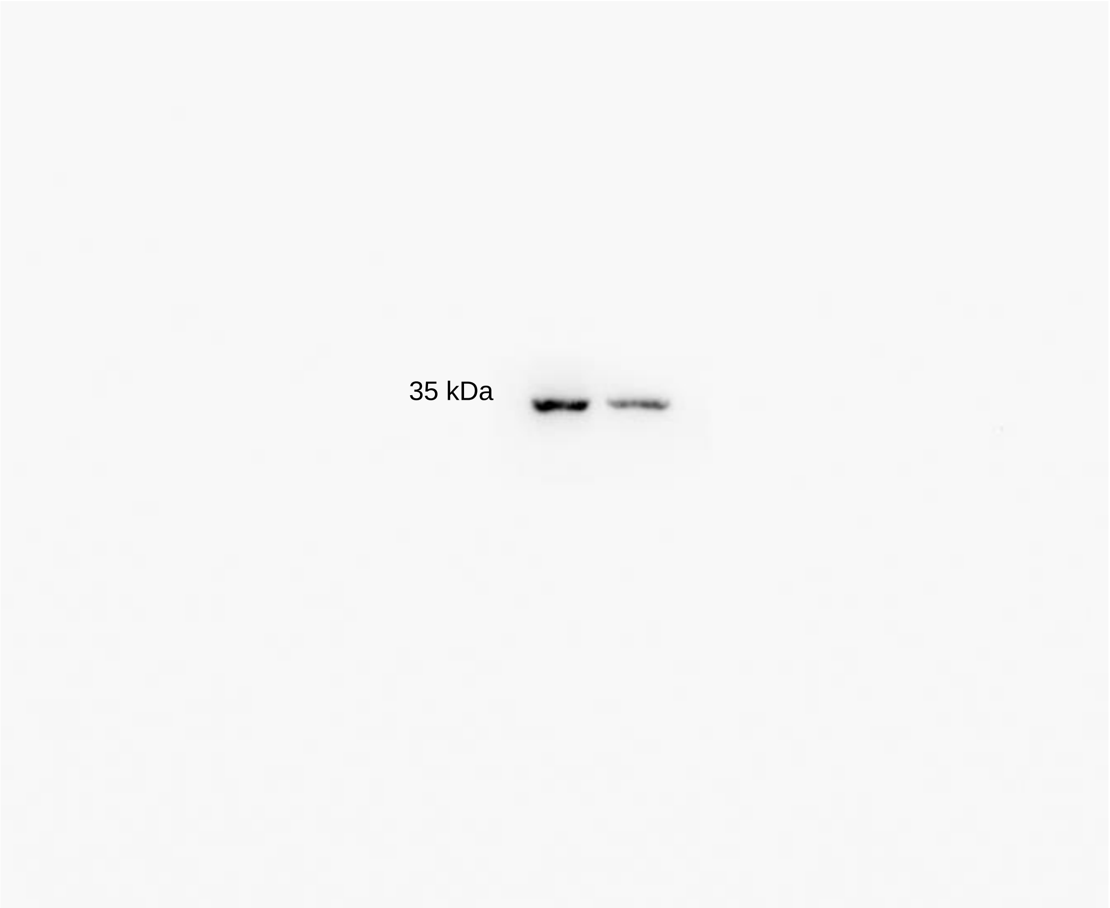

Fig 5D

IB: CACT

Groups (ES2 cells): shCtrl, shSLC25A46

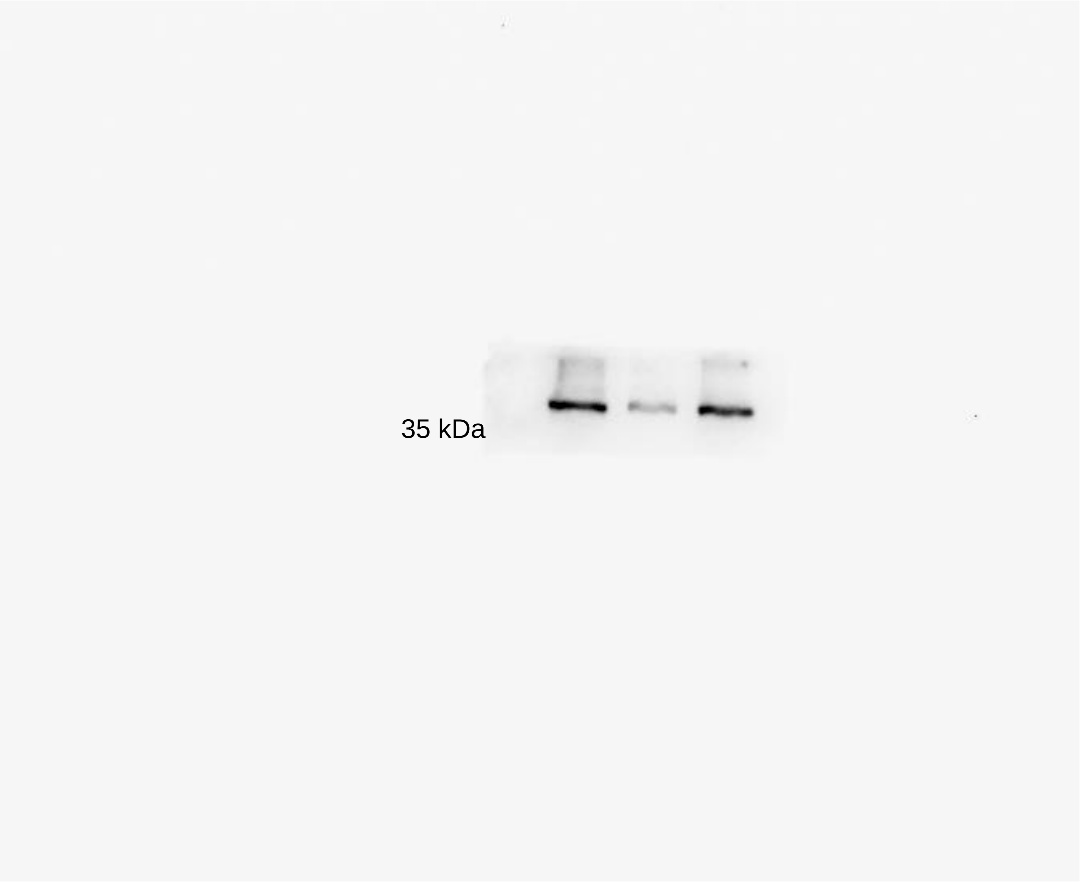

Fig 5B

IB: SLC25A46

Groups (SKOV3 cells): Input, IgG (IP), CACT (IP)

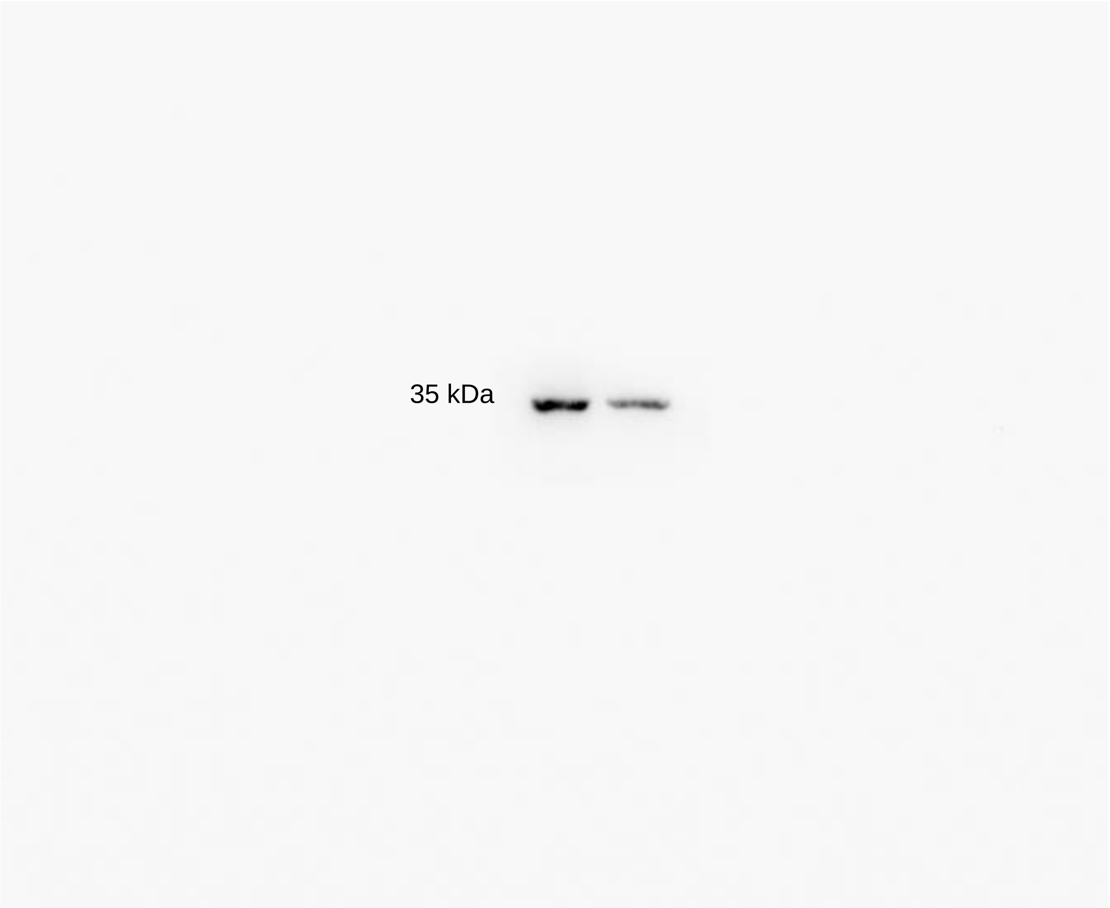

Fig 5D

IB: CACT

Groups (ES2 cells): shCtrl, shSLC25A46

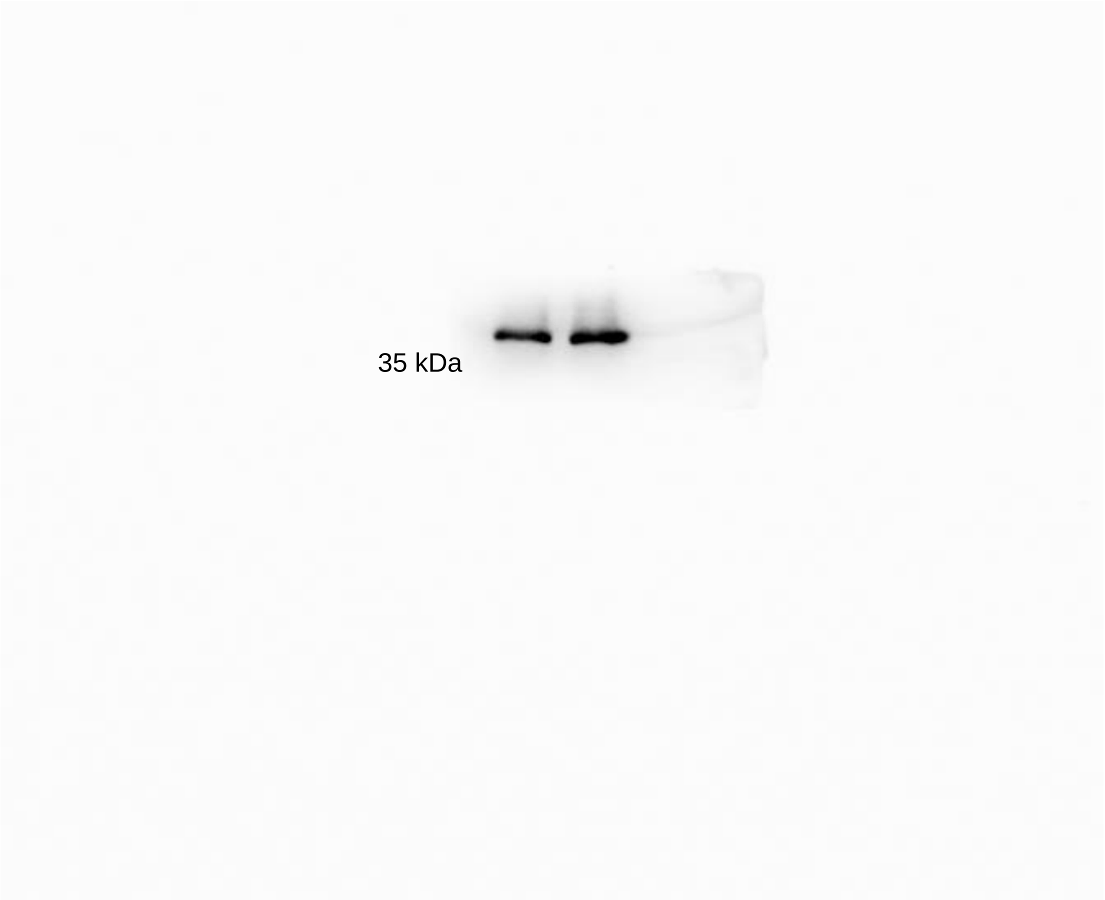

Fig 5D

IB: β-actin

Groups (ES2 cells): shCtrl, shSLC25A46

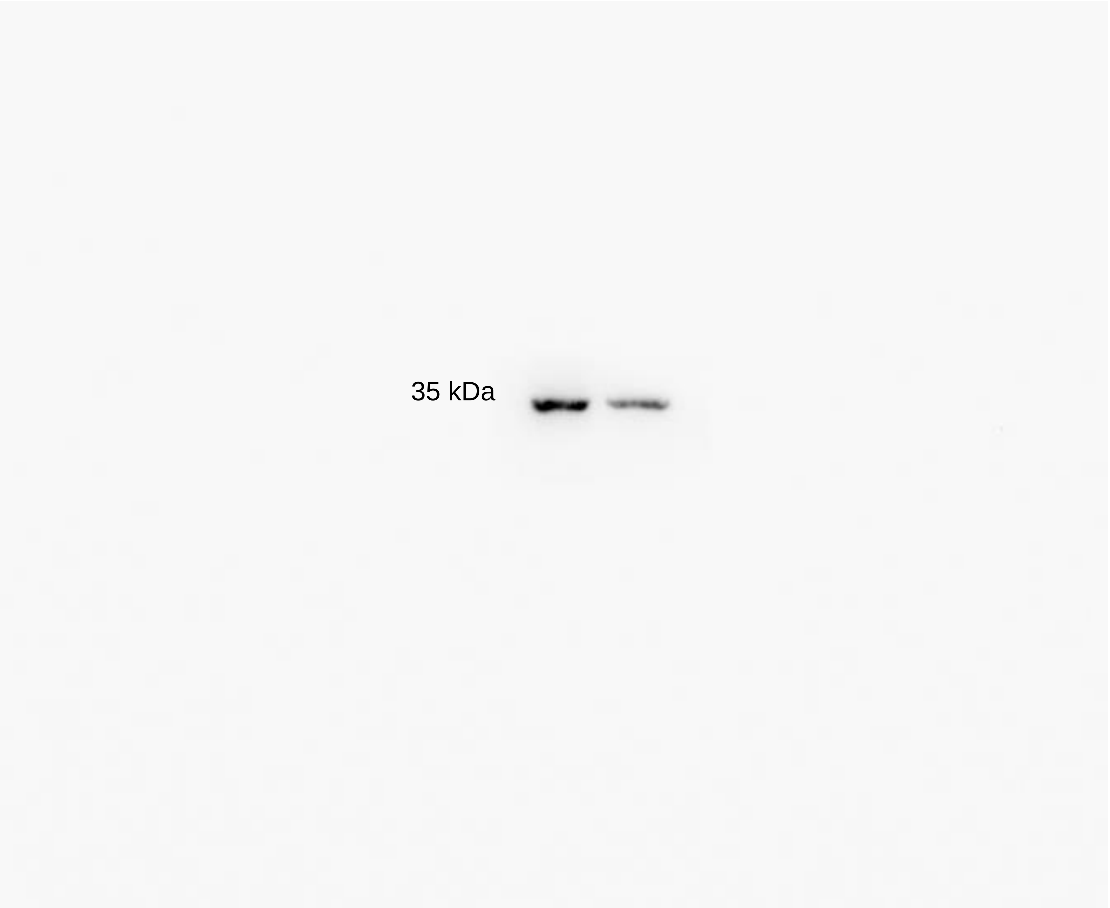

Fig 5D

IB: CACT

Groups (SKOV3 cells): shCtrl, shSLC25A46

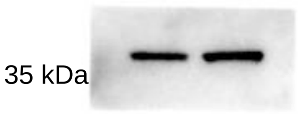

Fig 5D

IB: β-actin

Groups (SKOV3 cells): shCtrl, shSLC25A46

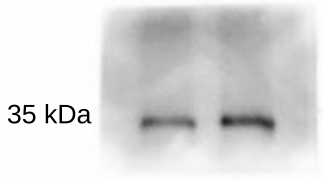

Fig 5D

IB: CACT

Groups (HEY cells): EV, SLC25A46

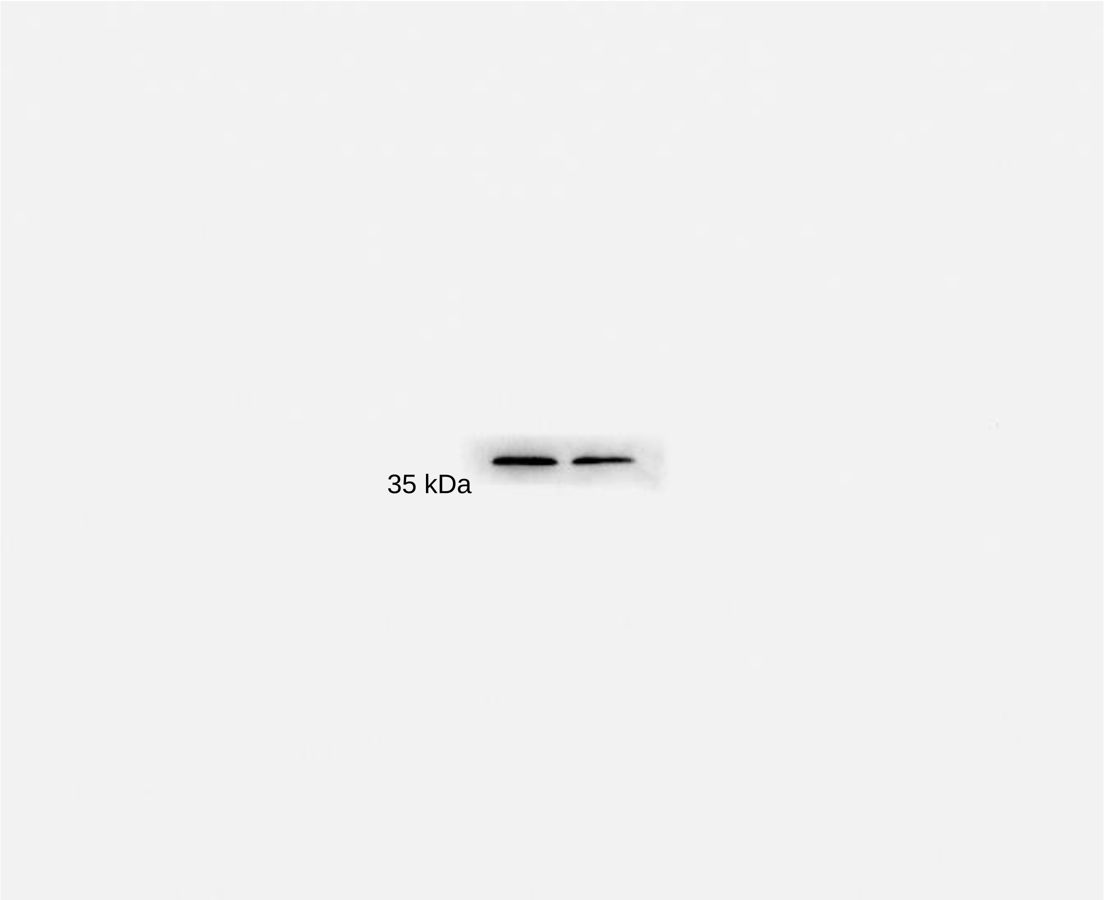

Fig 5D

IB: β-actin

Groups (HEY cells): EV, SLC25A46

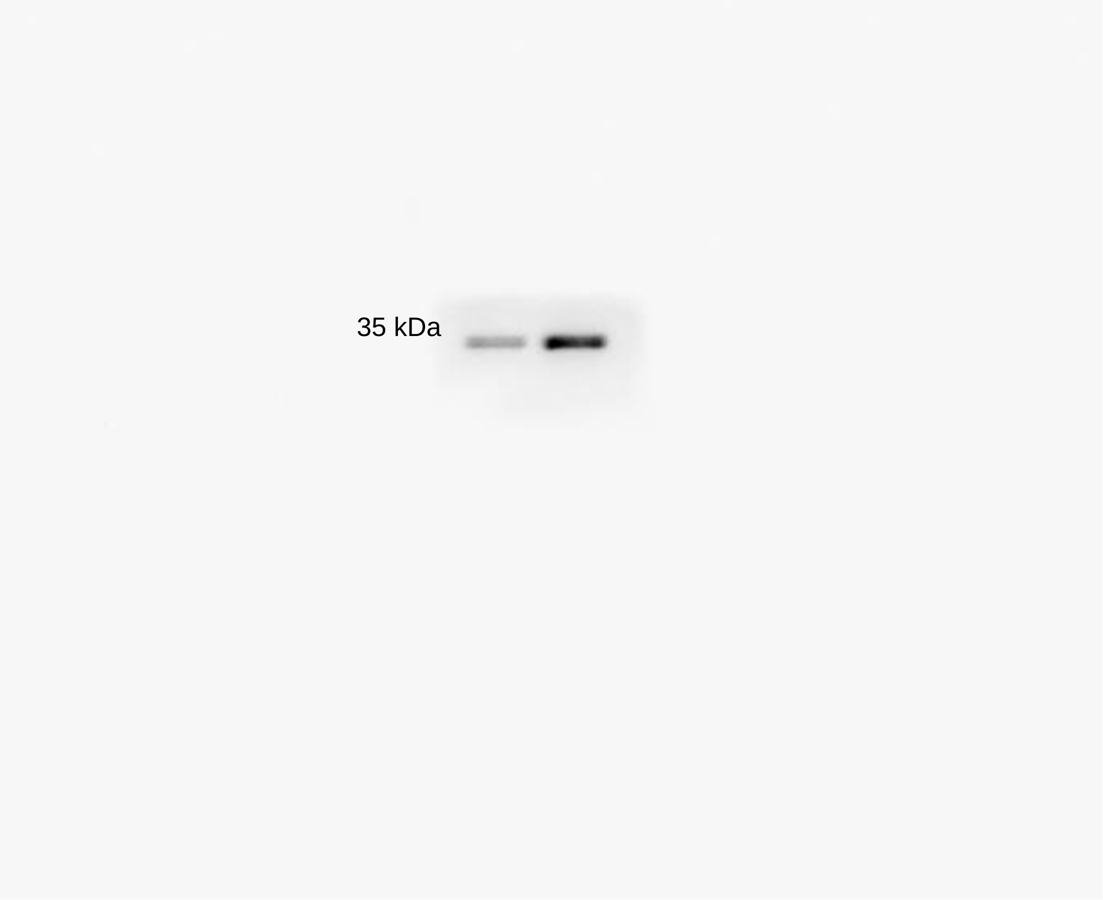

Fig 5D

IB: CACT

Groups (OVCAR3 cells): EV, SLC25A46

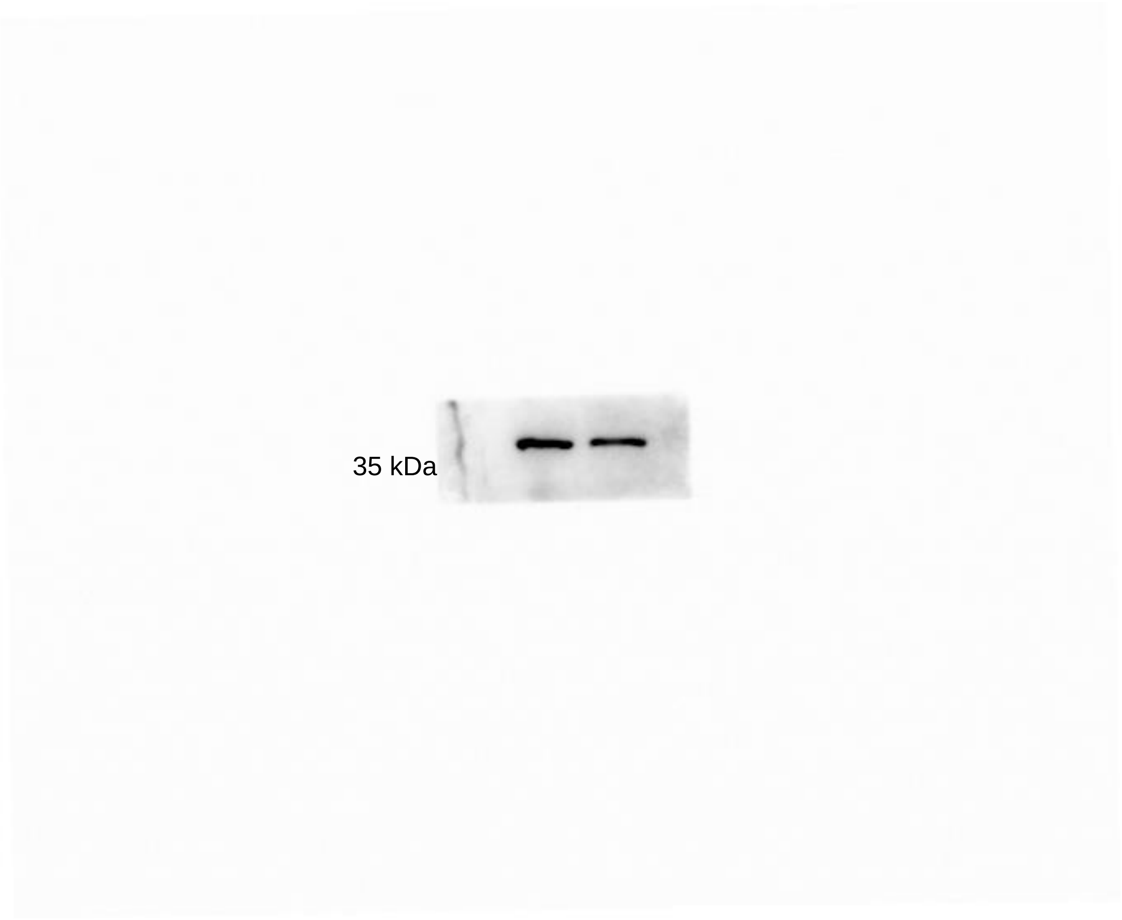

Fig 5D

IB: β-actin

Groups (OVCAR3 cells): EV, SLC25A46

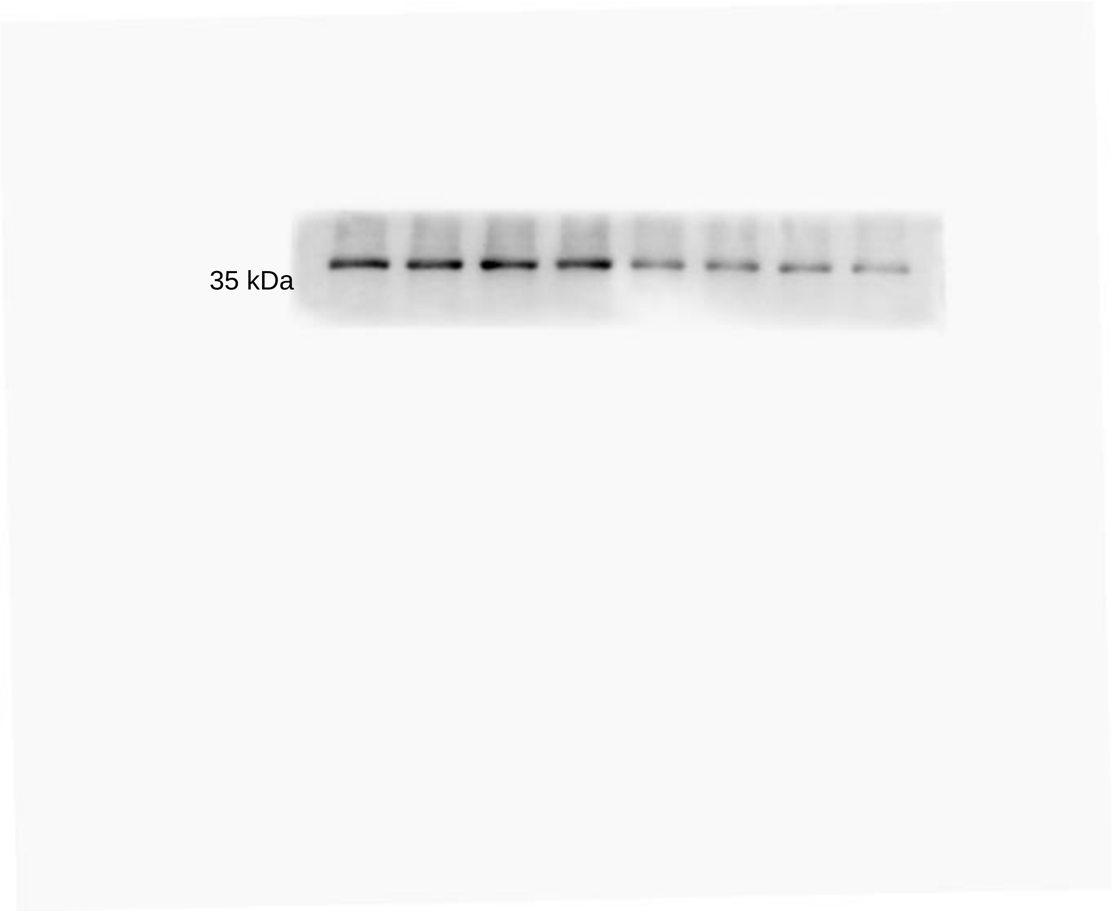

Fig 6A

IB: SLC25A46

Groups (ES2 cells): shCtrl (CHX: 0h, 1h, 2h, 4h), shSLC25A46 (CHX: 0h, 1h, 2h, 4h).

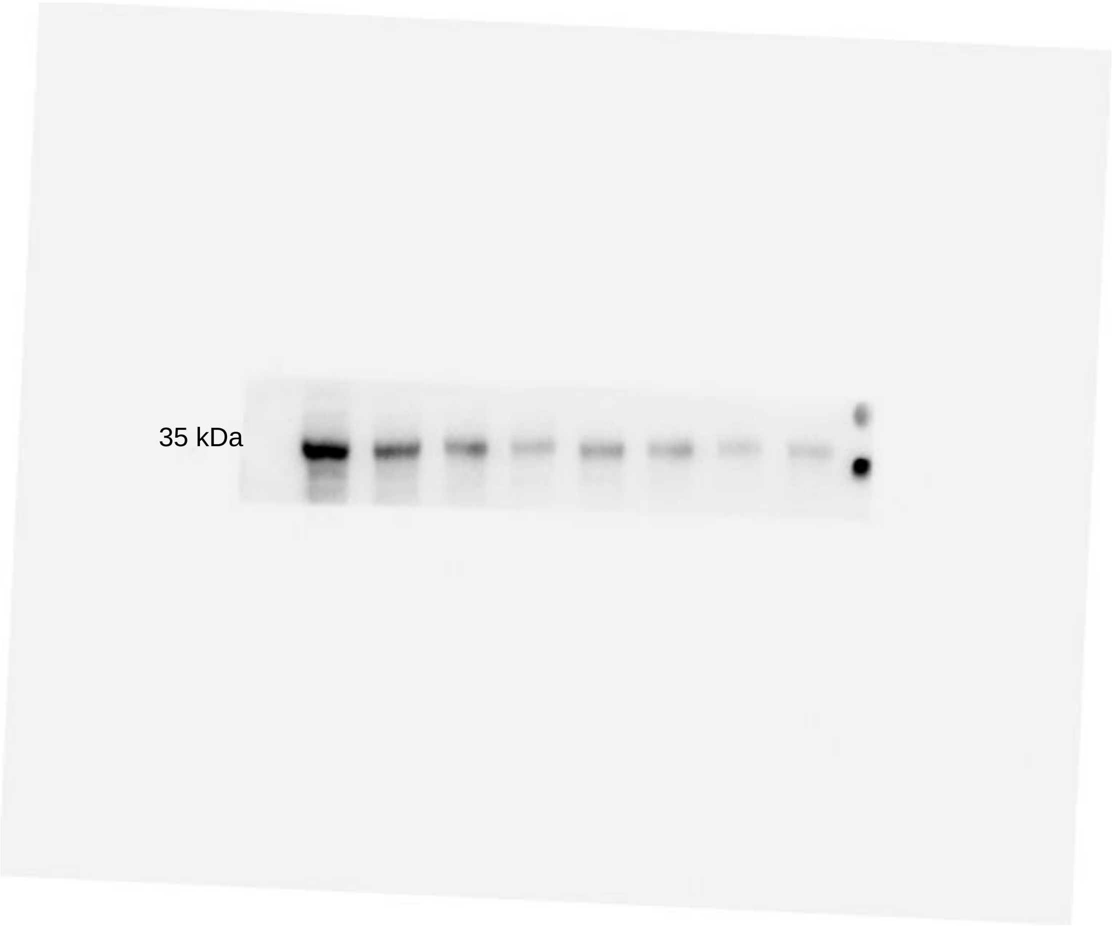

Fig 6A

IB: CACT

Groups (ES2 cells): shCtrl (CHX: 0h, 1h, 2h, 4h), shSLC25A46 (CHX: 0h, 1h, 2h, 4h)

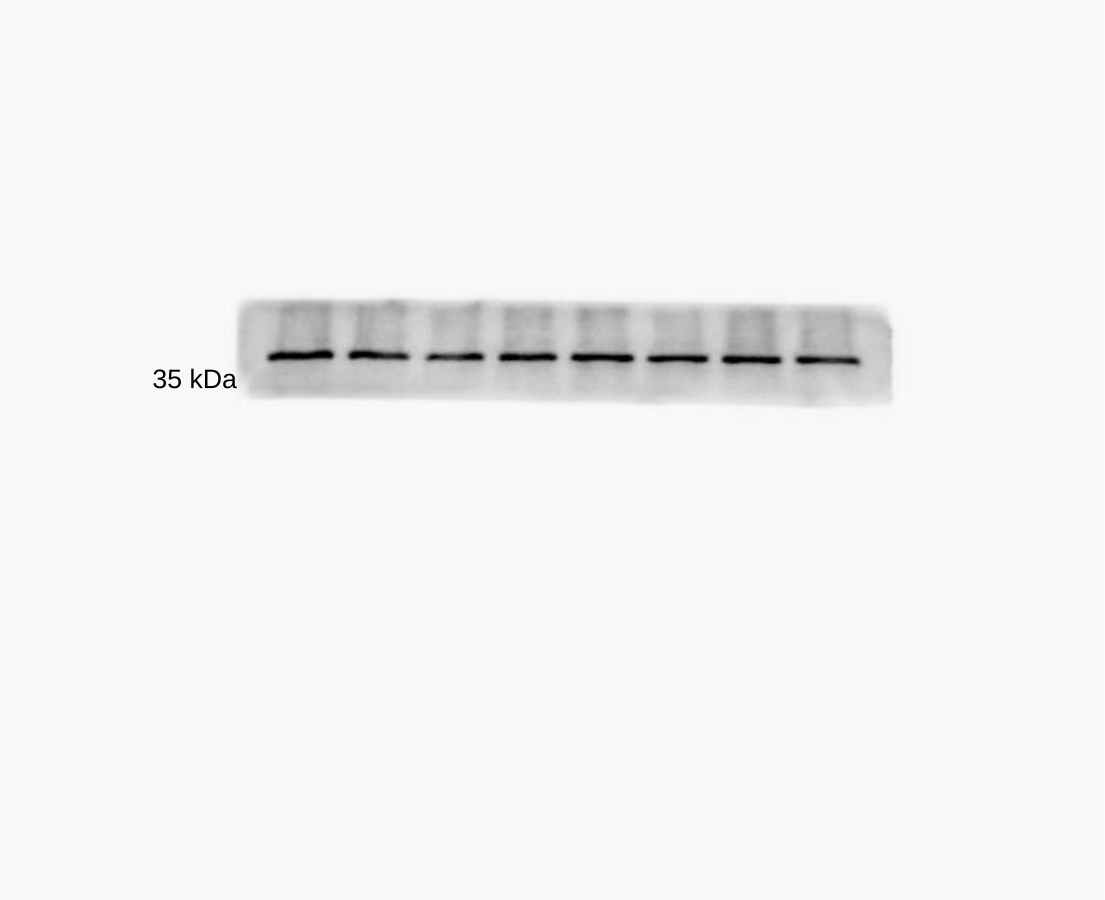

Fig 6A

IB: β-actin

Groups (ES2 cells): shCtrl (CHX: 0h, 1h, 2h, 4h), shSLC25A46 (CHX: 0h, 1h, 2h, 4h)

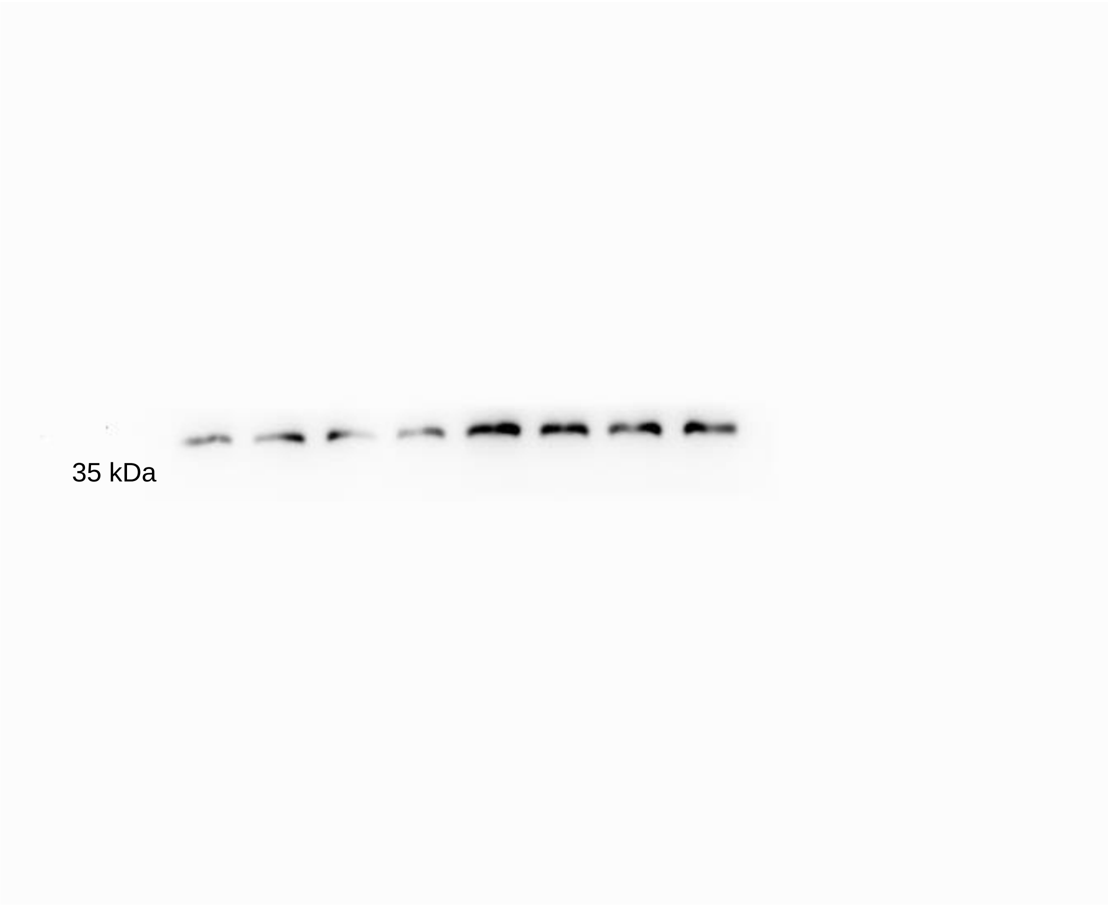

Fig 6A

IB: SLC25A46

Groups (HEY cells): EV (CHX: 0h, 1h, 2h, 4h), SLC25A46 (CHX: 0h, 1h, 2h, 4h).

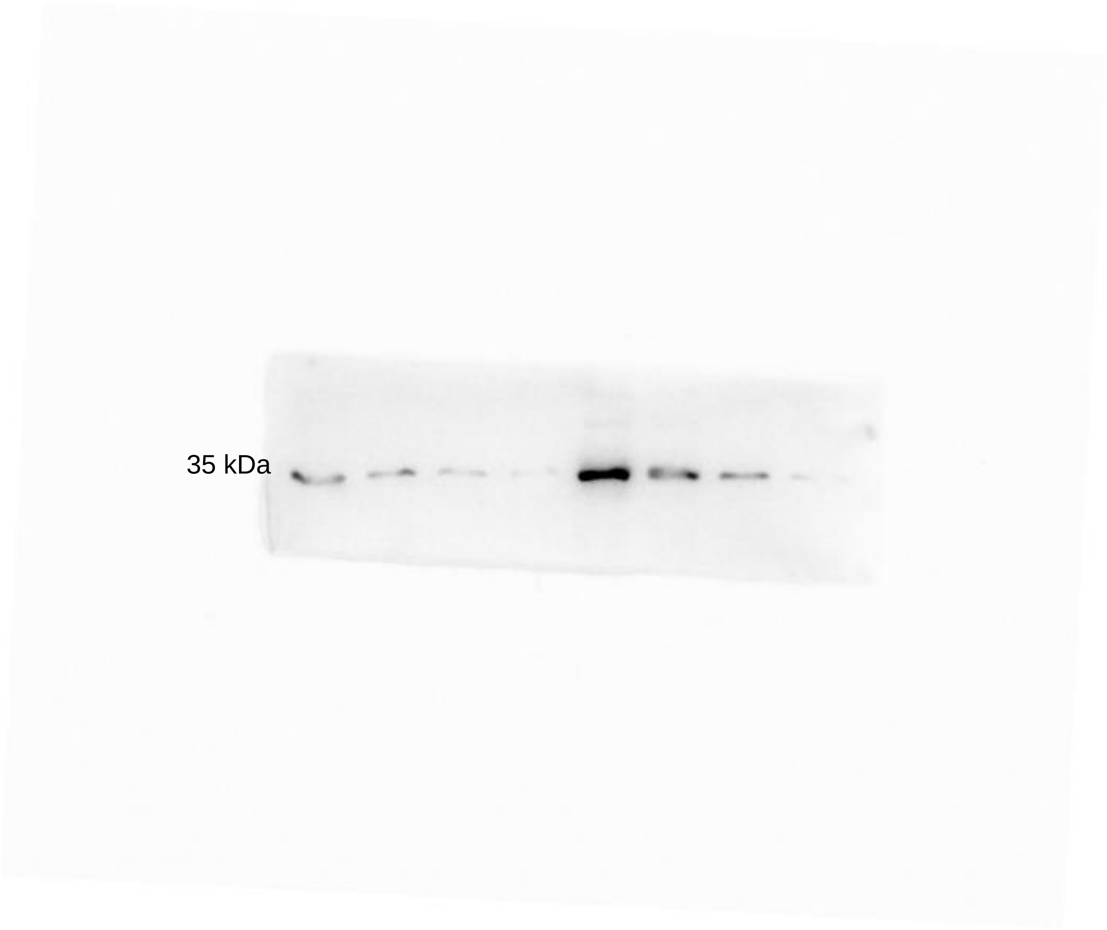

Fig 6A

IB: CACT

Groups (HEY cells): EV (CHX: 0h, 1h, 2h, 4h), SLC25A46 (CHX: 0h, 1h, 2h, 4h)

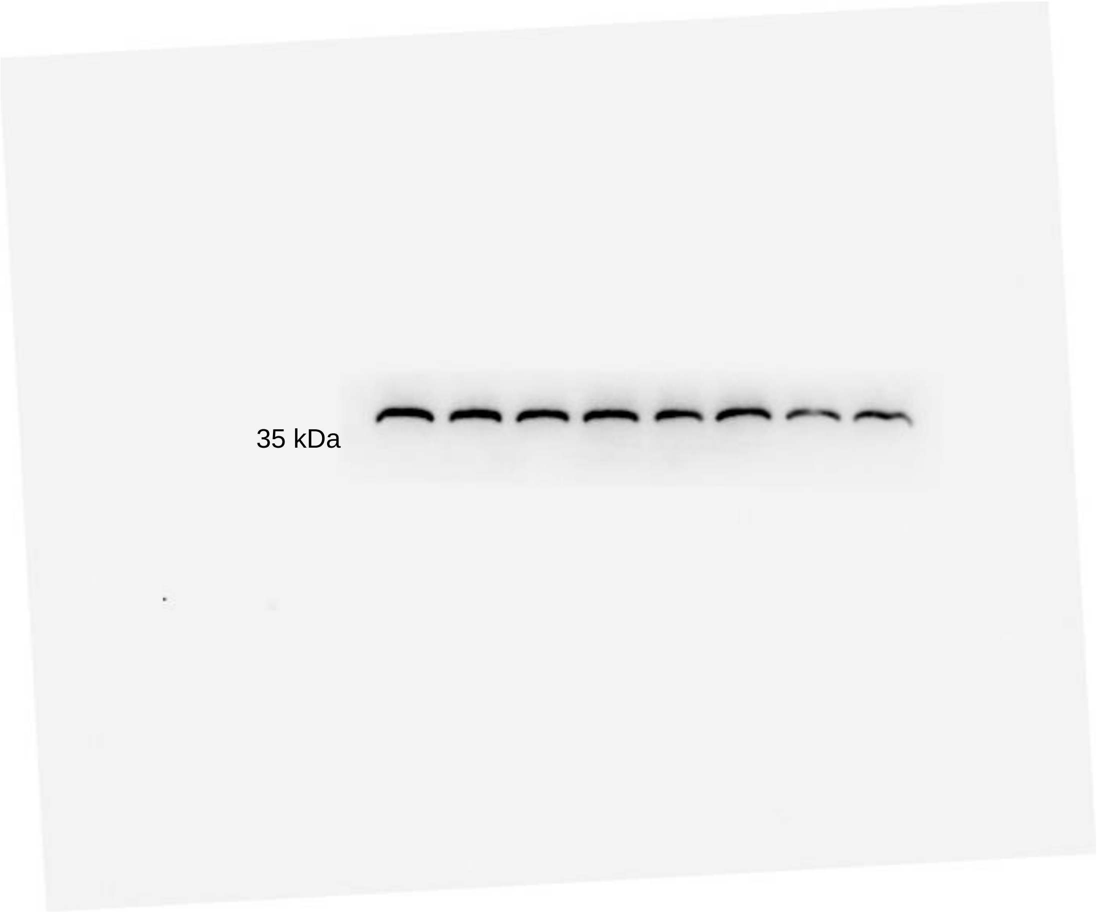

Fig 6A

IB: β-actin

Groups (HEY cells): EV (CHX: 0h, 1h, 2h, 4h), SLC25A46 (CHX: 0h, 1h, 2h, 4h)

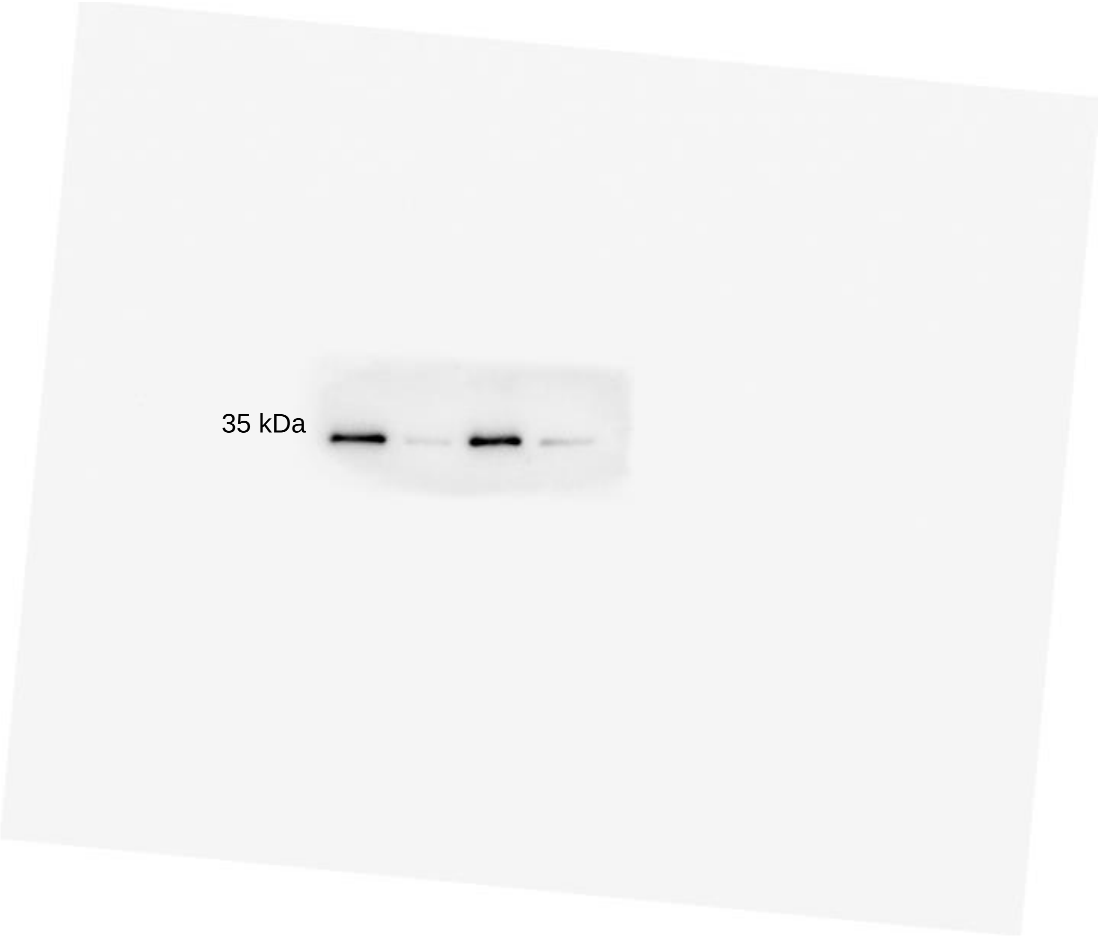

Fig 6B

IB: CACT

Groups (ES2 cells): shCtrl, SLC25A46 (DMSO, MG132, CQ)

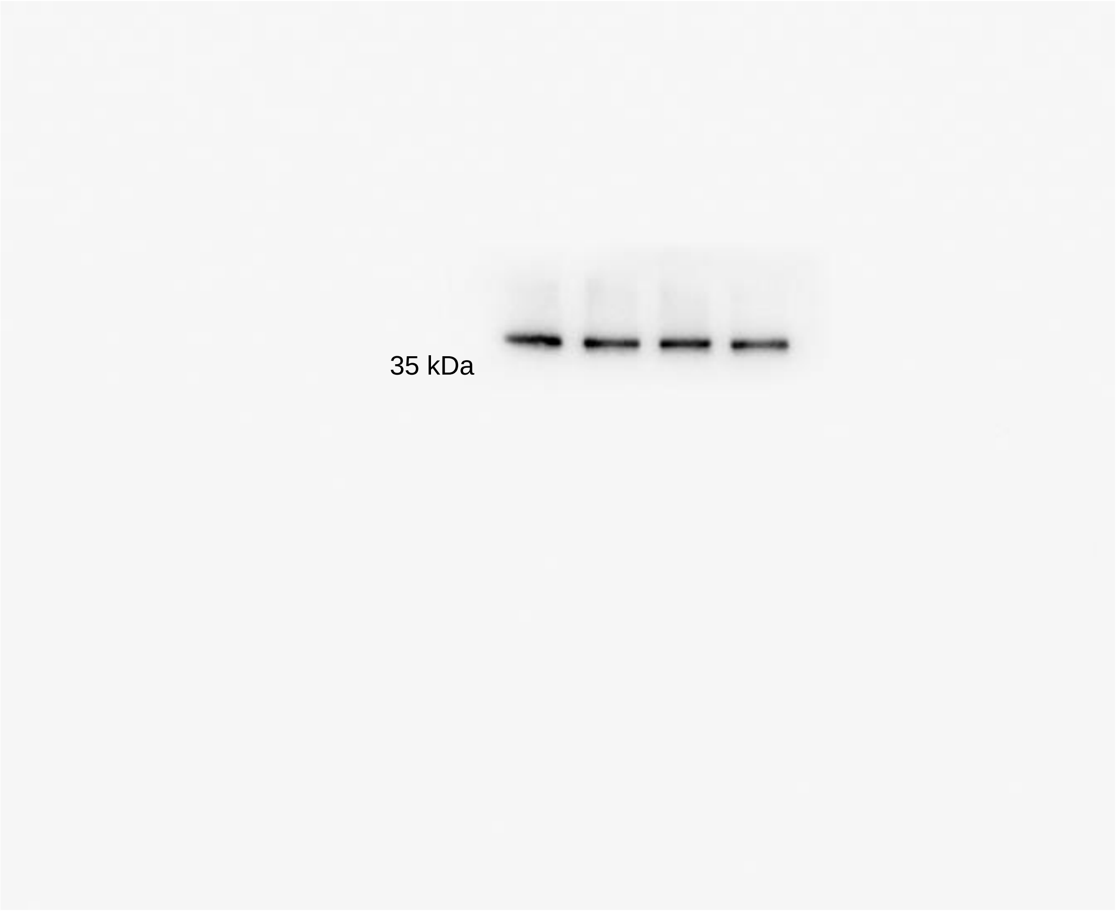

Fig 6B

IB: β-actin

Groups (ES2 cells): shCtrl, SLC25A46 (DMSO, MG132, CQ)

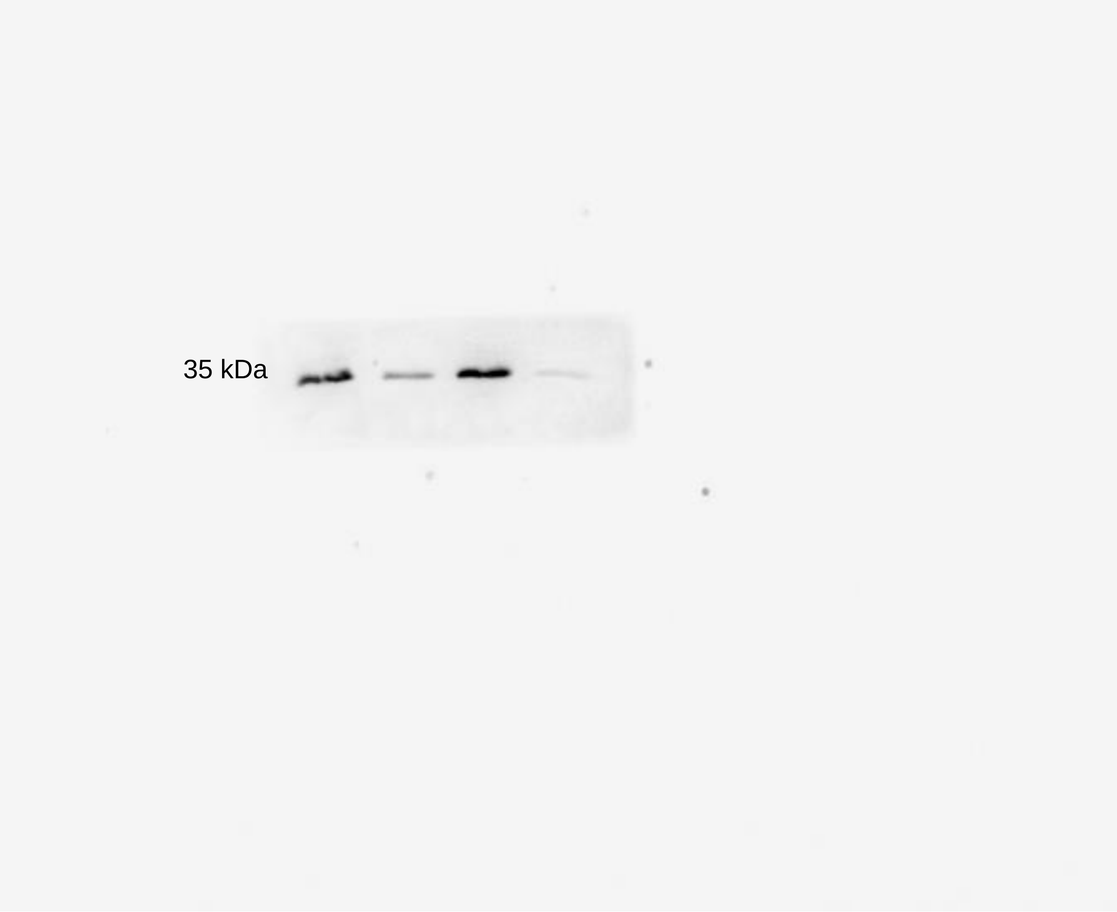

Fig 6B

IB: CACT

Groups (SKOV3 cells): shCtrl, SLC25A46 (DMSO, MG132, CQ)

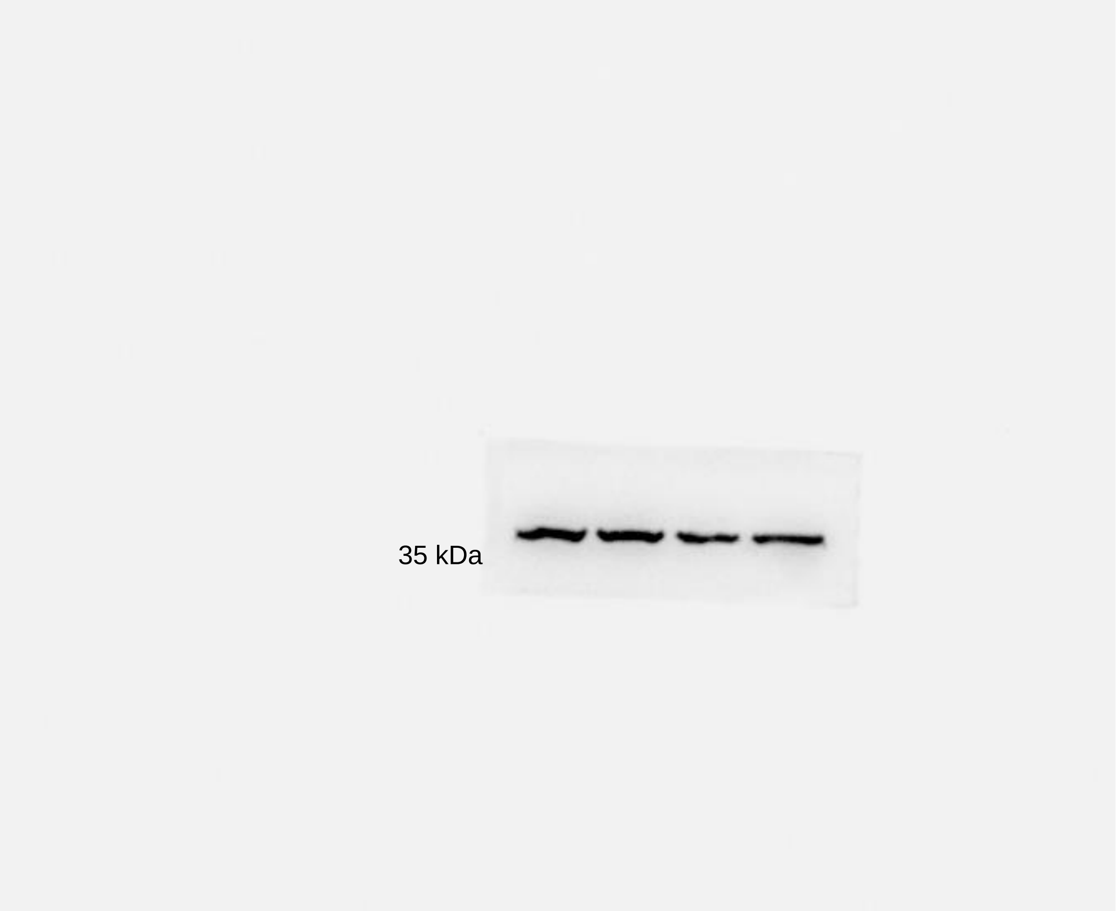

Fig 6B

IB: β-actin

Groups (SKOV3 cells): shCtrl, SLC25A46 (DMSO, MG132, CQ)

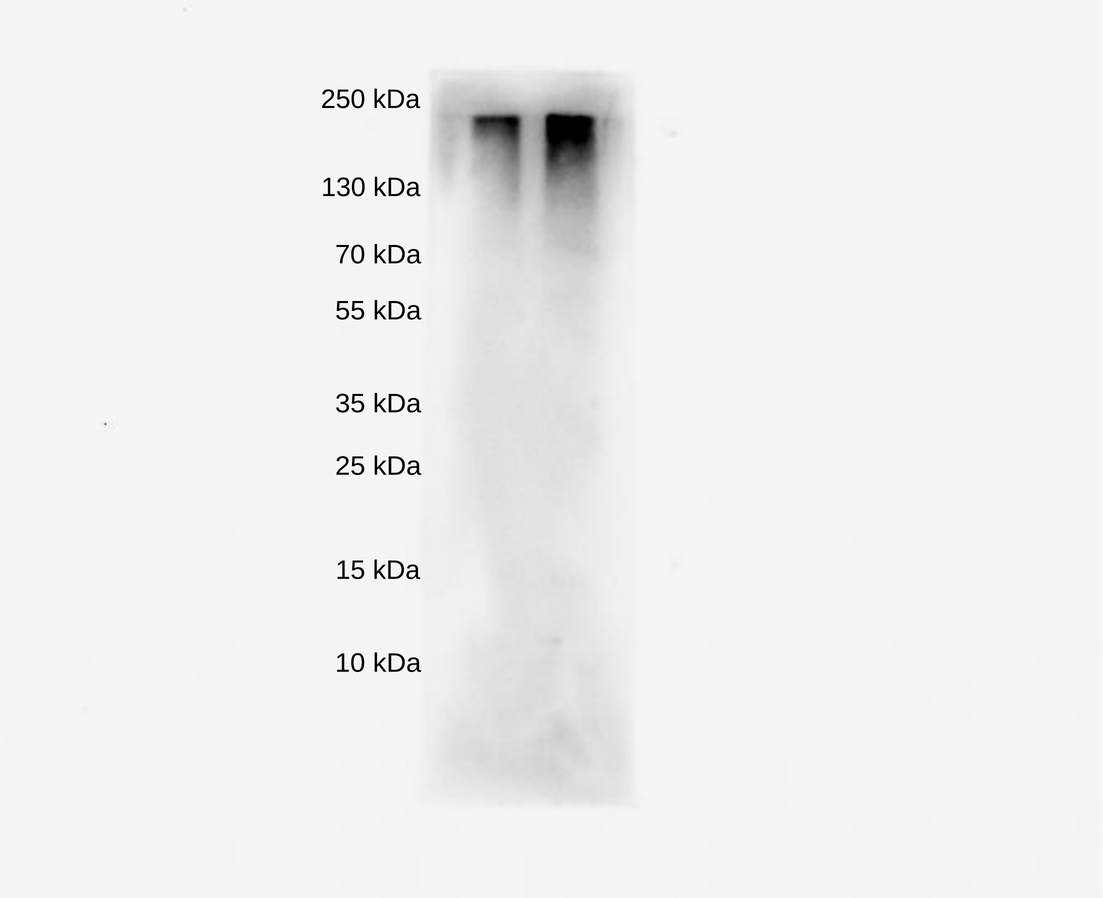

Fig 6C

IB: Anti-Ub

Groups (ES2 cells): shCtrl+MG132, SLC25A46+MG132

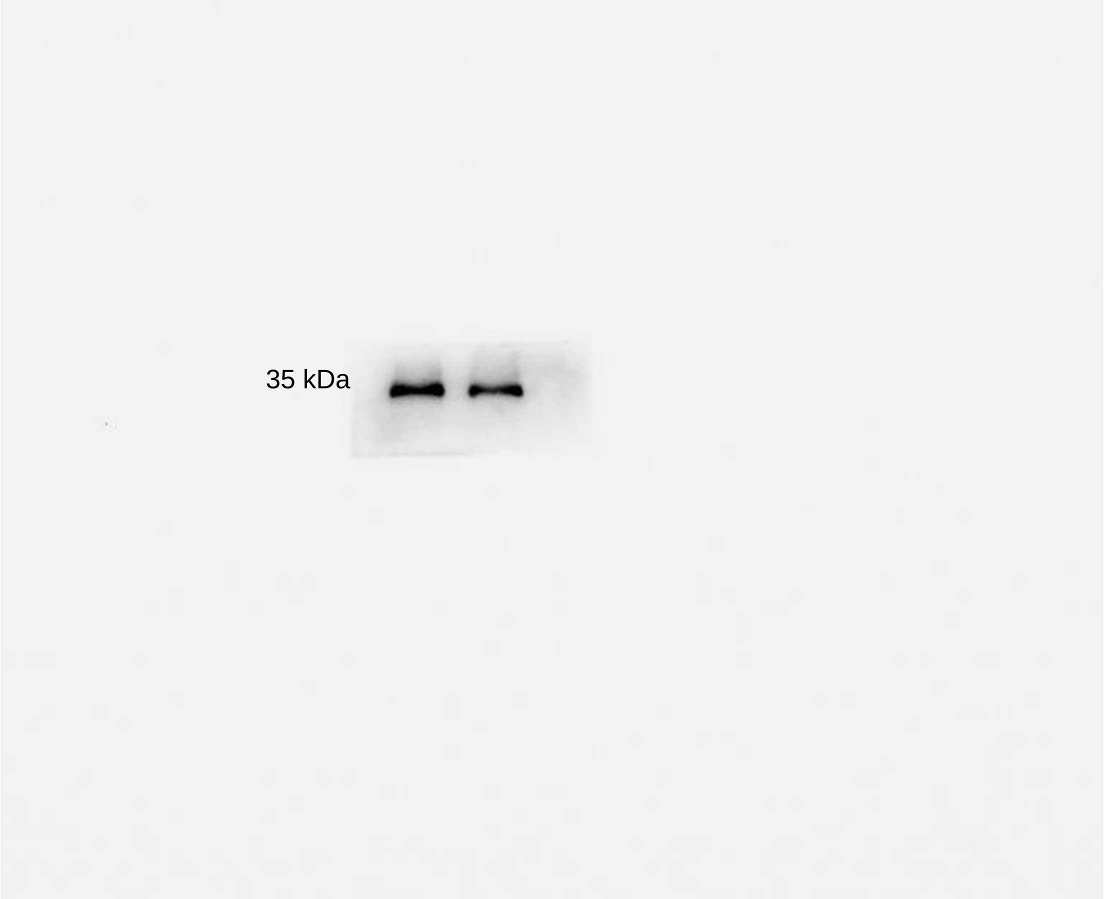

Fig 6C

IB: CACT

Groups (ES2 cells): shCtrl+MG132, SLC25A46+MG132

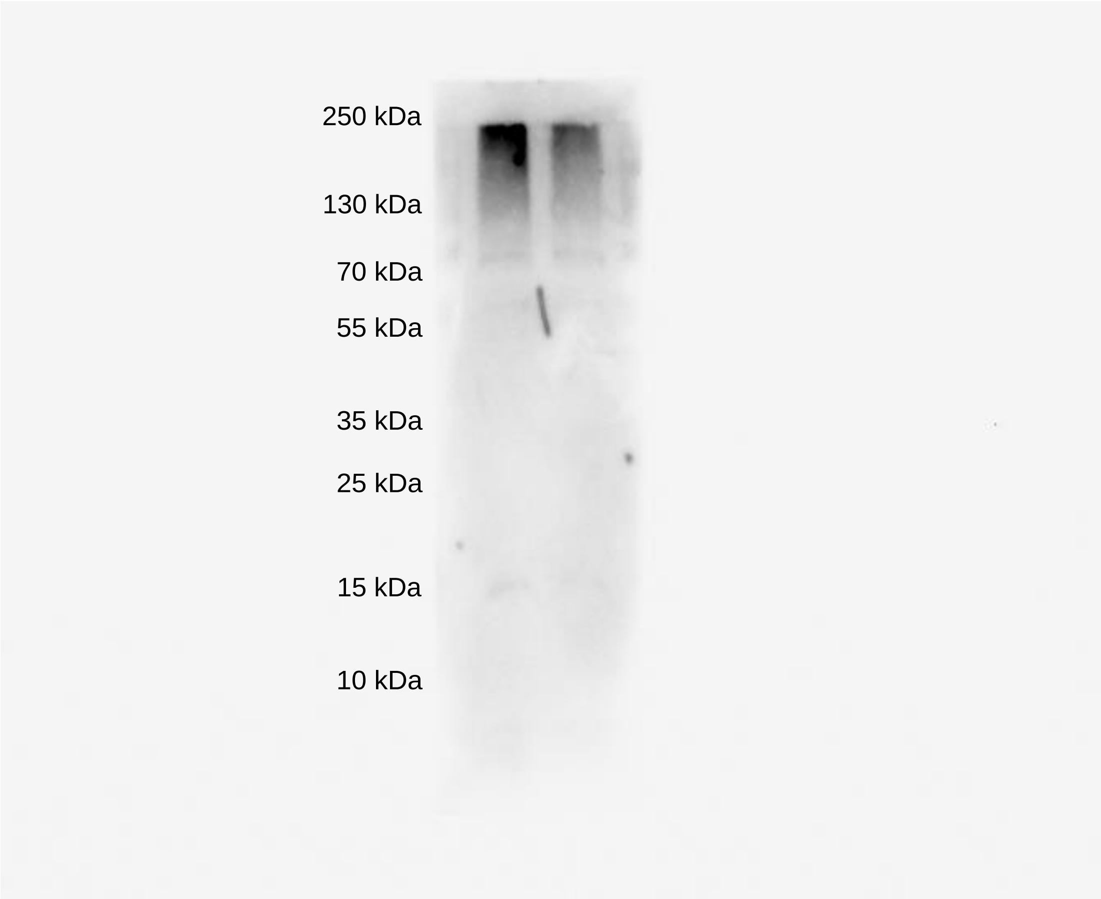

Fig 6C

IB: Anti-Ub

Groups (HEY cells): shCtrl+MG132, SLC25A46+MG132

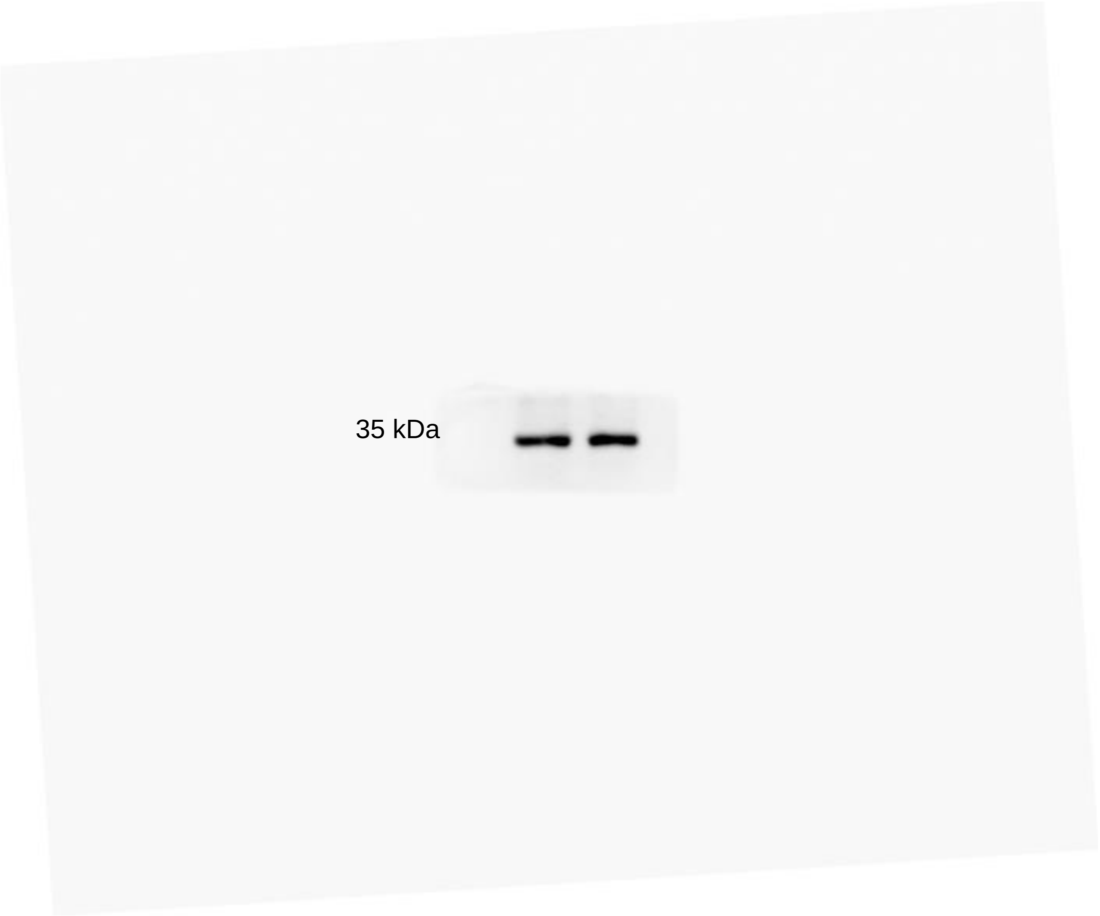

Fig 6C

IB: CACT

Groups (HEY cells): shCtrl+MG132, SLC25A46+MG132

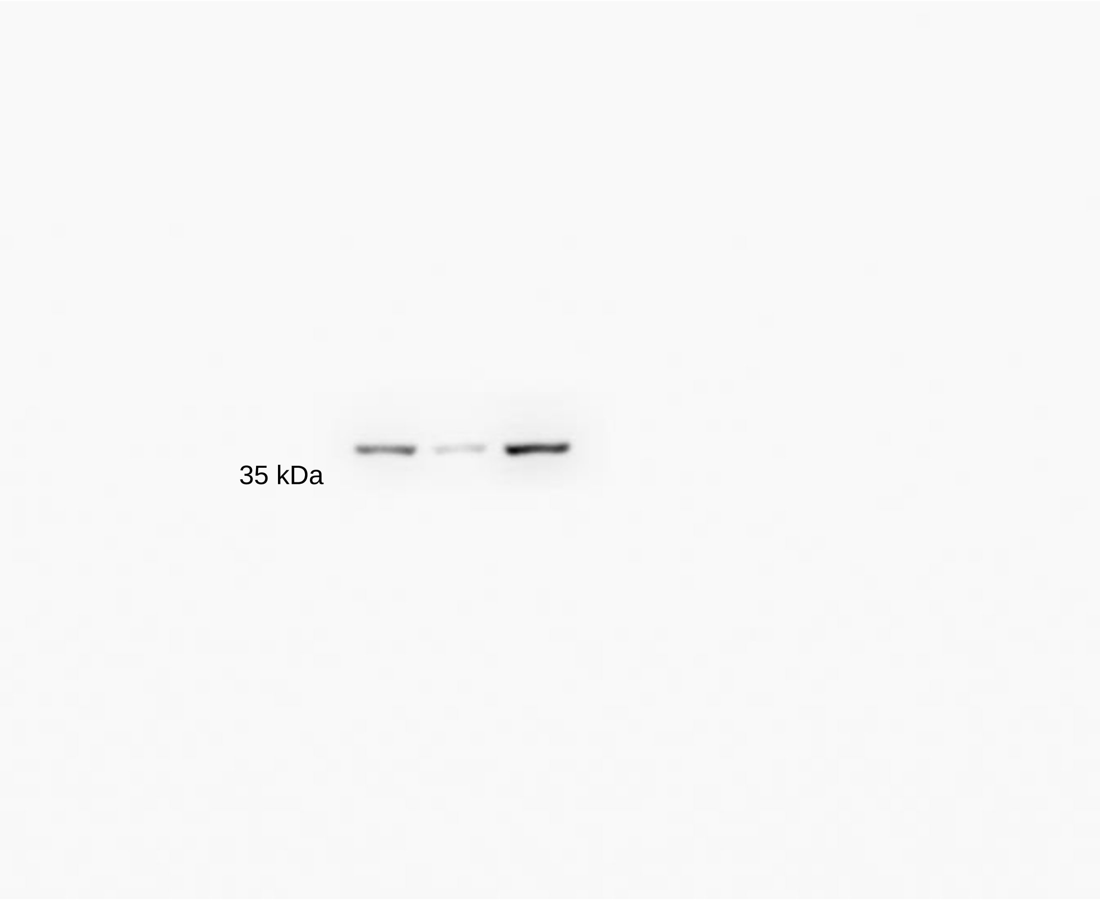

Fig 6D

IB: SLC25A46

Groups (ES2 cells): Input, IgG (IP), SLC25A46 (IP)

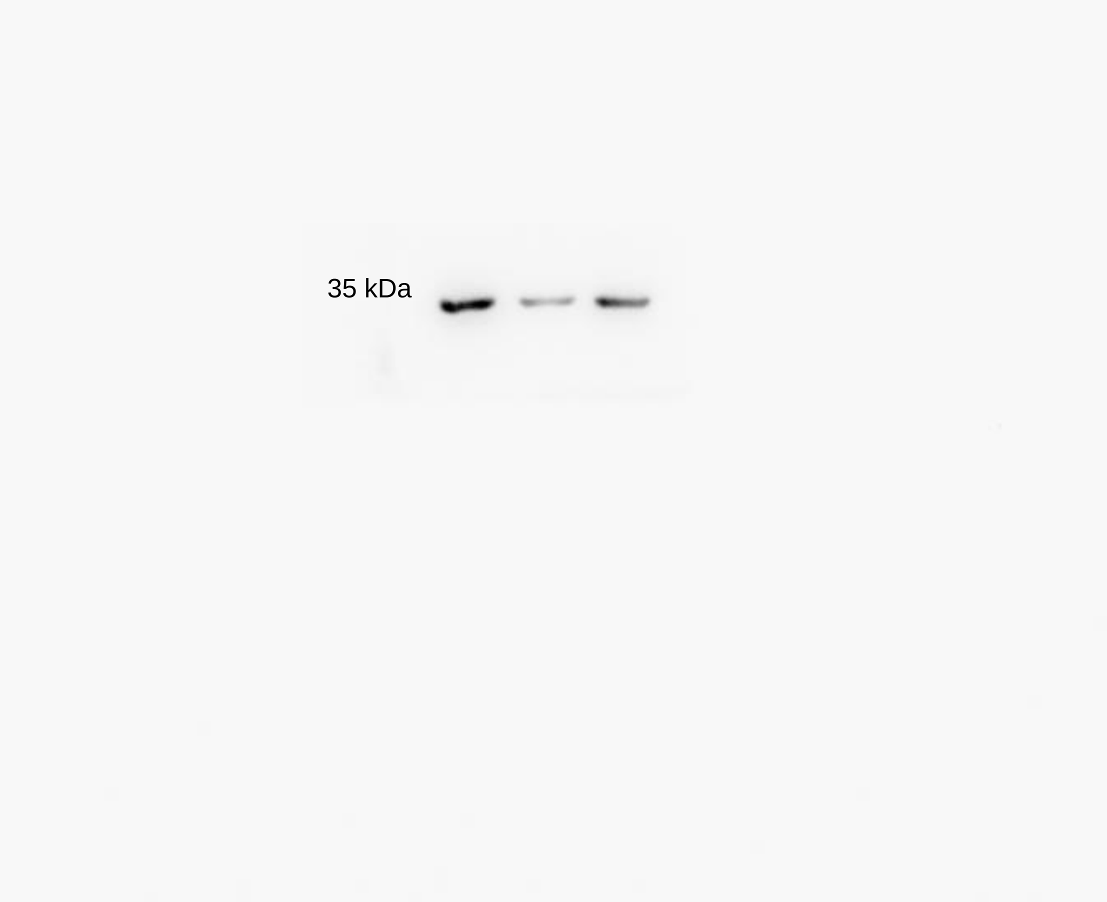

Fig 6D

IB: MARCHF5

Groups (ES2 cells): Input, IgG (IP), SLC25A46 (IP)

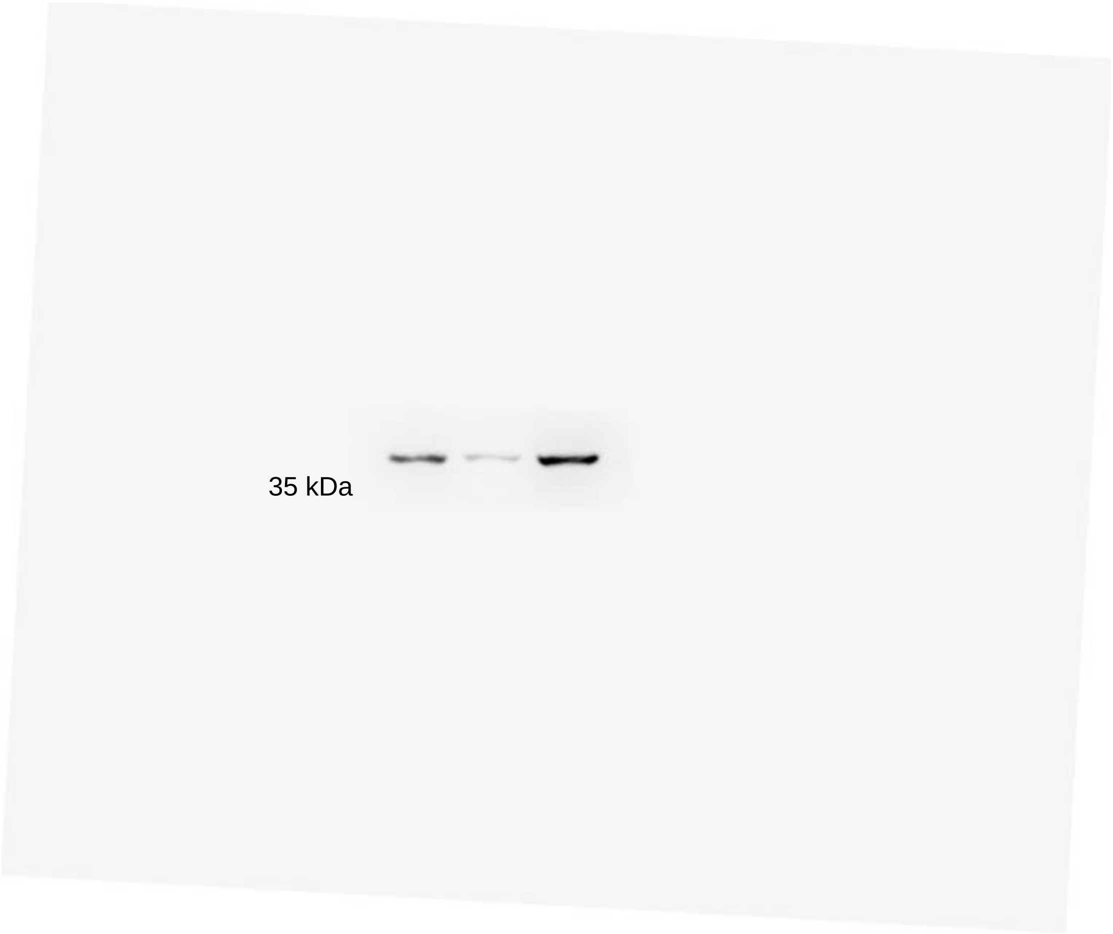

Fig 6D

IB: SLC25A46

Groups (SKOV3 cells): Input, IgG (IP), SLC25A46 (IP)

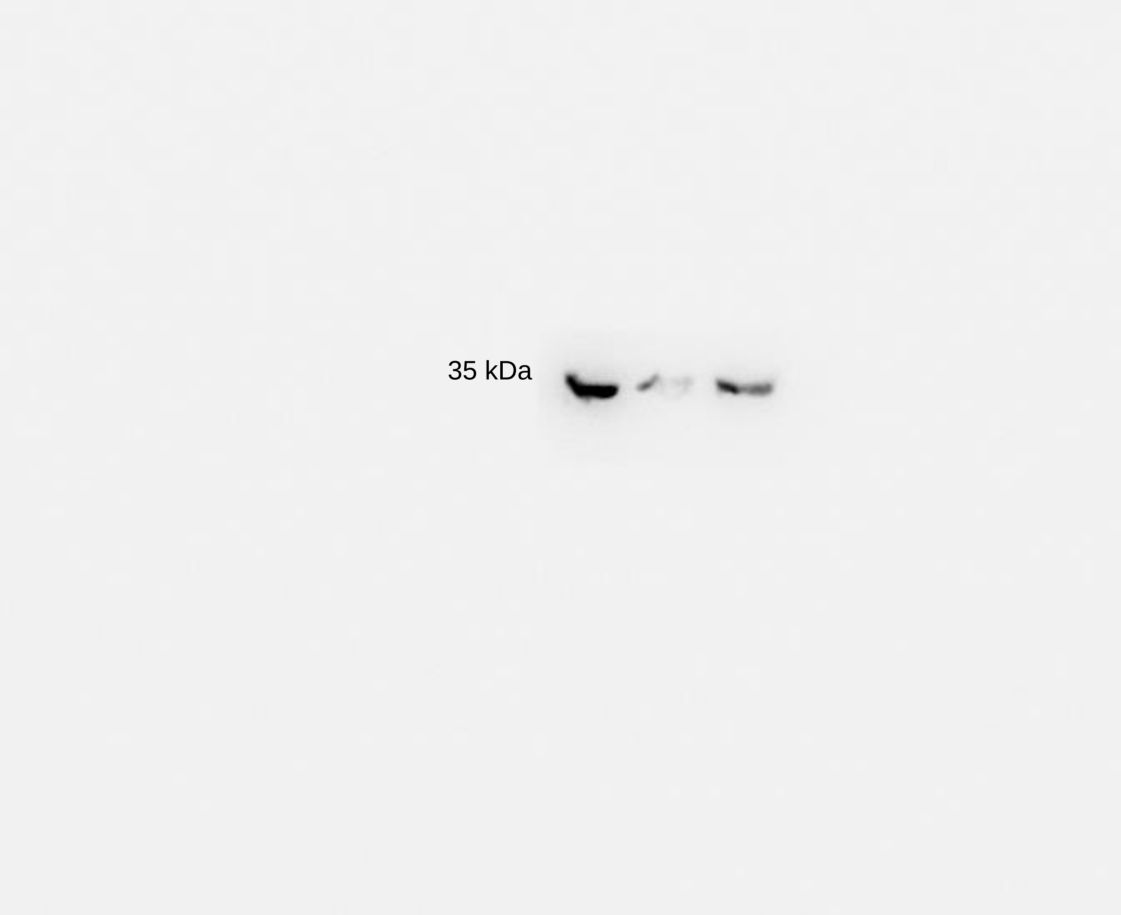

Fig 6D

IB: MARCHF5

Groups (SKOV3 cells): Input, IgG (IP), SLC25A46 (IP)

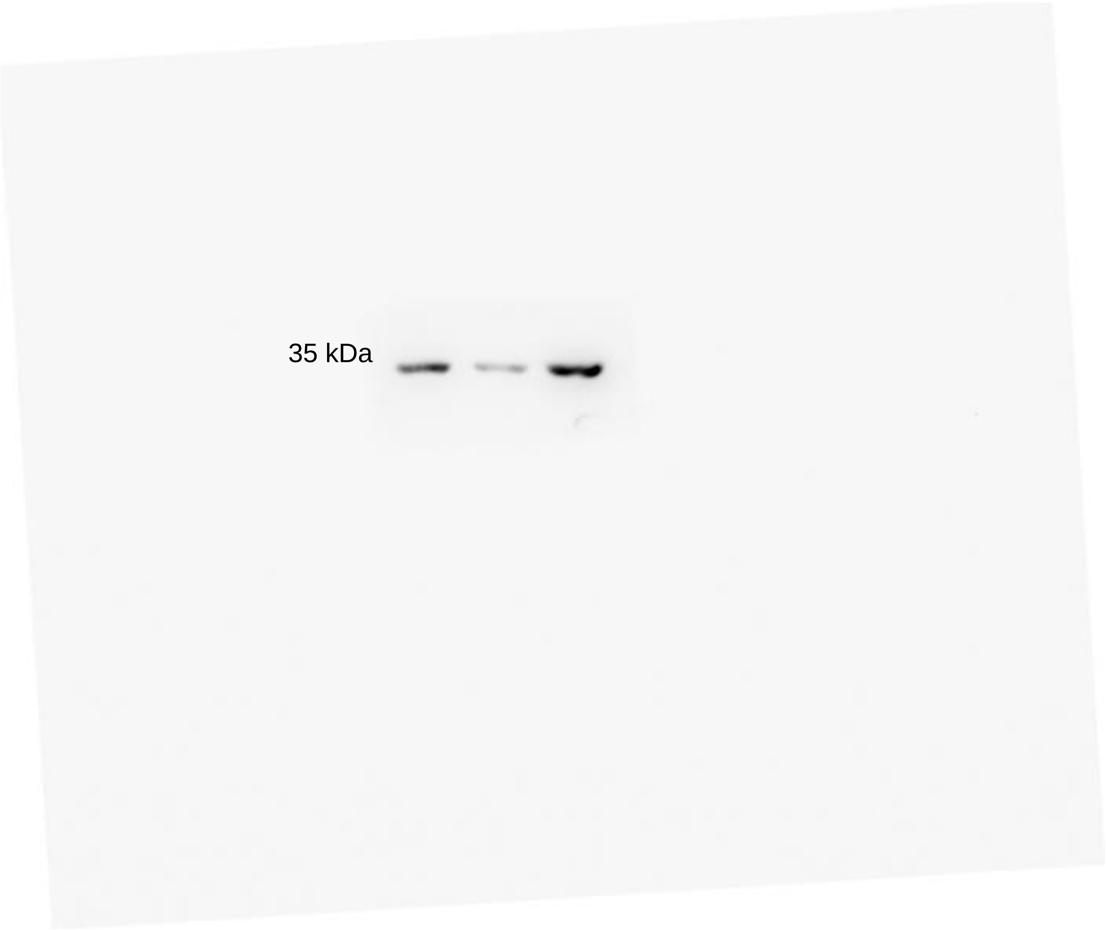

Fig 6E

IB: MARCHF5

Groups (ES2 cells): Input, IgG (IP), MARCHF5 (IP)

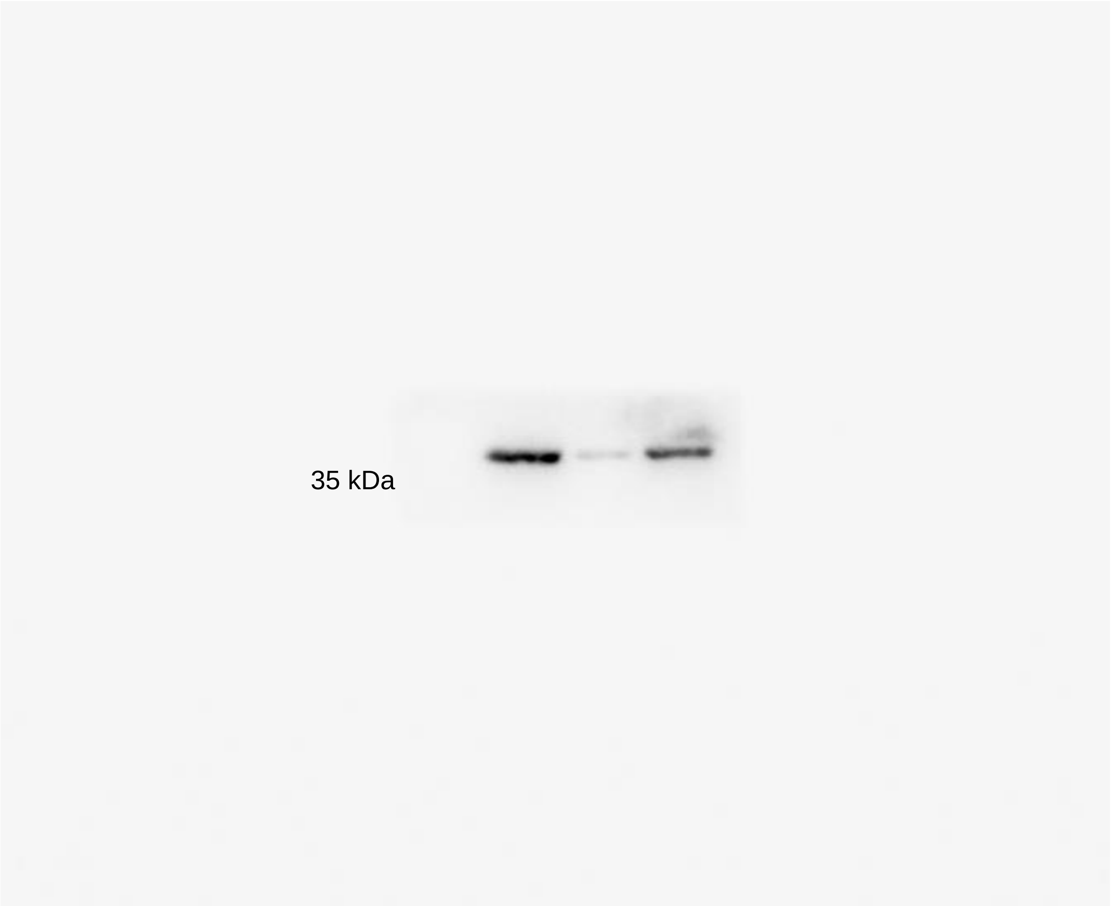

Fig 6E

IB: SLC25A46

Groups (ES2 cells): Input, IgG (IP), MARCHF5 (IP)

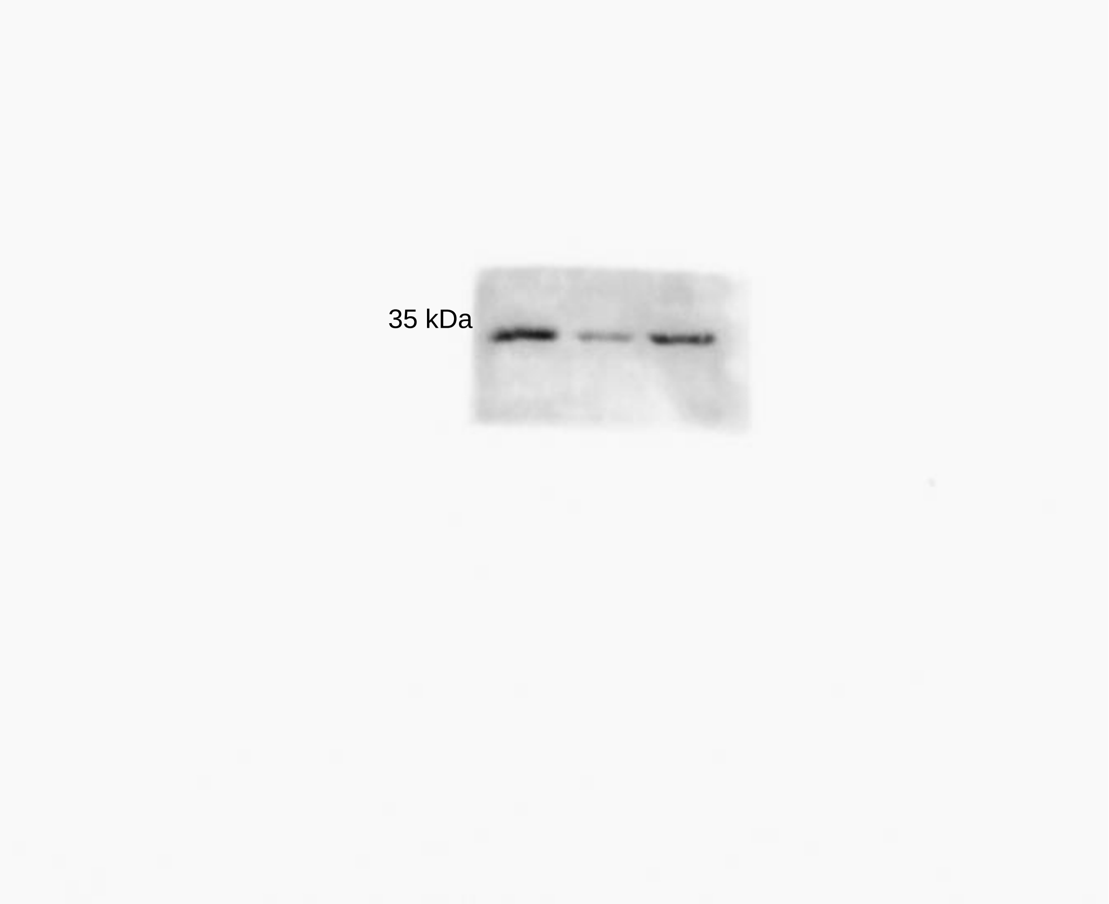

Fig 6E

IB: CACT

Groups (ES2 cells): Input, IgG (IP), MARCHF5 (IP)

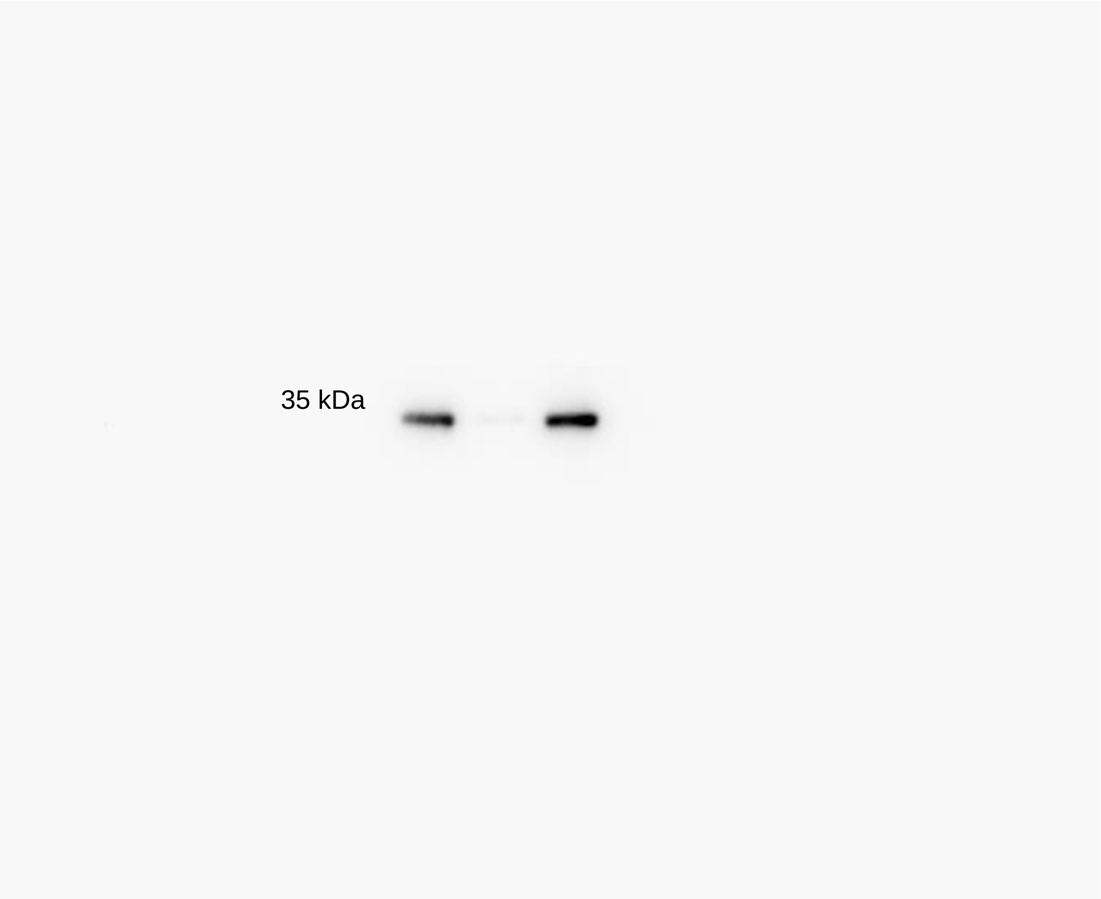

Fig 6E

IB: MARCHF5

Groups (SKOV3 cells): Input, IgG (IP), MARCHF5 (IP)

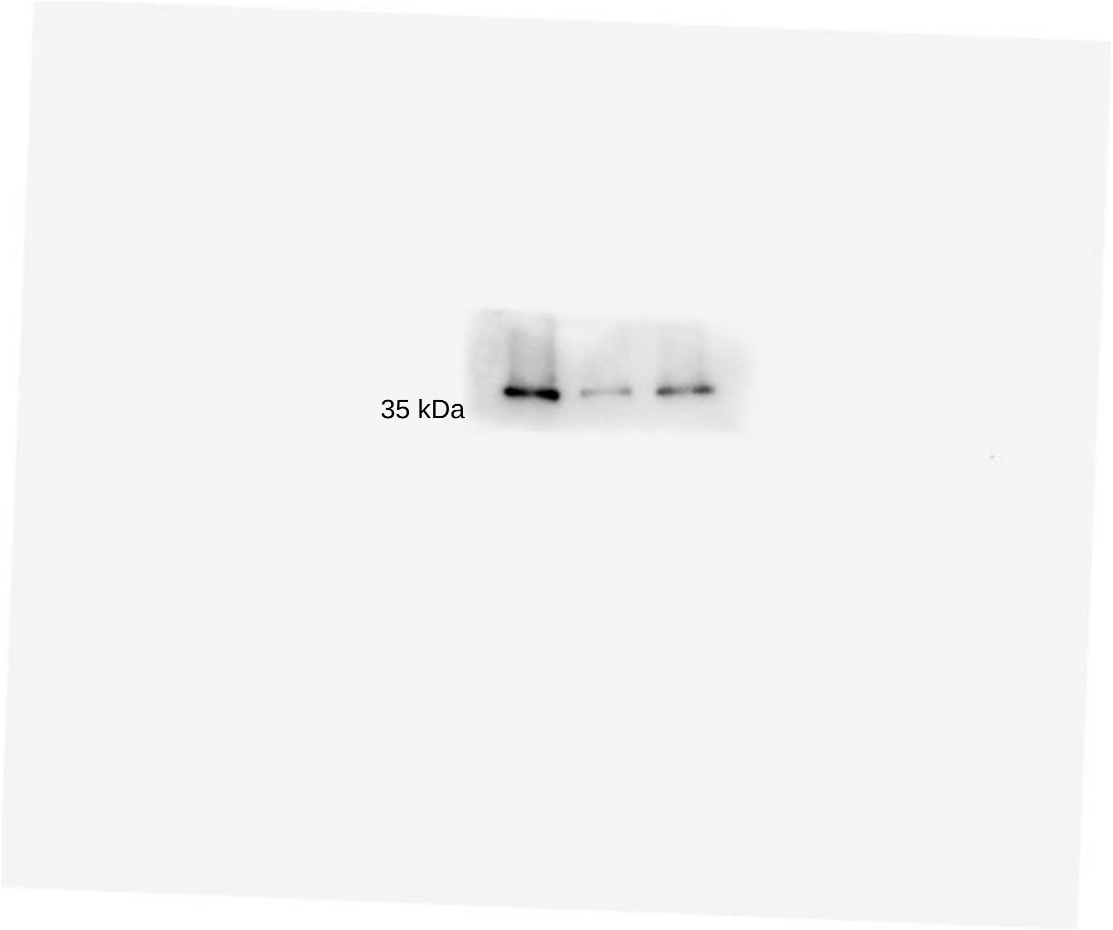

Fig 6E

IB: SLC25A46

Groups (SKOV3 cells): Input, IgG (IP), MARCHF5 (IP)

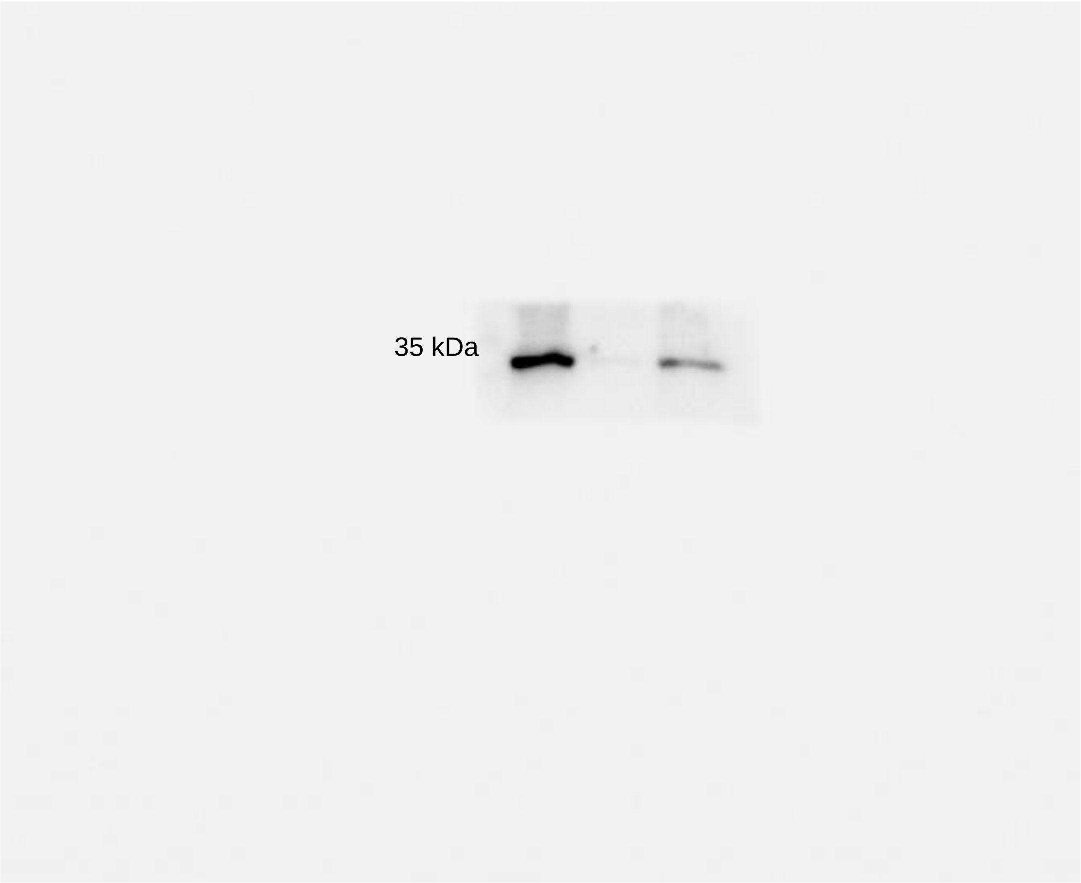

Fig 6E

IB: CACT

Groups (SKOV3 cells): Input, IgG (IP), MARCHF5 (IP)

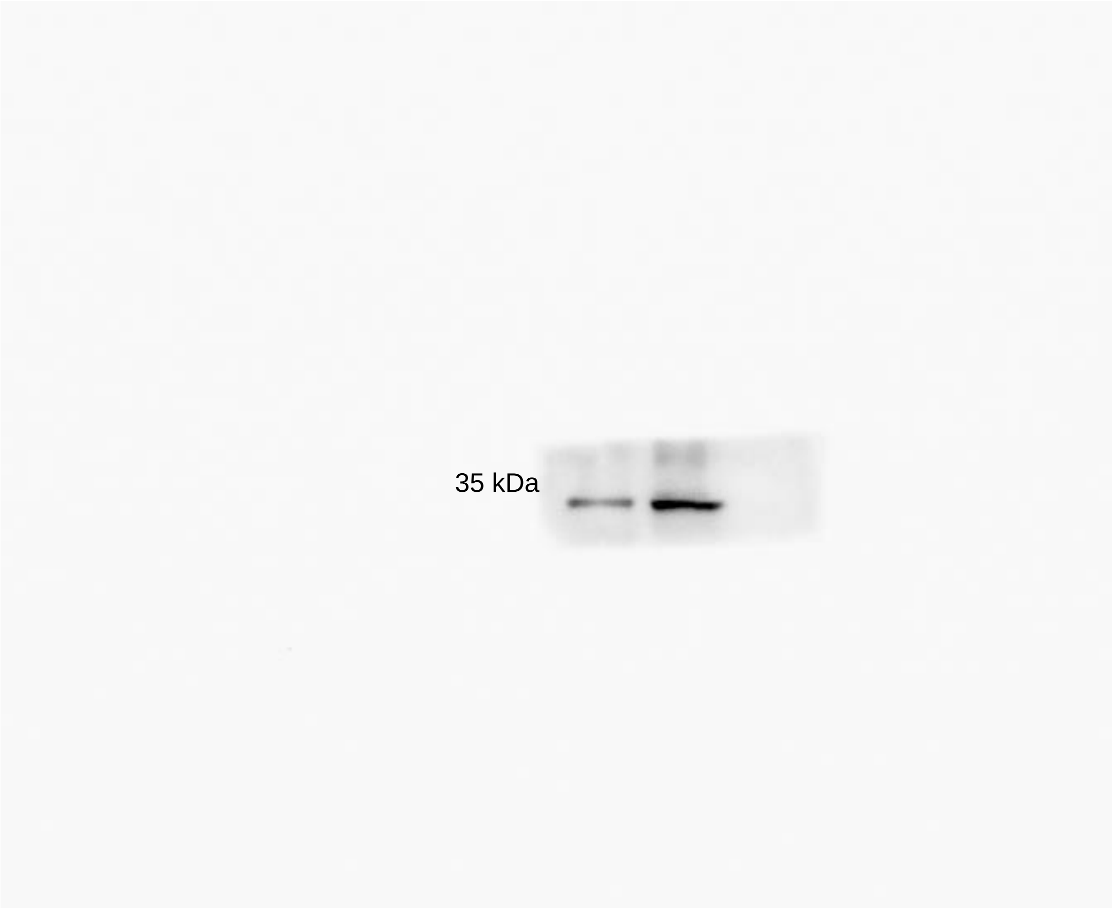

Fig 6F

IB: CACT

Groups (ES2 cells): shCtrl, shMARCHF5

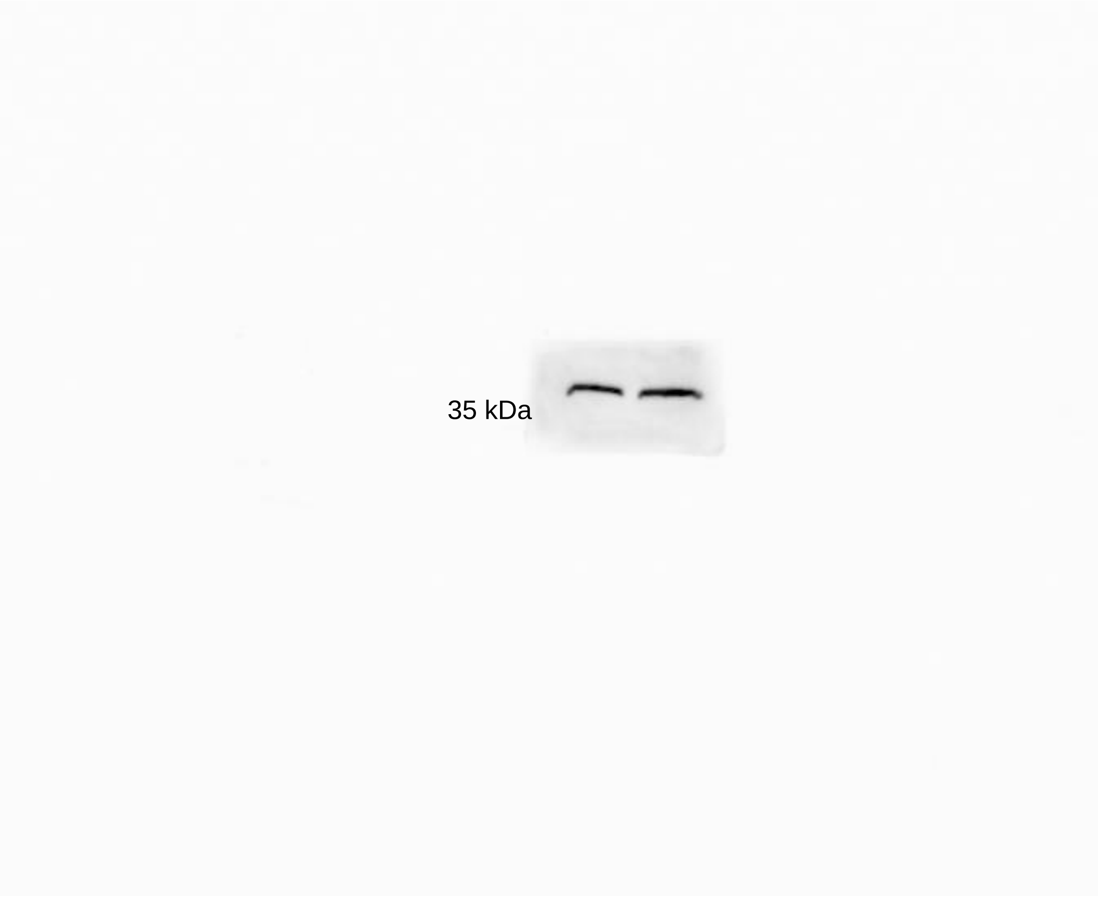

Fig 6F

IB: β-actin

Groups (ES2 cells): shCtrl, shMARCHF5

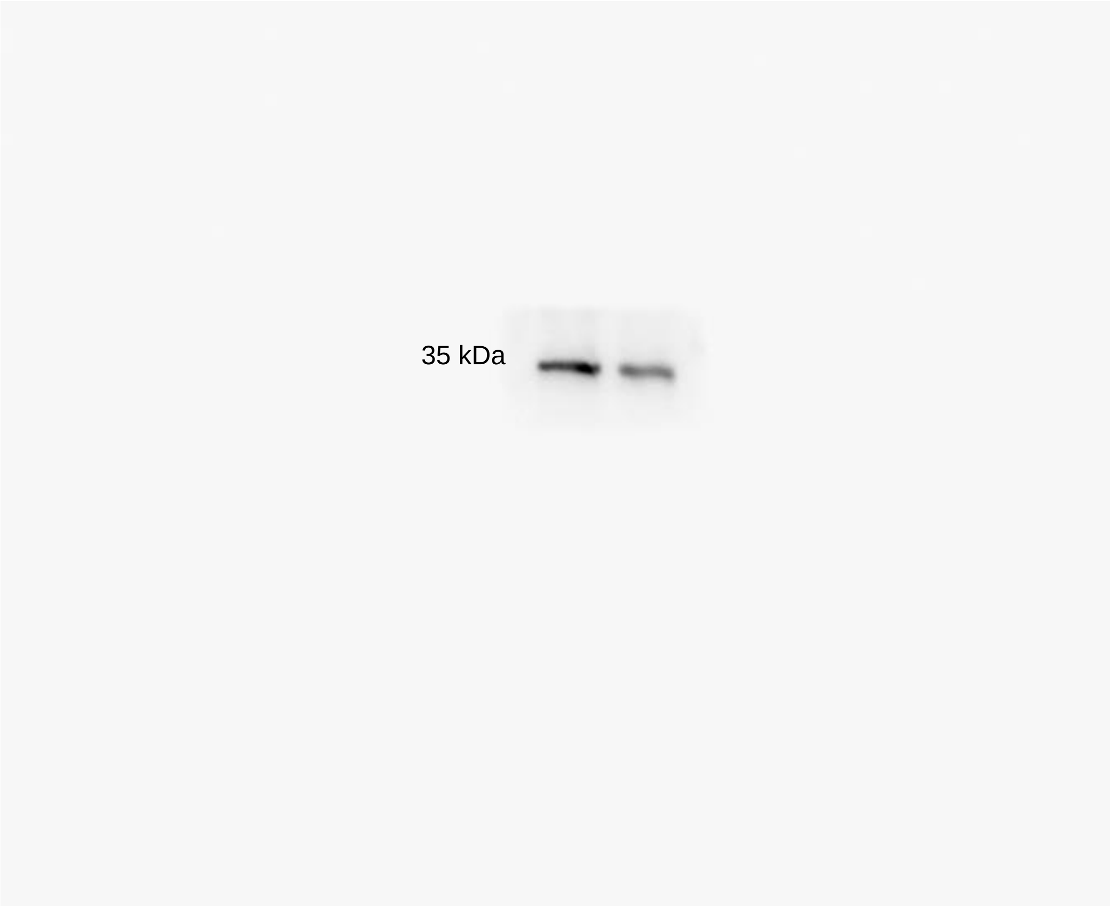

Fig 6F

IB: CACT

Groups (HEY cells): EV, MARCHF5

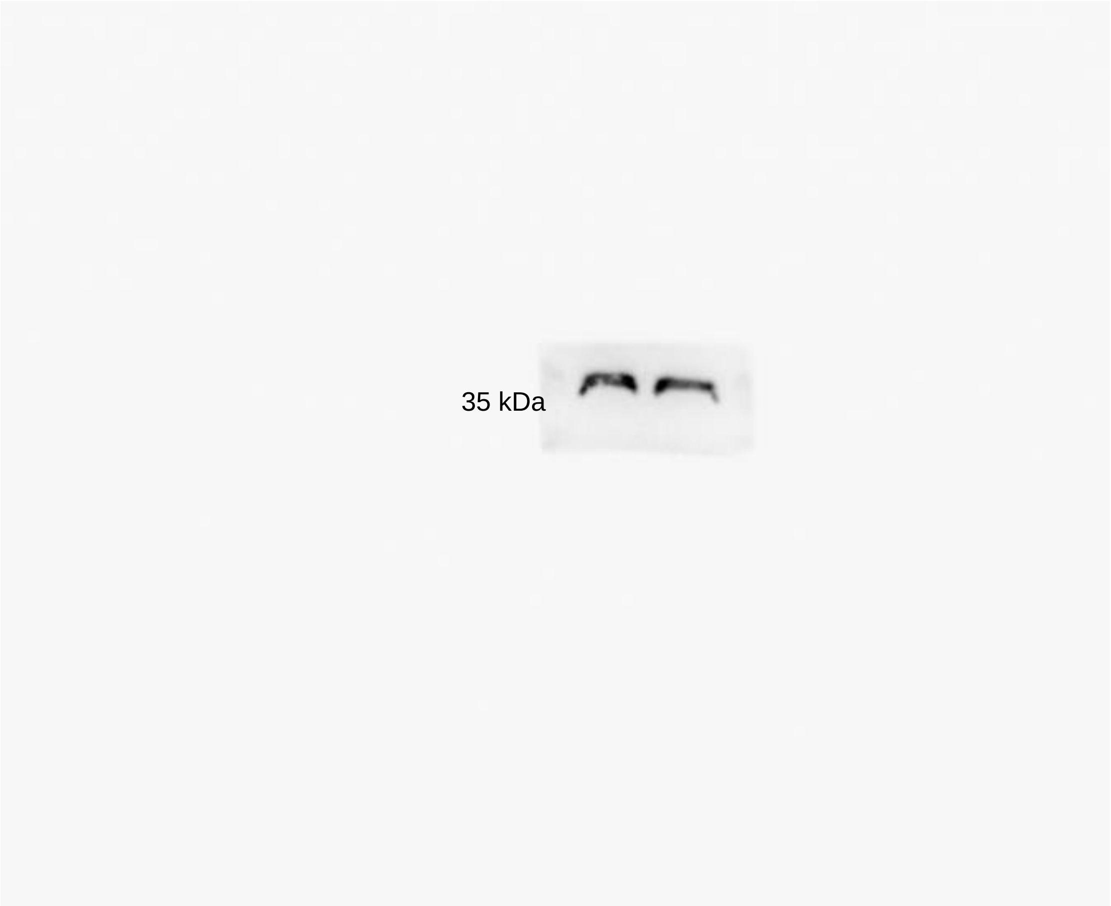

Fig 6F

IB: β-actin

Groups (HEY cells): EV, MARCHF5

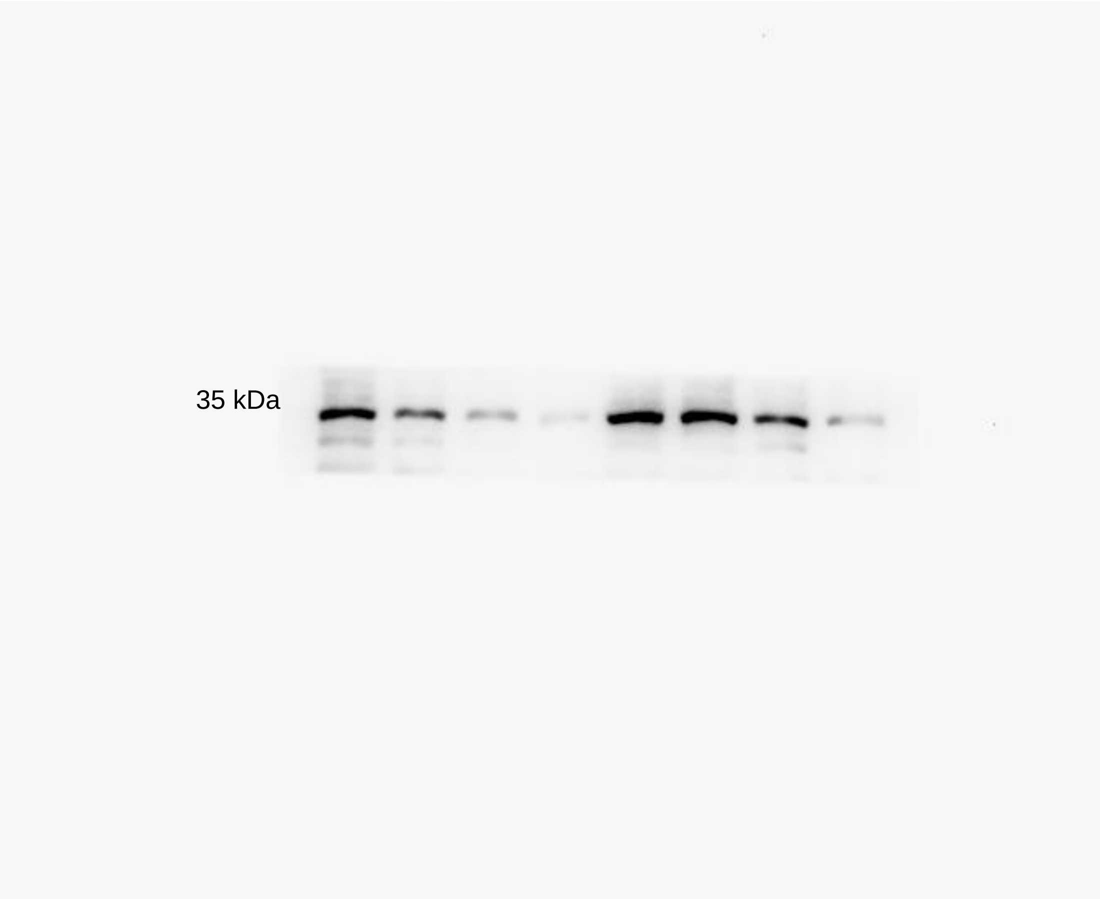

Fig 6G

IB: CACT

Groups (ES2 cells): shCtrl (CHX: 0h, 1h, 2h, 4h), shMARCHF5 (CHX: 0h, 1h, 2h, 4h)

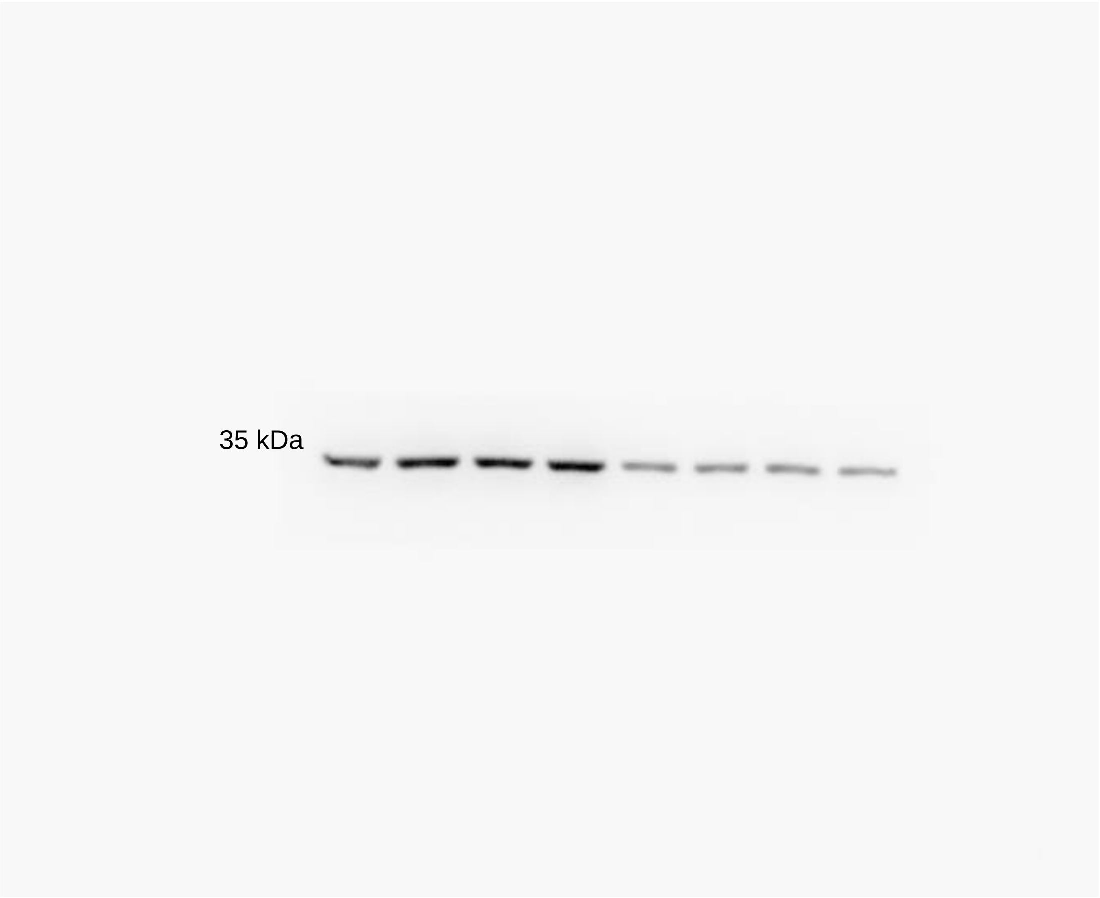

Fig 6G

IB: MARCHF5

Groups (ES2 cells): shCtrl (CHX: 0h, 1h, 2h, 4h), shMARCHF5 (CHX: 0h, 1h, 2h, 4h)

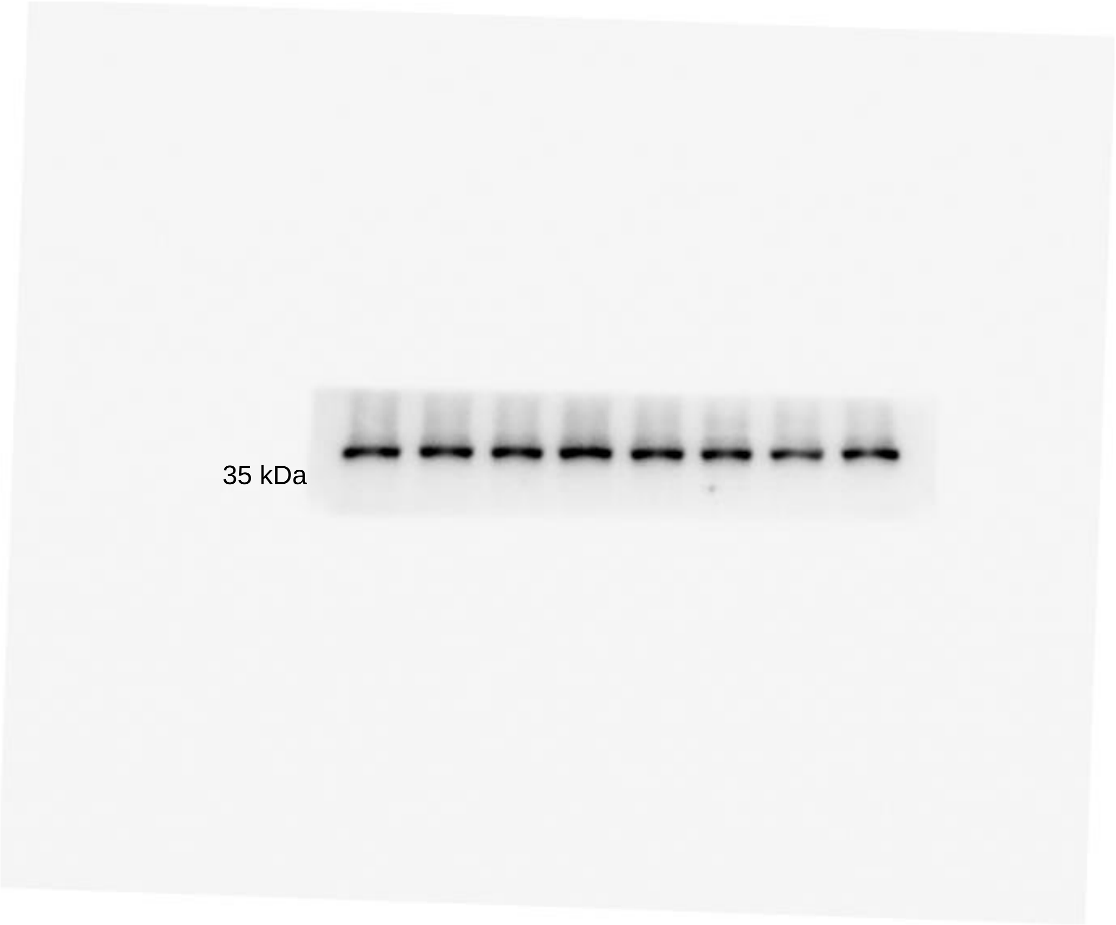

Fig 6G

IB: β-actin

Groups (ES2 cells): shCtrl (CHX: 0h, 1h, 2h, 4h), shMARCHF5 (CHX: 0h, 1h, 2h, 4h)

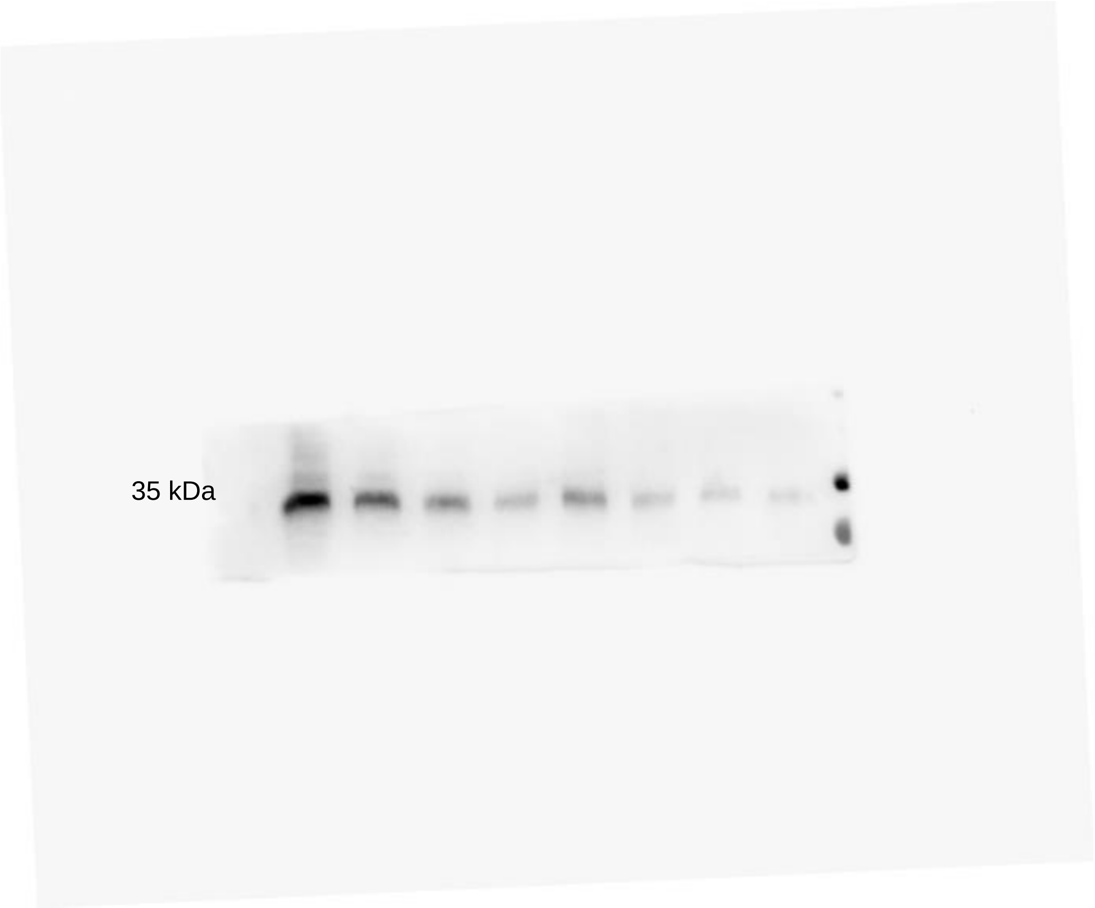

Fig 6G

IB: CACT

Groups (HEY cells): EV (CHX: 0h, 1h, 2h, 4h), MARCHF5 (CHX: 0h, 1h, 2h, 4h)

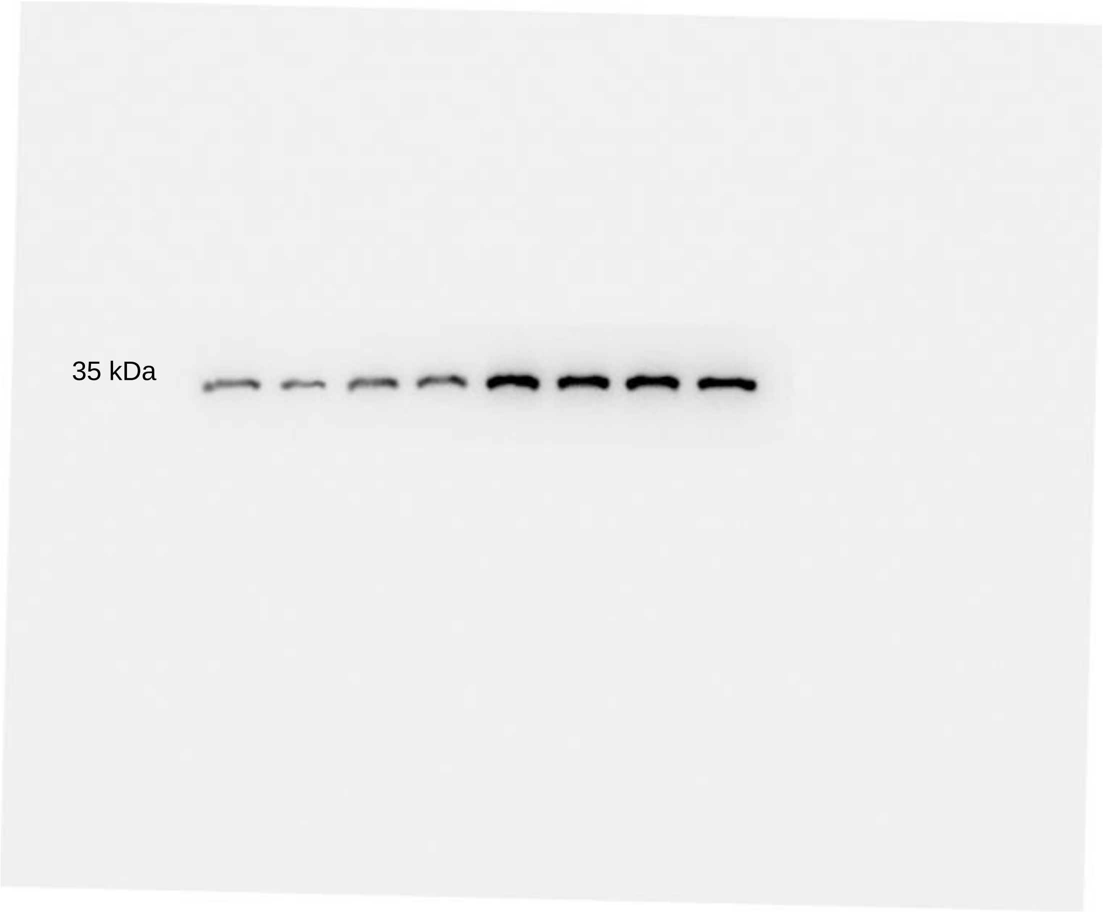

Fig 6G

IB: MARCHF5

Groups (HEY cells): EV (CHX: 0h, 1h, 2h, 4h), MARCHF5 (CHX: 0h, 1h, 2h, 4h)

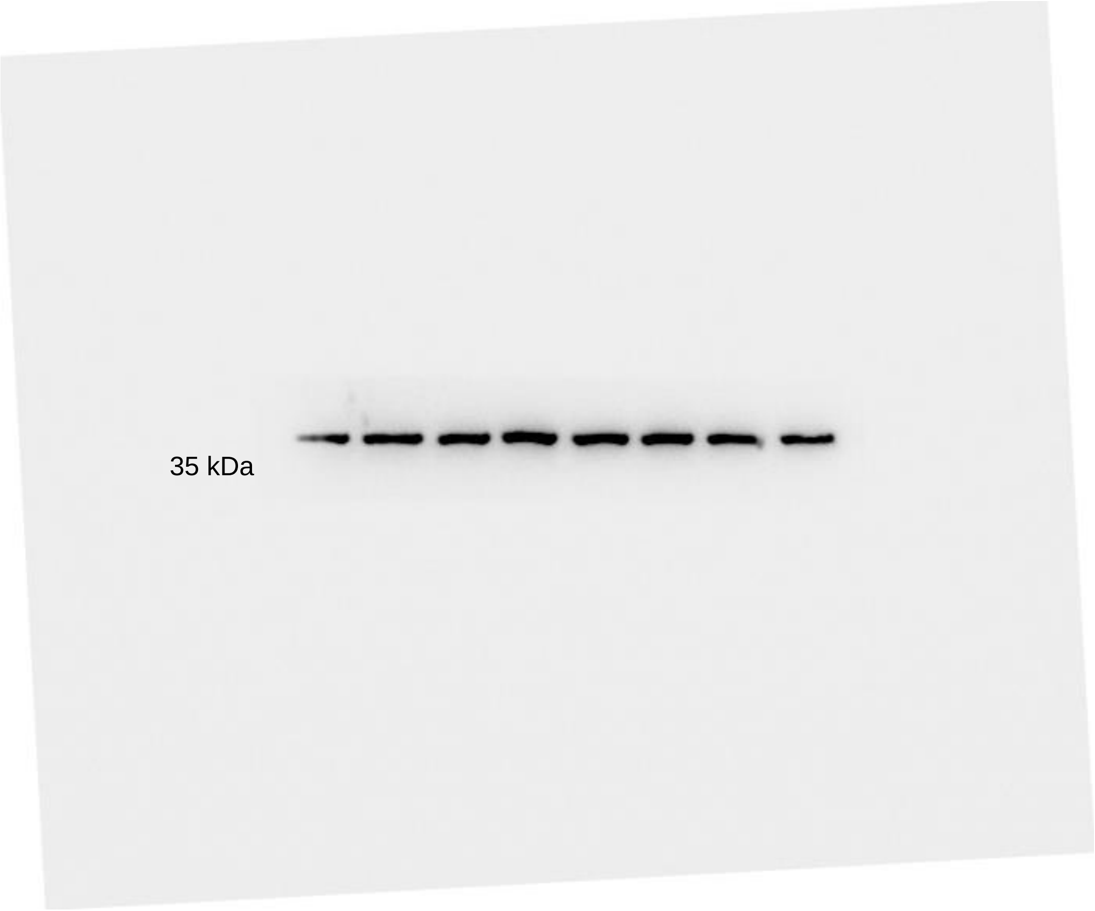

Fig 6G

IB: β-actin

Groups (HEY cells): EV (CHX: 0h, 1h, 2h, 4h), MARCHF5 (CHX: 0h, 1h, 2h, 4h)

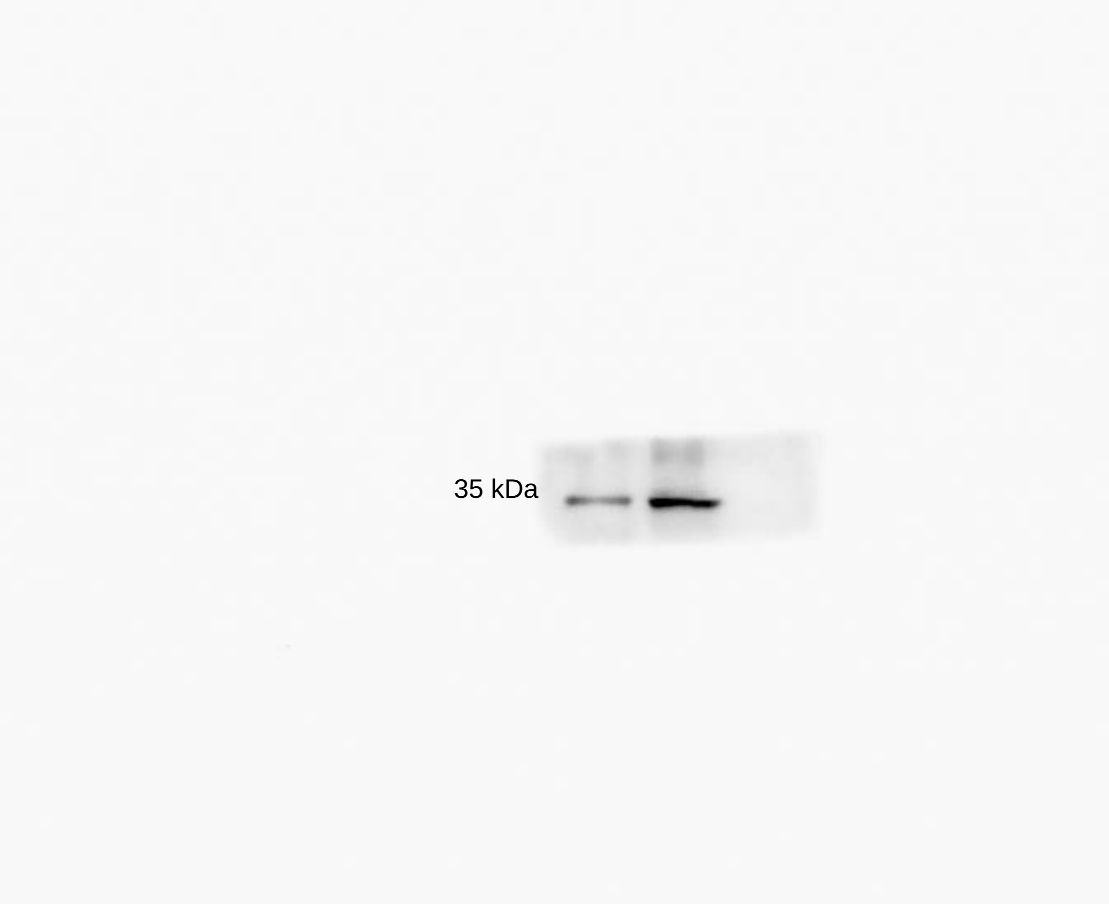

Fig 6H

IB: CACT

Groups (ES2 cells): shCtrl, shMARCH5

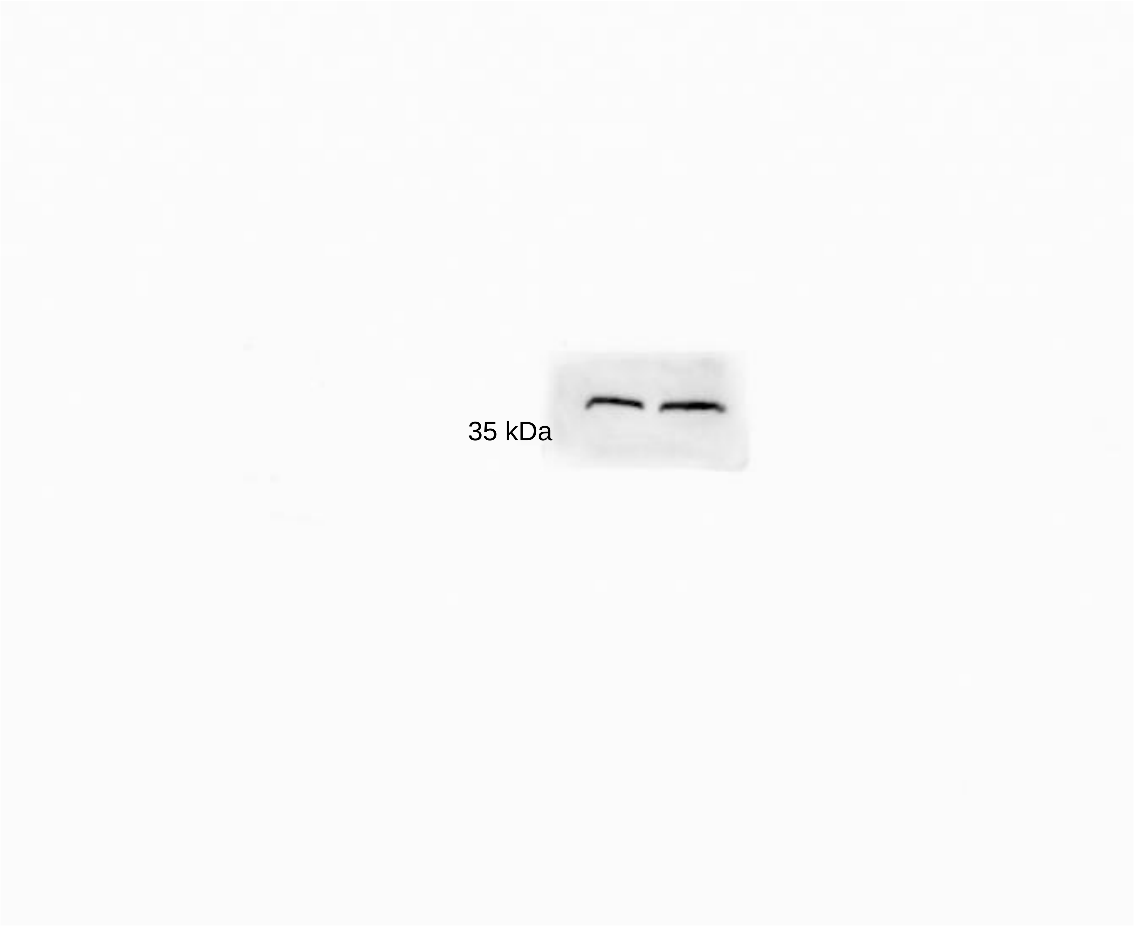

Fig 6H

IB: β-actin

Groups (ES2 cells): shCtrl, shMARCH5

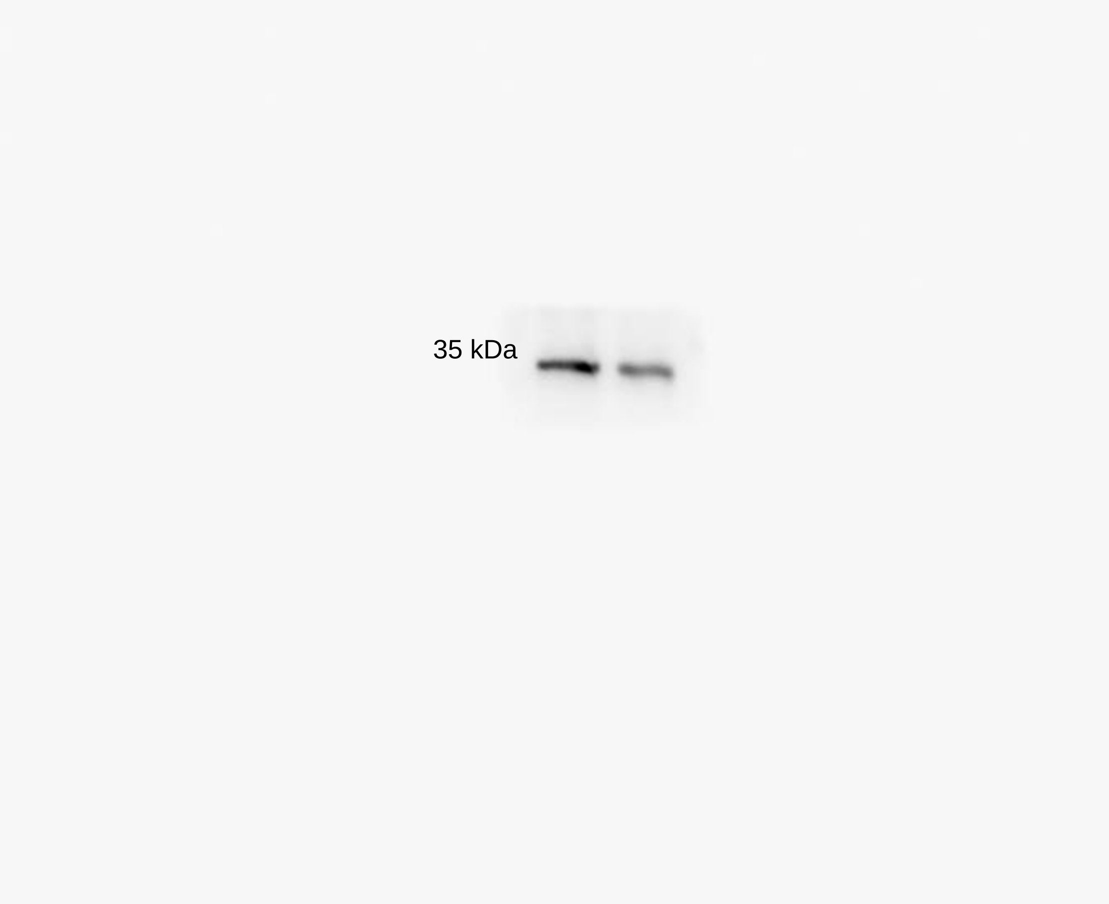

Fig 6H

IB: CACT

Groups (HEY cells): shCtrl, shMARCH5

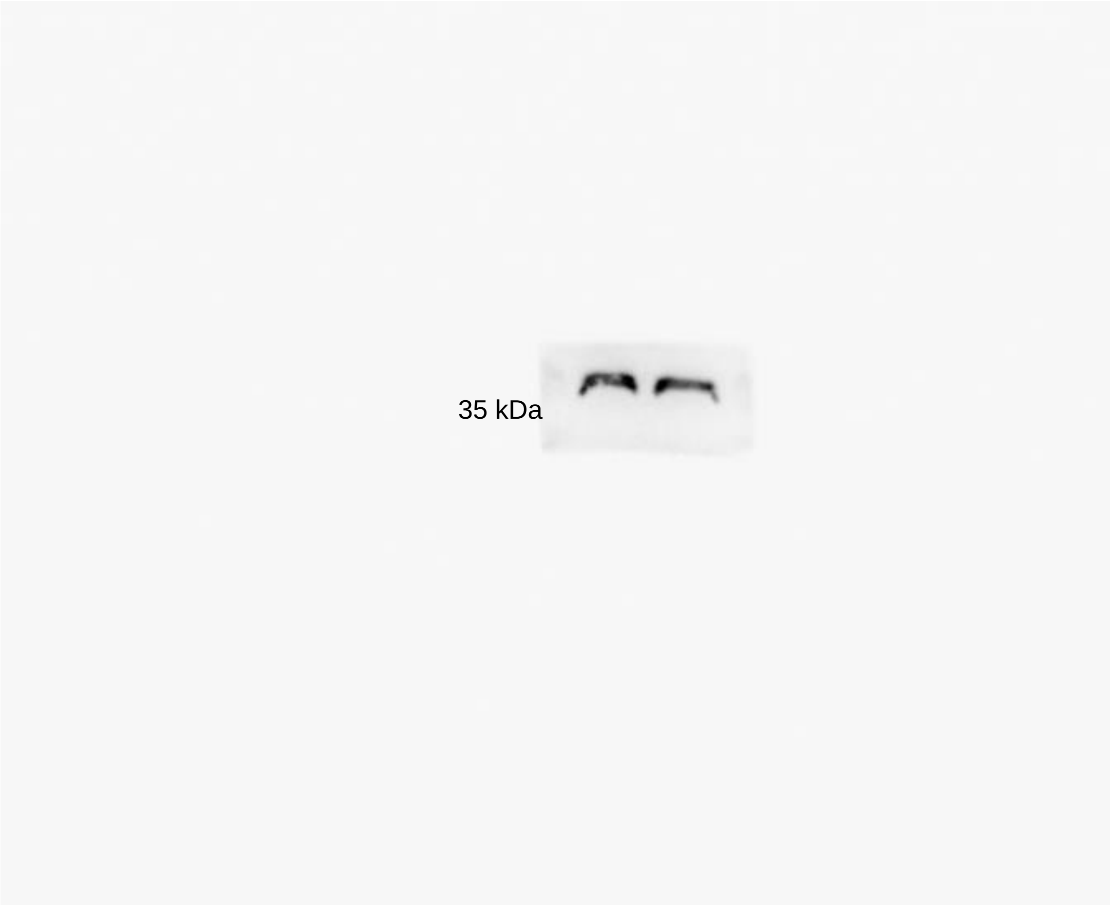

Fig 6H

IB: β-actin

Groups (HEY cells): shCtrl, shMARCH5

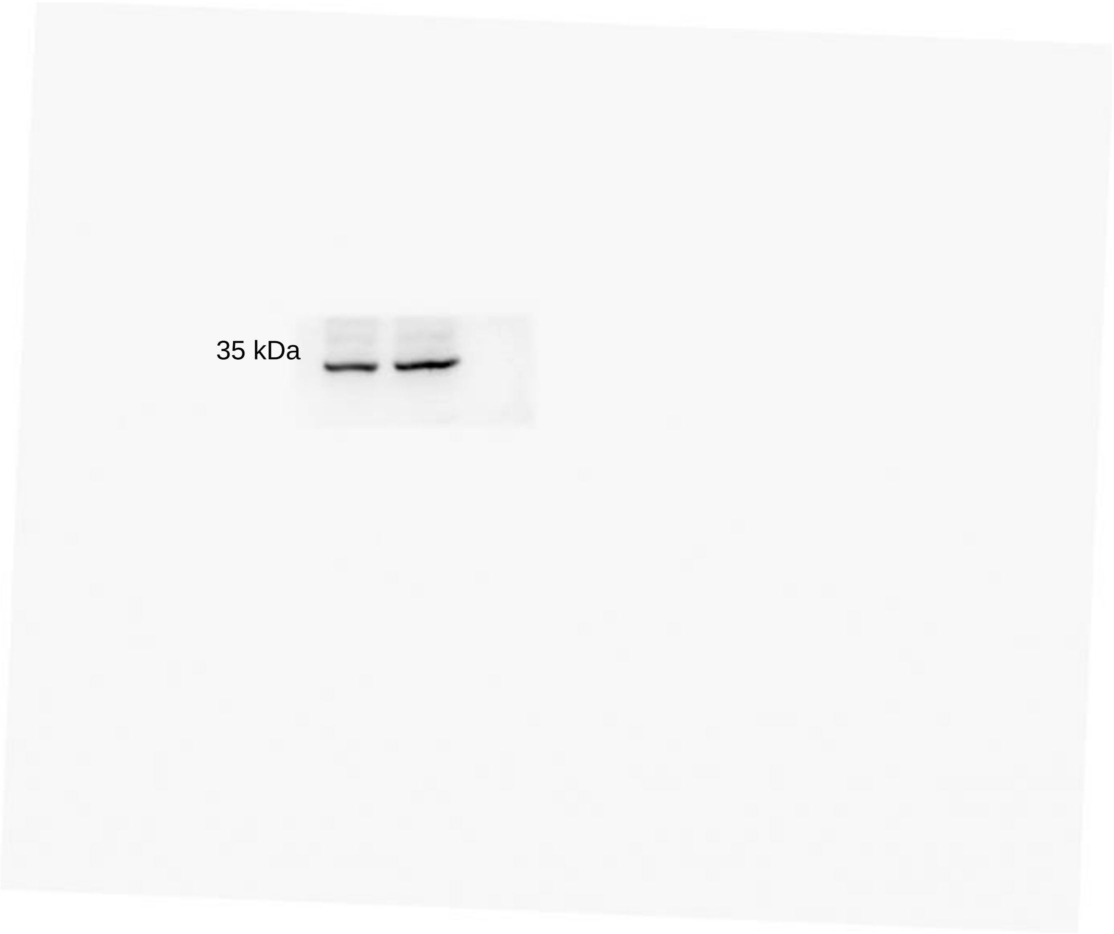

Fig 6I

IB: MARCHF5

Groups (ES2 cells): shCtrl, shSLC25A46

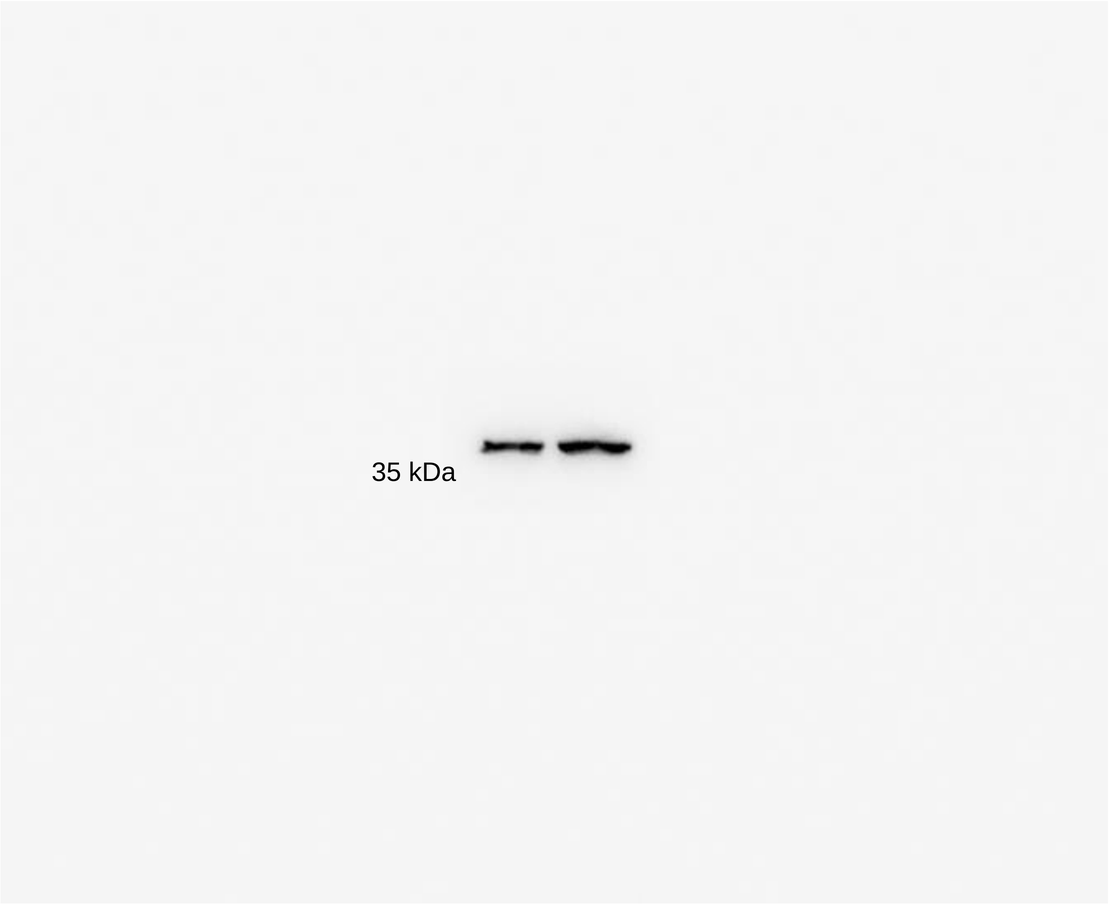

Fig 6I

IB: β-actin

Groups (ES2 cells): shCtrl, shSLC25A46

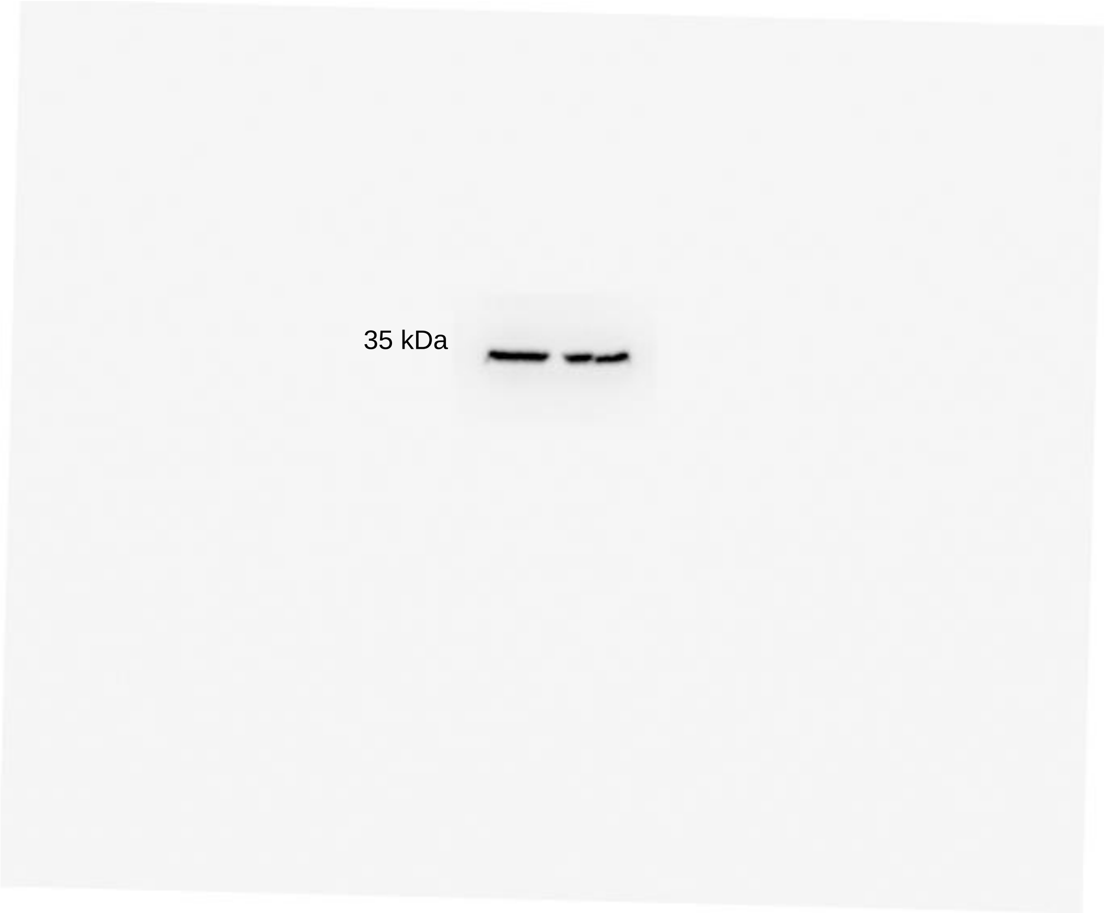

Fig 6I

IB: MARCHF5

Groups (HEY cells): EV, SLC25A46

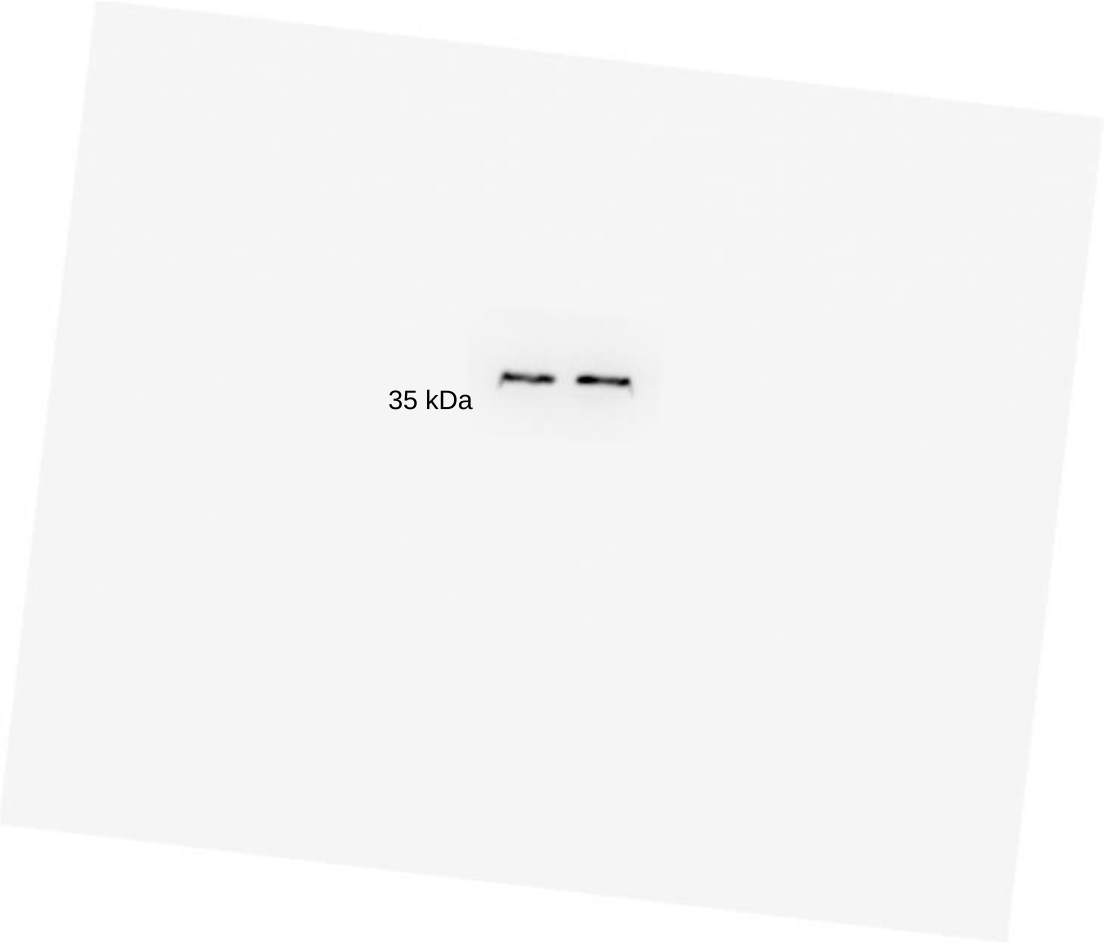

Fig 6I

IB: β-actin

Groups (HEY cells): EV, SLC25A46

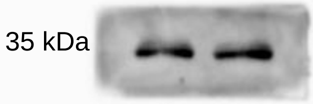

Fig 6J

IB: MARCHF5 (mito)

Groups (ES2 cells): shCtrl, shSLC25A46

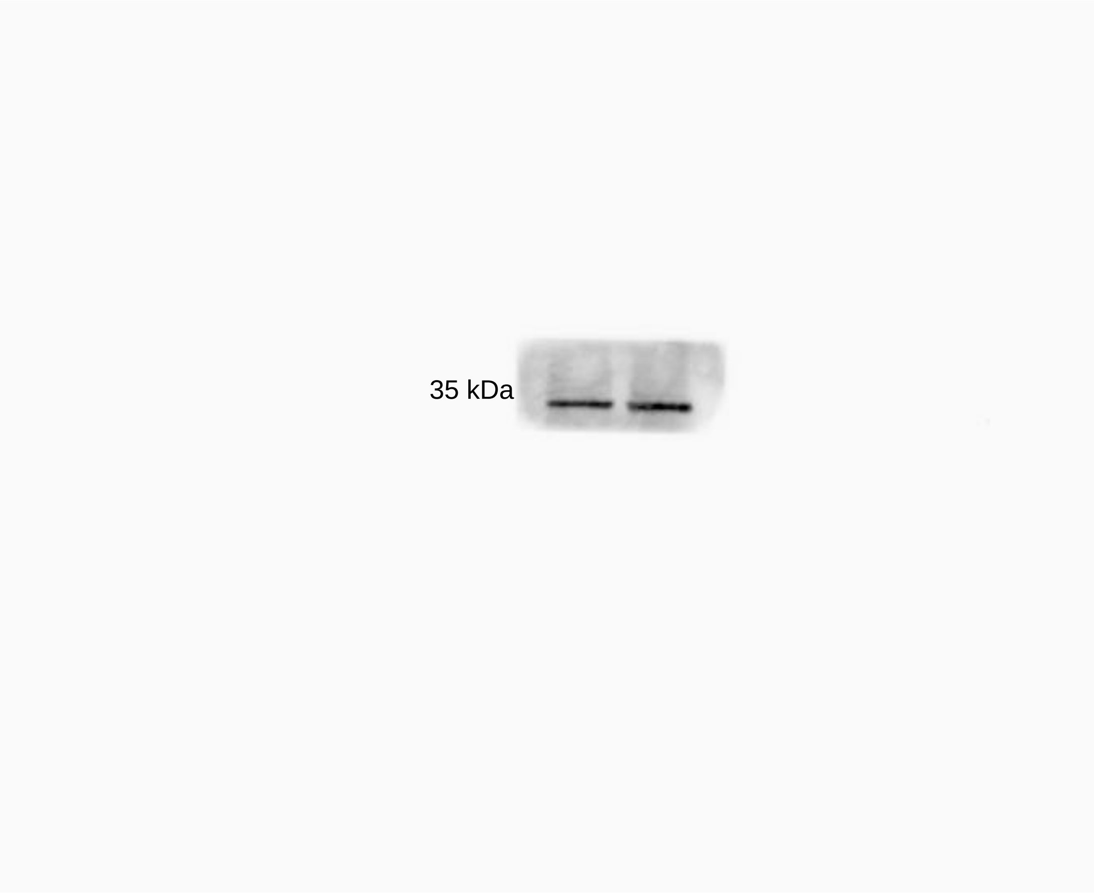

Fig 6J

IB: VDAC (mito)

Groups (ES2 cells): shCtrl, shSLC25A46

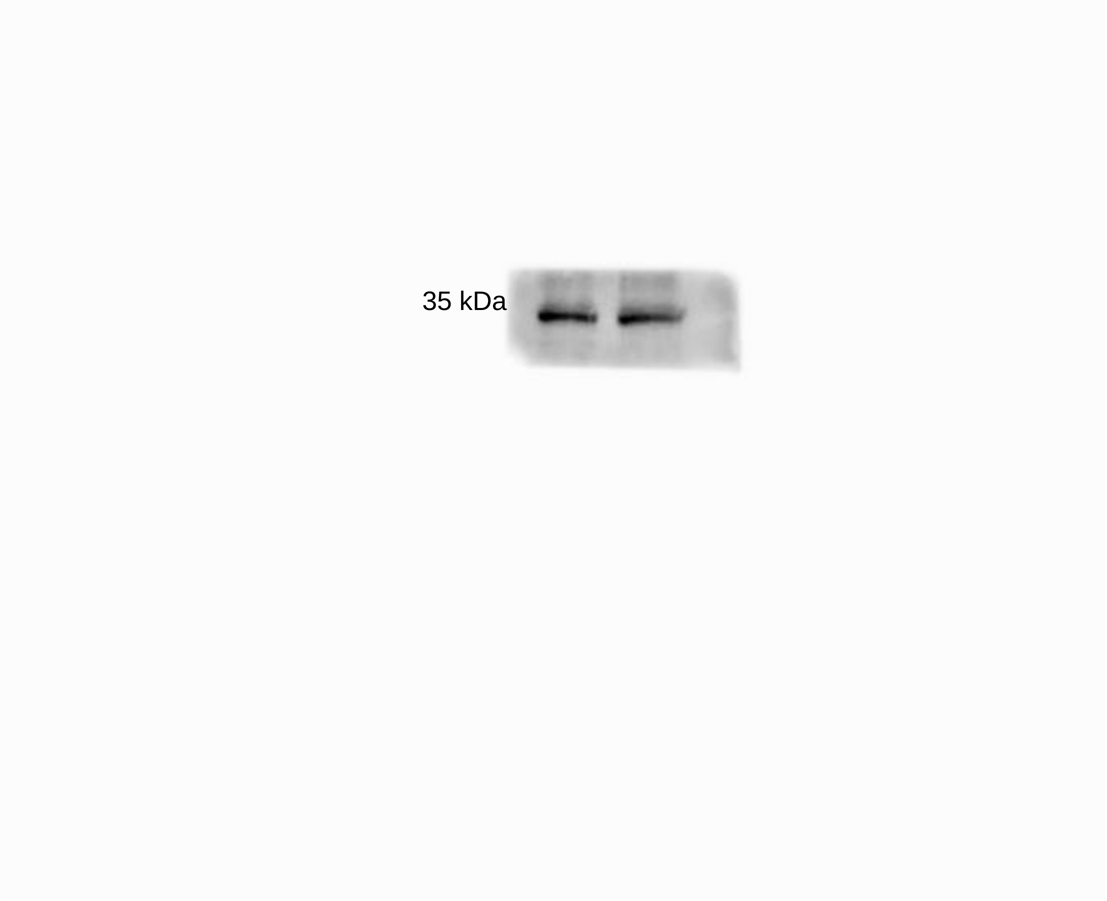

Fig 6J

IB: MARCHF5(mito)

Groups (HEY cells): EV, SLC25A46

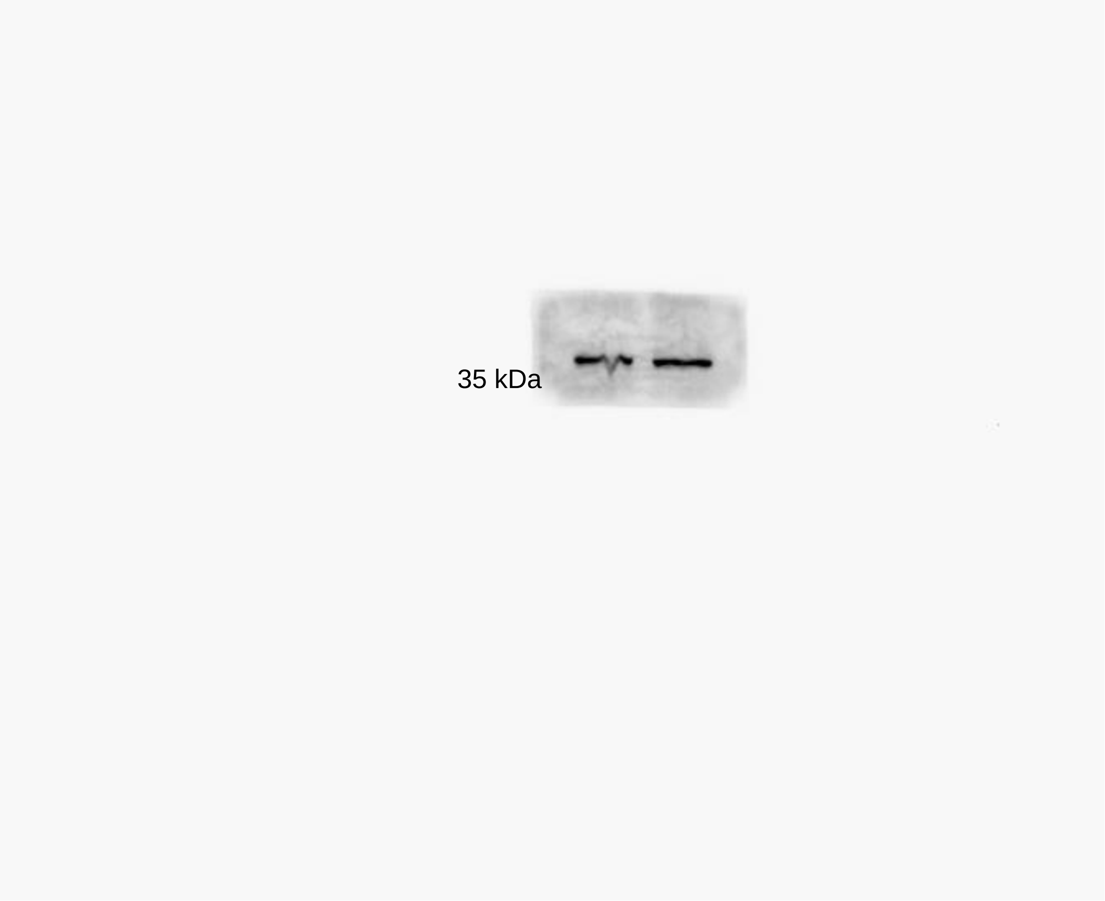

Fig 6J

IB: β-actin (mito)

Groups (HEY cells): EV, SLC25A46

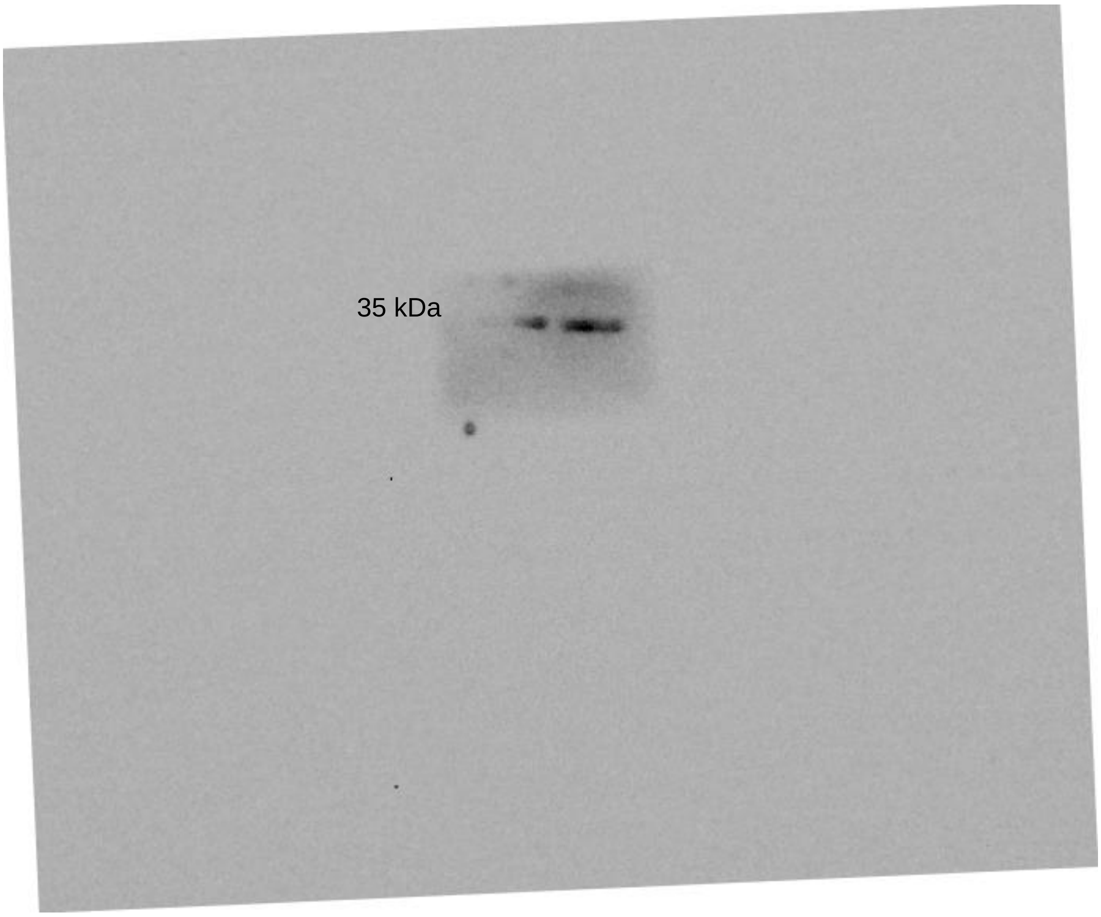

Fig 6K

IB: MARCHF5

Groups (ES2 cells): shCtrl+MG132, shSLC25A46+MG132

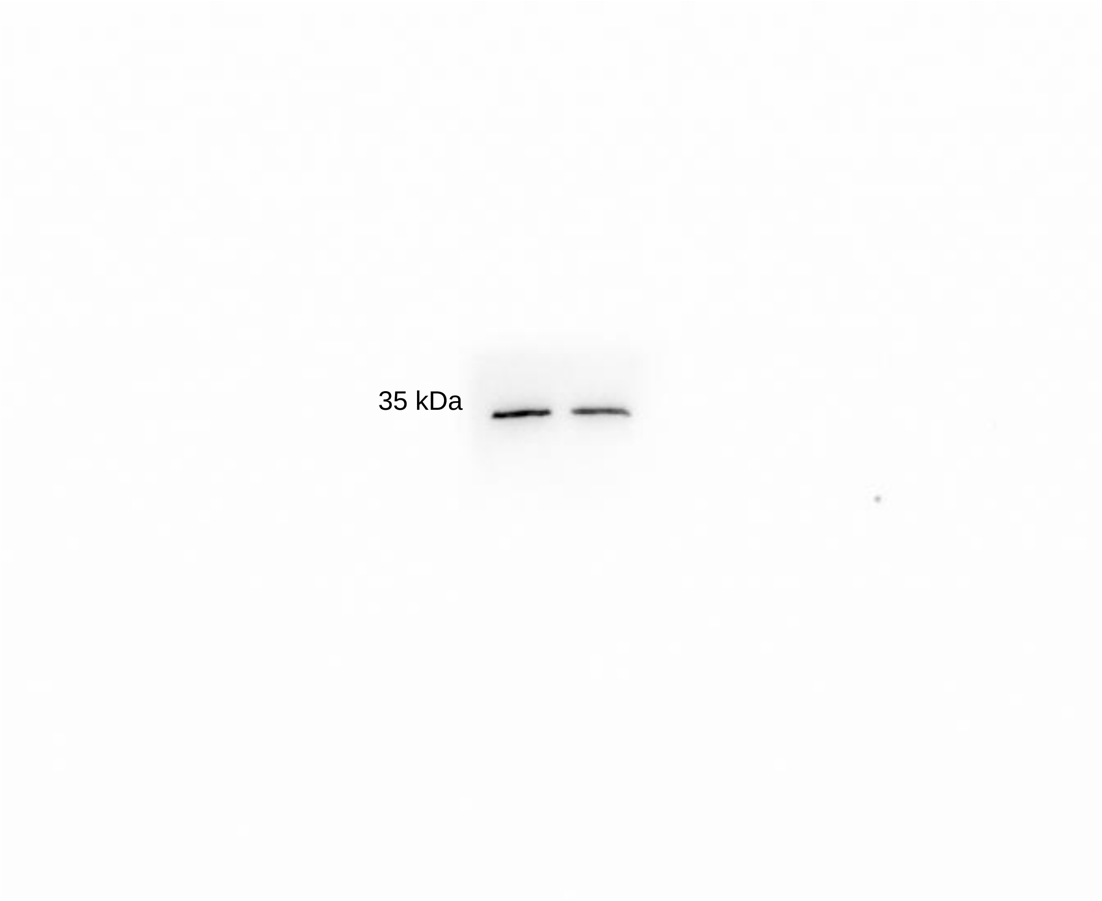

Fig 6J

IB: CACT

Groups (ES2 cells): shCtrl+MG132, shSLC25A46+MG132

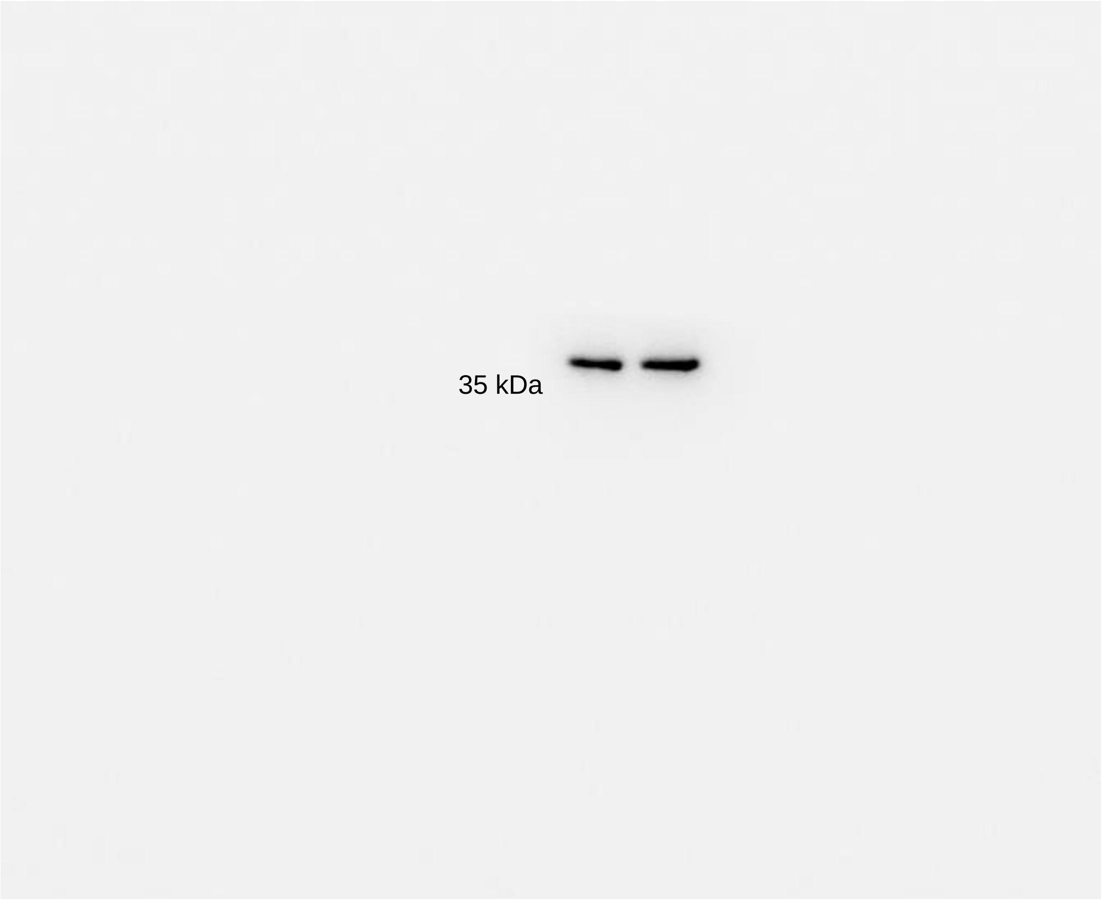

Fig 6K

IB: β-actin

Groups (ES2 cells): shCtrl+MG132, shSLC25A46+MG132

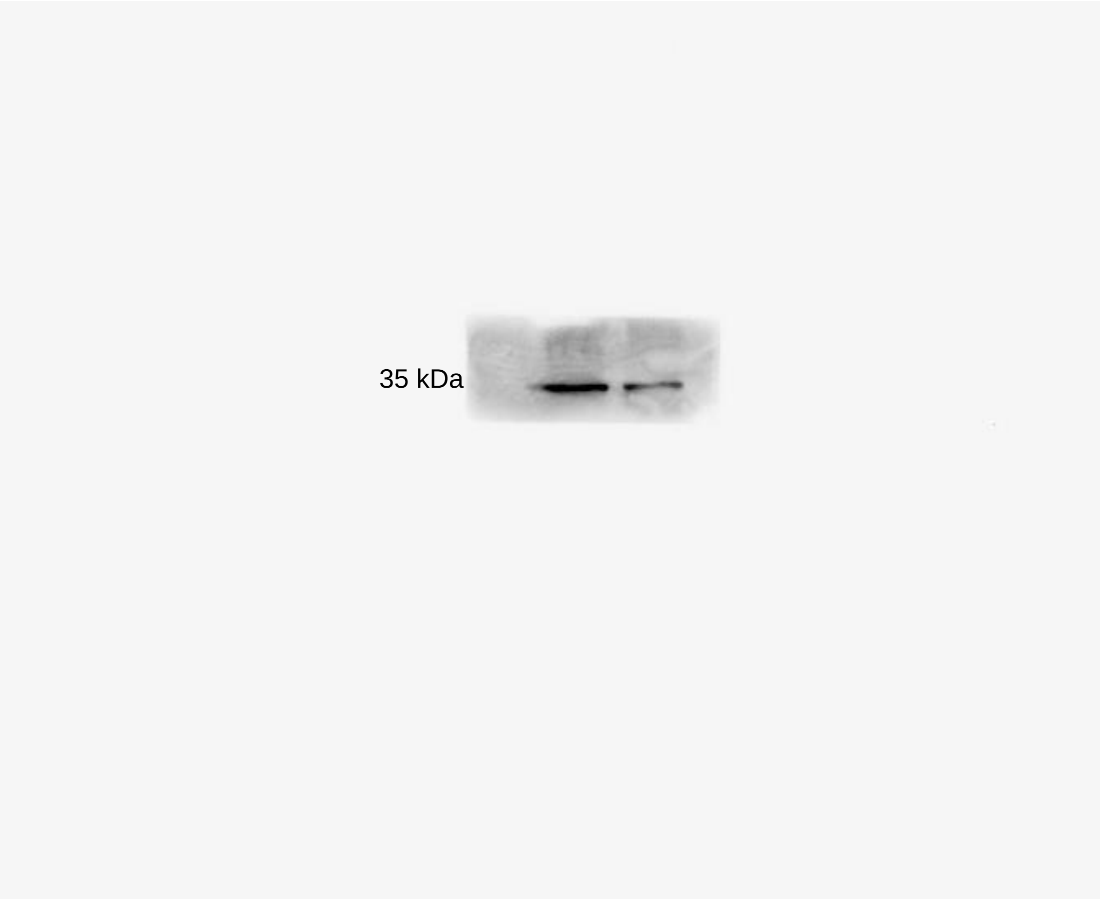

Fig 6K

IB: MARCHF5

Groups (HEY cells): EV+MG132, EV+MG132

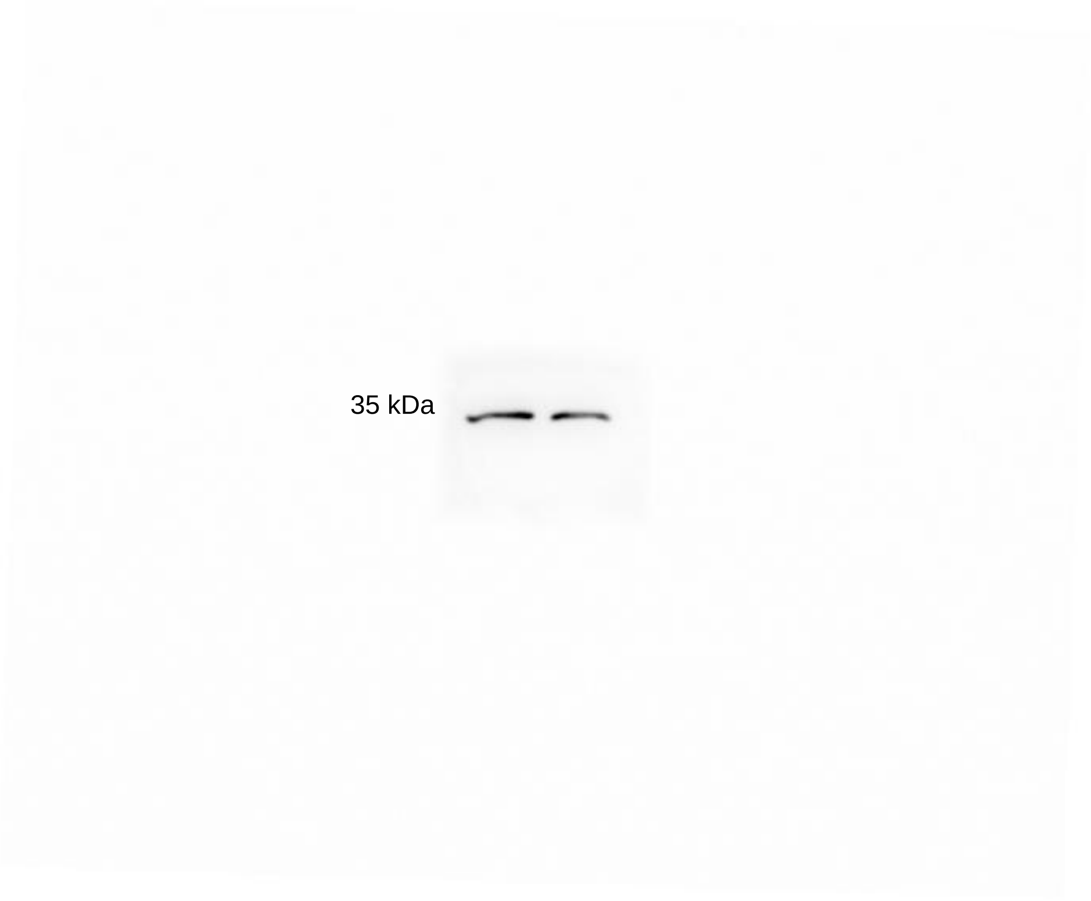

Fig 6K

IB: CACT

Groups (HEY cells): EV+MG132, EV+MG132**Fig 6J**

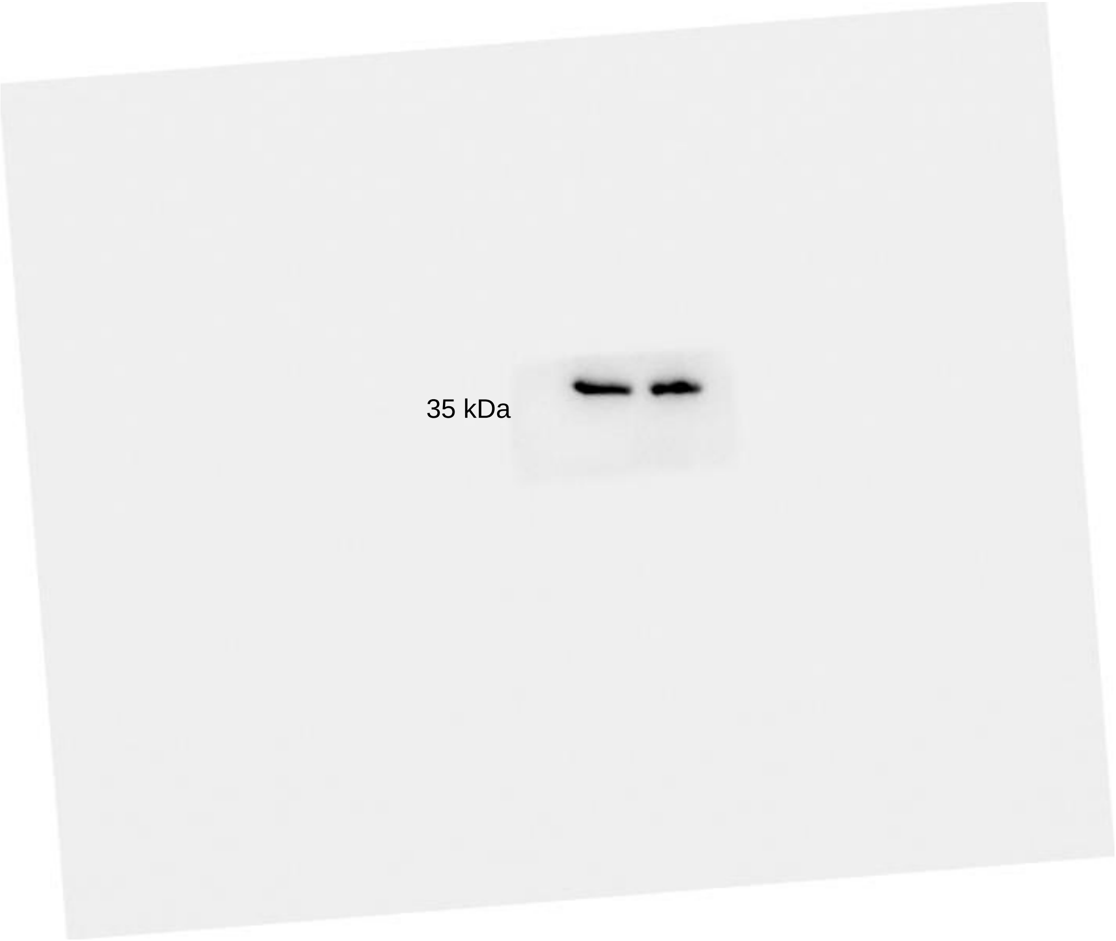

Fig 6K

IB: β-actin

Groups (HEY cells): EV+MG132, EV+MG132

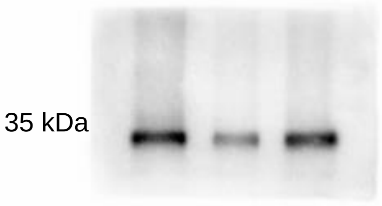

Fig 6L

IB: CACT

Groups (ES2 cells): shCtrl, shSLC25A46, shSLC25A46+shMARCHF5

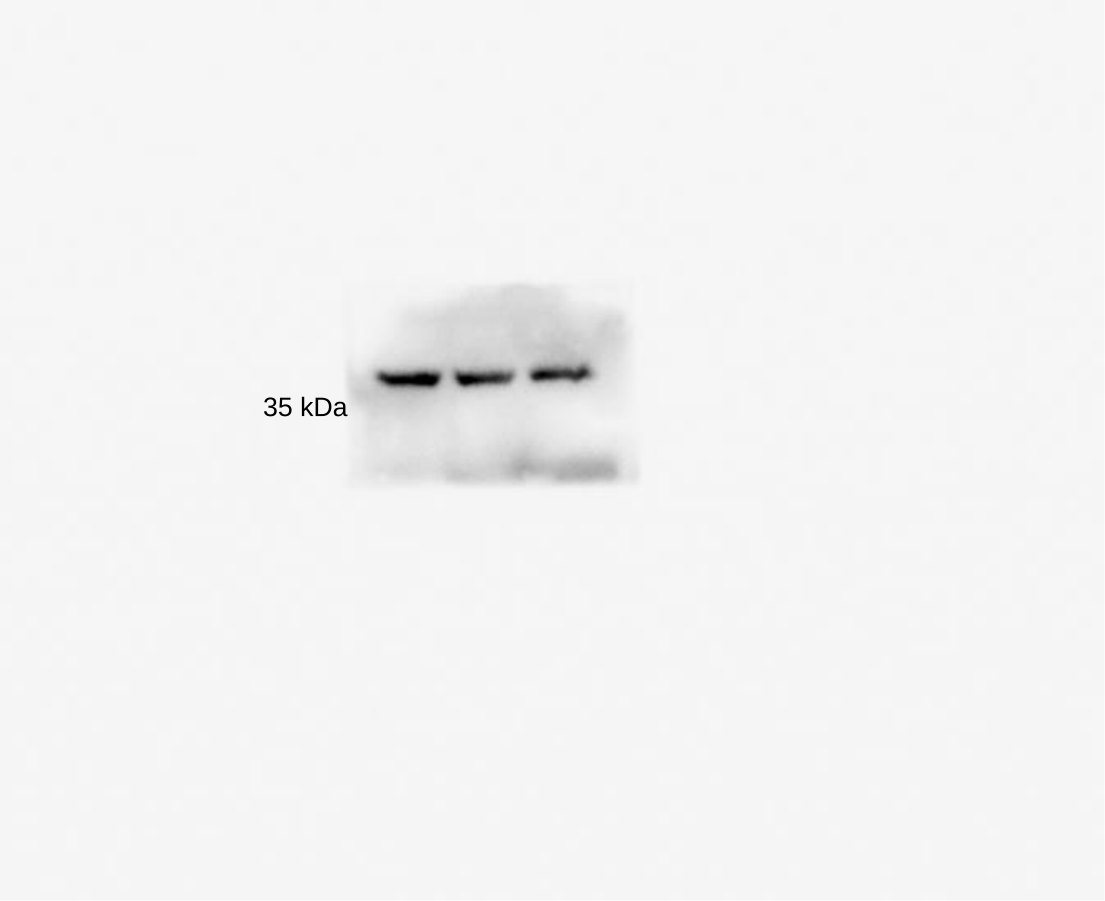

Fig 6L

IB: β-actin

Groups (ES2 cells): shCtrl, shSLC25A46, shSLC25A46+shMARCHF5

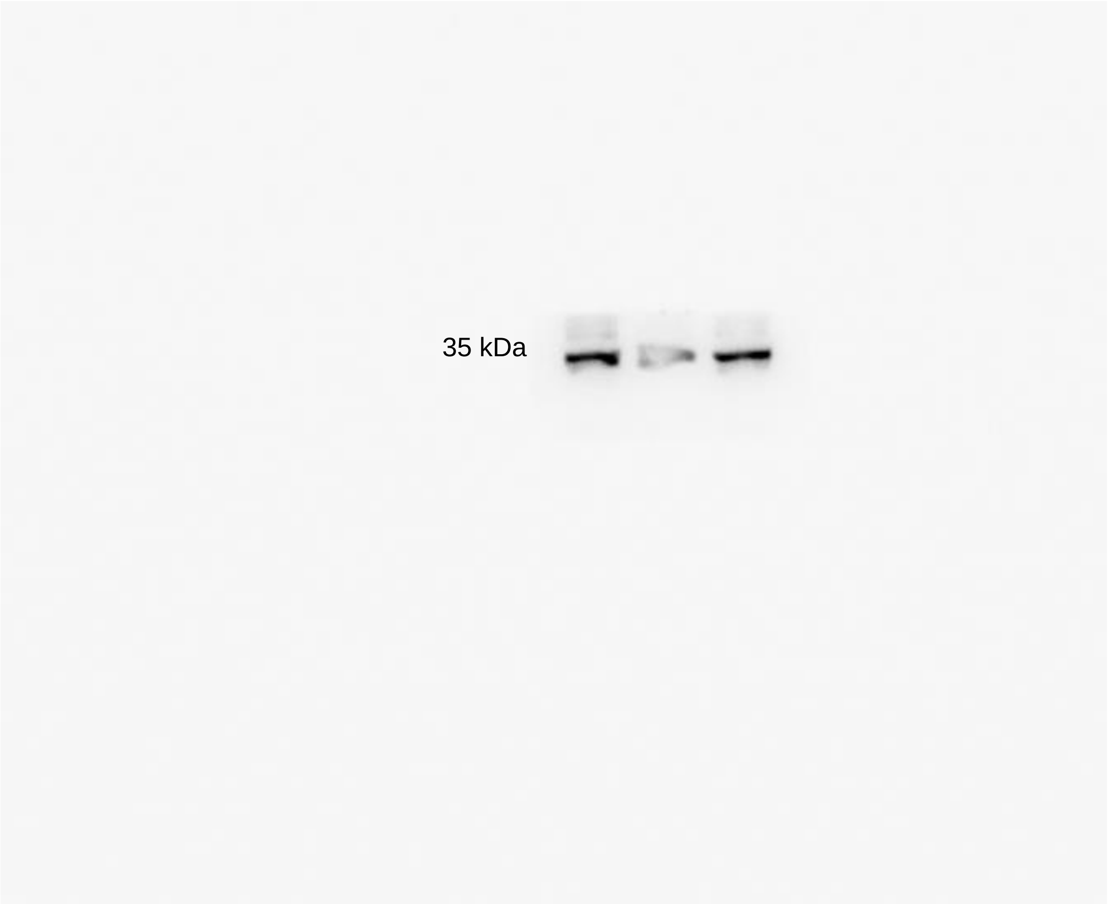

Fig 6L

IB: CACT

Groups (SKOV3 cells): shCtrl, shSLC25A46, shSLC25A46+shMARCHF5

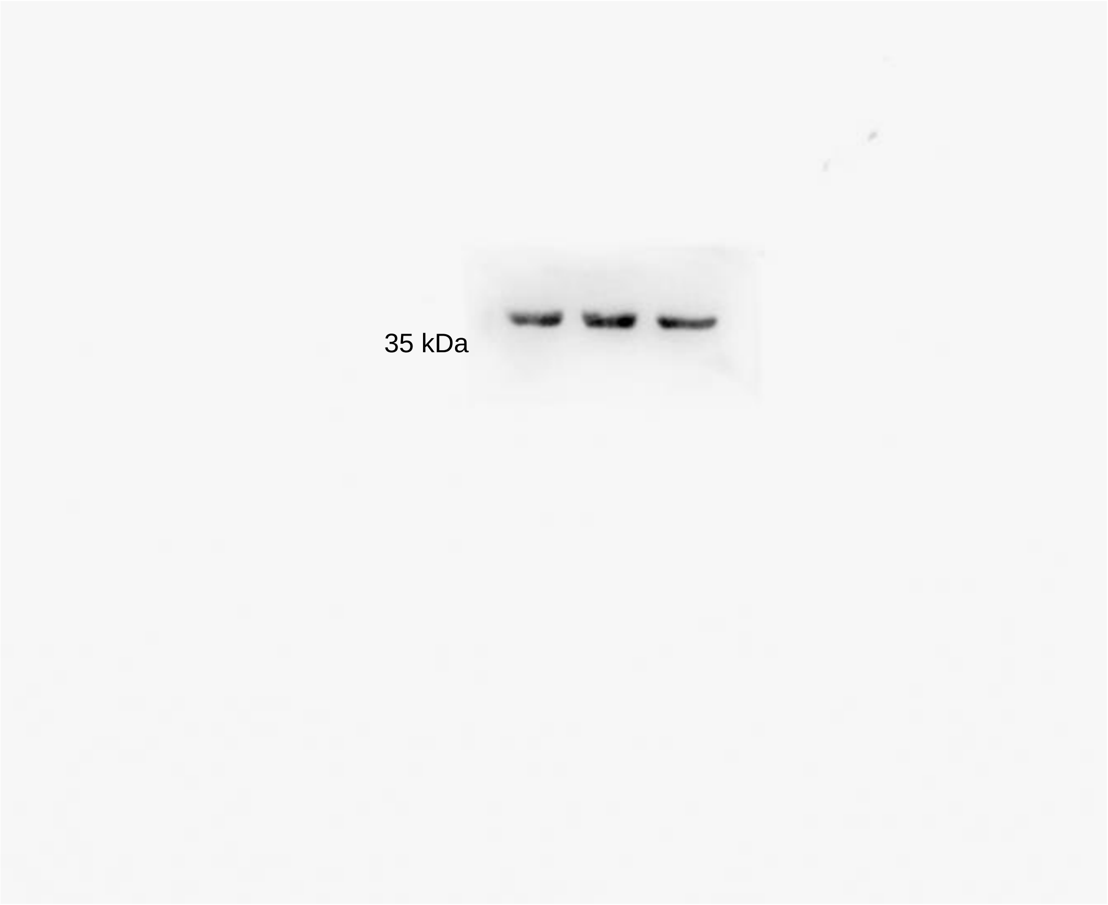

Fig 6L

IB: β-actin

Groups (SKOV3 cells): shCtrl, shSLC25A46, shSLC25A46+shMARCHF5

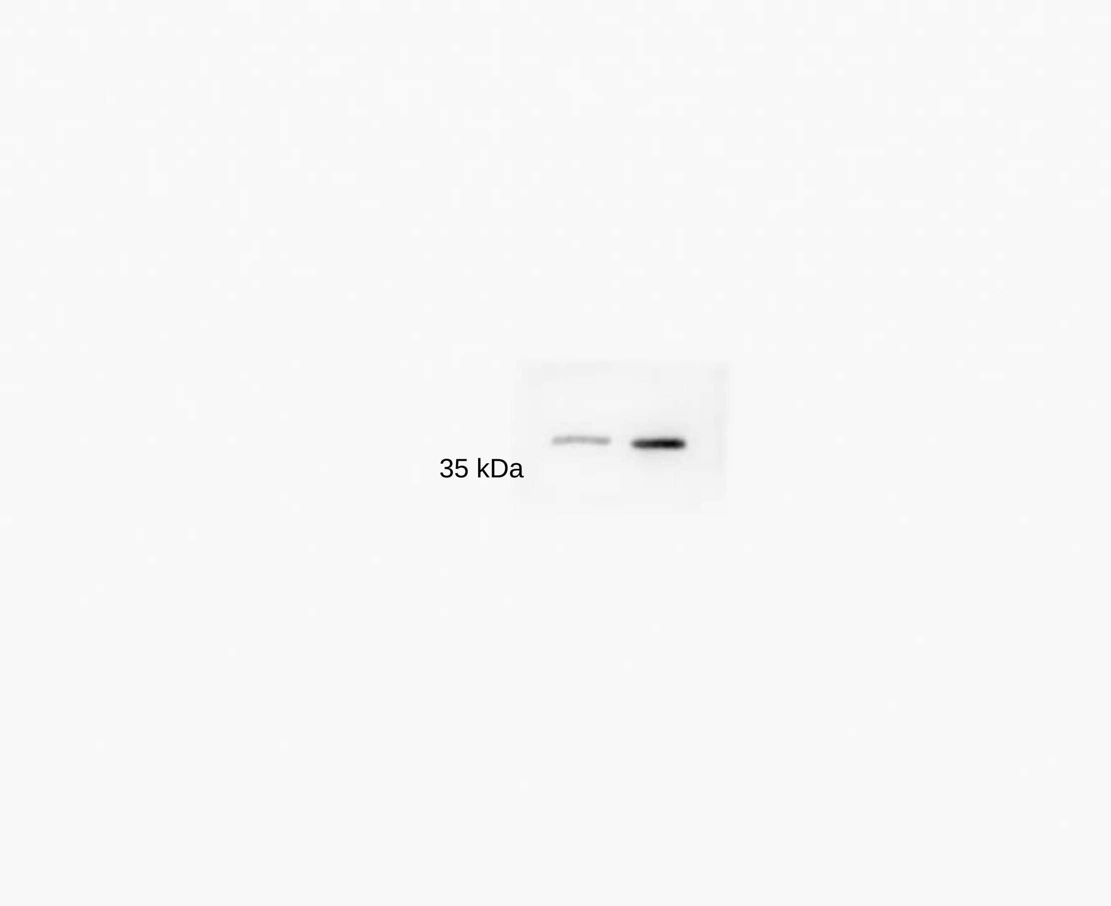

Fig 8K

IB: SLC25A46

Groups (HEY cells): Parental, Resistant

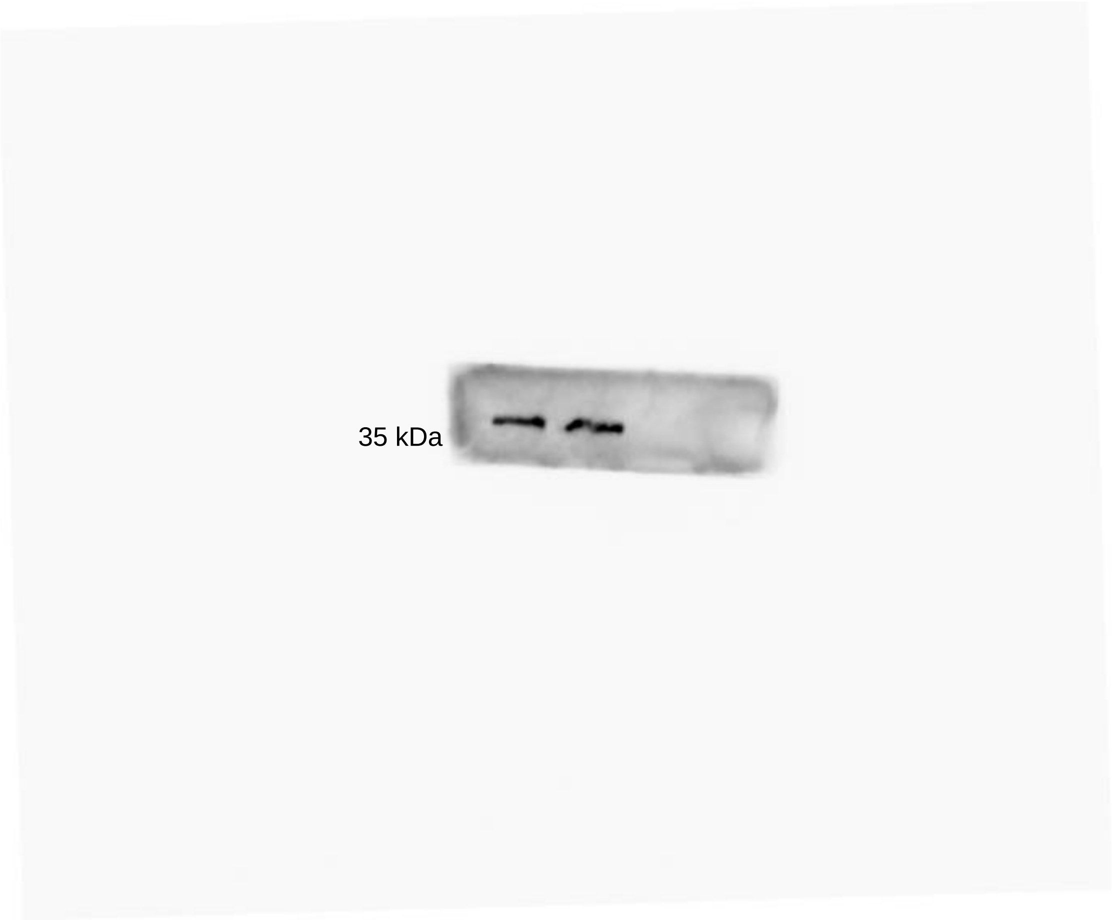

Fig 8K

IB: β-actin

Groups (HEY cells): Parental, Resistant

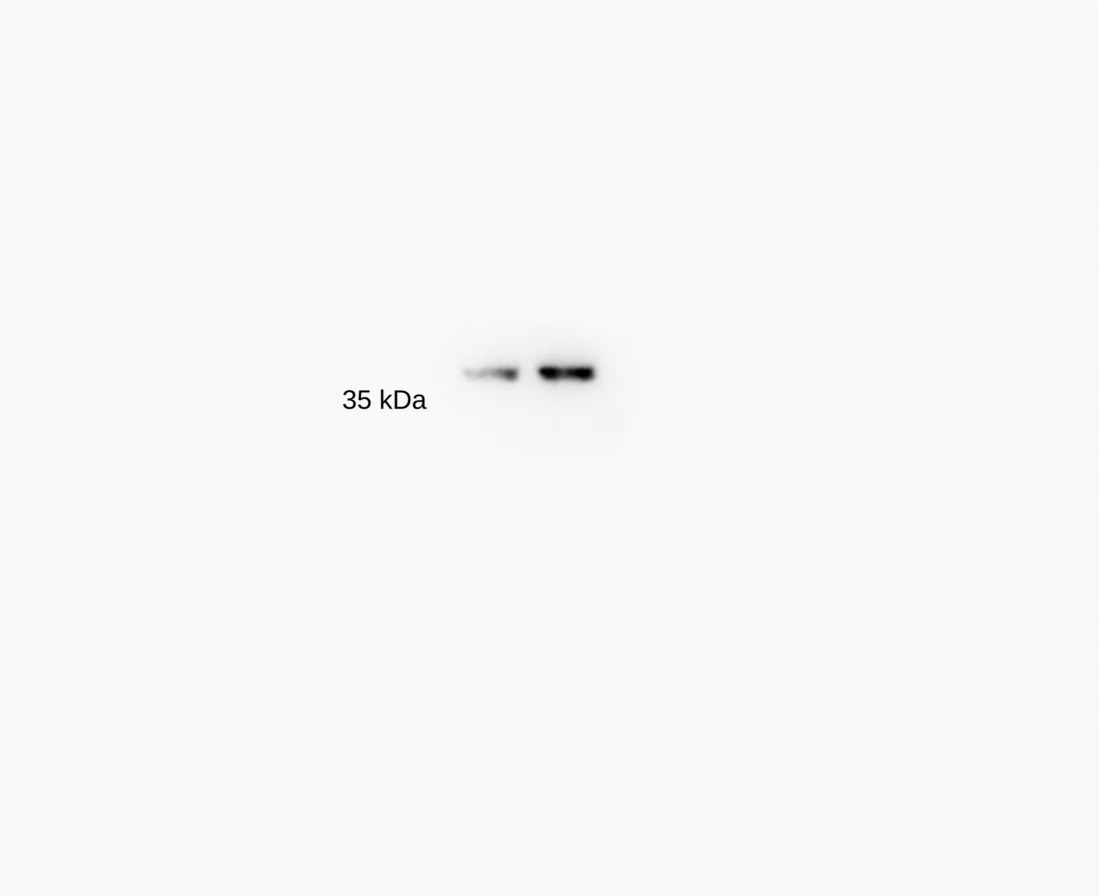

Fig 8K

IB: SLC25A46

Groups (SKOV3 cells): Parental, Resistant

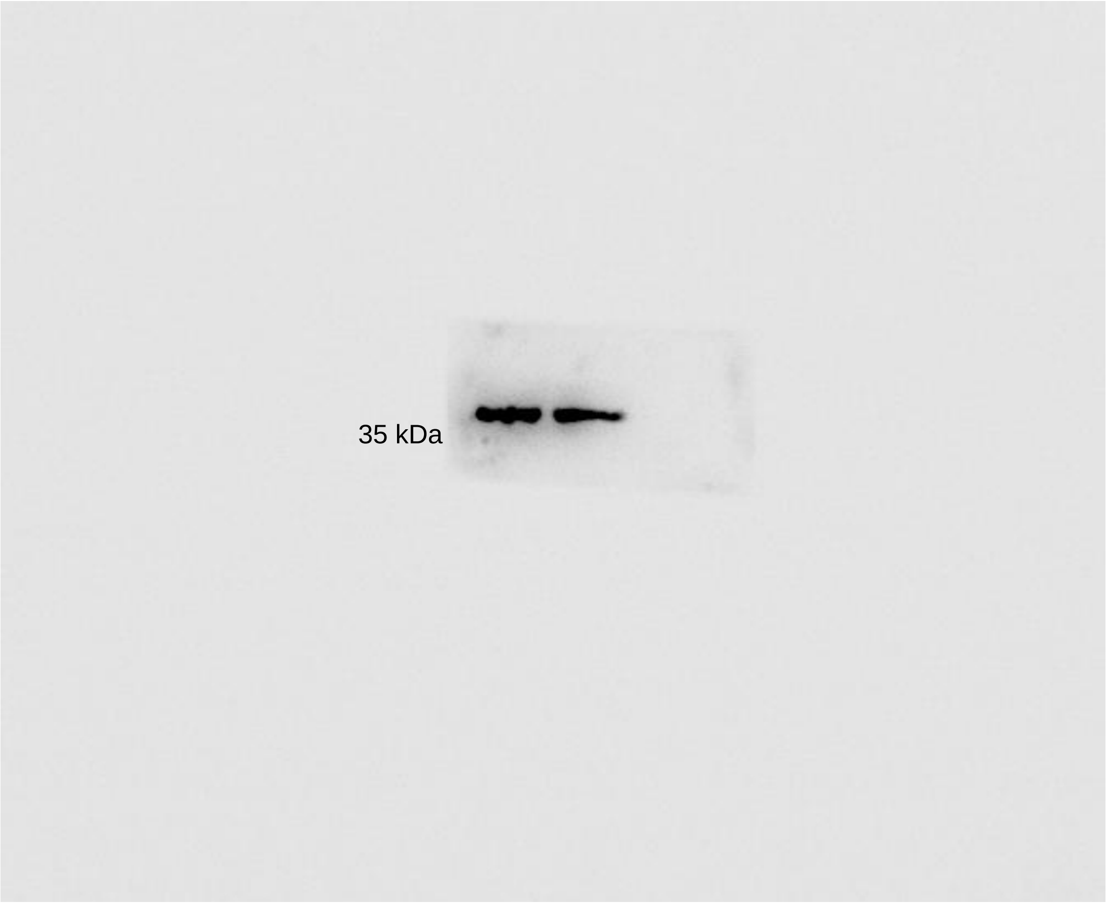

Fig 8K

IB: β-actin

Groups (SKOV3 cells): Parental, Resistant

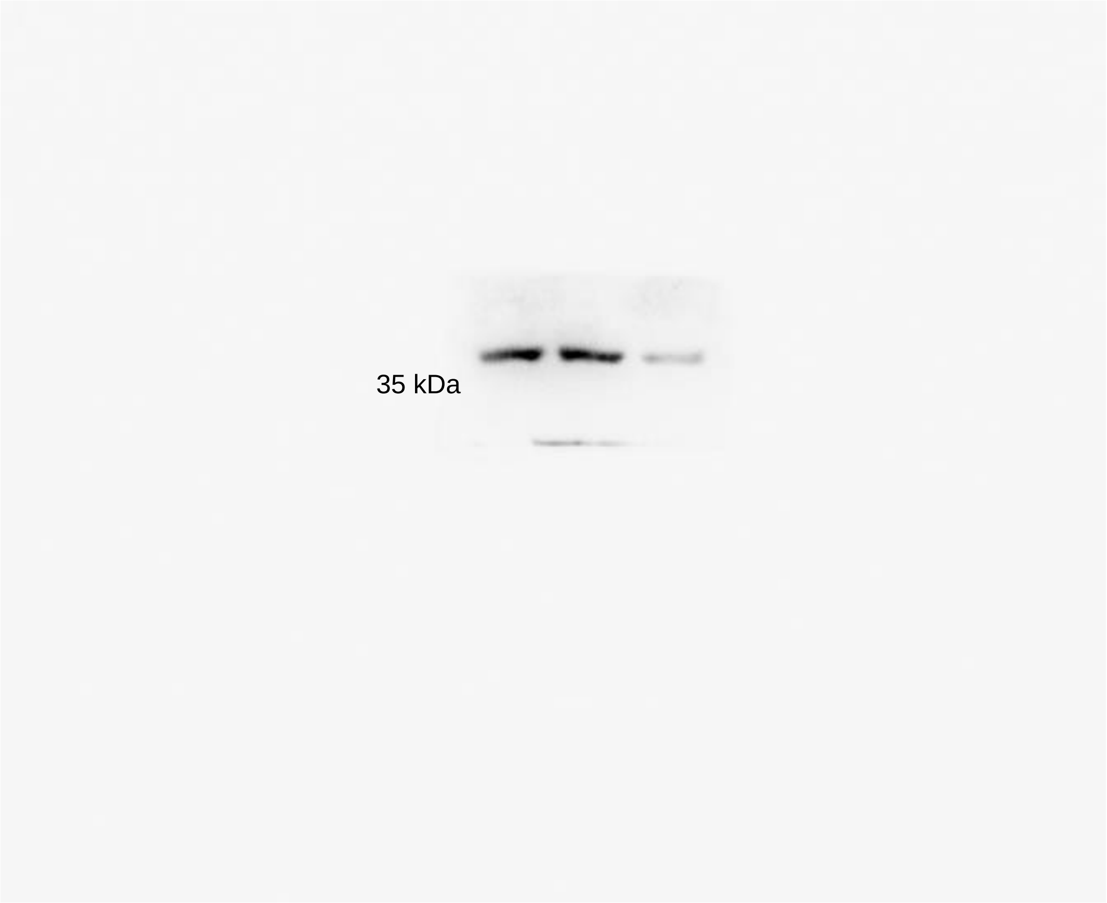

Fig 9D

IB: SLC25A46

Groups (ES2 cells): shCtrl, shHOXB13, shPBX1

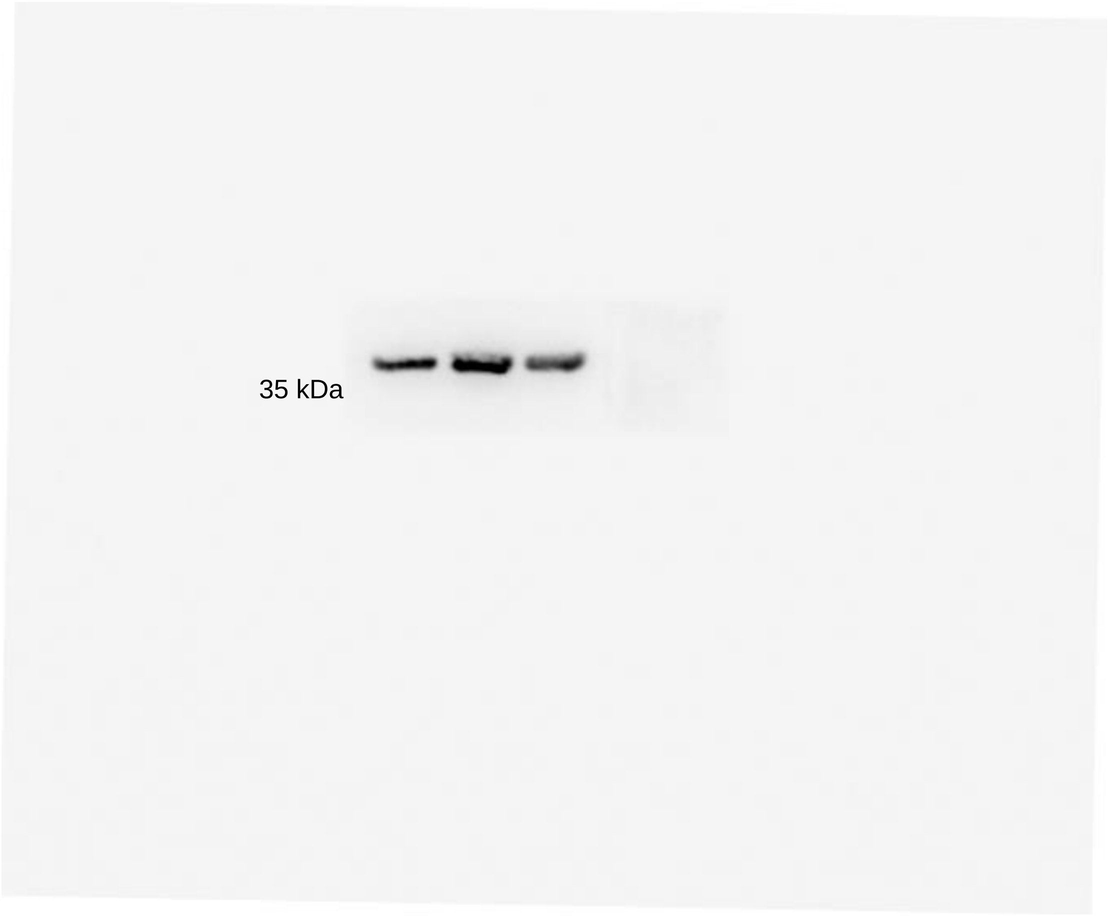

Fig 9D

IB: β-actin

Groups (ES2 cells): shCtrl, shHOXB13, shPBX1

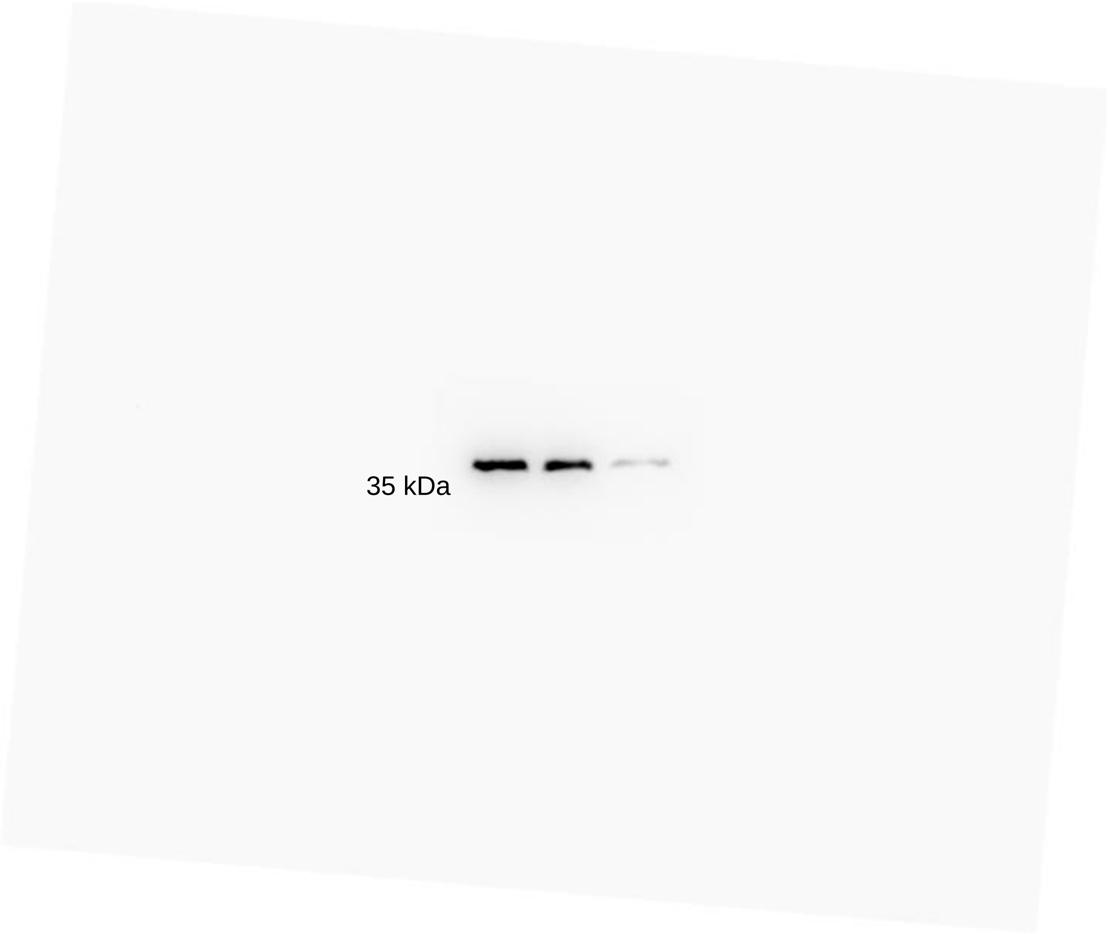

Fig 9D

IB: SLC25A46

Groups (SKOV3 cells): shCtrl, shHOXB13, shPBX1

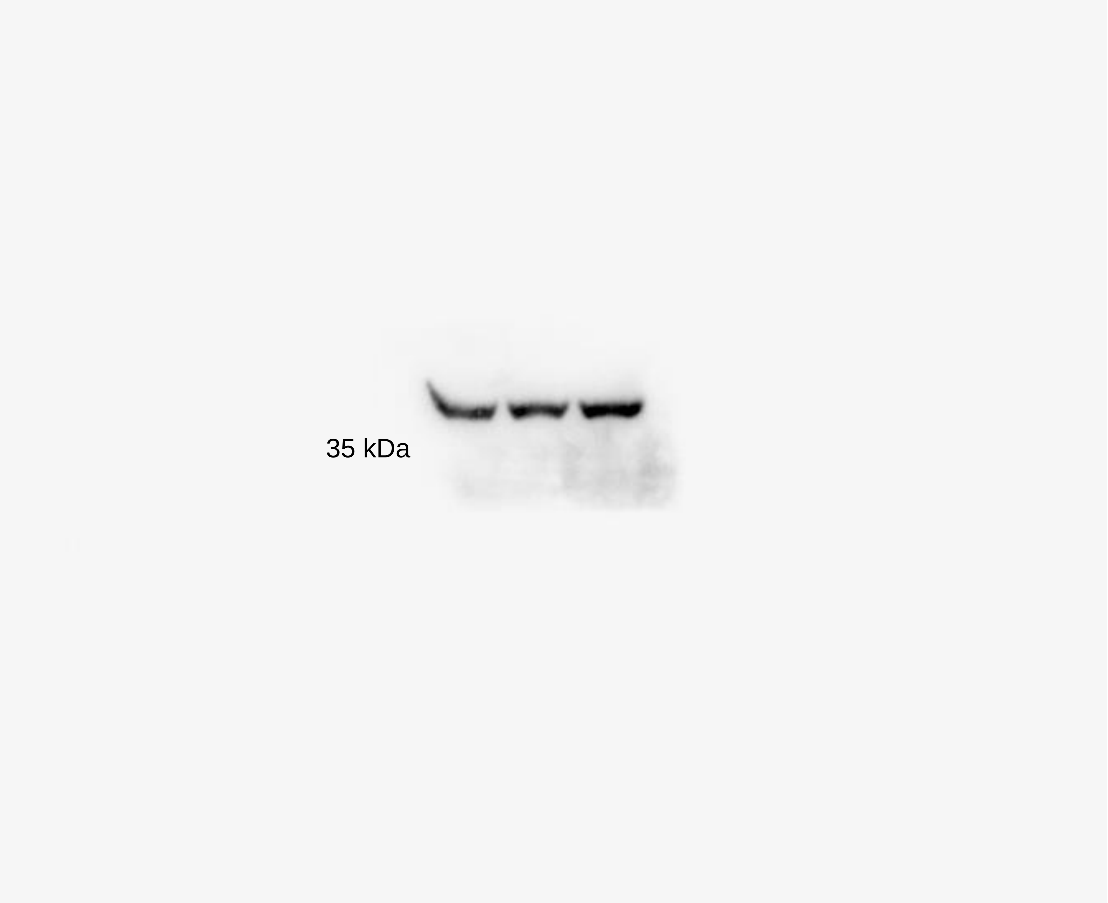

Fig 9D

IB: β-actin

Groups (SKOV3 cells): shCtrl, shHOXB13, shPBX1
